# Current Status of Newborn Bloodspot Screening Worldwide 2024: A Comprehensive Review of Recent Activities (2020–2023)

**DOI:** 10.3390/ijns10020038

**Published:** 2024-05-23

**Authors:** Bradford L. Therrell, Carmencita D. Padilla, Gustavo J. C. Borrajo, Issam Khneisser, Peter C. J. I. Schielen, Jennifer Knight-Madden, Helen L. Malherbe, Marika Kase

**Affiliations:** 1 Department of Pediatrics, University of Texas Health Science Center San Antonio, San Antonio, TX 78229, USA; 2 National Newborn Screening and Global Resource Center, Austin, TX 78759, USA; 3 Department of Pediatrics, College of Medicine, University of the Philippines Manila, Manila 1000, Philippines; cdpadilla@up.edu.ph; 4 Detección de Errores Congénitos—Fundación Bioquímica Argentina, La Plata 1908, Argentina; borrajog@net-alliance.net; 5 Jacques LOISELET Genetic and Genomic Medical Center, Faculty of Medicine, Saint Joseph University, Beirut 1104 2020, Lebanon; issam.khneisser@usj.edu.lb; 6 Office of the International Society for Neonatal Screening, Reigerskamp 273, 3607 HP Maarssen, The Netherlands; peter.schielen@isns-neoscreening.org; 7 Caribbean Institute for Health Research—Sickle Cell Unit, The University of the West Indies, Mona, Kingston 7, Jamaica; jennifer.knightmadden@uwimona.edu.jm; 8 Centre for Human Metabolomics, North-West University, Potchefstroom 2531, South Africa; research@rarediseases.co.za; 9 Rare Diseases South Africa NPC, The Station Office, Bryanston, Sandton 2021, South Africa; 10 Strategic Initiatives Reproductive Health, Revvity, PL10, 10101 Turku, Finland; marika.kase@revvity.com

**Keywords:** newborn screening, global, international data, North America, Asia–Pacific, Latin America, Europe, Middle East North Africa, Sub-Saharan Africa, Caribbean

## Abstract

Newborn bloodspot screening (NBS) began in the early 1960s based on the work of Dr. Robert “Bob” Guthrie in Buffalo, NY, USA. His development of a screening test for phenylketonuria on blood absorbed onto a special filter paper and transported to a remote testing laboratory began it all. Expansion of NBS to large numbers of asymptomatic congenital conditions flourishes in many settings while it has not yet been realized in others. The need for NBS as an efficient and effective public health prevention strategy that contributes to lowered morbidity and mortality wherever it is sustained is well known in the medical field but not necessarily by political policy makers. Acknowledging the value of national NBS reports published in 2007, the authors collaborated to create a worldwide NBS update in 2015. In a continuing attempt to review the progress of NBS globally, and to move towards a more harmonized and equitable screening system, we have updated our 2015 report with information available at the beginning of 2024. Reports on sub-Saharan Africa and the Caribbean, missing in 2015, have been included. Tables popular in the previous report have been updated with an eye towards harmonized comparisons. To emphasize areas needing attention globally, we have used regional tables containing similar listings of conditions screened, numbers of screening laboratories, and time at which specimen collection is recommended. Discussions are limited to bloodspot screening.

## 1. Introduction

Regarding the beginning of newborn bloodspot screening (NBS), Dr. Robert “Bob” Guthrie, the “Father” of newborn bloodspot screening (NBS) reported that “*… screening had its origin in Jamestown, New York in 1961*” when he began receiving specimens from two Jamestown hospitals soon after giving a talk on phenylketonuria (PKU) to the local chapter of the Association for Retarded Children [1]. Implementation of NBS as a public health prevention program began in earnest in 1962 when the federal Children’s Bureau funded a national trial aimed at screening 400,000 newborns in 29 USA states [1,2,3]. In the mid-1960s, as word of the Guthrie test [4,5] spread, including in the popular press [6], screening began expanding globally [7,8,9,10,11,12,13]. During the more than 60 years since, NBS has continued to grow, evolving differently in different political, economic, cultural, and geographic environments. 

In most high-income countries (HICs), NBS generally exists as an efficient disease prevention system administered by a public health program with the aim of reducing newborn morbidity and mortality for the benefit of the family and society. Its success in this regard is well documented. The USA Centers for Disease Control and Prevention (CDC) recognized NBS as contributing to one of the “Ten Great Public Health Achievements” during the first 10 years of the century noted that, “*Improvements in technology and endorsement of a uniform newborn-screening panel of diseases have led to earlier life-saving treatment and intervention for at least 3400 additional newborns each year with selected genetic and endocrine disorders*” [14]. Unfortunately, this accomplishment has not been fully realized in all countries, particularly in low-income countries (LICs) where NBS does not yet exist, in lower middle-income countries (LMICs) where NBS is relatively new, and upper middle-income countries (UMICs) where NBS is not yet well organized.

In 2015, the United Nations (UN) established 17 interlinked sustainable development goals (SDGs) as part of a blueprint for world peace and prosperity both now and in the future [15]. NBS directly impacts Goal 3.2, which seeks to end preventable deaths of newborns and children under 5 years of age by 2030. As we noted in our 2015 NBS status report [16], NBS becomes increasingly significant in national health considerations (both budgetarily and actionably) as the infant mortality rate (IMR) approaches the teens and single digits in LMIC countries. This is consistent with attacking SDG target 3.2.2, which seeks to reduce the IMR to at least 12 per 1000 live births, and SDG 3.2.1, which seeks to reduce the under-5 mortality (U5M) to at least 25 per 1000 live births [15]. 

Organizations within the UN family have provided varying degrees of support for NBS through the years beginning in 1968, when the World Health Organization (WHO) commissioned Wilson and Jungner to review screening practices. This activity resulted in publication of their 10 principles of population screening and recognition of the importance of mass public health screening [17]. Notably, their 10 principles included several examples of NBS for PKU and resulted in these principles forming the basis of NBS policies that continue in use today. In the 1990s, the WHO also published guidelines on the prevention and control of both PKU and congenital hypothyroidism (CH), which contained general principles for initiating sustainable NBS programs in developing countries [18,19]. Also in the 1990s, the International Atomic Energy Agency, Vienna, Austria (IAEA), the UN family’s global focal point for nuclear cooperation, recognized the value of NBS for CH using radioisotopes as part of the testing process, and provided funding for numerous NBS technical cooperation projects in LMIC settings [20]. As part of technical cooperation, the IAEA also published a book designed to assist LMIC countries in initiating NBS for CH [21].

More recently, the WHO, which has had a long-standing interest in sickle cell disease (SCD) and thalassemia, has recognized the importance of NBS as a useful public health intervention in reducing SCD-related morbidity and mortality. The 2006 Report by the Secretariat on sickle cell anemia noted that, “*There is evidence that the neonatal screening for sickle-cell anemia, when linked to timely diagnostic testing, parental education and comprehensive care, markedly reduces morbidity and mortality from the disease in infancy and early childhood*” [22]. Beginning in 2010, the WHO African Regional Office included NBS as part of its recommended early identification strategies for SCD [23]. In 2020, recognizing the need for guidance on screening programs in general, the European WHO office published screening program guidance, using NBS as examples in several of its chapters [24]. In 2023, the WHO Director of the Department of Maternal, Child, and Adolescent Health and Ageing presented a keynote address in which he noted that “WHO encourages member states, especially in LMIC settings, to consider universal NBS programs to screen for a few context relevant birth defects.” Further, the WHO intends to develop guidelines that “focus on 5–6 priority conditions” including, for example, “CH and hemoglobinopathies (HGB)” [25]. The importance of WHO’s endorsement of NBS as a useful, cost-saving public health initiative cannot be overstated since WHO guidance often plays a major role in setting health priorities globally. 

NBS continues to be institutionalized in countries with developing economies and expanded in those jurisdictions where it has been successfully initiated and sustained. In 2015, building on a series of regional NBS reports published in 2007 [26,27,28,29,30,31], we consolidated and reported on worldwide NBS activities [19]. In this report, we celebrate 60+ years of NBS by reviewing recent NBS activities within and between countries and global regions. Expanding on our previous report, we have divided the world into six regions for easier reporting: North America, Asia–Pacific, Europe, Latin America (LATAM) and the Caribbean, Middle East North Africa (MENA), and sub-Saharan Africa (SSA). This represents an expansion that includes both the Caribbean islands and SSA, and further illustrates the extent of NBS expansion globally. We have provided information on the NBS activities in each region to review continuing progress and to provide a comprehensive resource for comparative NBS program data. Where possible, these activities have been documented through reference to scientific publications, meeting presentations, or media reports. 

## 2. Methodology

The authors, residing in the various regions, have drawn on published reports and professional contacts to review, consolidate, and tabulate current NBS information within each region. We have carefully reviewed the published literature and other public sources, including meeting reports, public websites, and other data sites to update and expand the information in our 2015 report with particular emphasis on the last few years (2020–2023). In cases where available data did not appear to exist, to be reliable, or appeared to be in conflict with other data, we attempted to obtain new data or to resolve discrepancies through professional contacts. As a reality check, we have collaborated with commercial partners to compare data and fill in missing information whenever possible.

For each regional discussion we have first included a brief introduction to the region followed by a review of NBS activities within the region that either impact(ed) the region as a whole or were the product of collaborations between more than one jurisdiction within the region or globally. Regional discussions are followed first by a review of activities within individual jurisdictions within the region and then with a tabular summation of NBS activities across the region. The summary tables are similar in format from region to region to facilitate comparisons. Exceptions include the North American tables which are more complex and the sub-Saharan Africa (SSA) table which contains additional descriptive information due to a lack of NBS activities. Data tables have been designed to harmonize regional and national information where possible, so that the reader can make easier global comparisons. Each regional table contains similar information on the conditions screened, numbers of screening laboratories, and the time at which specimen collection is recommended. In the more active regions, we have focused on activities since 2020. Where there was little or no recent public information available on NBS, our review period has been extended slightly to provide the reader with information to assist in understanding the current state of NBS in that location, e.g., in some parts of LATAM and the Caribbean, MENA, and SSA. In order to provide similar data comparisons globally, we have used the same data source, United Nations International Children’s Emergency Fund (UNICEF), throughout: 2023 population and birth data, and 2022 IMR (<1 year) data. The exception is North America, for which equivalent local data were used. A listing of diseases mentioned, and the abbreviations used is provided at the end of the report.

## 3. Results

The number of references cited reflect the relative activities in the six regions. A total of 1590 citations have been included: North America, 400; Asia and the Pacific, 267; Europe, 435; LATAM and the Caribbean, 194; MENA, 146; and SSA, 148. While it is impossible to accurately cite every NBS activity in the past three years, the similar processes by which the data were obtained and the citations recorded provide a reasonable reflection of the comparative regional distribution of NBS activities globally. The following discussions are intended to provide the reader with a ready resource for basic NBS program information with documented resources from which to obtain additional details. 

### 3.1. North America

**Figure 1:**
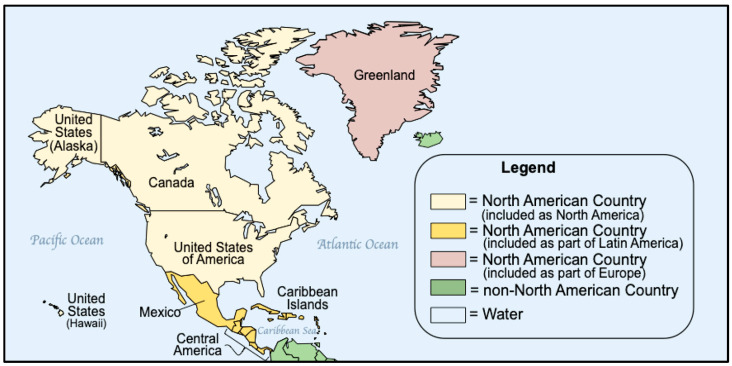
Map showing countries considered to be in North America. (Note: This portion of the report only addresses the United States and Canada).

North America (Figure 1) is the third largest continent and includes the USA, Canada, Greenland, Mexico, Central America, and dozens of possessions and territories in the Caribbean. For purposes of this report, North America will be limited to NBS in the USA and Canada. Mexico, the Caribbean, and countries in Central America are included in the discussions of Latin America, and Greenland, an autonomous dependency of Denmark, is included as part of European discussions.

The population of the USA is approximately 335 million distributed unevenly across the 51 jurisdictions [50 states and the National Capital Region—the District of Columbia (D.C.)]. The USA ranks third in population globally, accounting for approximately 4.25% of the world’s population. The most populous states are located near the borders on the West Coast, East Coast, and South. State populations range from almost 40 million in California to around 600 thousand in Wyoming. Aside from state jurisdictions, there are five permanently inhabited USA territories: Puerto Rico and the USA Virgin Islands in the Caribbean Sea, Guam, and the Commonwealth of the Northern Mariana Islands (CNMI) in the North Pacific Ocean, and American Samoa in the South Pacific Ocean. In the absence of a national NBS law, each state or territory has its own NBS law and administrative structure, often functioning like the national programs in other countries. With NBS implementation currently being planned for American Samoa, all states and territories will have a screening program. A detailed description of the history and functioning of the USA NBS system was reported previously [16,27]; however, a brief discussion below reviews the status of national NBS recommendations and related recent activities.

**Figure 2:**
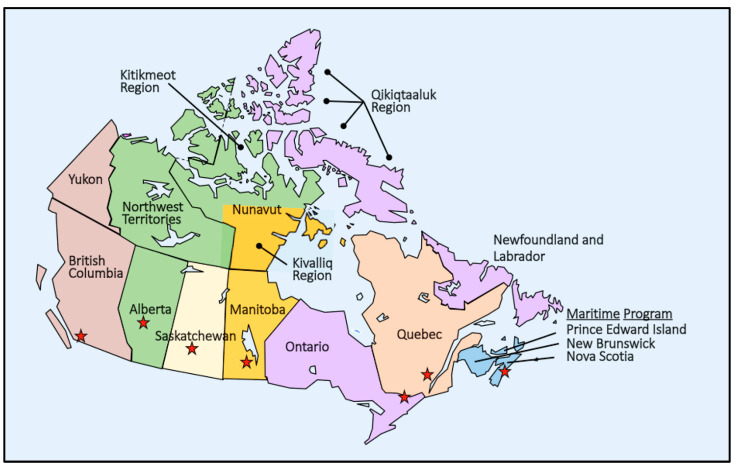
Map showing Canadian provinces and territories. Jurisdictions of the same color utilize the same screening laboratory (laboratory location indicated by a star).

The population of Canada is approximately 37 million with most of the population (~80%) distributed along the southern border with the USA leaving vast areas of the north that are only sparsely populated. Canada is the second largest country in the world by area covering approximately 3.5 million square miles, almost as large as the entire European Union. The birth population is approximately 360,000, slightly less than Texas, USA. Like the USA, there is not a national NBS law and each province functions independently. In the low birth populations in the north, NBS services may be provided by another province, usually based on the historic provision of health services (see Figure 2). While conditions included in NBS panels are expanding, there continue to be variations in the screening panels across provinces. A more detailed discussion of NBS in Canada has been previously reported [16,27,32].

#### 3.1.1. National NBS Activities Involving Multi-State and Multi-Provincial Collaborations

There are no USA or Canadian federal laws concerning NBS; however, in the USA, the Secretary of Health and Human Services (SHHS—equivalent to the Minister of Health in most other countries) is empowered to make recommendations to the states regarding the composition of their screening panel. The SHHS receives suggestions from an advisory committee, the Advisory Committee on Heritable Disorders in Newborns and Children (ACHDNC), which was established by the Children’s Health Act of 2000 [33]. In November of 2020, the ACHDNC was officially reorganized as a Discretionary Committee to the SHHS [34]. A history of the legislative and policy NBS advancements in USA NBS (with a tabular timeline) and possible future directions was published in 2022 [35]. 

Shortly after its first meeting in 2004, the ACHDNC reviewed the panel of NBS conditions recommended for national implementation by an expert group from the American College of Medical Genetics (ACMG—now the American College of Medical Genetics and Genomics) that was developed using an empirical scoring algorithm based on scientific evidence, expert opinion, and parent input [36]. Review of the ACMG report was finalized in 2005 and the panel of conditions included was recommended to the SHHS. Official SHHS approval was obtained in 2008, with a recommendation that this basic screening panel be considered for implementation in each state. The “Recommended Uniform Screening Panel” (RUSP) contained 29 primary conditions, including hearing screening (Table 1), and 25 secondary conditions (Table 2). “Primary” conditions were those whose score indicated readiness for immediate implementation and “secondary” conditions (targets) were those lacking in some aspect of their scoring and needing further research even though they may be revealed through testing for a primary condition. In Canada, the Provincial–Territorial Health Ministers created an Intergovernmental Newborn Screening Working Group in 2015 as an opportunity for program officials and jurisdictional clinicians to share information and improve NBS practices in Canada. This group requested feedback from NBS stakeholders including patient groups and advocates as part of the preparation of “*…a recommended Canadian NBS List of 22 diseases (see* Table 1*) [all were included on the original RUSP except SCID, which has now been added] to inform and provide guidance to provincial NBS programs*” [37]. While this listing has been helpful in informing Canadian NBS programs, the selection process in the USA and the RUSP continues to serve as a national model which can be modified to suit the local needs of Canadian and other jurisdictions. We will use a slightly modified version of this listing as a basis for harmonizing regional data in the tables published in this report and suggest its use as a base on which harmonize NBS globally. 

Although the ACMG expert panel developed a RUSP, it did not propose a mechanism for counting or naming conditions. Almost immediately, this was recognized as a problem (as one example, sickle cell disease was counted as a single condition in the Canadian list of 22, but as 3 separate conditions in the USA RUSP list of 29—see Table 1). Soon after adopting the RUSP, a small expert group assembled by, and including representatives from, the Health Resources and Administration (HRSA—original funders of the ACMG RUSP project) published recommendations to remove ambiguity and increase uniformity in naming and counting screening conditions [38]. To date, there remain issues in this regard and further efforts to improve naming and counting conditions are ongoing.

Building on the original RUSP, and utilizing a redefined evidence review process [39], the SHHS has now (December 2023) approved eight primary and one secondary condition for addition to the original RUSP, an additional five primary conditions since our 2015 report (see Table 3) [40]. During the last week of January 2024, Krabbe disease (KD) was approved by the ACHDNC and forwarded to the SHHS for final approval for addition to the RUSP. Reports in both the USA and Canada have recently commented on the selection process for NBS conditions: a chapter on NBS condition selection in Canada focused on risk governance concluding that transparency in Canadian screening panel selection is low, with the exception of Ontario, and resource limitations are at the heart of condition selections [41]; and a USA report briefly reviewed the history of NBS and discussed NBS mandates and the risks to their public acceptance/rejection posed by expanded screening and the potential for increased false-positive screens [42]. Recommendations for improvements in the USA system were included in a 2022 USA report [35] that suggested, “*…better defining the criteria by which screening targets are established; financing the NBS system’s responsiveness to opportunities for expansion, including engagement and funding from stakeholders; creating a national quality assurance, data, IT, and communications infrastructure; and improving intra-governmental communications*”. Interestingly, the 2023 Canadian report on NBS for SMA [32] noted that, “*Learning from the USA experience, Canadian provinces must work to standardize NBS panels across the country to reduce the existing inequities that can have life-altering consequences for children born in some but not other areas within our country*”.

Timely identification of newborns impacted by NBS is critical and the USA ACHDNC addressed this challenge in 2015 by recommending timeliness goals for state NBS programs (time-critical results reported by five days of life, and non-time-critical results reported by day seven). Federal financial support was provided for a multi-year quality improvement initiative to decrease the time for laboratory result reporting, the results of which were reported in 2020 [43]. An independent review of 10 state public health laboratories identified additional system improvements that might further improve NBS timeliness, noting that, “*Sustained improvements will require implementing robust data systems, integrating laboratory and follow-up processes, and improving communication among all NBS stakeholders*” [44]. Quality improvements in this regard are continuing. 

Expansion of NBS programs occurs slowly. A 2020 survey of state programs assessed the time and factors required for implementing NBS for Pompe disease (PD), mucopolysaccharidosis type-1 (MPS-I), adrenoleukodystrophy (ALD), and spinal muscular atrophy (SMA), finding that the usual time from start to finish was five to six years [45]. In 2022, a study by the USA’s federally sponsored Newborn Screening Translational Research Network (NBSTRN) [46] reviewed state expansion practices, identified expansion challenges, outlined areas for NBS improvement, and suggested how modeling could be used to evaluate changes and improvements [47]. A companion report identified at least one model to overcome each of the four challenges to RUSP implementation identified in the first report: (1) screening panel variability; (2) information limitations from short pilots; (3) expansion of NBS definitions by screening new conditions; and (4) capacity constraints in the RUSP review process [48]. In an effort to speed the expansion process, the NBSTRN developed web-based tools and resources that enabled NBS stakeholders to advance their research and potentially improve clinical care for patients and their families [49,50,51]. Reports in 2020 and 2023 provided aggregate data and reviewed the NBS experiences of the USA states and two territories for conditions on the RUSP from 2015–2017 [52] and 2018–2020 [53], and a separate 2023 report reviewed the rate of implementation of the added conditions from 2010–2018 [54].

##### Multi-Jurisdictional NBS Activities Related to RUSP Expansion (2016–2023) 

Activities aimed at assessing and improving NBS systems are ongoing, and their scope is often multi-jurisdictional. New conditions on the RUSP since 2006 and future directions for NBS have been a major focus of both Canadian and USA research activities. In this section, we review activities that had, or are having, an impact beyond a single program or that involved multi-jurisdictional collaborations. Reviews are limited to recent activities (2020–2023) and are intended only to give the reader a flavor of more recent NBS activities and as such, should not be taken as a comprehensive assessment or complete history of screening activities for a particular disorder or jurisdiction. Recent jurisdictional-specific NBS activities are reviewed in Section 3.1.2. 

To begin, SCID was added to the RUSP in 2010 (Table 2) and was a part of the 2016 Canadian recommended panel [37]. By 2019, all states, almost all provinces, and at least 20 countries had included some version of SCID and other T-cell lymphocyte deficiencies (a secondary RUSP target) in their NBS program (often supported by the Jeffrey Modell Foundation) [55]. A 2020 report reviewed the status of USA NBS for SCID, including methodologies and follow-up practices [56], and a 2021 review analyzed the way that SCID NBS has changed the disorder presentation and provided important lessons for public health programs, immunologists, and transplant specialists globally [57]. A review of the history of SCID NBS discussing SCID subtypes, T-cell receptor excision circle (TREC) assay limitations, and diagnostic/management considerations for infants with a positive NBS was published in 2021 [58], and in 2022, a report reviewed the success of molecular NBS for SCID and the ability of NBS to determine the SCID population prevalence [59]. In 2023, the Primary Immune Deficiency Treatment Consortium (PIDTC) reported on a 36-year longitudinal study measuring the effect of NBS on survival after hematopoietic cell transplantation for SCID [60]. 

While SCID is the primary NBS target, other conditions have been reported as part of the clinical follow-up after an abnormal TREC screening result for SCID: confirmation of the DNA repair defect in newborns with ataxia telangiectasia [61]; identifications of newborns having in utero exposure to immunosuppressive medications (a case series on natural history and management) [62]; diagnosis of cartilage-hair hypoplasia (an autosomal recessive, short-limb skeletal dysplasia with a variable immunologic phenotype) in an Amish cohort [63]; identification of Omenn using high-throughput DNA sequencing [64]; a case-based review of positive TREC results when the diagnosis was not SCID (e.g., prematurity, variants of T-cell lymphopenia (TCL) [65]; a case review of neonatal abstinence syndrome [66]; and a study characterizing the history and genetic findings of infants with non-SCID TCL [67]. 

Implementation of SCID NBS has resulted in the development of “SCID Compass”, an educational resource, funded by the USA government, for parents needing accurate information to assist in decisions about treatment and care for identified newborns [68]. A 2023 USA report reviewed the need for education for physicians, using case-based scenarios to review the principles of TREC-based NBS, the genetics and subtypes of SCID, and clinical management of a newborn with a positive SCID NBS result [69]. Further responding to physicians’ needs for better disease definitions when diagnosing SCID from NBS, the USA–Canada Primary Immune Deficiency Treatment Consortium (PIDTC) implemented and published updated definitions in 2022/2023 [70,71]. A recent report of pooled SCID NBS data from seven state NBS programs “*highlighted the trajectory of TREC values over time, both between and within newborns, and provides evidence for improved SCID screening recommendations in the premature and low birth weight population*” to reduce unnecessary follow-up [72].

Critical congenital heart disease (CCHD) was added to the RUSP in 2011; however, a detailed discussion is beyond the scope of this report. It suffices to say that all states and some provinces have included CCHD on their screening panel. The reader is referred to three recent reports assessing adherence to nationally endorsed screening protocols in state NBS programs [73], across USA military hospitals [74], and a joint 2017 position statement from the Canadian Cardiovascular Society/Canadian Pediatric Cardiology Association [75].

PD was added to the RUSP in 2015 and MPS-I in 2016. In 2020, three reports reviewed the development/status of NBS for PD and/or MPS-I: a report emphasizing the role of industry in leading and financing research for rare diseases in general, and PD in particular [76]; a report on the progress of NBS for PD and MPS-I in the USA and predictions for the future [77]; and a report on a federally funded USA project supporting the implementation of new RUSP conditions in state NBS programs with an initial focus on 15 states beginning to screen for PD [78]. Other 2020 reports focused on laboratory testing for lysosomal storage disorders (LSDs): a prospective comparative effectiveness study of three high-throughput screening assays for simultaneously screening four LSDs (Fabry disease, Gaucher disease, MPS-I, and PD) [79]; a report clarifying the limitations and rationale for optimizing a molecular genetic testing approach with targeted next generation sequencing (NGS) for PD [80]; and a report on the development and practical experience of using digital microfluidics to screen for MPS-I in the USA with mention of its possible use for MPS-II [81]. 

Continuing research into NBS for LSDs has identified some the complexities of population screening, including the identification of diseases that have later onset, are untreatable, or have uncertain significance, which inform decision-making considerations about NBS implementation [82], and the various economic considerations and health outcomes for infantile-onset PD [83]. A 2022 report discusses parental issues in dealing with the uncertainty of NBS in identifying late- or infantile-onset PD [84]. At least two reports in 2022 focused on improving NBS laboratory techniques: a report providing information on developments/applications of bivariate normal limits for the pre-symptomatic detection of MPS-I, PD, and KD [85]; and a report evaluating techniques that might improve the efficacy of MPS-II screening [86]. Reports in 2022 and 2023 focused specifically on the addition of MPS-II to the USA RUSP (RUSP 2022): a brief review of the impact of adding MPS II to the RUSP [87]; and a summary of the evidence supporting the addition of MPS-II to the RUSP [88]. A recent report reviewed a multi-state, proof-of-concept project aimed at harmonizing NBS results for both PD and MPS-I using regression and multiples of the median, with varied cutoffs and result values observed [89]. 

In addition to MPS-I, ALD was added to the RUSP in 2016. A 2020 review summarizes the understanding of experimental therapeutic treatment strategies at the time and provides an overview of critical historical developments, therapeutic trials, and the advent of NBS in the USA [90]. A 2022 report noted that the addition of ALD to the RUSP resulted in published recommendations on the surveillance and care of boys detected by NBS [91], but challenges remain, which will be addressed with a periodic review of which treatments work, and which do not [92]. At least three reports on NBS impacts on ALD were published in 2023: a review on the era of NBS with the aim of providing improving case conceptualization, informing prognostic counseling to optimize neuropsychological and mental healthcare for patients and their families [93]; a report on the impact of NBS on earlier detection and treatment given the large number (29 at the time) of states including ALD on their screening panels [94]; and a critical review on the impact of NBS on the diagnosis and treatment of male children with adrenal dysfunction reported during the first 10 years of NBS in the USA [95]. 

SMA was added to the RUSP in July 2018 on its second nomination. While several states and provinces have begun NBS for SMA, treatment costs and cost effectiveness remain an issue. Early treatment with nusinersen is thought to lead to better outcomes with pre-symptomatic patients detected through NBS. A 2020 report provides information on the cost effectiveness, with and without universal NBS, for infantile-onset SMA from a societal perspective [96]. A 2021 report describes the landscape of NBS for SMA in the USA including current SMA screening processes, challenges, and status [97] and the extent of SMA NBS across Canada was recently reviewed [32]. 

GAMT was added to the RUSP in January 2023, and a report detailing the findings of the evidence review team has been published [98]. A 2021 report of pilot testing in New York and Utah described a laboratory technique and algorithm feasible for NBS for GAMT and reported on the successful identification of two newborns from NBS, one in each state [99]. 

While other LSDs besides PD, MPS-I, and MPS-II are not yet on the RUSP, NBS for some has begun in some states (since state programs are independent in their condition selection) and KD has already been implemented in 10 state NBS programs at the time of this writing (see state reports Section 3.2.1). Although previously disapproved for addition to the RUSP on two occasions (Table 3), KD was approved for addition by the ACHDNC on its third nomination on 30 January 2024 and forwarded to the SHHS for consideration. Final action(s) rests with the SHHS and should occur later in 2024. A number of reports concerning KD have occurred in recent years. A 2022 report reviews considerations and concerns about NBS for KD based on state advisory committee deliberations [100] and a letter in response provides support for screening with answers to questions raised in advisory committee discussions [101]. A 2022 article on KD NBS [102] reviews progress made over the past five years in meeting RUSP requirements and notes that, “*…ethicists and newborn screening advisory committees continue to disregard the progress … made in the treatment and screening of KD.*”. A 2024 publication reported on a national-level analysis of race/ethnicity of patients with KD, comparing identification from NBS to patient trends in children’s hospitals observing “*that NBS detected increased rates of KD diagnosis in individuals of Asian ancestry, and a trend in black individuals*” [103]. [Note: NBS experiences in California, Georgia, Massachusetts, New York, Wisconsin are reviewed in Section 3.1.2.

##### Multi-Jurisdictional NBS Activities Related to the “Original” RUSP 

In addition to research with conditions recently added to the RUSP, several reports of activities related to conditions in the “original” RUSP (Table 1) have also been recently published. Most numerous have been activities associated with CF. An article in 2020 from Europe is referenced here because it reviews the history of CF NBS and lays the foundation for CF NBS activities in the USA and Canada [104]. A contemporary report by USA authors reviews progress made in making CF NBS universal (particularly where it is prevalent), reviewing the lessons learned over time, and providing an analytical framework for possible NBS in undecided locations [105]. A 2020 Canadian report analyzed CF and SCD carrier detection as a part of NBS and possible association with vulnerable child syndrome [106]. A 2021 report reviewing the outcomes of infants born during the first 9 years of NBS in the USA found that the median age at the first CF event decreased and there were positive health impacts from NBS, but early life nutritional deficits and high rates of infant hospitalizations persisted [107]. A 2021 Canadian report evaluated the health outcomes at school age for children who were classified as “cystic fibrosis screen–positive with inconclusive diagnosis” (CFSPID) [also known as “CF-related metabolic syndrome” (CRMS)] after NBS to ascertain the value of the sweat chloride test in predicting CF risk [108]. There were at least three reports dealing with NBS for CF in 2022: a discussion of the variations across USA screening programs in their cutoff levels for immunoreactive trypsinogen (IRT) and a survey of other factors affecting CF screening that might result in missed cases of CF [109]; a study to determine facilitators and barriers to timely diagnosis and treatment as a part of NBS for CF [110]; and a national study showing that children up to age 10 with CF, born after implementation of NBS, had no significant pulmonary improvement but significantly greater weight and height percentiles and a lower hazard of chronic infection with *Pseudomonas aeruginosa* [111]. Four publications focused on CF NBS in 2023: a report showing that detection rates of disease-causing CFTR variants (using CFTR variant panels) are lower in minority racial and ethnic groups, which may result in delayed diagnoses and health disparities [112]; a report on the variations in CF screening laboratory algorithms (F508del variant detection, CFTR gene sequencing, CFTR variant panels) and the possibilities for case detection inconsistencies and racial/ethnic inequities [113]; a study of whether inequitable identification of CFTR gene variants and/or bias may influence timeliness of evaluation after an out-of-range NBS [114]; and a 46-year retrospective review of NBS for CF looking at why CF NBS programs have failed to meet original expectations until now [115].

Recent articles have also appeared concerning other RUSP conditions. A multi-national study (including both USA and Canadian collaborators) of false-positive screening results for succinylacetone when screening for TYR-I found that maleylacetoacetate isomerase deficiency (clinically insignificant) was a recognizable cause, and that a second-tier screening test with (urine) maleic acid (a biomarker for) might be useful to improve screening efficacy [116]. A survey of the USA practices regarding screening for alpha-thalassemia was reported in 2020 [117] and a similar survey with beta-thalassemia was reported in 2021 [118]. Interestingly, there were reports regarding sickle carrier NBS in both Canada (2021) and the USA (2022), each with a different message. In the Canadian screening program in Ontario, carrier results are not disclosed and so an unbiased comparison of healthcare service utilization was possible between carriers and controls confirming that carrier status is likely benign in early childhood [119]. In the USA study, where carrier results are reported to the physician, many parents did not recall receiving the results, but more importantly, they wanted more information about the condition and its implications [120]. In a 2022 report on NBS for SCD, collaborators from Canada and Guyana investigated the feasibility of NBS for both SCD and CH in 2294 Guyanese mothers/infants, developing incidence data for both conditions and assessing the feasibility of NBS [121]. NBS for SCD and CH is also the subject of a recent 2024 publication that revisits the concept of NBS with early immunizations in locations with successful immunization programs. The authors note the need for point-of-care (POC) test development for CH and research and collaborations between HICs and LMICs so that POC tests for both SCD and CH could be combined with immunizations to increase NBS globally [122]. 

Several publications in 2020 dealt with NBS for CAH: a report describing the landscape of NBS for CAH in the USA [123]; a review of the variations in CAH screening algorithms across NBS programs [124]; and a discussion of the factors affecting NBS accuracy [125]. Several reports on economic issues related to screening processes also have recently been published (co-authors from both USA and Canada): a 2020 report reviews the challenges in assessing the costs and cost effectiveness of NBS for CAH (differences in methodologies, data sources for estimating health outcomes, and costs of early versus late diagnosis) [126]; a 2021 report on the benefits and costs of a successful public health NBS program for both CH and CAH [127]; and a “letter to the editor” in 2023 [128] challenging a report of “net cost savings” in NBS for CH and PKU in Sweden [129], noting that, “*Optimizing child development is sufficient to justify extending CH screening and treatment …There is no need to overestimate cost savings...*”. A report on the screening and management of CH in 2023 noted the need for “*… timely confirmation of the diagnosis, accurate interpretation of thyroid function testing, effective treatment, and consistent follow-up*” [130]. A debate on the value of including CH as part of NBS developed following a proof-of-concept report from French colleagues [131,132,133].

##### Multi-Jurisdictional Activities Related to Conditions Not Yet on the RUSP  

In addition to research related to conditions on the RUSP, there have been significant activities aimed at conditions that are not yet on the RUSP. In addition to interest in NBS for KD mentioned previously, there is continuing interest in MPSs, including screening specificity for MPS-I by incorporating second-tier glycosaminoglycan (GAG) testing into the screening algorithm [134]. Recent NBS assay developments for other MPSs have included a highly multiplexed (18-plex) dried bloodspot (DBS) biochemical assay for LSDs and other inborn errors of metabolism (IEM) [135], MS/MS assays for 10 MPSs [136], and NBS for the full set of MPSs based on a first-tier enzymatic assay and a second-tier analysis of glycosaminoglycans [137]. Emerging approaches for NBS of MPSs using digital microfluidics and fluorescence-based DBS assays have also been described [138]. There is continuing interest in NBS for Duchenne muscular dystrophy (DMD) with a number of recent reports: demonstration that hospital-based NBS using a two-tiered approach with an enzyme assay for creatine kinase (CK) coupled with targeted NGS for the DMD gene is feasible and can fill the gap until NBS is available [139]; development of an algorithm for screening and diagnosis of DMD-carrier females, including both NBS and cascade molecular testing of family members as part of the output from a pilot screening project in New York [140]; recommendations from a pilot for multi-tiered cutoffs as part of the CK enzyme assay based on age at time of specimen collection and birth weight or gestational age, with the possibility that other cutoff determinants may be appropriate (e.g., sex, race/ethnicity, and seasonal temperature) [141]; and multi-laboratory evaluation of prototype DBS quality control materials for CK-MM isoform assay for NBS [142]. Interest in NBS for metachromatic leukodystrophy (MLD) and development of an assay for arylsulfatase A (ARSA) activity in DBS leukocytes using liquid chromatography tandem mass spectrometry LC MS/MS [143] contributed to the development of a NBS testing algorithm [144]. A study on more than 27,000 newborn DBSs for MLD in Washington State successfully demonstrated the feasibility of NBS [145] and a larger comprehensive, multi-disorder NBS pilot in New York, with MLD as one of its disorders, is ongoing [146]. Other conditions for which studies have been reported from 2020–2023 include GM1-gangliosidosis [147], cerebrotendinous xanthomatosis (CTX) [148], *MT-ATP6*-related mitochondrial disease [149]; and Menkes disease [150]. NBS has also been suggested as a tool for epidemiologic studies of childhood leukemias [151] and a custom-made NBS test to detect Wilson’s disease has been recommended for use in Puerto Rico where the incidence is reported to be 1:14,000 [152]. Recent Canadian studies on other conditions have generally been within individual provinces and will be discussed later.

##### Other Multi-Jurisdictional NBS Activities 

The use of post-analytic, condition-specific laboratory tools such as CLIR (collaborative laboratory integrated reports, Mayo Clinic, Rochester, MN, USA) has found widespread use in NBS since 2020: a report on the combined impact of CLIR and second-tier testing on NBS for disorders of propionate, methionine, and cobalamin metabolism [153]; a report on using stored dried bloodspots (DBSs), CLIR, and longitudinal metabolomics to develop profiles informing NBS for succinic semialdehyde dehydrogenase deficiency [154]; a report of NBS improvements in detecting CH by integrating covariate-adjusted results of different tests into CLIR tools [155]; a report of a new DBS test method incorporating CLIR scoring to predict disease likelihood into biochemical analysis of plasmalogens from patients with peroxisomal disorders [156]; and a report of the use of CLIR to enhance augmented artificial intelligence and improve the accuracy of detecting rare aminoacidopathies in a developing NBS program (Pakistan) [157]. Other state- and country-specific uses are noted elsewhere in this report (e.g., California, Georgia, Kentucky, New York, The Netherlands, Norway, Sweden). The development of a database and web-based tools (dbRUSP) for analyzing 41 NBS metabolites and six variables has been reported, which contains separate modules to study the influence of single variables and joint effects of multiple variables on metabolite concentrations [158].

Various other studies relating to the use NBS laboratory data and/or DBSs have been reported: the use of archived DBSs to retrospectively provide valuable etiological information on the complex interplay between environmental exposures, biological response, and population phenotypes [159]; the underutilization of archived NBS specimens in health disparity research [160]; the influence of ethnicity on false-positive metabolic marker levels for four screened conditions [GA-I, methylmalonic acidemia, ornithine transcarbamylase deficiency (OTCD), and VLCAD] in a diverse newborn population [161]; and systematic examination of NBS data to determine whether timing of specimen collection (<24 h of age, 24–48 h of age, >48 h of age) could impact the performance of NBS for selected metabolic disorders on the RUSP [carnitine transport defect (CTD), IVA, MMA, and PKU] [162]. A number of recent reports have described continuing studies involving USA and Canadian partners with other collaborators, to predict gestational age using NBS DBS specimens to help inform medical care for newborns in low-resource settings: a 2021 report outlining a research project in which specimens from sub-Saharan Africa and Asia would be analyzed in Canada [163]; a report defining a project to validate a gestational age estimation algorithm using DBSs spot in low-resource settings in Zambia, Kenya, Bangladesh, and Zimbabwe [164]; a 2022 description of the feasibility specimen collection, shipping, and analysis for DBSs collected in Bangladesh, Kenya, and Zambia, and tested in Ontario, Canada for NBS, and possibly other activities [165]; and a 2023 report of three gestational age estimation models using ELASTIC NET multivariable linear regressions on cohorts in Canada and their application in Zambia and Bangladesh (data from heel sticks were superior to cord blood data) [166].

##### NBS Regional Activities Focused on the Future 

NBS continues to expand in response to increasing genomic knowledge and research, and opportunities for system-wide improvements persist. Several recent USA reports have emphasized NBS administrative/policy elements that may need adjustments to better embrace the evolving changes: a survey study identifying and evaluating expert recommendations for modernizing NBS in preparation for rapid increases in the number of early life therapies [167]; potential solutions to NBS challenges in the USA as a starting point for policy makers and other stakeholders seeking to maximize the impact of new transformative therapies for babies, families, and society [168]; opportunities to understand and potentially reduce rates of infant mortality with a need for equitable implementation and additional resource allocation [169]; public health focus group discussions on improving screening modalities, supporting diagnostic procedures, and screening for a wider spectrum of disorders while addressing costs, parent/provider education and potential negative impact of genomic information. [170]; arguments that genomic sequencing of newborns risks increasing health disparities [171]; and recommendations for policy changes, and their strategic and effective integration, needed to implement molecular testing in NBS and take advantage of emerging molecular therapies [35,172].

There are several recent reports concerning new genomic research and NBS: review of the involvement of the USA National Institutes of Health (NIH) in support of NBS-oriented research and its future in supporting genomic research [173]; lessons learned from early NBS studies examining the use of whole genome and exome sequencing as screening tools and the technical, ethical, and societal challenges to broad implementation [174]; the role of exome sequencing in NBS for IEM [175]; the need for innovative trial designs in genomic NBS research and the necessity for partnerships with health authorities, patients, researchers, and drug developers to match pace with the development of targeted therapeutics [176]; discussions from a June 2021, 3-day NIH-sponsored workshop entitled, “Gene-Targeted Therapies: Early Diagnosis and Equitable Delivery” [177]; discussion of the knowledge gaps in genetic NBS globally, the opportunities for innovative research, and the need for attention to equitable implementation of research findings [178]; a survey study of 238 rare disease experts showing broad endorsement of the availability of genomic sequencing for monogenic treatable conditions for all newborns [179]; and a review of the challenges and opportunities of genomic NBS for rare diseases and the need to generate evidence of benefit and answers to ethical, legal and psychosocial questions [180].

Genomic NBS has already begun and CFTR gene analysis is ongoing in some USA NBS laboratories. A proposal and two subsequent discussions have been published aimed at scalable prototypes for NBS (and diagnosis) of genetic diseases by rapid whole genome sequencing (WGS) along with virtual acute management guidance [181,182,183]. The Centers for Disease Control and Prevention (CDC) has evaluated and reported on nine commercial or laboratory-developed DBS extraction methods for DNA and library preparation methods for CFTR gene analysis, noting that, “*It is our expectation that for each new gene, panel of genes or even human exome, the DNA extraction and library preparation will need to be evaluated to ensure that it will give accurate and robust sequencing results*” [184]. The NIH-funded BabySeq project [185] (a newborn genomic sequencing project investigating medical, behavioral, and economic outcomes along with consent mechanisms, gene curation, variant interpretation, and disclosure methods) has begun producing reports including one in 2023 suggesting that large-scale comprehensive sequencing of newborns will reveal actionable unanticipated monogenic disease risks sometimes resulting in lifesaving, downstream medical care for newborns and their family members [186]. A systematic review of the principles of genomic NBS programs suggests that an atmosphere of transparency on the part of policy makers will be essential for program success [187]. A 2022 report suggests that consent may be an issue in USA NBS programs where there are currently state mandates, and that care must be taken in policy development going forward to avoid dismantling a critical public health system [188]. Interestingly, a separate 2022 report similarly notes the tremendous possibilities of genomic NBS and the policy issues that must be carefully addressed paying attention to the “*…ethical, communication, data management, legal, and social implications of genomic screening…*” [189]. A 2023 report notes that, “*…traditional NBS led to improvements in infant mortality and health equity only when it was implemented in association with measures to improve healthcare access for children*”. Correspondingly the authors suggest that genomic NBS, “*…will lead to better child health only when the same degree of attention devoted to genomic technologies will be directed to the promotion of public health measures that facilitate access to high-quality healthcare for all children*” [190].

#### 3.1.2. NBS Activities within Jurisdictions (State and Provinces)

##### State Activities (USA) 

The following listing of NBS activities in the various states was created from a review of online information, two requests for information from state NBS liaisons via a national listserv, and personal contacts. All NBS programs in the USA have comprehensive websites easily accessible by querying the Internet. The information listed here provides limited insight into the amount and type of research ongoing within the states and other documented newsworthy activities within the 2020–2023 time period. The state activity reviews are intentionally brief, and the reader is referred to the program’s website for additional detailed information about the state’s NBS history, functioning, and current requirements. Summary information on testing is included in Section 3.1.3.

- **Alabama**—The Alabama NBS program is one of about 12 states that require a second screen on all babies at 2–6 weeks of age. In March 2023, ALD and adenosine deaminase deficiency (ADA) were added to the screening panel [191].
- **Alaska**—NBS in Alaska includes laboratory testing by the Iowa NBS laboratory. NBS research sometimes focuses on the CPT-1A Arctic variant, a secondary condition on the RUSP (Table 3) that is highly prevalent among the Indigenous Arctic peoples of Alaska. A recent focus group study in two tribal settings showed opportunities for additional education for healthcare professionals and caregivers, improved educational outreach, and guidance for the care of infants and children who might have the condition [192].
- **Arizona**—A second NBS is required on all Arizona newborns, although some tests (CF, SCID, SMA, ALD, MPS-I, and PD) may not be run on the second specimen if the first screen is normal. Data from the first two years of NBS for SCID confirmed an incidence 2.5 times above the national average. This likely reflects Arizona’s unique population profile, which includes a higher percentage of Native Americans and an increased percentage of Hispanic/Latinos compared to the general US population [193].
- **Arkansas**—Consistent with the national push to make state screening mandates responsive to the RUSP and its changes, the Arkansas State Board of Health unanimously approved rules that add conditions to the screening panel as they are approved for the RUSP in July 2023, with the caveat that screening implementation must occur within 36 months following an RUSP addition [194].
- **California**—The California (CA) NBS program reaches the largest jurisdiction of newborns in the USA. As such, it can provide faster reviews of new conditions under consideration for implementation in state NBS programs. This can assist other programs in their decisions regarding screening methods and related screening issues. An extensive, carefully maintained, temperature-controlled biobank of NBS specimens exists, which is available for research under certain conditions. Several research initiatives have been recently reported that involved improvements in screening laboratory methodology: a comparative effectiveness study of three methods (MS/MS, digital microfluidics, and immunocapture) for detecting four LSDs (Fabry disease, Gaucher disease, PD, MPS-1), which demonstrated that all techniques had high sensitivity and that use of CLIR software tools markedly improved the performance of each [79]; the NBSeq project, using archived residual dried bloodspots (DBSs) and data from 4.5 million California newborns, showed that whole exome sequencing (WES) could be useful as a second-tier test for MS/MS, reducing false-positive results, facilitating timely case resolution, and occasionally suggesting a more appropriate or specific diagnosis [175]; a study showing that a Random Forest machine learning classifier could be trained with screening data to improve prediction of true and false-positive screens for 39 MS/MS metabolic analytes [195]; and a machine learning study that improved screening and demonstrated the utility of supervised machine learning in reducing false-positive screens for an expanded metabolite panel (with second-tier testing of screen-positive cases) of four disorders [GA-1, MMA, ornithine transcarbamylase deficiency (OTCD), and VLCAD] [196].
  Reports of the results of screening for various conditions have proven useful in decision making in other states. For example, a 2021 review of SCID NBS acknowledged the importance of CA NBS results (3.25 million newborns) in demonstrating the effectiveness of early identification of both SCID NBS and non-SCID T-cell lymphopenia [197]. NBS in CA for other conditions has also proven to be important: the results of screening 1.8 million newborns for ALD in CA during the first four years of NBS (16 February 2016–15 February 2020) [198]; the use of CLIR software tools in affecting the rapid resolution of post-NBS confirmatory testing for ALD [199]; first year results of NBS for PD using a two-tier screening process on 453,152 newborns validated the algorithm and showed that African American and Asian/Pacific Islanders had higher allele frequencies for both pathogenic and pseudodeficiency variants [200]; results of clinical and epidemiological outcomes for the first 18 months of NBS for SMA on 628,791 newborns (including assay specificity and sensitivity) [201]; and a report on the first three years of screening for MPS-I using a two-tiered approach (α-L-iduronidase (IDUA) enzyme activity followed by DNA sequencing for IDUA gene variants) on 1295,515 newborns [202].

Integration of a strong follow-up system into the CA NBS has resulted in better definitions of the conditions screened and other clinical possibilities during differential diagnosis of various conditions: elevated C26:0-lysophosphatidylcholine, the screen for ALD, warrants consideration of Aicardi–Goutières syndrome [203] and Lupus [204] as clinical possibilities; and a low citrulline NBS for CIT has been shown to lead to identification of MT-*ATP6* mitochondrial disease but further studies are needed to establish the prevalence and natural history this disease and to determine if it is an appropriate condition to nominate to the RUSP [205].

- **Colorado**—The Colorado NBS program requires a second specimen on all newborns between 8–14 days of age and provides laboratory testing for the neighboring state of Wyoming. Recently, the NBS laboratory relocated into a new laboratory space, which includes a larger area for MS/MS and a dedicated molecular suite allowing for easier integration of testing for new disorders.
  State NBS programs are increasingly responsive to the development of novel therapeutics and political advocacy, and there is increasing oversight and support for harmonization at the federal level. An increased number of conditions screened, increased false-positive screens, and national program improvement initiatives are creating new and increased scrutiny of state NBS systems, potentially posing an existential risk to the public’s acceptance of mandatory NBS. A recent report from Colorado reviews NBS history, current state, and program challenges with suggestions for overcoming some of the challenges [42]. Seeking to continuously evaluate and improve the Colorado NBS program, a review of late-diagnosed CF cases over a five-year period (358,000 newborns—IRT/IRT/DNA protocol) showed a need to change from a fixed IRT screening cutoff (50 ng/mL) to a floating cutoff (96th percentile or 50 ng/mL, whichever is lower) and the importance of continuous quality improvement in preventing late diagnoses [206].

- **Connecticut**—The Connecticut Newborn Screening Network, established at Connecticut Children’s Medical Center in collaboration with Yale New Haven Hospital in 2018, is a statewide network responding to all reports of out-of-range NBS results. The Network coordinates the diagnostic work-up with the newborn’s family and physician, arranging initial treatment and LTFU care with the appropriate specialty care team if a disorder is identified. The Epic electronic health record (EHR) system (Verona, WI, USA) has been used to establish registries for LTFU tracking, serving as a use case for applying and achieving the adoption of population health tools within the EHR system to track care delivery and quickly fill identified care gaps. Expansion of the LTFU registry to all NBS specialty care teams is underway [207].
- **Delaware**—Delaware was one of the first states to implement NBS in the early 1960s [3]. In 2018, the NBS program partnered with Nemours Hospital for Children to help manage the screening program. Screening laboratory services were discontinued at the Delaware Department of Health and Social Services and contracted to a private laboratory. In 2022, the Legislature formalized the Newborn Screening Advisory Committee to advise the Director of the Division of Public Health and discontinued the requirement for a second NBS specimen [208].
- **District of Columbia (DC)**—The DC NBS program uses a private laboratory for NBS and is one of two programs routinely offering NBS for G6PD (Pennsylvania is the other). A recent study of the experiences with MPS-I screening from December 2017–February 2019, reviews the “impact of overrepresentation of screen positives in a minority group and unintentional creation of health disparities and community wariness regarding medical genetics evaluations” and advocates for “MPS I secondary testing … in conjunction with DBS-IDUA activity levels on all infants as part of the initial newborn screen, regardless of race, ethnicity, or ability to pay.” [209].
- **Florida**—The Florida NBS program began screening for MPS-I and PD in February 2020, SMA in April 2020, MPS-II was approved for addition in 2022 and GAMT in 2023. Florida law requires the addition of conditions added to the RUSP within one year [210]. Genetic counseling via telemedicine is the subject of a 2023 report from one of the Florida follow-up centers. This center was able to demonstrate that genetic counseling for CF via telemedicine was feasible across a variety of distinct geographic locations with diverse socioeconomic status throughout the United States [211].
- **Georgia**—The Georgia NBS program is a collaboration between the Department of Health for testing and program administration and Emory University for follow-up and treatment/management. A study of 52 SCAD and 9 IBDD cases detected by NBS between 2007–2016 revealed no major health problems overall, no significant difference in cognitive development, and only a slightly higher incidence of reported neonatal hypoglycemia in the SCAD group. As a result, the anxiety reported by parents may be unwarranted and the study authors suggest that these conditions should be considered for removal from NBS panels [212]. As part of NBS follow-up, the Georgia program uses CLIR post-analytical tools to triage abnormal results by assigning each case a risk level, which is used to guide follow-up recommendations. Based on experience, the follow-up program has reportedly moved forward in a more prospective manner with CLIR tools to reduce the number of cases needing follow-up [213]. The efficacy of using CLIR tools were also evaluated for the detection proximal urea cycle disorders (PUCD) in the Georgia NBS program. A high number of false-positives regardless of cutoff adjustments led the authors to conclude that PUCD is not suitable for statewide NBS using their procedure and suggested that a method that separates glutamine from other amino acids may work better [214].
  Experiences with ALD, PD, MPS I, and SMA have also been reported. The ALD pilot used a two-tiered strategy to quantify very long-chain lysophosphatidylcholines (LPCs) using flow-injection MS/MS (FIA-MS/MS) as an initial screen and LC MS/MS as a second-tier screen [215]. Pilot screening for PD and MPS I involved a two-tier strategy of FIA-MSMS enzyme assays. While false-positive screening results were reduced for PD, the frequency of pseudodeficiency was problematic with MPS-I, suggesting that LC-MS/MS analysis of dermatan and heparan sulfate might be a better alternative [216]. For SMA, a 2022 report detailed findings from a year of pilot screening (February 2019–February 2020) and the first year of standard screening (February 2020–February 2021) using real-time PCR assays. In addition to assay performance data, the report included disease incidence, time to diagnosis and treatment, and early clinical outcomes [217].

- **Hawai’i**—Since October 2019, NBS specimens on Hawai’i newborns have been tested at the Washington State NBS laboratory, which provides testing services on weekends allowing for more timely testing. On 3 July 2023, both PD and MPS-I were added to the Hawai’i NBS panel and on 1 January 2024, SMA and ALD were added [218].
- **Idaho**—The Idaho NBS program requires a second screen at 10–15 days of age. Since April 2021, Idaho NBS specimens have been analyzed by the Washington State NBS laboratory. On 1 February 2022, four new conditions were added to the Idaho NBS screening panel—PD, MPS-I, ALD, and SMA [219]. In addition to the NBS success story shared in the Block Grant Executive Summary (formal request for federal funds), Idaho NBS Program staff joined with the Idaho Medicaid staff to successfully remove prior insurance authorization restrictions for diagnostic testing and treatment for NBS conditions that prevented access to early diagnosis and treatment [220]. NBS staff were also successful in meeting with and educating the Director of the Department of Health and Welfare about NBS and getting the Governor to recognize September as “Newborn Screening Awareness Month,” which recognizes the program’s importance and increases awareness among prospective parents [221].
- **Illinois**—The Illinois Legislature has taken an aggressive approach towards NBS in recent years with Illinois becoming one of the first states to require screening for LSDs. In 2023, Illinois became the first state to require NBS for metachromatic leukodystrophy (MLD) and the Illinois Department of Public Health NBS laboratory is in the early stages of preparation for implementation in 2024 [222]. As a result of the earlier screening in Illinois, publications of Illinois experiences have provided valuable information for other state programs: a report on screening experiences with 684,290 Illinois newborns for PD [223]; a report of the incorporation of psychosine measurements in KD screening to identify newborns with infantile KD and infants with probable late-onset KD [224]; a report on the successful implementation of ALD screening in Illinois reviewing the screening experiences with 276,000 newborns using LC MS/MS [225]; an update on measuring iduronate-2-sulfatase (I2S) activity to detect MPS-II in 339,269 newborns [226]; and a further update on in 586,323 newborns screened for MPS II with the suggestion that, based on Illinois data, MPS-II may be more common than previously recognized with a higher prevalence of attenuated cases [227].
- **Indiana**—In 2020, the Indiana Legislature passed, and the Governor signed, a law expanding NBS to include PD. This law went into effect on 1 July 2020 and included KD and MPS-I [228]. With an eye towards the future and genomic sequencing, the Rady Children’s Institute for Genomic Medicine is involved in an investigatory project called BeginNGS^®^, which is an international, pre-competitive, public-private consortium that proposes to implement a self-learning healthcare delivery system for screening all newborns for over four hundred genetic diseases, diagnostic confirmation, implementation of effective treatment, and acceleration of orphan drug development [229,230].
- **Iowa**—The Iowa NBS laboratory provides laboratory services for Alaska and both North and South Dakota. As with eight other states at the time, the Governor signed legislation in April 2022, that requires the Iowa NBS panel to harmonize with the USA RUSP as new conditions are added/subtracted. Implementation is required within two-and-a-half years of addition with an annual status report of which new conditions were added to the panel and if/why implementation might have been delayed [231]. SMA was added to the screening panel on 13 September 2021 [232], and PD and MPS-I were added on 15 May 2023 [233]. In a letter to the public, the first fee increase for NBS since 2014 was introduced (USD 122 to USD 162) covering increased testing and courier services, among others [234]. A recent review of HGB screening data in Iowa documented the birth prevalence of hemoglobinopathies in Iowa and highlighted the need for periodic outcome evaluation to ensure that the healthcare needs of underserved, minority populations are met [235]. A summary of SCD data for each state from 1 January 1991 through 31 December 2010 has also been published [236].
- **Kansas**—The Kansas NBS program added screening for PD and MPS-I on 5 January 2021 [237]. A brief but comprehensive study guide on NBS was published by Kansas researchers in 2020 [238].
- **Kentucky**—A report in 2021 described two late-diagnosed cases of HCY following normal NBS results despite the use of CLIR post-analytical tools as part of the screening algorithm. The performance of methionine and homocysteine analyses as part of the screening process for HCY suggests that a change to NGS might be useful [239].
- **Louisiana**—In January 2022, the Louisiana Department of Health added NBS for SMA, MPS-I, and PD. Most NBS-related research focuses on clinical applications, which is beyond the scope of this report [240].
- **Maine**—The Maine NBS program added PD, ALD, MPS-I, and SMA to the screening panel, effective 1 April 2021 [241]. Laboratory testing services are provided by the Massachusetts NBS laboratory as part of the New England NBS collaboration. A 2022 law requires development of a CMV screening program though rulemaking that includes a gradual expansion from a targeted program to universal testing using either urine or saliva and currently does not affect the bloodspot NBS program [242].
- **Maryland**—The Maryland NBS program strongly recommends a second specimen between 7–14 days of age with a high success rate, and thus it is considered a two-screen state. Consent for NBS, which was required prior to 2008 is no longer required [243]. Maryland became the third state to pass RUSP alignment legislation in 2023 (10th overall) requiring harmonization with the national RUSP. The law requires implementation of a new condition within two years of its addition to the RUSP [244].
- **Massachusetts**—The New England Newborn Screening Program (NENSP) provides NBS laboratory services for five New England states (Maine, Massachusetts, New Hampshire, Rhode Island, and Vermont) and is part of the University of Massachusetts Chan Medical School. There are two types of NBS in Massachusetts, “required” and “voluntary” (pilot). “Required” NBS is conducted for conditions with known effective treatments and all newborns must be screened for these conditions unless there is a religious objection. “Voluntary” NBS allows for the study of new NBS conditions and is performed at no additional charge [245]. This NBS system has allowed the Massachusetts NBS program to be one of the USA leaders in contributions to NBS research. As an example, SMA analysis originally included a tiered algorithm looking for the absence of SMN1 Exon 7. When results from the first and second tier needed reconciliation, a third tier DNA sequencing assay was developed that confirms SMN2 copy numbers without the need for additional instrumentation [246]. A program report on screening 179,467 newborns confirmed the value of the screening algorithm in detecting SMA-affected infants who show absent *SMN1* Exon 7 by Real-Time™ quantitative PCR (qPCR) [247].
  Results from 10 years of NBS for SCID in Massachusetts support using a single NBS testing-and-referral algorithm for all gestational ages, despite lower median TREC values in premature infants, and that low naïve T-cell percentages are associated with a higher risk of SCID/CID and support the utility of memory/naïve T-cell phenotyping as part of follow-up flow cytometry [248]. As part of NBS research activities during the COVID-19 pandemic, deidentified NBS specimens were used as a source of maternal antibodies to estimate the cumulative incidence of severe acute respiratory syndrome coronavirus 2 (SARS-CoV-2) to assist in setting public health policies [249]. Additionally, editorials addressing research questions pertinent to NBS have been introduced by Massachusetts researchers: (1) whether NBS for vitamin B_12_ deficiency be incorporated into all NBS programs via a second-tier homocysteine assay that would increase the vitamin B_12_ deficiency cases identified and decrease the number of missed (false negative) cases of HCY [250]; and (2) whether more effective NBS for HCY is on the horizon using MS/MS with selective thiol derivatization [251].

- **Michigan**—The NBS laboratory in Lansing stores a residual NBS specimen on each newborn screened, while five more are sent to the Michigan Neonatal Biobank in Detroit for storage under climate-controlled conditions and possible research use. Since 2010, consent has been required for specimen storage and possible research use. In 2022, the Michigan NBS program agreed to destroy 3.4 million dried bloodspots as part of an ongoing lawsuit on privacy and consent [252]. Currently, legal issues continue with both sides obtaining wins and losses in the courts and continuing appeals.
  Michigan researchers have recently published reviews discussing both NBS for SCID [58] and NBS generally [253]. A cost-effectiveness and outcomes study for PKU was reported in 2021 based on new treatments and other new data [254]. At least four reports in 2022 addressed various issues pertinent to NBS: a report providing initial evidence of the value of screening for adenosine deaminase deficiency (ADA) using MS/MS [255]; a report showing the benign nature of transcobalamin receptor deficiency and the assertion that its detection should be considered an incidental finding with the caveat that long-term data are needed to ascertain the long-term outcomes of identified children [256]; a report on online NBS education for expectant parents showing that virtual baby fairs are cost effective, convenient, and equitable [257]; and an evaluation of national data currently maintained in a federally-supported database (NewSTEPs) that showed, “*… potential usefulness of NewSTEPs for research if investments in higher-quality data are made*” [258]. Physician education was the subject of a 2023 report that used case-based scenarios to review SCID NBS results, the principles of TREC-based NBS, the genetics and subtypes of SCID, and patient management for a positive TREC-based screening result [69].

- **Minnesota**—Press releases in February and August 2023 announced the addition of cCMV and a group of three other conditions (GAMT, MPS-II, and KD) to the Minnesota NBS panel. While the latter group is currently in the implementation phase, the Minnesota NBS program became the first in the nation to implement universal bloodspot screening for cCMV on 6 February 2023 [259]. This builds on earlier target NBS for cCMV in Utah, universal NBS in Ontario, Canada, and a Minnesota study that demonstrated sufficiently high analytical sensitivity and low cost for PCR for DNA from DBSs [260]. A study of urine collected on filter paper showed high sensitivity and specificity, but the collection method requires further study [261]. A 2023 report describing different newborn cCMV NBS approaches makes the case that implementing universal cCMV NBS rather than a targeted screening approach improves cCMV detection, ultimately leading to better health outcomes [262]. A recent report from the Minnesota program considering droplet digital PCR (ddPCR) as a primary DBS screening tool found that ddPCR does not demonstrate any enhancement in sensitivity compared to the use of real-time PCR testing of DNA from DBSs [263].
- **Mississippi**—The Mississippi Legislature became the first to adopt RUSP alignment legislation in 2022 and the eighth state to implement such legislation overall. In addition to requiring harmonization with the RUSP as new conditions are added/deleted, the legislation requires implementation within 3 years, and a status report every 12 months if the addition of a condition is delayed [264].
- **Missouri**—The Missouri NBS program was the first USA NBS program to require screening for SMA in 2017 and has been one of the most active programs considering, evaluating, and using the microfluidics assays for various LSDs and one of the earlier state programs implementing LSD NBS (in particular, screening for PD, MPS-I, Fabry disease, Gaucher disease, and KD). As such, its experiences have been helpful for other state programs beginning to implement LSD NBS. In 2020, a report on lessons learned from 6 years of PD NBS noted that the rates of later-onset PD phenotypes and pseudodeficiency alleles were higher than initially anticipated and should be considered during PD NBS implementation [265]. Another 2020 report detailed the analytical and clinical validations of a micro-well fluorometric assay measuring of iduronate-2-sulfatase (IDS) for MPS-II, which was implemented for full population screening after its validation [266]. A report from a Missouri tertiary care center in 2021 described the immunological findings and clinical outcomes of newborns who screened positive for SCID [267].
- **Montana**—In 2021, the Montana Legislature passed legislation requiring the Department of Public Health and Human Services to set up a Newborn Screening Advisory Committee to impartially hear testimony and approve or deny, by a majority vote, conditions nominated for inclusion on the NBS panel. The Committee was formalized in 2022 and began discussions of adding ALD. At its October 2023 meeting, the addition of ALD was approved [268].
- **Nebraska**—NBS in Nebraska is compulsory and no exemptions are allowed, including for religious reasons. NBS laboratory testing is through a contract with Revvity (Pittsburgh, PA, USA). A 2023 report outlines the experiences and challenges of implementing NBS for ALD in Nebraska including a lack of genotype-phenotype correlations, absence of predictive biomarkers for childhood cerebral ALD or adrenomyeloneuropathy, and a high proportion of *ABCD1* variants of uncertain significance combining to cause unique counseling difficulties [269].
- **Nevada**—The Nevada NBS program requires a second NBS specimen on all newborns collected at 10–14 days of age. Screening for SMA began 21 December 2023 [270].
- **New Hampshire**—New Hampshire obtains NBS laboratory services from the NENSP in Massachusetts. Three conditions were added to the New Hampshire NBS panel (MPS-I, ALD, PD) with screening starting on 26 August 2020. The screening fee increased from USD 104 to USD 146 [271]. The NENSP expects to begin screening for MPS-II in August 2024 and GAMT around January 2025, which will impact decisions by the New Hampshire NBS Advisory Committee [272].
- **New Jersey**—The New Jersey NBS program has found itself involved in lawsuits concerning the use of residual dried bloodspots. The first involved the release of information about police use of bloodspots [273,274] and the second (filed 2 November 2023) concerns the storage of residual bloodspots [275]. Both cases involve complexities outside of the scope of this report. Also in 2023, a report on a series of five cases diagnosed late with negative NBS results led to quality improvement in the New Jersey CF screening laboratory algorithm [276]. A new law requires NBS for cCMV contingent on the development of a suitable bloodspot cCMV test, addition to the RUSP, and acquisition of the necessary equipment to conduct the test, among others [277].
- **New Mexico**—The New Mexico NBS program requires a second screening specimen for all newborns 10–14 days after birth. Specimen testing is performed at the Oregon NBS laboratory, which began screening for Fabry disease and Gaucher disease in New Mexico on 1 July 2022 [278]. A recent meeting of the Northwest Regional Newborn Bloodspot Screening Program Advisory Board, which advises the Oregon laboratory, considered whether to remove Fabry and Gaucher diseases from the screening panel and a detailed report of current evidence was prepared for its consideration. The decision was not to remove either condition and the Oregon NBS laboratory reported that it would be able to continue testing for New Mexico even if discontinued in Oregon [279].
- **New York**—New York has the third largest newborn population in the country and significant research efforts exist, often in collaboration with other programs as noted in Section 3.1.1. Currently, screening activities are focused on screening for cCMV which began as a 1-year pilot on 2 October 2023 [280] and Duchenne muscular dystrophy (DMD), which was officially added to the screening panel on 25 October 2023, for implementation in early 2024 [281]. In the latter case, implementation builds on a prospective consented pilot begun in 2019 that was aimed at addition of DMD to the RUSP and was developed in collaboration with expert partners interested in muscular dystrophies and NBS (Duchenne NBS Consortium). The 2-year pilot allowed for validation of the testing protocol and follow-up procedures and demonstrated the feasibility of NBS for DMD through successful case detections [282]. The results demonstrated the value of NBS for DMD [283]. During the validation, several factors affecting variability of creatine kinase-MM (CK-MM) levels were studied. The most prominent was an inverse relationship between the CK-MM concentration and age of collection. This information was used to implement age-related cutoffs for CK-MM [284]. Addressing the need for external quality control materials, in collaboration with the CDC and RTI International, prototype dried bloodspot quality control materials for CK-MM NBS assays were also evaluated [142].
  A number of other NBS activities have been ongoing since 2020, including experiences during the COVID-19 pandemic, experiences that led to development of new recruitment strategies for pilot studies, which may allow other research studies to adapt novel and more effective recruitment methods [285]. At least four NBS reports were published by New York researchers in 2020: a review of the impact of using CLIR post-analytical tools on NBS for KD (referrals reduced by ~80%) and PD (referrals reduced by ~32%) [286]; a report on the first year of SMA screening in New York (225,093 newborns screened) produced an incidence of SMA 2.6- to 4.7-fold lower than expected [287]; a review describing the current knowledge of the pathophysiology of ALD and updates on NBS, diagnosis, monitoring, and treatment [288]; and a retrospective analysis of data from a SCID Referral Center demonstrating that TREC value can be used to stratify infants for further confirmatory testing [289]. Reports of at least three NBS program activities were reported in 2021: results of a 10-year study on CH explored the effect of manufacturer’s lot changes on the daily mean values of thyroxine (T4) and the influences of seasonal variations on values for both T4 and thyrotropin (TSH), examining the effect of both on screening positive predictive values (PPVs) [290]; details of a modification to the CF NBS algorithm using a new custom NGS platform for more comprehensive CFTR gene analysis improving the PPV of CF NBS from 4% to 25% [291]; and a report on the first two cases of GAMT identified by NBS in New York and Utah [103]. A 2022 report describes a modification to the screening algorithm for GAMT eliminating the need for second-tier screening and reducing referrals for follow-up by 85% [292]. A review of the clinical outcomes from the first three years of SMA NBS was also reported in 2022 [293] along with and a description of potential improvements in the pre-symptomatic detection of MPS-I, PD, and KD using tools based on bivariate normal limits [85]. A 2023 report outlines the basic steps in organizing universal CF NBS with the evolution of CF NBS in New York as a guide, and discusses how to reduce bias, highlights challenges, offers guidance and recommendations for future consideration [294].
  The New York NBS program is currently actively participating in two consented pilot studies, one exploring the use of first-tier genome sequencing for highly penetrant early onset conditions. This study, Genomic Uniform-screening Against Rare Diseases in All Newborns (GUARDIAN), is screening 100,000 newborns for 250 medical conditions not currently screened by New York state [295]. The second, ScreenPlus, is a consented pilot study screening for 14 diseases (mostly lysosomal disorders), which includes long-term follow-up studies and will evaluate the ethical, legal, and social challenges associated with screening for diseases that include late onset phenotypes [146].

- **North Carolina**—In 2021, North Carolina became the sixth state nationally to enact RUSP alignment legislation requiring implementation of NBS for a condition within three years of its addition to the RUSP and a report on the status of the addition at 18 months and every 6 months the addition is delayed past 3 years [296]. In February 2023, MPS-I and PD were added to the NBS panel of tests. SMA and ALD were added to the screening panel in 2021 and 2022, respectively [297]. Screening for each built on pilot studies, the results of which have been published: a report on the ALD pilot with particular focus on the single-tier HPLC-MS/MS screening test and its PPV and recall rate [298]; and a report on the SMA pilot that included evaluation of the SMA q-PCR screening method to confirm its robustness [299]. These studies were aided by the existence of the Early Check program, which is a comprehensive opt-in program that supports pilot studies of new NBS conditions. Its overarching objective is to demonstrate the feasibility and acceptability of statewide screening and follow-up for inborn conditions, inform public policy, and support USA RUSP nominations [300]. Other Early Check-related projects have included the use of a patient portal to recruit research participants for Early Check [301], direct mail and email outreach/recruitment of new mothers for NBS research [302], and education and consent in large-scale DNA screening [303]. With the research foundation in place, future studies are beginning including genome sequencing [304]. Other recent NBS activities within the state have included research and development in microfluidic instrumentation directed at mucopolysaccharidoses [81], validation of NBS for Fragile X using a custom FMR1 PCR assay system [305], and evaluation of a commercial assay for DMD NBS (CK-MM) and the DBS stability of CK-MM [306]. A 2024 report chronicles two years of newborn screening for Duchenne muscular dystrophy as a part Early Check [307].
- **North Dakota**—NBS specimens from North Dakota are analyzed and reported by the Iowa NBS program. A report in 2019 provided preliminary evidence of association between NBS refusal and provider type, home births, and hepatitis-B vaccine refusal while noting that, “*Additional studies of obstetric providers, home births and women are needed to improve our understanding of the reasons for NBS refusal to better deliver preventive services to newborns*” [308].
- **Ohio**—In July 2021, the Governor signed RUSP alignment legislation requiring implementation of screening for conditions that are added to the USA RUSP [309]. In July 2023, there was a NBS fee increase, which covered, among other things, testing for SMA and ALD, which were added to the screening panel in October 2022 [310]. Also in July 2023, the Governor signed legislation that requires the Ohio Department of Health Director to specify in rule the addition of DMD as a disorder for NBS making Ohio the first state to have a mandate for DMD NBS [311]. The Ohio NBS program includes KD and a report in 2022 discussed a qualitative assessment of parental experiences with false-positive NBS for KD, which showed concern regarding the lack of knowledge and short-term counseling skills among non-genetics providers and a need to integrate genetic counselors within the NBS result disclosure process [312]. Another 2022 report reviewed the value in additional screening for very low-birthweight newborns with “normal” screening results until thyroid function has been ascertained [313]. A 2024 publication reviewed parental experiences with NBS and gene replacement therapy for SMA [314].
- **Oklahoma**—The Oklahoma public health laboratory, including NBS, moved from Oklahoma City to Stillwater in 2020. Planning was limited and NBS testing was outsourced to a private laboratory for a brief period. This change added four conditions (PD, ALD, SMA, MPS-I) to the NBS armamentarium along with the addition of screening for SUAC, as a second-tier test for TYR-I. The screening laboratory is now operational in the new facility with these additions, although some challenges remain [315,316,317]. In May 2022, legislation requiring alignment with the USA RUSP was signed into law, which goes into effect in November 2024 [318].
- **Oregon**—The Oregon NBS program (also known as the Northwest Regional Newborn Bloodspot Screening Program) has been updating its screening algorithms for several screened conditions, including cystic fibrosis. Screening has expanded from 23 conditions to 62, most notably adding SMA in June 2022, and ALD in January 2023. The screening fee has also increased from USD 80 to USD 175 for two screens (August 2022), one near birth and the other at 1–2 weeks of age [319]. The 2022 Advisory Board meeting considered whether to remove Fabry and Gaucher diseases from the NBS panel and decided they did not meet their criteria for removal [279]. Other studies have included a framework to balance sensitivity with the unnecessary treatment of healthy infants due to *A143T* and other variants of uncertain clinical significance [320], and a review CAH screening accuracy and biochemical and clinical outcomes of CAH cases detected by the Oregon program, which collects specimens at two time periods following birth [321].
- **Pennsylvania**—The Pennsylvania NBS program is unique in its administration and operation and is governed by the Newborn Child Testing Act, which was amended in 2020. As part of the amendments, the NBS panel and program financing were modified/clarified. The NBS panel was expanded to 34 named conditions, including the addition of KD. Birthing facilities must continue to “*… utilize and enter into agreement with the NBS laboratory contracted with the Department [of Health]*”. This legislation also required compliance with the USA RUSP. Other program changes have included CF screening algorithm adjustment to include NGS (1 October 2022), addition of MPS II to the screening panel (1 July 2023), and implementation of GAMT screening (1 January 2024) [322]. Other NBS studies ongoing in Pennsylvania include a 2020 report on experiences with PD screening [323] and a 2022 report on data and outcomes for ALD NBS [324].
- **Rhode Island**—Rhode Island has a small birth population and partners with the Massachusetts NBS laboratory (NENSP) for laboratory services. In 2020, the program expanded by adding screening for SMA [325].
- **South Carolina**—The South Carolina NBS laboratory implemented Sunday courier service in September 2020, and weekday courier in September 2021. Screening for MPS-I and PD began in February 2021. Screening for SMA, argininemia, and ALD began in 2022 and KD in May 2023. In April 2022, the NBS program began a partnership with the Bureau of Vital Statistics to match newborn vital records with newborn blood-spot screening records to ensure that all newborns receive NBS [326]. Given that the pediatric provider of record has a significant role in NBS and infant health after birth, a recent South Carolina study examined how the pediatric provider of record is selected by parents and factors that might affect NBS education and processes in the perinatal period [327].
- **South Dakota**—The South Dakota NBS program utilizes the services of the Iowa NBS laboratory. In September 2022, PD was added to the NBS panel, and the fee was increased from USD 91 to USD 98. The South Dakota NBS program began providing long-term follow-up for diagnosed disorders through the Sanford Children’s Specialty Clinic in Sioux Falls in August 2023. The Sanford long-term follow-up team will provide care coordination, insight, primary care and specialist communication, support, and referral services to various state and private programs to NBS patients and parents [328]. A 2022 report summarizes NBS in South Dakota, including changes over time, the groups of conditions included in screening, the process of adding conditions to the screening panel, and the role of the primary care physician when a positive screening finding is reported [329].
- **Tennessee**—An open-access dashboard was developed to address NBS needs for methods to better visualize performance data, promote data transparency, and drive quality improvement. Eight NBS performance indicators can be visualized across several views, which are designed to provide an overview of NBS performance data at first glance, then allowing a drill-down to specific data. Dashboard development experiences can be applied to future dashboard development in or other public health programs implementing similar measures [330]. Personnel from the Tennessee NBS program have contributed as co-authors to publications in collaboration with other state NBS programs (as examples, see discussions on PD, MPS-I, α-, and ß- thalassemias, CF in Section 3.1.1).
- **Texas**—The Texas NBS program is one of the largest in the world processing some 800,000 specimens annually for over 30 different conditions (each newborn is required to be tested near birth and at 1–2 weeks of age.) NBS for SMA was begun in June 2021, and in June 2023, Texas became the first state to pass RUSP alignment legislation, and the 11th state overall. The RUSP alignment legislation initiates a three-year timeline in which the screening must begin for new conditions added to the RUSP and requires an annual report to state leadership that outlines the capacity to implement additional RUSP tests [331]. In addition to participating in several projects nationally already noted, a project analyzing the association between NBS analytes measured on a second screen specimen and childhood autism was reported in 2020 [332], a study demonstrating the robustness of the Texas NBS program before and after COVID-19 in South Texas [333], and a 2023 report reviewed cCMV testing in DBSs and found it to be as accurate as traditional urine, saliva, and plasma testing within the first 21 days of life [334]. Texas NBS program personnel have contributed as co-authors to publications in collaboration with other state NBS programs (as examples, see discussions on MPS-II, GAMT, CF in Section 3.1.1).
- **Utah**—The Utah NBS program is operated by the Utah Department of Health, requires two screening specimens on all newborns, one near birth and one at 7–16 days of age, and provides laboratory testing through the Public Health Laboratory (9 tests) and ARUP Laboratories (30+ tests; Department of Pathology, University of Utah). The NBS fee was increased in July 2003 to USD 140, which covers both screens, and two conditions were added to the NBS panel, MPS-I, and PD [335].
  In addition to its progressive work with GAMT [96], noted earlier, significant other NBS research is ongoing in Utah. A 2020 report looked at NBS knowledge and attitudes of midwives and out-of-hospital-birth parents to examine factors that drive NBS nonparticipation looking to improve their educational resources [336]. A review of the importance of timing in diagnosing and treating presymptomatic SMA patients identified through NBS was reported in 2021 with a recommendation for treatment by 14 days of life) [337]. Also in 2021, a data model was described that provides a foundation for implementing a standardized electronic data exchange across NBS programs that can accelerate implementation of electronic data exchange between healthcare providers and NBS programs ultimately improving health outcomes [338]. Two other reports in 2021 dealt with secondary screening testing: a report describing a scalable, exome sequencing-based next-generation sequencing (NGS) pipeline with a priori analysis restriction that can be universally applied to any NBS disorder [339]; and a report discussing how NGS can be used as a NBS secondary testing method, the importance of genomic variant repositories for the annotation and interpretation of variants and barriers to incorporation of NGS and bioinformatics into NBS systems [340]. A 2022 report of focus groups of parents who have interacted, or will soon interact with the NBS system, found generally positive reactions with some frustrations with result communications, reliable program/disorder information, and insurance complexities [341]. A recent report reviewed data related to detection of ALD from the perspective of a reference lab concluding that NBS has resulted in a significant increase in follow-up and presymptomatic case detection [342].

- **Vermont**—Vermont’s NBS specimens are tested as part of the NENSP using the screening laboratory in Massachusetts. The laboratory is currently assessing the capability of testing for MPS-II and GAMT, which were added to the RUSP in 2022 and 2023, respectively [343].
- **Virginia**—A 2020 report provides an educational review of NBS for both CH and CAH including the published studies of one- and two-screen states, concluding that continued method improvements and cutoff adjustments over time have improved case detection for NBS [344]. A 2022 article reviewed the history of attempts in Virginia to add KD to the screening panel concluding that until and unless KD is added to the USA RUSP, it will continue to face an uphill battle for inclusion in state NBS panels [89]. Recognizing the value of NBS, the Governor’s office announced the addition of SMA and ALD to the screening panel in March 2022 [345]
- **Washington**—The Washington State NBS laboratory provides testing, including weekends, for Washington, Hawaii (October 2019), and Idaho (April 2021). Washington is considered a “two screen state” since a second NBS at 1–2 weeks is strongly recommended with high compliance. SMA was added to the screening panel in 2020. An expanded CF DNA testing algorithm was recently initiated (1 July 2023). The Washington NBS program has a research collaboration with the Pacific Northwest Research Institute in the five-year CASCADE (Combined Antibody Screening for Celiac and Diabetes Evaluation) study to demonstrate the feasibility of detecting Type 1 diabetes and celiac disease from DBSs through population-based screening. There is also a collaboration with Key Proteo, Inc. to demonstrate that their novel, multiplexed proteomic-based assay can be used for DBS screening to detect Wilson disease, X-linked agammaglobulinemia, Wiskott-Aldrich syndrome, and adenosine deaminase deficiency [346].
  A 2020 review of the literature involving proximal urea cycle disorders and the possibility of their inclusion in NBS showed that while these conditions meet the medical, diagnostic, treatment, and public health rationales for NBS, screening sensitivity and specificity would need improvement before the screening test would be satisfactory for NBS [347]. A pilot study of 100,000 specimens using a 5-plex LC MS/MS assay for MPS-II, MPS-IIIB, MPS-IVA, MPS-VI, and MPS-VII) demonstrated the feasibility of NBS in a state screening laboratory [348]. A collaborative project involving the Washington NBS program and Seattle Children’s Hospital resulted in the development of a novel proteomics-based assay for rapid and accurate second-tier screening test for PD and MPS I that can possibly reduce the false-positive rate and shorten the clinical follow-up for severe patients resulting in better clinical outcomes [349].

- **West Virginia**—The West Virginia NBS Program is in the Office of Maternal, Child, and Family Health and the Office of Laboratory Services, in the Bureau for Public Health. On 7 March 2023, the NBS program expanded to include LSDs defined by the Code of State Rules: PD, Fabry disease, MPS-I, MPS-II and Morquio syndrome (MPS-IVa) [350]. A 2020 report from West Virginia researchers reported that parents/guardians of children with a positive NBS result reported less difficulty in receiving needed specialty care than parents/guardians of children with genetic conditions were diagnosed later. This finding implies greater population level benefits realized in jurisdictions with expanded screening versus those conducting minimal testing [351].
- **Wisconsin**—The Wisconsin NBS laboratory is part of the Wisconsin State Laboratory of Hygiene located within the University of Wisconsin at Madison. The NBS program as a whole is contained within the laboratory and there is an active research component. Given the tendency of NBS programs to move toward molecular testing and based on their own program experience and literature reports, current applications of molecular technologies in routine NBS practice were tabulated in 2020 to begin to catalog NBS molecular uses and assay principles [352]. NBS thyroid research has continued with three recent publications: TSH reference charts from day 1 until day 14 for moderate-to-late preterm infants were constructed using a state-wide cohort of NBS patients with the intent to examine relationship between age-adjusted NBS TSH percentiles and long-term neurodevelopmental outcomes in future studies [353], a study that tested the hypothesis that term-born small for gestational age (SGA) neonates have elevated NBS TSH concentrations and an increased incidence of CH compared with non-SGA term neonates did not support the hypothesis after adjusting for potential confounders, however, NBS TSH concentration were higher in term SGA neonates compared with term non-SGA neonates [354], and a study that looked at a cohort of preterm infants with higher NBS TSH percentiles, suggesting potential subclinical hypothyroidism, found no prediction of any adverse effect on neurodevelopmental or growth outcomes [355]. Two studies relating to improvements in CAH laboratory processes have also recently been reported: a 2020 report described the use of principal component analysis (PCA—a statistical method that reduces high-dimensional data to a small number of components and captures patterns of association relevant to the outcome of interest) to improve the PPV of the current screening algorithm from 20% to 67% [356]; and a 2022 report noted that multiple 17-OHP cutoff co-variates (birthweight, time of collection) failed to improve the accuracy NBS for 21-OH CAH suggesting the need to use alternative approaches not related to 17-OHP for assay improvements [357].
  The Wisconsin NBS program has continued to assume a leadership research role with CF. In addition to co-authorships in many multi-state and multi-national publications related to CF NBS, Wisconsin researchers have also focused on information and improvements for the local NBS program. In 2020, a comprehensive report outlined the impact of the CFTR gene discovery on CF diagnosis, counseling, and preventive therapy [358]. A 2023 report describes current refinements in NBS algorithms by applying NGS as a screening method to enhance sensitivity and equity while minimizing incidental findings [359]. Even with NGS, the Wisconsin program will require sweat testing on all patients with one identified CFTR variant. In a separate 2023 article, a listing of misperceptions and counter arguments favoring single variant sweat testing are provided [360]. A 2021 report reviewing long-term pulmonary and mortality outcomes of CF patients in the original Wisconsin Cystic Fibrosis Neonatal Screening Project, which ended in 2012, found that, “*NBS alone does not improve pulmonary outcomes in CF, particularly when other risk factors supervene. In an era prior to strict infection control and current therapies, NBS for CF may be associated with worse pulmonary outcomes*” [361].
  As one of the first USA NBS programs to implement SCID screening (pilot in 2008), the longer NBS history has allowed for a 10-year review of referrals to a single referral center describing a broad spectrum of medically actionable and idiopathic T-cell lymphopenia [362]. Additionally, the longer SCID testing experience has allowed for modifications in laboratory procedures, including using multiples of the median values for TREC assays, which eliminates the need for standards with known TREC copy numbers and allows for assay normalization and assay comparisons between different laboratories [363], and data suggesting that TRECs increase at a steady rate as gestational age increases, which provides a rationale for Wisconsin’s NBS program’s recommendation for a second NBS test following a screen-positive SCID result in a premature infant (instead of performing flow cytometry) [364].
  More recently, focus has turned to SMA and ALD. The first year of screening for SMA has been reported in which a multiplex real-time PCR assay was used to detect homozygous *SMN1* exon 7 deletion, triggering a droplet digital PCR assay for *SMN2* copy number assessment. This method’s positive predictive value was reported to be 100%. and facilitated timely clinical follow-up, family counseling, and treatment planning [365]. For ALD, flow-injection MS/MS in a negative ion mode was reportedly used to shorten ALD analysis run-time to 1.7 min, thus maintaining the advantage of negative mode MS for eliminating isobaric interferences that might lead to false-positive screening results [366].

- **Wyoming**—The Wyoming NBS program requires a second screening specimen at 7–14 days of age on all newborns. Screening laboratory services are provided in partnership with the neighboring Colorado NBS program and research activities within the state are minimal. Unlike most other states, Wyoming law requires parental consent for NBS [367].

##### Provincial Activities (Canada) 

The following listing of NBS activities in the various Canadian provinces was created from a review of online information and requests for information from professional colleagues. All NBS programs in Canada have comprehensive websites easily accessible by querying the Internet, thus as with the USA states, the information listed here provides limited insight into the amount and type of research ongoing within the states and other documented newsworthy activities within the 2020–2023 time period. The provincial activity reviews are intentionally brief, and the reader is referred to the program’s website for additional detailed information about the province’s screening requirements. Summary information is included in Section 3.1.3.

- **Alberta**—NBS in Alberta is a collaboration of government and health service organizations providing NBS to newborns in Alberta, the Northwest Territories, and the Kitikmeot region of the Nunavut territory. The Newborn Metabolic Screening Laboratory located at the University of Alberta Hospital (UAH) in Edmonton provides all screening testing except for SCID and second-tier testing for CF, which are performed at the Molecular Diagnostics Laboratory at UAH [368]. NBS for SMA was begun on 28 February 2022 and included a qPCR screening assay to detect the absence of SMN1 exon 7, which was multiplexed with an already established SCID qPCR assay allowing for a smooth cost-effective implementation. The results of screening 47,005 newborns during the first year of the program (prevalence of 1:9401) were recently reported [369]. NBS for classical galactosemia (galactose transferase deficiency—GALT) has been ongoing since April 2019, using a two-tier screening approach, which secondarily identifies infants with glucose-6-phosphate dehydrogenase (G6PD) deficiency. A study of three years of case data shows that reporting G6PD cases “may lead to increased identification of neonatal hyperbilirubinemia and may decrease the incidence of episodes of hemolysis in these patients…[and]… saved a significant number of families from the anxiety associated with the work-up required to rule out a potential GALT diagnosis [370]”.
- **British Columbia** (BC)—The BC NBS program, administered by the Provincial Health Authority, facilitates screening of approximately 45,000 newborns annually in BC and the Yukon. “Newborn Screening BC” is a collaboration between BC Children’s Hospital, BC Women’s Hospital and Health Centre, Provincial Laboratory Medicine Services and Perinatal Services BC. The NBS program was expanded on 1 October 2022 to include SMA, SCID, and BIO [371]. A 2020 study report on the clinical impact of NBS for CAH and the incremental costs in screened vs. unscreened newborns found that while NBS did not result in cost savings, it was cost effective since screened newborns were less likely to require medical transport and had shorter hospital stays resulting in lower hospitalization costs [372]. Another study reported in 2020 found that an IRT-DNA-IRT algorithm, with a repeat IRT measurement for apparent carriers at 21 days, successfully reduced the number of sweat tests required without significantly impacting CF case detection sensitivity [373]. Report of a program study in 2023 described the validation of a second-tier dual derivatization approach with LC-MS/MS to detect 2-methylcitric acid, methylmalonic acid, and total homocysteine in DBS cards amenable to NBS [374].
- **Manitoba**—The Cadham Provincial Laboratory (Winnipeg), which provides NBS, announced program expansion to include SMA on 8 June 2022 [375]. A 2020 report details experiences with GA-I cases detected in Manitoba over a 40-year period that included two cohorts of patients (before and after the introduction of NBS in 2000). In addition to reporting on the outcomes observed, suggestions are included for improving the program going forward [376]. A 2021 report noted the high incidence of SCID in Manitoba related to two founder mutations in the Mennonite and First Nations of Northern Cree ancestry populations. The authors report the development, validation, and implementation of a multiplexed qPCR assay to detect both mutations, which should, “*…decrease the morbidity and mortality of SCID in Manitoban neonates and establish a precedent for future population-specific screening programs in Canada*” [377].
- **New Brunswick** (NB)—NB, along with Prince Edward Island (PEI), is part of the Maritime NBS Program served by the NBS laboratory at the Izaak Walton Killam (IWK) Health Center in Halifax, Nova Scotia. See Nova Scotia for more information.
- **Newfoundland and Labrador**—NBS management and laboratory services are provided by the Ontario newborn screening laboratory [378].
- **Nova Scotia**—The Maritime NBS Program (Nova Scotia, NB and PEI) uses the screening laboratory at IWK Health Center in Halifax, Nova Scotia. There is currently interest in implementing NBS for SMA in the Maritime provinces. A recent report describes the status of SMA NBS in 2022, and illustrates the lack of screening across Canada, including the Maritime provinces [32]. In late 2021 a significant grant (about $322,600) was provided to the Maritime NBS Program by Muscular Dystrophy Canada and Novartis Canada to assist in overcoming the challenges of adding SMA to the NBS panel [379]. As part of the process, a recent epidemiological study looked at SMA prevalence in the region finding an incidence of about 1:11,900 [380]. Another condition, infantile onset CPT-II, has also been suggested as a possibility for screening in a 2021 report [381].
- **Ontario**—The Ontario NBS began expansion in 2006 guided by the USA RUSP. An updated provincial NBS program was also implemented, which was reported to be different from conventional NBS programs. The updated program is a structured and fully funded partnership between the Ministry of Health (MOH), the NBS laboratory, and the provincial treatment centers, each component with a defined role and accountability. Recalled newborns are immediately referred to a regional treatment center for evaluation and further follow-up service coordination [382]. Considerable research has been ongoing in the Ontario NBS program since 2020 in addition to clinical studies and the international collaborations already noted. A 2020 report on the ability of NBS to detect newborns who will develop inflammatory bowel disease (IBD) in childhood found that an expanded panel of metabolites would be necessary for IBD detection [383]. Reports of two 2021 studies addressed the role of primary care providers (PCPs) in NBS for CF: the role of the PCP in notifying the parents of positive NBS findings [384]; and the PCPs’ preferred roles and confidence in caring for infants with a screen-positive CF NBS result and in assisting with CF family planning issues [385]. The latter showed a lack of confidence in addressing carrier issues demonstrating a need for PCP genetics education. A study evaluating the health outcomes of children with CFSPID (or CFRMS) at school age across Canada and the value of the sweat chloride in predicting CF risk [108]. A commentary on the benefits and harms of NBS identified this study as filling an evidence gap potentially useful as a predictive tool useful in maximizing NBS benefits and minimizing harms [386].
  Several other publications in 2021 dealt with NBS issues: a study of the predictive value of NBS analytes found a lack of association with infant mortality between 7 days and 6 months of age [387]; a study of sickle cell carrier status, which is not reported in Ontario NBS, identified statistically significant differences in health services use among sickle carriers relative to controls, with small effect sizes and inconsistent association directions across age groups and health service types indicating that carrier status is likely benign in early childhood [119]; a report recommending testing and follow-up protocols for implementation of NBS for SMA in Ontario included a standardized path to early diagnosis and treatment for optimal screening outcome [388]; a report estimating a minimum prevalence for 22q11.2 deletion of 1:2148 making it one of the more common rare genetic conditions and supporting the importance of NBS for early diagnosis and disorder management [389]; and a report of low TREC copy numbers when screening for SCID likely caused by neonatal abstinence syndrome [66].
  Following the implementation of SMA NBS, a 2022 report of the first year of SMA NBS identified several improvements that could potentially reduce time to treatment: molecular laboratory operation on weekends; reduced specimen transit time; reduced confirmatory testing time; and (4) more efficient approval of treatment medication [390]. Other 2022 reports related to the NBS program concerned prenatal alcohol exposure and transient CH. In the former case, residual DBSs were used as a population resource to estimate the extent of PAE exposure in Ontario by quantifying phosphatidyl ethanol homologues and to use the findings to reassess the effectiveness of current interventions [391]. In the latter case, a possibly more efficient clinical method for differentiating transient from permanent CH was proposed using predictive factors to create a risk score for predicting the likelihood of a successful off-therapy trial at age 1 year [392]. At least three reports in 2023 addressed NBS issues: a report demonstrating how analytical imprecision and biomarker distribution within a population can be used to inform decisions on screening thresholds in general, with NBS for GALT as an example [393]; a report reviewing the pre-analytical factors influencing screen-positive findings for BIO using a time series analysis of print lots of collection cards to demonstrate contributions from both seasonal temperatures and print lots [394]; and a report of a study of parents attitudes about expanded NBS with NGS finding a preference for conditions with supportive interventions in childhood, despite other possible benefits [395].

- **Prince Edward Island** (PEI)—NBS began in PEI in the mid-1960s and is now a part of the Maritime NBS Program in Halifax, Nova Scotia. See Nova Scotia for more information.
- **Quebec**—The Quebec NBS program includes two specimens, each tested for its own screening panel. An initial DBS specimen is collected at 24–48 h of age and a urine specimen is collected at 21 days of age. This program has been described as a “unique-in-the-world” program and has functioned within the provincial healthcare system since the early 1970s with over 3.5 million newborns screened. The urine NBS program is complementary to the blood NBS program, allowing detection of disorders not readily identifiable in blood and provides a unique research opportunity, with informed consent of the parents. A 2021 report described the steps being taken to transition the urine screening program from thin layer chromatography to MS/MS [396]. In addition to a probable French Canadian founder effect for hyperornithinemia–hyperammonemia–homocitrullinuria (HHH) syndrome, which is detectable in the urine screening program, there is also an increased prevalence from a founder effect for vitamin D-dependent rickets type 1A in the Saguenay-Lac-Saint-Jean region, and a report on implementation of a local NBS program was reported in 2022 based on the criteria of the Quebec National Screening Committee [397]. Other NBS recent reports include the description of a case of hyperlysinemia with a profile of cystinuria (but with high urinary lysine) identified with urine screening, further establishing this rare condition as a biochemical abnormality, which may not require any intervention [398], and a report on the benefits of early detection and referral of children with SCD detected by NBS to comprehensive care [399].
- **Saskatchewan**—The Saskatchewan NBS program is administered by the Saskatchewan Health Authority. In February 2022, the program announced its expansion to include screening for SMA, HGB, SCID and cCMV [400].

#### 3.1.3. Tabular Summary of Information (States, Provinces, and Territories)

##### USA States/Territories 

The information in Table 4 was compiled from publicly available sources including state NBS websites, national websites, and personal contacts. In cases where the information available was inconsistent, telephone contacts were initiated to resolve any issues. 

All state programs use a single screening laboratory, which for smaller population states may be a shared public or private laboratory as indicated in the table, and all but a few charge a NBS fee usually collected as a part of the collection card distribution process. State programs that either require or strongly recommend a second screen with high compliance are also noted in the table, an in such cases, any screening fee usually includes both screens. Usual specimen collection times for all states except California occur after 24 h of age with specimens in California collected after 12 h of age. Further details of NBS in each state can be found on the state’s NBS website.

Readers who wish additional information on the methods used for each screening disorder are referred to the federally supported website: https://www.newsteps.org/data-center/state-profiles. This website includes more specific information on methods and analytes for the various screened disorders (among other summary data). Once on the website the user can select a particular state and view its screening profile. By choosing “Disorder” in the profile, the user can see a listing of screened conditions and additional details about methods/biomarkers.

NBS is now present in all USA territories with the pending implementation of NBS in American Samoa. In the case of small birth population territories, NBS specimens are usually sent to screening laboratories in other states or countries utilizing negotiated service contracts. Currently specimens from Guam and Saipan [Commonwealth of the Northern Mariana Islands (CNMI)], including specimens from newborns from families residing on other islands who are born on Guam or Saipan, are sent to Oregon and specimens from the Virgin Islands are sent to Revvity (Pennsylvania). Puerto Rico has its own NBS laboratory and the NBS program currently being organized in American Samoa, for logistical reasons, will likely collaborate with the New Zealand NBS laboratory.

##### Canadian Provinces/Territories

The information in Table 5 was compiled from publicly available sources including provincial NBS websites, other NBS stakeholder websites, scientific publications, and personal contacts. In cases where the information available was inconsistent, email and telephone contacts were initiated for resolution. All provinces use a single screening laboratory, with laboratory sharing for territories with low newborn populations: NBS specimens from New Brunswick and Prince Edward Island are sent the Nova Scotia screening laboratory (Maritime provinces) NBS specimens from Nunavut Territory and Newfoundland and Labrador are sent to NBS screening laboratories in Ontario, Manitoba, and Alberta depending on local health service agreements; NBS specimens from Northwest Territories are sent the Alberta screening laboratory; and NBS specimens from Yukon Territory are sent to the British Columbia screening laboratory (see Table 5 footnotes). Further details of NBS in each province can be found on the province’s NBS website.

### 3.2. Asia–Pacific

**Figure 3:**
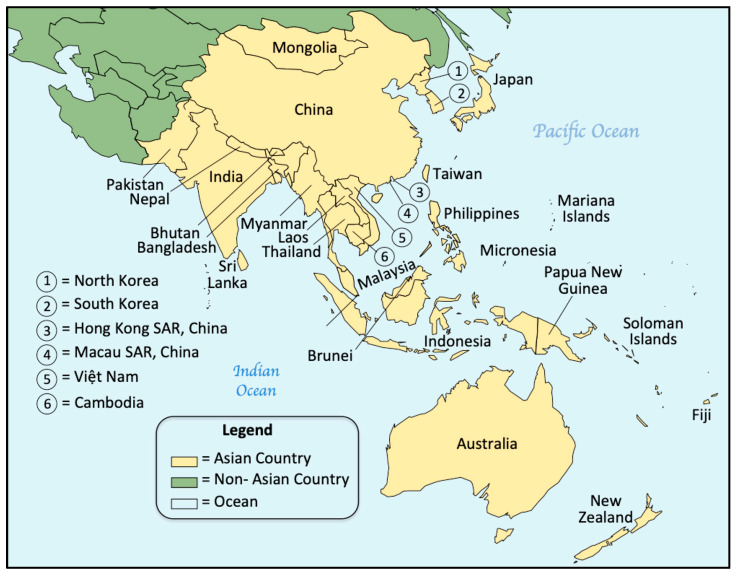
Map showing extent of Asia–Pacific region.

The Asia–Pacific region extends from Mongolia in the north to New Zealand in the south and reaches beyond India to Pakistan in the west (see Figure 3). Of the approximately 134 million births in 2023 (latest reliable statistics), over 43% (57.5 million) are born in the Asia–Pacific region. Three of the four countries with the most births are included in the region—1. India, 2. China, and 4. Pakistan. Across the region there are large variations in country sizes, economics, governmental structure, languages, and cultural sensitivities, each presenting a different challenge with respect to establishing and maintaining sustainable NBS. Vast size differences exist and geographic obstacles including mountains, deserts, and islands create unique opportunities for creativity in NBS service delivery.

For the purposes of this report, we will focus on 28 countries. Little or no information on NBS is available from four countries in the region, Bhutan, Brunei, Korea (North), and Papua New Guinea. Of the 24 remaining countries, NBS activities include little or no organized (sustainable) screening in some (Cambodia, Laos, Nepal, Myanmar), minimally organized screening for at least one disease but still seeking formal government support (Bangladesh, India, Indonesia, Pakistan), organized programs screening for at least one condition and expanding to include ENBS using MS/MS (Mongolia, Sri Lanka, Việt Nam), organized nationwide screening for some conditions and expanding to include MS/MS (Malaysia), and well-organized national programs with ENBS panels of 10 conditions or more (e.g., Australia, China, Hong Kong, Korea (South), New Zealand, Philippines, Japan, Singapore, Thailand, Taiwan). While national NBS panels of conditions exist in most countries, their implementation is sometimes by state or province rather than nationally (e.g., Australia, India, Japan) and a formal NBS law is present in some [China, Philippines, Pakistan (Sindh Province)].

In most Asia–Pacific countries, there are opportunities for expanded screening in private sector birthing facilities and laboratories when a fully functioning ENBS program does not exist. NBS financing schemes vary across the region with the government directly absorbing screening costs fully or partially in some countries, government and private insurance covering some or all costs in some, and patients assuming full financial responsibilities in some (often the challenge in beginning programs). In the case of beginning programs, funds are sometimes available from outside sources (foundations, grants, etc.), but these funds are time-limited and intended to serve as a stopgap while the beginning program sorts out its internal finances. In any case, a sustainable NBS program must have the support (not necessarily financial) of the health ministry in order to succeed, as illustrated by the successful programs within the region.

This section will be divided into a review of recent NBS activities affecting parts of the region or the region as a whole (Section 3.2.1), and activities within the various countries (Section 3.2.2). While this report serves as an update to our 2015 publication on NBS worldwide [16], it will focus on activities from 2020–2023 with some exceptions for activities of special importance or activities in less developed countries. Section 3.2.3 contains tabular information for comparison both within the region and with other regions.

#### 3.2.1. NBS Activities Focused on the Asia–Pacific Region

Many LMICs exist within the region and, building on a network of these countries created in the 1990s by the IAEA [20] and continued through a partnership with the Asia Pacific Society of Human Genetics (APSHG) [401], there continue to be periodic meetings and other electronic interactions to reinforce each other’s implementation and sustainability efforts, to share experiences and ideas, and to set goals for future progress. Through these efforts, several programs have matured and now reach over 90% of newborns (e.g., China, Philippines, Sri Lanka) and examples from these successful programs are being used to encourage others continuing to establish a national NBS presence (e.g., Bangladesh, India, Indonesia, Pakistan). Those countries just beginning (e.g., Cambodia, Laos, Myanmar, Nepal) benefit from the shared experiences and expertise of others in the group as do those who are well along in establishing a sustainable national program (e.g., Mongolia, Việt Nam).

While the 2018 statement of Shibata, et al., “*Expanded newborn screening (ENBS) utilizing tandem mass spectrometry (MS/MS) for inborn metabolic diseases (IMDs), such as organic acidemias, fatty acid oxidation disorders, and amino acid disorders, is increasingly popular but has not yet been introduced in many Asian countries*” [402] remains true in 2024, there is continuing progress in this regard, even in LMICs. There has been successful MS/MS training for screening personnel from multiple laboratories in China, Malaysia, Philippines, Singapore, and Thailand from partners in developed programs abroad and within the region, and all of these have successfully implemented MS/MS screening. The 2019 meeting of the International Society for Neonatal Screening (ISNS), held in China in conjunction with the Asia–Pacific Regional ISNS meeting, provided an excellent forum for regional information exchange and the abstracts of this meeting provide a summary of regional activities, particularly in China [403]. Within the Asia–Pacific region, there are several outstanding NBS research teams (e.g., Australasia, China, Japan, Taiwan) working to improve NBS and create a vibrant future. The recent publication by the Taiwan team on, “The Modern Face of Newborn Screening”, not only describes the successes in improving NBS techniques through the years, but also describes where we might see screening headed in the future [404].

#### 3.2.2. NBS Activities within Countries

- **Australia**—NBS has been ongoing in Australia since 1964. The Standing Committee on Screening of the Human Genetics Society of Australasia/Royal Australasian College of Physicians provides oversight and advice to the NBS programs in both Australia and New Zealand (six laboratories including five Australian States and New Zealand). The NBS program is delivered at the state and territory level with each acting autonomously with funding from the Australian Government through the National Health Reform Agreement [405]. Shortly after our 2015 report, the NBS policy framework and decision-making process became the subject of studies aimed at developing national NBS policies in Australia [406,407]. Currently, there are five independent Australian NBS laboratories (programs) in New South Wales (NSW), Queensland (QLD), South Australia (SA), Victoria (VIC) and Western Australia (WA). The Australian states/territories that do not have NBS laboratories partner with those that do; therefore, specimens from the Australian Capital Territory are sent to NSW, specimens from Tasmania go to SA, and specimens from the Northern Territory are split between QLD and SA [405]. Further screening details are provided in Section 3.2.3.
  Currently, all five programs provide similar testing services with the ability to screen for different conditions. Both SCID and SMA are being added to each program on different timeframes. In 2022, an economic evaluation of NBS for SCID was published suggesting the value of SCID screening on both clinical and economic grounds [408] and government cost savings were also illustrated for a combined NBS approach with SCID and SMA [409]. Several reports involving NBS for SMA, including results of pilot studies, have been recently published as Australian NBS programs consider its possible addition: a prospective description of the experiences in implementing NBS for SMA in NSW [410]; a discussion of the first pilot screening project from August 2018 until January 2021 [411]; a thematic analysis of parents’ perceptions and the psychological impact of NBS for SMA [412]; a “value-for-money” assessment of NBS and gene therapy from a clinical and policy point of view [413]; a study demonstrating that NBS and early access to disease-modifying therapies effectively ameliorate the functional burden and associated comorbidities for affected children [414]; and a study demonstrating the technical feasibility of NBS for SMA using NGS in a multiplex platform may provide simultaneous testing for hundreds of inherited conditions [415]. SMA screening has now been recommended by the Australian Government to be rolled out nationally with support from a research team who will help develop standardized guidelines. NBS for SMA is now available in NSW and WA, and NBS for SCID is available only in NSW. Other laboratories have pilot studies either planned or ongoing.
  Aside from studies of new conditions, studies of long-standing screening conditions have also recently been published, including CH, CAH, and CF: assessing a lower CH (TSH) screening threshold with a concurrent 8-fold increase in the recall rate [416]; harmonizing CH screening protocols across Australasia in response to 50 years of data and variable incidence levels across the six programs [417]; evaluating a two-tier NBS laboratory protocol for CAH in NSW [418]; considering the use of 21-deoxycortisol analysis and LC-MS/MS in the screening/diagnosis protocol for 21-OH CAH [419]; and evaluating increasing median CF survival calculations relative to factors such as NBS, improved care etc. [420]. Looking at other NBS-related research, a 2020 report sought to demonstrate linkages between diabetes, biochemical genetics and NBS suggesting that NBS specimens may be underused in diabetes research [421], and a 2021 publication reported characteristic subtle (nonpathological) changes in neonatal metabolism from fetal metformin exposure evident during NBS [422]. Looking to the future, studies were also reported on the candidate conditions ALD [423] and DMD [424], and the Medical Services Advisory Committee recently announced their recommendations to add ALD and SCD to the Australian NBS panel [425].PD screening was proposed to the Australasian Standing Committee for Screening in 2018 but was not approved prompting publication of a report of PD screening activities in support of NBS [426]. The possibility of NBS for chromosome 15 imprinting disorders using genetic techniques [427] along with the knowledge and attitudes of Australian parents and health professionals towards genomic sequencing in NBS [428] and public perspectives on the risks, benefits, and preferences for implementation of gNBS [429]. A 2023 report notes that the addition of genomic sequencing into population-wide NBS will invariably expand the knowledge of treatable rare diseases, potentially benefiting health over a lifetime. This report discusses the challenges and opportunities ahead, particularly “*the need to generate evidence of benefit and to address the ethical, legal and psychosocial issues*” raised [180]. An Australian multi-disciplinary expert group has now reviewed 604 of a possible 1279 genes for possible inclusion in gNBS and developed a consensus gene list for possible use in systematic harmonization efforts for gNBS in Australia and internationally [430].

- **Bangladesh**—The NBS movement for CH was introduced in Bangladesh in 1999 by Bangladesh Atomic Energy Commission (BAEC) and as a regional project of the IAEA. Studies from local pilots have demonstrated an occasionally effective program suitable for government support/management as part of a policy for combatting congenital diseases [431]. The screening laboratory at the Nuclear Medicine Institute at Bangabandhu Sheikh Mujib Medical University, Dhaka is a well-equipped NBS center under BAEC with 3–4 peripheral NBS centers (currently inactive). Currently, screening coverage is <5% and is not considered sustainable and the lack of a national policy that includes funding remains the biggest challenge [432,433]. A recent survey of the NBS knowledge among health professionals found that NBS for CH, CF, PKU, galactosemia (GAL), HGB, and SCID were available in certain locations and suggested an overall lack of awareness of NBS that needed to be addressed nationally [433]. An investigation of PKU in the Rajshahi Division, Bangladesh led to similar conclusions and a national implementation strategy was outlined [434].
  Other NBS studies on Bangladeshi newborns have been reported with particular emphasis on local incidences of metabolic and other conditions detectable by NBS: a study of LC-MS/MS techniques, establishing cutoff values for various amino acids and acylcarnitines, to assist government authorities in installing and establishing NBS [435]; a Canadian collaboration study of CH and HGB in Matlab, Bangladesh to determine incidences and to validate the use of an offsite remote laboratory [436]; a report on the ease of use of DBSs for remote laboratory testing as part of NBS in LMICs [165]; a study to assess the prevalence of α-thalassemia and provide a model for NBS [437]; and a study to demonstrate the feasibility of NBS for SCD and β-thalassemia [438]. Since NBS for conditions like SCD, thalassemias and other genetic conditions is aided by population education and family counseling when cases are detected, it is important to consider the availability of trained genetic counselors. A 2021 report reviewed the status of genetic counseling in Bangladesh including the current scenario, challenges, and a framework for genetic service implementation [439].
  Studies have been ongoing in Bangladesh and certain other LMIC countries to determine the feasibility of an algorithm (using NBS specimens) developed in Ontario, Canada to reliably estimate the gestational age of newborns when other methods may not be available: a validation protocol for metabolic gestational age assessment in low-resource settings [164]; a study of the cost-effectiveness of a gestational age metabolic algorithm for preterm and small-for-gestational-age classifications [440]; a study to facilitate the use of machine learning prediction of gestational age using metabolic screening markers resistant to ambient temperature transportation [441]; and a recent report on the development and external validation of machine learning algorithms for postnatal gestational age estimation using clinical data and metabolomic markers [166].

- **Cambodia**—NBS in Cambodia is in the beginning phase and has been ongoing only in limited hospitals. The Calmette Hospital, Phnom Penh, Cambodia has been providing NBS for CH and G6PDD. In addition, some private clinics are also offering NBS. The major challenges are the lack of trained specialists in endocrinology and difficulties in securing life-long treatment for diagnosed patients [442].
- **China**—NBS has been ongoing in China since the first CH and PKU pilot in 1981, formalized by law (PKU and CH) in 1994 and management measures in 2009. While somewhat complex, a report of the history of NBS in China until 2012 is available [443]. Today, NBS in China is a complete network system with strict management and quality control throughout. Expert consensus documents have been published under the supervision of the Special Committee of Birth Defects Prevention and Control: organizational management and dry blood sample collection for neonatal inherited metabolic disease screening [444]; diagnosis and treatment of inherited metabolic diseases by NBS [445]; and laboratory testing technical guidelines of neonatal genetic metabolic disease screening [446]. MS/MS testing is now available nationwide, although its implementation may vary by location, and there are over 240 screening centers. NBS is administered at the provincial level, which results in slightly different screening panels as the program expands. There are several recent reports related to expanded NBS with MS/MS: an evaluation of MS/MS implementation in Suqian city (North) [447]; analyses of the cost effectiveness of ENBS in Shenzhen (South) [448] and Zhejiang province (East) [449]; and studies to determine the disease spectrum and genetic characteristics of IEMs detected by ENBS in Xi’an city (Northwest) [450] and Changsha (Central) [451]. A robust system-wide quality management system exists including both laboratories (National Center for Clinical Laboratories) [452] and non-laboratory components [453], sometimes augmented by regional quality improvement activities [454]. A large study was reported in 2021 that determined normal ranges for 35 MS/MS conditions in the Chinese newborn population [455]. Two 2023 reports review IEM findings in both the eastern coastal region [456] and the north [457].
  Research reports concerning ENBS tend to focus on individual conditions or groups of conditions: investigating the biochemical, clinical, and molecular profiles of IVA patients in Quanzhou, China (incidence 1:84,469) [458]; determining the biochemical and genetic characteristics of patients with primary carnitine deficiency (PCD) and identifying the need for genetic testing to improve screening efficiency [459]; screening for mitochondrial carnitine-acylcarnitine cycle disorders in Zhejiang Province [460]; establishing the incidence and identifying 10 novel variants of PCD in Zhejiang Province [461]; establishing the incidence and identifying 5 novel variants of PCD in Quanzhou [462]; establishing the incidence and identifying 6 novel variants of PCD in Ningbo [463]; establishing the incidence for screened FAODs in Chongqing (PCD is most common FAOD) [464]; using second-tier ultra-performance liquid chromatography (UPLC–MS/MS) to screen for short-chain acyl-CoA dehydrogenase deficiency (SCADD) and isobutyryl-CoA dehydrogenase deficiency (IBDD) to reduce referrals [465]; and assessing the long-term prognosis of 35 patients with methionine adenosyl transferase deficiency (incidence ~1:116,161) based on NBS in Zhejiang [466]. Other recently reported investigations include a method for detecting Duchene muscular dystrophy using creatine kinase isoform MM (CK-MM) [467], estimating the DMD frequency in Henan province (1:4370) [468]; and using MS/MS to define the cutoff values and screen for six newborn LSDs in Shandong province (total incidence ~1:3000) [469].
  Looking to the future through an ethics-first, forward-looking lens, a group of Chinese NBS experts published a consensus statement in 2021 on NBS for monogenic diseases (genetic NBS) in China to further standardize the genetic screening system for newborns and provide guidance on the application of NGS in genetic NBS [470]. Chinese researchers continue to report results of pilot studies of various gene panels potentially applicable to genetic NBS: a 573-gene panel in combination with biochemical screening [471]; a 134-gene panel for 74 inborn disorders [472]; a 465-gene panel for 596 disorders as part of the Newborn Screening with Targeted Sequencing (NESTS) program in eight hospitals across China and one hospital in Beijing [473]; and a 135-gene panel for 75 disorders [474]. Related reports provide updates on the current status of genetic NBS and a look at the future [475], the use of NGS as a first-tier screening test [476]; the current attitudes and preconceptions on genetic NBS in the Chinese reproductive-aged population [477], the value of combining traditional NBS and genetic screening to reduce false negatives and to improve the early/accurate identification of CH [478], the viability of incorporating genetic screening for neonatal intrahepatic cholestasis caused by citrin deficiency (NICCD) into the current NBS program [479]; and the identification of increased numbers of actionable variants with fewer false-positive NBS results with WGS [480].
  Research into new procedures and technologies for ENBS not detected through MS/MS has been the subject of at least five studies since 2020: a report on an artificial intelligence (AI) disease model to identify IEMs and decrease the occurrence of false negatives and false positives [481]; a study that develops artificially intelligent disease risk prediction models for quickly, accurately, and with minimal false-positive rates interpreting tandem mass spectrometry data [482]; a report of increased sensitivity and fewer false-positive screens for carnitine-acylcarnitine translocase NBS by using acylcarnitine ratio indices [483]; a pilot study of a commercial assay using matrix-assisted laser desorption/ionization time-of-flight mass spectrometry to screen for SMA [484]; and studies of NBS for ALD using FIA-MS/MS [485,486] and LC MS/MS [487] to detect lysophosphatidylcholines and acylcarnitines. Other published NBS studies include optimizing the PKU cutoff value in Xinjiang Uygur Autonomous Region based on sampling time [488], obtaining the birth prevalence of tetrahydrobiopterin deficiency in China using NBS data [489], improving the sensitivity NBS for CAH by including second-tier steroid profiling and liquid chromatography–tandem mass spectrometry (LC-MS/MS) in Shanghai [490], optimizing delivery and storage conditions to effectively reduce the degradation of certain amino acids and carnitine in DBS to improve assay accuracy and reliability [491], and the clinical features and outcomes of 31 children with CH missed by neonatal screening in Guangzhou [492]. A 2021 review of the national CH data from 245 screening centers in 30 provinces [almost 92 million newborns screened with about 43,000 cases of primary CH detected (1:2145)] between 2013 and 2018 and found a higher incidence of CH in the east than west when all cases were mapped [493]. The cost effectiveness of NBS for congenital cytomegalovirus infection was also studied, but looking at saliva or urine screening instead of blood [494].

- **Hong Kong Special Autonomous Region (SAR), China**—NBS in Hong Kong has been in place since 1984 with NBS for CH and G6PD provided on cord blood at no cost for babies born in public hospitals with maternity services [495]. A private expanded NBS program at the Chinese University of Hong Kong was begun in 2013 and continues today screening for 31 inherited metabolic disease (IMD) conditions [496]. A 2021 report published in 2023 reviewed the progress with the Chinese University NBS program noting the addition of NBS for CF in 2014, CAH in 2016, ALD, SCID, and SMA in 2021 [497]. Since our 2015 report, there have been at least 2 NBS pilots for multiple IMDs, one led by The University of Hong Kong [498] and one government-led [499] resulting in phased-in ENBS using dried bloodspots. The number of IMD disorders screened in the government-led program increased from 21 to 24 in 2016 and then 24 to 26 in 2019 [495]. A government-led pilot of NBS for SCID was conducted after a follow-up review of NBS for SCID recommended its inclusion on the Hong Kong screening panel before it was formally added to the NBS program in 2023 [500]. Building on a healthcare burden and life cost study of spinal muscular atrophy cases in Hong Kong that supports the idea of NBS [501], SMA is currently in pilot testing in Hong Kong [502].
  Three reports have been recently published involving parents: one assessing the content of online parental resources, including information on both urine screening and blood screening [503], and two reviewing positive attitudes of parents and healthcare providers on storage and the use of bloodspots after screening [504,505]. An immunoassay for ceruloplasmin concentration on DBS for Wilson’s disease was successfully modified and automated allowing for its inclusion on NBS panels [506]. To summarize, the government led NBS program in Hong Kong covers 26 IMD conditions, SCID and SMA (pilot testing) whereas the private Chinese University of Hong Kong NBS program covers 31 IMD, SCID and SMA.

- **India**—Despite more than a generation of studies, including government studies, and repeated indications of imminent actions to initiate/recognize a NBS program at the national level, no nationally recognized government-backed NBS program yet exists. While NBS is not widely available in public hospitals where more than 60% of births occur, it is becoming increasingly available in the private sector, including expanded testing. There continue to be data reviews and appeals for government recognition of NBS based on these data: an appeal based on positive experiences from regional (model) programs in Chandigarh, Goa, and Kerala [507]; an appeal for all hospitals in urban areas to initiate NBS for CH, CAH, and G6PD deficiency (the local common disorders) [508,509]; a call for the government to support expanded NBS due to increasing public awareness and large numbers of cases detected annually in regional programs [510]; a review of 1.5 decades of case detection data from three tertiary care centers in Bangalore supporting NBS [511]; a systematic review and meta-analysis of the prevalence, screen positivity rates, and etiology of NBS for CH [512]; an analysis of the biochemical basis, clinical manifestations, treatment advances, and present status of screening [513]; a study of the cost effectiveness and cumulative economic benefits of NBS for CH [514]; and a study to show general acceptance by the public, and the need for increased general awareness of NBS [515].
  A large multicenter program covering over 230,000 newborns in Delhi, supported by the Science and Engineering Research Board, has recently demonstrated the need and feasibility for NBS for CH, CAH, G6PD, BIO and GAL and has provided information on amino acid, fatty acid and organic acid disorders. These data form the basis of a program in Delhi called Mission NEEV (Neonatal Early Evaluation Vision). It envisages NBS for IMDs, along with POC tests for critical congenital heart disease, hearing, retinopathy of prematurity, and visible birth defects [516].
  Reports continue to accumulate on screening experiences despite the lack of national coordination. Reviewing reports from 2020 until now, 2 multi-condition studies have been reported: a 3-year study of CH, CAH, G6PDD, GAL, and PKU in Bengaluru, South India [517]; and a Canadian collaboration study of CH, CAH, G6PDD, GAL, PKU and BIO in Udupi district of South India [518]. Reports continue regarding NBS for endocrinopathies: CH screening, prevalence, and etiology in Indian preterm babies [519]; a report of confirmatory testing from the original DBS to speed diagnosis of CAH (assuming that the DBS and patient match) [520]; a review of the challenges and opportunities resulting from NBS for CAH [521]; a case study of a newborn with unnecessary devastating consequences from CAH resulting from no NBS [522]; a standard operating protocol for routine NBS for CAH in Indian settings [523]; and a discussion of genetic confirmatory testing for CAH from the original DBS as a means of speeding the diagnostic process [520].
  Because the second largest national health burden results from hemoglobinopathies, here is increasing interest in NBS for SCD: a 6-year review of NBS for SCD in the tribal populations of Gujarat and Madhya Pradesh (noting that Indian SCD is not always mild and tertiary care centers are lacking) [524]; a review of the clinical profile of children detected with SCD from NBS in Gujarat and recognizing a high prevalence of α-thalassemia [525]; a description of the development/implementation of a NBS for SCD implemented in 12 SCD-endemic and tribal-dominated primary/community health centers across six districts of India [526]; and an evaluation of a point-of-care test for SCD (HbA, HbS, HbC) suitable for onsite NBS [527]. The Prime Minister’s vision of elimination of SCD by 2047 is an incentive to expand NBS for these diseases by initially targeting pockets in tribal populations as well as areas where SCD is known to be common [528]. The 2020 report on NBS implementation in India, in addition to providing a brief but comprehensive history of NBS in India makes two important points: (1) “*the increasing awareness and programs over the past decade have led to more babies being screened every year*”; and (2) “*the results of the existing programs suggest to the policy makers in India that there is a benefit in implementing a universal NBS program*” [507].

- **Indonesia**—Beginning in 1999, the IAEA aided in starting NBS for CH. A pilot project and health technology assessment was conducted in two hospitals between 2000–2005, which was then followed by the expansion of CH NBS to eight provinces in 2008 (West Sumatra, Jakarta, West Java, Central Java, East Java, Yogyakarta, Bali, and South Sulawesi). Although the MOH released a decree recommending CH NBS in 2014, lack of public awareness and lack of prioritization did not lead to sustainable NBS [529]. An interest in NBS has continued and in 2020 a review of CH and CAH activities in five Indonesian cities from October 2015 to January 2016 reaffirmed the high rates for both conditions, the high false-positive rate when screening for CAH and the overall need for NBS [530]. A 2022 report reviewed the literature over the last decade to identify screening problems and solutions for implementing an improved CH program [531]. In 2022, the coverage of congenital hypothyroidism in Indonesia was 2.3%. The MOH policy reorganized NBS by requiring that all newborns receive screening for CH beginning 1 September 2022 [532]. The year after, the MOH enacted a new decree requiring all healthcare professionals to perform CH NBS as a requirement to claim delivery rates from the national insurance.
  The challenges and future implications of CH NBS in Indonesia were recently analyzed in view of its importance in improving healthcare. Parent refusal, early hospital discharge, and unavailability of specimencollection cards were identified as the most challenges. [533]. At the 67th U.N. Session of the Commission on the Status of Women in May 2023, the Indonesian Minister of Health, presented a keynote address in which he noted that part of the health system transformation underway in Indonesia would include, “*NBS for CH and CCHD for every child born in Indonesia*”. With increasing government commitment and NBS being viewed as a national health priority, the country has seen a stark increase in the capacity of NBS for CH nationally with current screening capacity reaching up to 50,000 samples a week at the end of 2023. While there are no laboratories specifically dedicated for NBS, Indonesia maximizes 12 existing national referral laboratories, each responsible for different provinces. In order to increase screening coverage to 80,000 samples a week in 2024, there are plans to add more laboratories to accommodate the geographic extremes that come with 17,000 islands, 38 provinces, 416 counties, and 98 cities. Additional screening for CAH, G6PD, critical congenital heart disease and hearing is currently in the planning stage by the Directorate of Nutrition and Maternal and Child Health in the MOH [533].

- **Japan**—Japan continues to have a progressive NBS program which began in 1977 with PKU and continues today with over 20 conditions. The screening history has recently been reviewed through a topical collection of articles in this journal, an overview of which is described in the referenced article [534]. Greater details of the history of Japanese NBS are included in the historical review of NBS for CH [535]. As part of this history, Guidelines for Mass Screening of Congenital Hypothyroidism were developed by the Mass Screening Committee of the Japanese Society for Pediatric Endocrinology in 1998, revised in 2014, and slightly revised again in 2021 [536]. Part of the NBS history is also detailed in the 30-year review of lessons learned from CAH screening [537]. And a recent report provides an algorithm for CAH screening using second-tier steroid analysis. The reference ranges for newborns at 4–6 days are also determined [538].
  The history of certain other NBS conditions and procedures has also been recently reviewed: the discovery of galactose mutarotase (GALM) deficiency in Japan [539]; the measurement of glycosaminoglycans by LC-MS/MS for mucopolysaccharidoses [540]; the development of second-tier LC-MS/MS as part of screening for acylcarnitines, acylglycines, amino acids, and organic acids [541]; the introduction of NBS for ALD and peroxisomal disorders in Japan [542]; and DBS screening for glycogen storage disease type 1a [543,544]. Several studies of MS/MS detectable disorders have also been reported: the clinical and genetic characteristics of patients with mild hyperphenylalaninemia [545]; changes in the frequencies of genetic variants of VLCADD since the implementation of ENBS [546]; the discovery of an unexpectedly high incidence of patients with mild propionic acidemia resulting from a common variant [547]; strict therapeutic observation to prevent sudden death in infants with CPT2 deficiency even if the child appears to have a mild clinical case [548]; CPT2 deficiency does not always have high sensitivity and specificity at the screening level, requiring retesting in a relatively large number of cases. Recently, Japanese researchers have identified markers with high sensitivity and specificity for the detection of CPT2 deficiency by MS/MS [549]; and a pilot study that examines the detection of specific subtypes of MMA and HCY [550]. The long-term outcomes of Japanese adult patients with both HCY [551] and PKU [552] were compared before and after the introduction of NBS, thus demonstrating the importance of lasting strict management in adult life.
  NBS methodology studies have included the report of a simple screening method to effectively detect isovaleric acidemia by distinguishing isovalerylcarnitine (i-C5) from pivaloylcarnitine (p-C5) by flow-injection MS/MS [553]. There have also been several reports describing NBS for LSDs: the use of gene analysis with NBS for the definitive diagnosis of PD [554]; a study of the frequency of infantile onset PD screening in Kumamoto and Fukuoka prefectures from 2013–2020 preliminary to expansion throughout Japan [555]; a review of the past, present, and future of NBS for mucopolysaccharidoses [556]; a discussion of NBS screening for Fabry disease in western Japan [557]; a study of novel Fabry disease-associated pathogenic variants in Japanese patients detected by newborn and high-risk screening [558]; and a study of DBS detection of bile acid metabolites for bile acid synthesis disorders, Zellweger spectrum disorder, and Niemann–Pick type C1 and the possibility of NBS [559].
  There is increased interest presently in adding SMA to the Japanese screening panel and several reports informing NBS for SMA have recently been published: a brief review of the current status of SMA treatment and the value of SMA NBS with new drugs [560]; a report on the pilot SMA screening project in Osaka confirming the usefulness of their workflow [561]; a report on pilot NBS using data from Osaka and Hyogo provinces to estimate incidences and to confirm the need for SMA NBS [562]; a review of disease history, treatment options, and NBS algorithm, along with the results of 1 year of SMA screening in Kumamoto Prefecture [563]; a report on 18 months of NBS for SMA in Hyogo Prefecture [564]; validation studies of DBS procedures for detecting SMA carriers suitable for implementation in NBS laboratories [565,566]; and the results of a new simple screening system based on DNA melting peak analysis applicable to a real-world NBS program for SMA with or without PCR equipment [567]. Looking to the future, there are two recent reports on the use of NGS in NBS: the use of 24 causative genes for NGS following a positive screen with TSH [568]; and the use of a 349-gene panel to detect genetic mutations associated with primary immunodeficiencies (PIDs) following a positive screening result using TREC/KREC analysis for SCID [569].
  Recently, a model for the quantitative assessment of NBS in Japan was developed using the analytic hierarchy process [570]. The emergence of new target NBS conditions such as SMA, LSDs, SCID, or ALD in newly expanded NBS may require a re-evaluation of the hierarchical disorder assignments.

- **Korea (South) [Democratic Republic of Korea]**—NBS for IEM was adopted by the Ministry of Health and Social Affairs for low-income families in 1991 and expanded to cover all newborns in 1997. There are currently 11 conditions included in routine NBS [571]. Recent publications have dealt with the implementation of NGS in NBS and laboratory issues. A doctoral dissertation in 2019 focused on development of a target NGS panel for NBS [572], which led to a report on the implementation of a 198-gene NGS panel at a hospital in Seoul and its potential to aid in reducing false-positive screening results [573]. Also addressing false-positive screening results was a study of their effect on parents through an analysis of NBS posts regarding NBS from an online parenting community [574]. Other recent publications have focused on laboratory issues. An online report of the annual external quality assurance for the public screening laboratory is published annually [575]. The Korea Research Institute of Standards and Science (KRISS) has developed a limited number of DBS Certified Reference Materials (CRMs) to enhance the reliability of NBS testing, with plans to develop more CRMs for other NBS markers [576]. A report of an issue with a commercial FIA-MS/MS procedure sought to alert product users of the potential problem and its solution [577].
- **Laos**—Sustainable NBS is under development in Laos. Previously, a 2008–2010 pilot project in the large maternity hospitals in Vientiane utilized laboratory services in Hamburg, Germany to demonstrate the feasibility of NBS for CH and CAH in Laos. The availability of cell phones facilitated retesting follow-up; however, early discharge and physician knowledge were identified as challenges [578]. In 2019, the German group once again assisted with initiation of NBS, but limited to CH. Local laboratory capability was established, workshops were held for health personnel, and screening was again established [579]. Screening with financial support from Else Kröner-Fresenius-Stiftung, a German foundation, is scheduled to continue through 2024 after which time it will require local support [580].
- **Macau SAR, China**—NBS for G6PD began in 1977 and since then it has expanded slowly in collaboration with other programs, including the Shanghai Xinhua Hospital NBS laboratory. Specimens from Macau babies born in Hong Kong are routinely analyzed in the Xcelom laboratory in Hong Kong [581].
- **Malaysia**—NBS in Malaysia began with cord-blood screening for glucose-6-phosphate dehydrogenase deficiency (G6PD deficiency) in 1980. In 2003, the Ministry of Health Malaysia implemented a nationwide, stepwise, congenital hypothyroidism (CH) screening program for all babies delivered in government hospitals [582], including a pilot project with MS/MS [583]. While all government maternity facilities offer G6PD and CH screening, ENBS is available only at four main centers on request (i.e., it is not compulsory) and a lack of awareness among clinicians and laboratory diagnostic facilities has likely resulted in under-reported cases [584]. Researchers have taken advantage of MS/MS technology to review and develop reference ranges for Carnitine-Acylcarnitine Translocase (CACT) deficiency and Carnitine Palmitoyl Transferase 2 (CPT II) deficiency [585]. Other research has centered on experiences in applying NGS to screening for thalassemia [586], validating a POC screening test for G6PDD [587], the need for additional training when setting up a new condition based on experiences with CH in 12 government hospitals and 20 health clinics [588], and the need for a mandated IEM-related course for certain healthcare disciplines at the university level [589].
- **Mongolia**—With some support from the IAEA, pilot NBS for CH began in Mongolia in 2000. The program has grown from three hospitals in 2000 to five city hospitals and five rural hospitals by 2023 primarily in central Mongolia [590,591]. During this period, more than 44,000 infants were examined for CH and CAH, and about 130 children were detected early and monitored. Also, the G6PD pilot study revealed a prevalence of 23.5%. In early 2023, a second laboratory (Center for Newborn Screening and Diagnosis) was established, and its scope of activities expanded. In Hovd (western border) and Dornod (eastern border) provinces, NBS branch centers were established and, in these provinces, in addition to CH and CAH, screening for G6PDD, GAL and CF was started. Beginning in 2024, health insurance will cover NBS for the five diseases. The NBS program continues to grow slowly, and the screening team is working to overcome sustainability challenges [591].
- **Myanmar**—Despite having participants in some of the IAEA regional NBS activities in the late 1990s, a sustainable public NBS program does not yet exist in Myanmar. There is some NBS availability in the private sector. There is interest in conducting a formal pilot study for at least CH.
- **Nepal**—There is currently no recognized NBS program in Nepal, although some screening is available in private settings. There have been at least 2 pilot studies in recent years, and while providing incidence information for various conditions and demonstrating that NBS is possible, no program has yet to be sustained: a pilot in collaboration with a NBS laboratory in Switzerland to lay the foundation for NBS and determine reference ranges for various analytes [592]; and a pilot collaboration between Paropakar Maternity and Women’s Hospital and a commercial NBS laboratory in India (NeoGen Labs Pvt. Ltd.; Bangalore, Karnataka, India) that confirmed the feasibility of NBS [593]. The latter pilot suggested that CH, CF, SCD, and LSDs should be considered for screening, although specimen transport issues slowed reporting on occasion. Inclusion of these conditions is supported by case reports and reviews in the literature: a summary of case reports of metabolic conditions, which also suggests inclusion of G6PD deficiency and Wilson’s Disease [594]; and a study of Hb S/S in the Tharu population, an indigenous sub-population living in a malaria endemic area in west Nepal [595].
- **New Zealand**—NZ was one of the first countries to establish a national NBS program. While it functions independently, NZ enjoys a collaborative NBS relationship with Australia as part of the Human Genetics Society of Australasia. Several significant updates to screening NZ protocols and policies have been published since our 2015 report. In 2015, new evidence was assessed by clinical experts and others, and it was decided that screening for six carboxylase deficiencies (3MCC; HMG; MCD; BKT; 3MGA; 2M3HBA) had no clinical benefit for the child and therefore NBS was discontinued [596]. In June 2017, screening for CUD was also discontinued due to screening sensitivity and treatment/safety concerns [597]. NBS for SCID was initiated in December 2017 [598].
  In addition to reviewing the factors affecting screening accuracy for CAH in NZ [125], considerable attention also has been given to improving the detection of CAH by including LC-MS/MS second-tier testing [599,600,601,602,603]. Similarly, attention to NBS system improvements in CH screening has been documented: adjustments in the TSH laboratory protocol improving case detection and PPV [604]; examination of the effect that demographic variables (e.g., ethnicity and age at time specimen collection) have on newborn TSH levels [605]; review of the actions to take when obtaining discordant results (case study) [606]; and caution against reducing TSH screening cutoffs to levels at which the diagnosis may not offer long-term benefit for those detected (TSH values less than 15 mIU/L (whole blood) found not to be associated with long-term hypothyroidism or cognitive impairment in NZ) [607].
  NBS reporting protocols are expected to result in clear and concise information exchange. A recent report in NZ emphasized that, to enable meaningful comparisons, CF screening reports should include the screening algorithm and target along with clarification of the steps in the screening pathway included in the assessment, the screening algorithm, and screening target [608]. To encourage faster recall returns for follow-up testing overall, a structured protocol for follow-up of inadequate and borderline positive NBS (including text messages to specimen submitters) was recently introduced [609].
  Other reports related to currently screened conditions included a review of the risk of having GALT if NBS results indicate borderline-positive galactose metabolites [610], and an assessment of the genotype–phenotype correlations in CPT1A deficiency detected by NBS, a condition more prevalent in Pacific populations (e.g., NZ and Hawaii) [611]. A study of the potential for NBS for neonatal hypoglycemia also has been recently reported with a call for urgent research to determine the optimal method of screening and which infants would benefit from screening and treatment [612]. The public health agency Te Whatu Ora announced the addition of SMA to the NBS panel in September 2023, with its implementation to take 12 months [613].

- **Pakistan**—NBS in Pakistan continues in its developing stage. Only one prefecture, Sindh, in the southeast has an NBS law to establish and integrate a sustainable NBS system within the public health delivery system, which became operational in all 29 Sindh districts on 1 January 2020 [614]. Several reviews of NBS operations and their impact on implementation in Pakistan have recently been published: a review of CH and the high burden of disease in Pakistan supporting a national NBS program [615]; a systematic review of CH and a plan for NBS implementation in Pakistan [616]; suggestions as to how a NBS system might best be organized and the conditions that might be a part of a Pakistan screening panel with CH as a prototype and then extending the panel to include CAH, BIO, GAL, G6PDD, SCD, [617]; and a review of why and how screening systems are configured [618]. Encouraging the expansion of NBS efforts around the country, in 2019, Aga Khan University, in collaboration with the Pakistan Society of Chemical Pathology, organized a meeting of genetics researchers, pathologists, and child health specialists from public and private sector organizations to launch an advocacy group, the Pakistan Inherited Metabolic Disease Network (Pak-IMD-Net). This group intends to overcome research challenges, medical education, and clinical practice related to IEMs and to encourage policy reforms to expand access to NBS [619].
  Recent project reports seeking to determine baseline information on screenable conditions found cases of HCY, MPS, and GAMT through biochemical screening of intellectually disabled patients [620]. Reports of reference range studies have included a multicenter study of TSH [621], a study of BIO [622], and a DBS study of various amino acids, succinylacetone and acylcarnitine [623]. Looking to the future, a study of an international partnership with an overseas facility performing NGS testing for PID patients suggests that resource-limited regions may require immunophenotyping and NGS in rather than TREC analysis, a screening strategy that can be met with through global partnerships [624]. Considering quality improvement of screening where it exists locally, NBS awareness in a hospital performing NBS for CH was found deficient in an audit of short-term follow-up with TSH results [625], a study of pregnant women found a similar lack of understanding about NBS resulting in fewer screens [626], and another study of parents found an increased knowledge from proper counseling led to better uptake NBS [627].

- **Philippines**—Since beginning as a pilot in 1996 and becoming sustainable through a national law in 2004, the Philippine NBS program has continued to grow in both coverage and disorders included in screening. Reports of expansion include successful implementation of screening for HGB [628] and the addition of MS/MS screening [629] so that the current program includes 29 conditions and newborn coverage exceeds 90%. To improve the quality of the overall program, a Philippine performance evaluation and assessment scheme (PPEAS) was developed as a means of defining responsibilities for various program components (e.g., NBS specimen collection facilities, NBS screening centers, etc.) [630]. Continuity clinics were organized and formalized to provide continuing long-term care assessment [631]. To maximize NBS acceptance, a hospital assessment of maternal utilization of NBS recommended that hospitals assign specific hospital staff to actively offer and be responsible for NBS prior to discharge [632]. A lack of knowledge about the roles of physicians, nurses, and medical students within the NBS were evident in a recent survey suggesting a role for enhanced NBS education among health professionals [633]. Most recently, a review of the challenges in sustaining NBS in the Philippine NBS program has been published [634].
- **Singapore**—NBS for G6PD deficiency began in 1965 followed by CH screening 25 years later. Universal hearing screening and screening for multiple IEMs were introduced as national programs in 2003 and 2006, respectively, with current NBS coverage at almost 100% without a screening law. A comprehensive review of the history of NBS and other screening activities with newborns was published in 2021 and notes the possibility of Singapore becoming a screening laboratory center for other developing programs in the region (e.g., Brunei, Myanmar, Laos, and Cambodia) [635]. An editorial discussing the history of NBS for CH also was recently published [636] along with an article advising on some of the issues related to cord blood screening for CH (e.g., mode of newborn delivery and TSH measurement method) [637]. In 2018, screening for SCAD and IBDD were removed from the screening panel. In 2019, NBS was expanded to include five additional disorders (CAH, BIO, SCID, GAL, CF). Along with the NBS for SCID came questions about its implementation in settings where BCG (bacille Calmette-Guerin) vaccination was given to newborns. The results of implementing SCID NBS in Singapore were published in 2022 and showed that patients with transient non-SCID T-cell lymphopenia and no underlying primary immunodeficiency can tolerate the BCG vaccination [638].
- **Sri Lanka**—NBS for CH using DBSs was introduced in 2006 and a regional screening center was established in 2008. In 2010, NBS began for all newborns in the Southern province, which led to financial commitments from the MOH. Program expansion to other provinces continued in 2016 and today the program is sustained and reaching over 95% of newborns in line with the National Strategic for Plan for Maternal and Newborn Health, 2017–2025 which set a goal of >95% of newborns screened annually for CH [639]. A 2018 report outlines the early history of the program and describes an audit process for five provinces (Southern, Uva, Sabaragamuwa, Central and Eastern) describing the need for improved turnaround times and community awareness [640]. A 2021 cost-effectiveness study confirmed the value of NBS for CH [641]. Another 2021 report described the negative health effects of CAH over two decades of unscreened newborns and sought to emphasize the need for adding CAH to the screening panel [642].
- **Taiwan**—A report on the history of NBS was published in 2019 [643] and updated in 2023 [404]. NBS reaches all newborns, is free to those in need, and subsidized for others. Perhaps the most progressive screening program in the region, the Taiwan NBS program was the first to add PD in 2005, and program researchers have contributed significant knowledge about both infantile onset—and later-onset PD through the years [644,645,646]. Research into various multiplex assays for Pompe and other LSDs recently have included a report of screening 70,000 newborns with an 8-plex assay [PD, Fabry disease, Gaucher disease, MPS-I, MPS-II, MPS-3B, MPS-4A (Morquio syndrome), and MPS-6] [647], and evaluation of initial cutoff values, rates of screen positives, and genotypes when testing over 100,000 Taiwanese newborns for MPS-I, -II, and -6 [648]. A long-term follow-up study of MPS-II from April 2015 until April 2022 provided baseline data on cases for future comparisons regarding treatment and outcome [649]. Research studies on a number of other NBS have also recently been published: a study of ALD showing a high incidence of null variants identified through NBS [650]; a report of the first 50,000 Taiwanese newborns screened for DMD [651]; an assessment of the significance of compound motor action potential (CMAP) amplitude in patients identified through NBS [652]; a report on second-tier molecular testing to improve NBS detection of citrin deficiency [653]; a review of the improved prognosis in early-identified newborns versus later-onset symptomatic infants with neonatal intrahepatic cholestasis caused by citrin deficiency (NICCD), supporting the need for NBS [654]; a report clarifying the etiology of false positives in NBS for citrullinemia. [655]; and a pilot study of a matrix metalloproteinase-7 as a possible screening test for biliary atresia using DBS collected at 3 days of age [656].
- **Thailand**—Neonatal screening for phenylketonuria (PKU) was introduced as a pilot project in Thailand from 1992–1995, and mass screening was started in 1996 by the Department of Medical Sciences, Ministry of Public Health. [657] NBS is now a mandated program under universal health coverage reaching over 95% of Thai newborns for the past 10 years. There is no parental written consent for NBS, but consent is required to share the leftover bloodspots for future research purposes. The National Health Security Office (NHSO), in October 2022, launched universal healthcare coverage ENBS for 40 treatable inborn metabolic diseases. Currently, there are seven NBS centers covering about one-third of Thai newborns for ENBS using MS/MS. The expansion of new NBS centers and the increase in capability of the existing centers is ongoing and seeks to achieve the government’s goal of 100% coverage by the end of 2024. These NBS centers have become a national network alongside seven rare disease centers, with administrative support from the Thai NHSO and the Ministry of Public Health (Department of Medical Science and Department of Health). The ENBS network meets every 3–4 months to encourage knowledge sharing, mutual assistance, and development of new NBS centers. They also share clinical geneticist consultants and confirmatory tests such as plasma amino acid, urine organic acid and genetic testing, as needed [658,659]. A 2018 report from southern Thailand found a significant increase in CH case detection after the implementation of NBS [660]. Prior to launching the national MS/MS program, a study funded by NHSO in Bangkok showed that Siriraj Hospital could provide ENBS to the 15 public hospitals in Bangkok [661]. A recent assessment of the knowledge and attitudes of parents regarding NBS found a generally favorable outlook but a need for increased awareness and education about the program [662].
- **Việt Nam**—Since 2006, the government has provided NBS for CH and G6PDD as part of government-paid healthcare. Other tests for a fee may be available, particularly at international and referral hospitals (typical listing shown in reference) [663]. The largest maternity and children hospital in the country offers NBS for CAH. Recently, a MOH spokesperson recently noted that in December 2020, the Prime Minister approved a program expanding the provision of NBS and prenatal screening, diagnosis, and treatment of some diseases and disorders until 2030 with a goal of “*…90% of newborn babies for at least 5 of the most common congenital health problems …*”. “*By 2025, five regional screening and diagnosis centers will be upgraded, and two new similar centers will be built in the northern midland and mountainous region and the Central Highlands*”. Facilities supplying NBS and other health services *“…will be set up in 90% of the communal-level localities nationwide*”. “*Prenatal and newborn screening and diagnosis facilities will also be developed at obstetric and pediatric hospitals and general hospitals in 56 provinces and cities*” [664]. Looking to the future, a recent study notes the lack of structured economic evaluations of NBS in LMICs to determine the best way forward and uses Việt Nam NBS as the case study [665]. Also looking to the future, studies of at least two screenable “rare” conditions in the country have been reviewed, both with founder effects linked to the Kinh ethnic population, and both noting the need for NBS: a report of MPS-I cases noting five patients in unrelated families from the same small community with the same previously unreported variant [666]; and a review of 10 years’ experience at a medical center in northern Việt Nam with 41 BKT deficiency patients [667].

#### 3.2.3. Tabular NBS Data for Asia–Pacific

NBS is growing and changes occur frequently. The data displayed in Table 6 represent the best Asia-Pacific NBS data available at the time of this writing. Readers should check local resources to validate the current NBS panel. In keeping with the other tables in this article, we have used UNICEF population, birth, and infant mortality data from the same time period. This is the first time that we have attempted to obtain the number of screening laboratories and the age at which specimen collection is recommended in order to review the degree to which there is international harmony. Perhaps most striking is the degree of variation in the time at which specimen collection is recommended. The reader is referred to other tables in this report that display similar findings.

### 3.3. Europe

For our report and consistent with the 2021 update on NBS in Europe [668], we will consider Europe to consist of 54 countries, which includes Russia and five former Russia republics east of the Ural Mountains (Kazakhstan, Kyrgyzstan, Tajikistan, Turkmenistan, Uzbekistan), even though Europe is generally considered to end at the Urals. The total population of these countries is around 924 million, with about 10 million births annually. Note that the smaller countries of Andorra, Liechtenstein, Monaco, and San Marino have their NBS covered by France, Switzerland, France, and Italy respectively. In keeping with ISNS regional definitions, Israel will be included as part of Europe [668].

**Figure 4:**
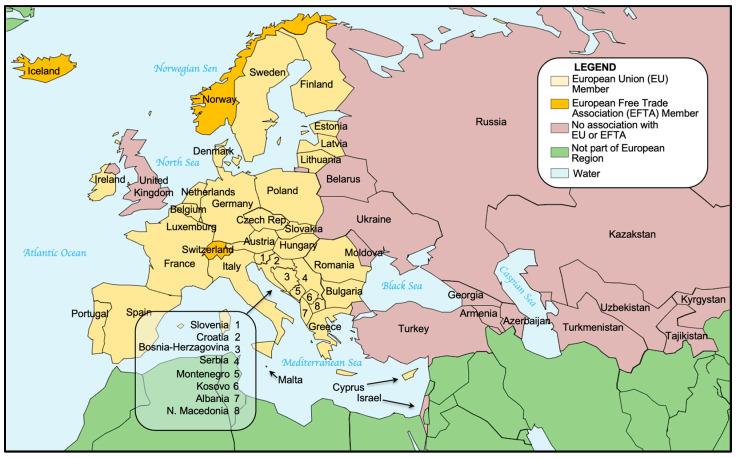
Map showing the European region.

NBS has been ongoing in Europe since the mid-1960s. Figure 4 provides a map view of the region illustrating those countries that are part of either the European Union (EU) or the European Free Trade Association. A color-coded historical NBS map may be found in reference [668]. While there is some NBS in individual hospitals, there are no official NBS programs in Albania, Tajikistan, and much of Kosovo. Our report will review recent published activities that are general to the European Region as a whole (Section 3.3.1), recent published activities within the individual countries (Section 3.3.2), and a tabular summary of NBS progress in the region. Because there is a 2021 update on European NBS [668] and a separate 2021 update on Southeastern Europe [669], we will build on these reports by focusing our review on published NBS-related activities in 2020–2023 (in some cases references may be slightly older to illustrate a particular point or in cases where there is limited published information on a country’s activities).

#### 3.3.1. NBS Activities Focused on the European Region

Since our 2015 report, there has been much NBS activity in Europe including discussions of a consensus screening panel and a possible regional reference center. Three articles recently have reviewed the variations in NBS programs within Europe, including the political, economic, and environmental factors (healthcare structure, pressure from professional groups, etc.) influencing NBS policy making [668,669,670]. The reader is referred to these reports for a comprehensive overview of European NBS issues and activities prior to 2021. While there are also many recent publications of European and/or international patient treatment/management (clinical) guidance for conditions and groups of conditions included in NBS, these are considered beyond the scope of this report and will not be included here. Newborn hearing screening (NHS) and congenital critical heart defect screening are also considered outside of the scope of this report. For information on NHS, the reader is referred to a comprehensive three-part series on NHS activities in 47 primarily European countries [671,672,673].

##### Review of NBS Harmonization in Europe 

Within the EU, individual countries make their own national policies, as with public health generally. Advisory reports are usually designed to inform on best NBS screening practices, carefully avoiding interference with national health policies and respecting the autonomy of national governments. In 2009, the European Commission [European Union’s (EU’s) executive body], launched a tender on NBS with deliverables aimed at improving and harmonizing NBS across Europe. Creation of an EU Network of Experts on NBS (EUNENBS) with representation from all member states was required [674]. An inventory of NBS activities in all countries west of Russia was created through an online survey and the results were published in two parts: (1) the period from bloodspot to screening result [675]; and (2) the steps from screening laboratory results to treatment, follow-up, and quality assurance (including a listing of suggested conditions for inclusion in European NBS programs [676]. Using the survey results and consulting with (1) professional and scientific organizations involved in NBS, (2) additional experts (e.g., ethics), (3) a representative of the U.S. Secretary’s Advisory Committee on Heritable Disorders in Newborns and Children, and (4) various advocacy organizations, the EUNENBS debated and created a comprehensive listing of 70 expert opinions on various aspects of the NBS system, which was presented to the EU Committee of Experts on Rare Diseases (EUCERD) [674].

In 2013, EUCERD reviewed the three NBS Tender deliverables: a report on the NBS practices in all Member States; the EUNENBS expert opinion document on the development of European NBS policies (which included a decision-making matrix); and an executive report to the European Commission on NBS in the European Union. These actions were summarized in an official Opinion to the European Commission. The Opinion listed 11 “Topics for potential European Collaboration” but no topics were prioritized, and health care was left as a matter for individual member states [677,678]. Late in 2018, an editorial suggested that the EU Commission should instruct the Steering Group on Health Promotion, Disease Prevention and Management of Non-Communicable Diseases take up a role in providing equal access to NBS for all newborns in the EU [679].

In 2019, EURORDIS (Rare Diseases Europe), a patient advocacy group, established a NBS Working Group to review current NBS policies and practices across Member Countries. Their goal was to develop principles for harmonizing NBS programs to maximize benefits and improved outcomes for newborns with rare diseases [680]. A position paper was developed emphasizing 11 areas including the need for European-wide standards on timing, sample collection methods, follow-up, and information for parents [670], and the need for storage of bloodspot samples in national biobanks for research purposes with appropriate safeguards for data protection and data access [680]. This approach should be distinguished from the Screen4Rare stakeholder platform (see next paragraph), as the demands of EURORDIS exceed what is considered to be within the limits of the Wilson and Jungner criteria.

In 2020, building on collaborations established during EUNENBS survey development and analysis activities, a follow-up survey was again distributed to assess NBS progress and achievements across Europe during the 2010–2020 period. Survey results showed significant program growth, stability, and technical improvements supporting the value of improved collaborations in the region [668]. In the interim, a multi-stakeholder platform, Screen4Rare, was launched by the International Patient Organization for Primary Immunodeficiencies (IPOPI), the ISNS, and the European Society for Immunodeficiencies (ESID) aiming to “*exchange knowledge and best practices on NBS for rare diseases*”. Screen4Rare initiated a “Call to Action—Newborn Screening for Rare Diseases” to the European Commission and Member States [681]. This collaboration expanded to include the European Reference Networks (ERNs) [including the European Reference Network for Metabolic Disorders (MetabERN) and the Rare Immunodeficiency, Auto Inflammatory and Auto Immune Disease (RITA) network], and together, focused on NBS as a system, and proposed ten elements for effective operation of NBS programs in Europe [682]. As the basis for their work, Screen4Rare seeks to adhere to the Wilson and Jungner criteria [683].

In 2021, the Slovenian Presidency of the Council of the EU convened a technical meeting on “Achieving Equity and Innovation in Newborn Screening and in Familial Hypercholesterolemia Paediatric Screening across Europe”. The 150 participants from over 40 countries included NBS leaders, patient representatives, members of the European Reference Networks for Rare Diseases and interested policy makers. Among the objectives of the meeting was the creation of “*a dedicated expert forum at EU level to bring together policy makers, patient group representatives and professionals to share experience, explore the options and offer impartial advice*”. An Executive Summary of the meeting has been published and includes a listing of the key points identified in the stakeholders’ discussion and initiatives needing urgent actions. The latter includes the formation of an independent NBS-EAC whose roles and responsibilities include, among others, facilitating collaborations and sharing of best practices, development of key performance indicators, establishment of case definitions, and consideration of genomics and related issues [670]. In 2022, a technical meeting, “Early Diagnosis of Patients with Rare Disorders in the EU: Crucial Role of Newborn Screening”, was held under the Czech EU presidency bringing together key stakeholders to discuss progress in the workstreams of the Screen4Rare initiative: consistency in the case definitions, interoperable case registries, and a blueprint for the “NBS pathway” for optimal NBS practice in the EU. The meeting supported developing key good NBS practices performance indicators with recommendations for data collection and governance in EU Member States [684].

##### NBS in Southeastern Europe 

In 2021, a report on NBS in Southeastern Europe (with a population of 76 million) showed NBS programs were not harmonized with the more developed European countries. Twelve Southeastern Europe countries/regions [Bosnia and Herzegovina (BIH—Federation of BIH, BIH—Republic of Srpska), Bulgaria, Croatia, Greece, Hungary, Kosovo, North Macedonia, Malta, Montenegro, Romania, Serbia, Slovenia) collaborated to assess the main characteristics of their NBS programs and plans. Comparison with a similar report in 2013/2014 showed that the regional situation had modestly improved. Overall, the status of NBS programs in Southeastern Europe is variable and still underdeveloped (or even non-existent) in some of the countries. The 2021 report also suggested an international taskforce to assist with implementation and harmonization of basic NBS services [669]. An earlier report on PKU screening and management in 11 countries/regions in Southeastern Europe (Albania, Bulgaria, Bosnia and Herzegovina, Croatia, Kosovo, Macedonia, Moldova, Montenegro, Romania, Serbia, Slovenia) confirmed that NBS was underdeveloped and noted that PKU management was behind internationally established standards-of-care [685].

##### Other European Regional NBS Activities 

Providing information about NBS to parents is recognized as an integral part of the screening process and was reported to be available either as written brochures or on websites in over 90% of European programs in 2020 (up from 60% in 2010) [668]. A 2018 content analysis of information for parents examined the differences in the way European countries inform parents about NBS and how they compared to certain knowledge aspects derived from the scientific literature [686]. A 2021 report provided survey data on whether any legally binding provisions, guidelines, or recommendations exist regarding the provision of NBS parents’ information across Europe. The lack of harmonization in providing NBS information further emphasizes the need for comprehensive guidelines at the European level [687].

The prioritization of conditions for the addition to European screening panels by the EUNENBS in 2012 did not preclude innovations in ranking conditions for prioritization [676]. In 2021, a point-based scoring algorithm to prioritize disorders for inclusion in NBS programs was created and used to assess the six disorders included in the UK NBS program [688]. This algorithm was used in a separate study to evaluate and prioritize 48 conditions (including 21 LSDs) that might meet the future needs of European NBS programs. Of these, 35 scores were deemed sufficient by the authors to fulfill the Wilson and Jungner screening principles and the authors recommended that these 35 conditions be included in European NBS programs after analyzing the economic, societal, and political impact of each condition [689,690,691]. Initiatives that develop frameworks and access the applicability of Wilson and Jungner criteria are expected to grow in the future emphasizing the continuing need for a European advisory body of experts.

Looking to the future and suitability for NBS, several congenital conditions have drawn closer scrutiny. As an example, a European Neuromuscular Centre (ENMC) workshop was held in 2019 to review the currently available information on the technical, ethical, economical, and practical aspects of NBS for SMA, and to identify gaps in knowledge relative to NBS follow-up and case identification [692]. A 2020 global survey queried 152 countries, obtaining 87 responses, and found wide variations in funding, screening methodology, program organization, and consent processes. Many respondents indicated that the paucity of cost/benefit data was the major obstacle to NBS implementation [693]. Working to bring together all stakeholders with a shared vision of NBS, SMA Europe established the European Alliance for NBS in SMA and published a white paper making the case for a wider introduction of population wide NBS and summarizing the current understanding and scientific consensus on NBS. Their stated goal is NBS for SMA in all European countries by 2025 [694].

Screening conditions with recent European regional activities include organic acidurias (OAs), sickle cell disease (SCD), and CF. OA harmonization arguments and efforts were published in 2017 based on a survey of EU States [695]. While stressing that expanded panels are not necessarily better panels (the panel is only a part of the screening system, that should also assure proper diagnostic and therapeutic follow-up for all cases identified by screening), an overview of the dimension of screening panels is given in Section 3.3.3. For SCD, a 2-day Pan-European consensus conference was held in 2018 to determine the status of NBS for SCD in Europe and to develop consensus-based guidelines on NBS screening methodology [696]. Both the World Health Organization (WHO) and United Nations (UN) have identified SCD as a global health burden and global migration is leading to more diverse populations and a higher prevalence of SCD in Europe. NBS for SCD is currently included only in a limited number of European NBS programs [697].

For CF, the most common screening protocol in 2017 was IRT-DNA [698]. However, a 2020 report noted that four countries and one local region in Europe had begun using Pancreatitis-Associated Protein (PAP) in their screening protocols [Netherlands (2011), Germany (2016), Portugal (2016), Austria (2017), and Catalonia (2018)], but not necessarily in the same way [699]. The addition of PAP analysis to the screening protocol avoids carrier detection, lowers the rate of detection of CFSPID [called CRMS in the U.S.], and seems to have advantages in detecting CF in ethnically diverse populations. A 2022 survey found 22 national and 34 regional (in four countries) CF NBS programs in Europe. A wide variety of screening algorithms were in use, but most national programs were using DNA as a second-tier screen. Reported barriers to establishing CF NBS in 2022 included cost and political inertia [700]. Other pertinent recent CF publications include a 2020 history of CF screening focusing on the evolution of screening protocols since the 1970s [104], a complementary look at how the lifespan of CF patients has grown over the years [701], a report on international approaches to transmitting positive CF NBS findings [702], a report on the need for more consistency in definitions and data acquisition with CF NBS [703]; and key outcomes for evaluating CF NBS performance [704].

There are several other studies and publications worth noting that were directed at the European region as a whole. A 2021 report discusses the challenges and debates concerning carrier detection/reporting as a part of NBS for rare diseases [705]. A 2022 review illustrates the benefits and limitations of patient registries on rare disease research focusing on inherited metabolic diseases (IMDs) [706]. A 2022 report points to the large numbers of refugees and immigrants from sub-Saharan Africa and Middle East countries arriving in Europe, the many newborns born on the way to Europe or in migrant camps, and their lack of access to NBS. To this end, this report notes that the Asylum, Migration, and Integration Fund (AMIF), established for the period 2021–2027 with a budget of EUR 9.9 billion, was initiated to enhance national capacities and ameliorate migration management and may be a source of funding to assist with NBS [707]. A 2024 report found multivariate independent component analysis (ICA) potentially useful as a tool in analyzing NBS laboratory data [708].

#### 3.3.2. NBS Activities within Countries

The following country reviews seek to give a glimpse of activities ongoing in Europe in the past few years. If a country is not listed, activities may have been limited, likely occurring in earlier years. While some historic references may be present, this section is not intended to give a detailed history of NBS in each country. Rather, it is intended to illustrate the level of activity currently ongoing as an aid to understanding the degree of progress in NBS implementation and/or expansion.

- **Albania**—Although there has been an NHS program as a component of the multicenter EUSCREEN project in Albania since 2018, DBS NBS has not yet been implemented [668,709]. A review of the circumstances in Albania compared to Germany and the UK have been used as an illustrative NBS model [710].
- **Armenia**—The Republic of Armenia implemented universal NBS for CH in 2012. In 2019, the screening records from 2012 through 2016 were analyzed looking at iodine nutrition status, including time dependence and regional variation and suggesting TSH as a means of monitoring iodine status [711]. In 2018, a Turkmenistan delegation visited to review NBS program operation as a possible model to be copied [712].
- **Austria**—The National Austrian Newborn Screening Program for inherited metabolic and endocrine disorders was implemented in the late 1960s by the Federal Ministry of Health in collaboration with the Ministry of Finance and the Medical University of Vienna. In addition to a recent review of the 100-year history of the Austrian national birth registry of IMDs [713], a 50-year review of the Austrian NBS program is also available [714]. Recent discussions within the NBS community have focused on whether and how to introduce PAP into the CF screening protocol [715]. Additionally, the NBS program added SMA in 2021 and SCID in 2022 [716].
- **Azerbaijan**—A study of CH incidence in newborns reported in 2012 [717] and showed an incidence of 1:666, indicating a pressing need for NBS. In 2020, a study of hemoglobinopathies in newborns found the presence of Hb S, Hb D, and pathological genes for α- and β-thalassemia favoring the initiation of NBS for HGB [718], and a 2022 report detailed the data from a NBS study of GALT [719]. Other information on NBS is scarce. A 2022 UNICEF Country Report noted that during the last part of 2022, over 6000 newborns received NBS [720].
- **Belgium**—Rather than a national NBS program, two regional programs exist in the French-speaking and Dutch-speaking communities, each with slightly differing screening panels. A 2017 report detailed experiences in establishing and evaluating screening methodologies for PD, Fabry disease and MPS I using liquid chromatography–tandem mass spectrometry [721]. A 3-year SMA pilot in Liège begun in 2018 has now expanded to full-scale screening throughout the country [722,723]. A recent publication provides real-world cost-effectiveness information on SMA NBS in Belgium [724]. The experiences of 20 years of limited screening for SCD and the potential impact of adding it to the NBS panel were reported with the hope of adding HGB to the screening panels [725]. A 2020 study in Flanders evaluated the cost effectiveness of four NBS strategies for CF [IRT-DNA (immunoreactive trypsinogen, cystic fibrosis transmembrane conductance regulator (*CFTR*) gene mutation analysis), IRT-PAP (pancreatitis-associated protein), IRT-PAP-DNA, and IRT-PAP-DNA-EGA (extended *CFTR* gene analysis)], assessing whether each met the guidelines of the European Cystic Fibrosis Society [726]. A systematic literature review in 2021 identified seven neuromuscular disorders that might be candidates for addition to the Belgium NBS program [SMA, DMD, myotonic dystrophy type 1 (MD 1), PD, ALD, MLD, and KD] [727].
- **Bulgaria**—While the extent of NBS in Bulgaria remains limited, studies have been initiated looking to future expansion. In 2017, a study was conducted that assessed attitudes and opinions on the potential use of whole-genome sequencing (WGS) with traditional NBS. Both pediatricians and geneticists considered selective WGS as a potential option for improving Bulgarian NBS [728]. The results of a pilot study demonstrating the feasibility of SCID screening were recently reported [729]. Cases of arginase deficiency in the Roma area point to the need for ARG screening in this region [730].
- **Croatia**—The Croatia genomics activities, including the NBS program, were recently included as part of an online workshop [731]. Building on a NBS program that began screening for PKU in 1978, the centralized testing laboratory at the Zagreb University Hospital Center recently added SMA screening for its expanding panel [732].
- **Cyprus**—The Cypriot neonatal metabolic screening program began started in 1990 as a civil society effort by a charity, the Centre for Preventive Paediatrics [733]. Ten-year data showed a higher prevalence of CH in Cyprus than in some of the surrounding countries. While a pilot was previously initiated for DMD, the current screening program continues to screen only for CH and PKU, with the Centre responsible for all aspects of the screening system (inviting parents to participate, identifying screen positives, diagnosing conditions, communicating with physicians, assessing long term outcomes, educating the public, and participating in public health policy formation.
- **Czechia**—Within the country, there are two clusters of laboratories for NBS, one in the Bohemia Region and another in the Moravia/Silesa Region. Each cluster has three laboratories (one for MS/MS, one for immunoanalytical, and one for second-tier genetic testing for CF, and screening has included 18 conditions since 2016 [734]. Pilot testing for SMA and SCID began in January 2022 in Prague and Moravia [735]. Recent research has focused on the epidemiology of diseases detected by NBS [736], with particular emphasis on low birthweight neonates [737] and the growth patterns associated with NBS-detected CAH [738]. A study of the way parents are informed about NBS and the variables associated with their awareness has resulted in several new educational measures including seminars for healthcare providers and the development and distribution of new educational materials [739].
- **Denmark**—In 2020, researchers described expanded NBS in Denmark, the Faroe Islands and Greenland (the reach of the Danish NBS program), by reviewing a project that began in 2002 and ended in 2019 [740,741]. In the interim CF (in 2016) [742] and SCID (in 2020) [743] became the 17th and 18th conditions added to the Danish NBS panel, and the first case of CF in a native Inuit was detected through NBS in Greenland [744]. In preparation for the addition of other conditions, development of a multiplex assay for SCID, SMA, and X-linked agammaglobulinemia (XLA) was reported [745] along with a MS/MS procedure for galactose-1-phosphate, reportedly superior to the traditional GALT enzyme analysis [746]. The addition of second-tier molecular testing to the Danish NBS program was described in a 2021 report along with the challenges and possible pitfalls of second-tier genetic testing [747]. Also noting the value of second-tier testing was a 10-year review of salt-wasting cases of CAH detected by NBS [748].
  The secondary use of NBS specimens for other purposes after NBS is a topic of discussion internationally. A 2019 report sought to identify and criticize some of the research uses of specimens contained in the Danish NBS biobank [749], which elicited a response describing the history, organization, and beneficial uses of specimens in this biobank, [750]. A 2022 report discussed the experiences of mothers with both collection and uses of NBS specimens noting their general acceptance of research re-use of the specimens [751]. And a 2023 report sought to provide an understanding of the advantages and possibilities of biobank research in Denmark given the huge number of specimens, the presence of at least a dozen other biobanks, and a unique civil registration number assigned to each Danish citizen [752]. Recent examples of biobank usage include a joint study with the California biobank to develop and validate a new commercial kit for creatine kinase to screen for Duchenne muscular dystrophy (DMD) [753] and LC-MS/MS-based studies of untargeted metabolomics in NBS specimens to define metabolomic profiles for autism [754]. Analyses of specimens in the Danish NBS biobank have been used retrospectively for other epidemiological metabolomic studies [755].

- **Estonia**—The NBS program in Estonia was re-engineered in 2014 as part of a doctoral project [756]. Vitamin B_12_ deficiency was included as a NBS pilot and after 2 years, and an incidence of 1:3000, was continued on the screening panel [757]. Several patient reviews have been completed in the past few years looking both to review the NBS program and to consider new conditions for screening. In 2018, a retrospective overview of PKU patients identified a high incidence of PKU estimated at 1:6700 [758]. In addition to a survey of SMA cases to define their extent in the population preparatory to possible inclusion on the NBS panel, a 30-year study of the prevalence of IMDs in Estonia was initiated to define both their prevalence and the effectiveness of new methods for diagnosis [759,760].
- **Finland**—A regional SCID pilot was initiated in 2019. The Council for Choices in Health Care in Finland (COHERE Finland) recommended NBS for SCID in 2020 and SCID was added to the national NBS panel during 2021–2022 [761]. Similarly, SMA was recommended but not implemented. A proposal for SMA screening has been sent to the Ministry of Social Affairs and Health and is awaiting evaluation [762]. A 2020 report on TYR-I showed improved prognosis from NBS [763]. A 2023 report on the incidence of CH and the proportions of mild, moderate, and severe disease, over the 24-year study period, showed no difference in incidence [764].
- **France**—NBS began in France in 1967 and its history and status recently have been reviewed [765,766,767]. NBS is mandatory and French overseas territories are included in the screening program. There are 17 regional screening centers (12 mainland and 5 overseas), each associated with a university hospital and a regional health agency. There is a national coordinating center, which is overseen by an epidemiological commission that monitors program effectiveness and a biological commission that defines the screening algorithms, including pertinent biomarkers and cutoffs. A national steering committee in the French MOH provides general NBS policies for NBS and recommended seven new metabolic conditions at the end of 2022 [768]. The history, outcomes, and analytical accuracy of other NBS conditions have been reviewed: PKU [769], CAH [770], TYR-I (with SUAC) [771], CF [772], CH (with an appeal to lower the screening cutoff) [773], and SCD [774]. A recent study of parents’ opinions about the NBS process found that there is a preference for more and better information about NBS during pregnancy and the information should focus on the non-mandatory nature of NBS and the need for informed consent for parents choosing to screen their newborns [775].
  A number of reports on CF screening recently have been published: a cost-effectiveness analysis that compared four CF neonatal screening strategies with or without DNA testing to assess the value of combining cost effectiveness and ethics evaluation in health policy development (NBS laboratory protocol) [776]; studies analyzing inconclusive diagnoses after a screen-positive test (CFTR issues) including gene-sequencing and genetic counseling [777,778,779,780]; and review of a centralized tracking process to optimize the CF screening program [781]. Similarly, NBS for SCD, presently a targeted condition based on the geographical origins of both parents (malaria endemic areas) and a family history of SCD, has been the subject of several reports: screening and case management [782]; possible harmonization of HPLC result interpretations by applying multiple of median cutoff and ratios [783]; evaluation of POC testing [SickleSCAN™ (BioMedomics, Morrisville, NC USA)] [784]; and a study of targeted screening failures over the years (pushing for universal NBS for SCD—recommended by the Health Technical Agency since 2022) [785].
  As a prerequisite for addition to the French NBS panel, feasibility and cost-effectiveness studies must be completed along with assay validation. Studies supporting the addition of SCID include performance of the TREC assay for SCID [786], a review of the evidence supporting SCID screening in France [787], and the status of screening implementation, including lessons learned [788]. Recent studies supporting the addition of other conditions also exist: a systematic review of NBS test accuracy for TYR-I using succinylacetone [789]; a world view of practices and pitfalls in defining an algorithm for primary carnitine deficiency screening [790]; and a review of costs and quality of life of patients with SMA [791].
  Two reports concern conditions already on the screening panel: for CAH, a rise in 17-OHP may be observed in newborns exposed to drugs [792]; and for CH, a change in the screening algorithm has been proposed to allow the detection of hyperthyroidism [131]. While there are a number of studies concerning the addition of NBS for cCMV, each concerned saliva testing instead of blood and will not be discussed here. Finally, a 2021 report provided an overview of the biological techniques currently used for NBS and suggested technological changes were inevitable [793].

- **Germany**—German NBS is coordinated through a national directive of the Federal Joint Committee (Gemeinsamer Bundesausschuss). Regional pilot studies looking at feasibility, diagnostics, and health benefits are required for new conditions [794]. Recent reports provide an overview of the current NBS program and some of the ongoing issues: a report detailing the legal background for NBS and related issues [795]; the potentials and limitations of second-tier testing in NBS [796]; a report detailing each of the NBS conditions currently included in NBS and the intricacies of their inclusion and follow-up [797]; a review of 20 years of screening for CAH and CH emphasizing the need for sustainable case registries for all screened conditions to better measure screening outcome [798]; and a thought provoking discussion on genomic medicine and the challenges and suggestions for incorporation of genomic NBS into public health programs, including how to balance expected benefits against possible harms to children and their families [799].
  The addition of SMA and SCD to the screening panel in 2021 has resulted in several reports. For SMA, the focus has been on pilot testing data and the logistics of national expansion, the additional challenges of screening and diagnosis for a new screening condition, and the impact on parents [800,801,802,803,804]. For SCD, a brief narrative reviewing the pathway for adding SCD to the screening panel has been published [805]. Additionally, a report reviews the value of NBS for HGB in recognizing the current immigration patterns across Europe, the needs of the population, and the value of increased case recognition [806]. Recognizing the need to address the transition from pediatric to adult medicine as the screened population ages, a guideline for transition has also been developed [807]. A 2022 report provides pilot testing data for a multi-analyte MS/MS procedure that simultaneously detects SCD, BIO, and TYR-I [808], and a 2023 report provides information on a combination multiplex qPCR and MS/MS procedure for simultaneously detecting SCD, SMA, and SCID [809].
  Recent reports on other conditions on the screening panel include CF (added 2016) and SCID (added 2019). The SCID report is a review of the first four years of NBS on the largest SCID screening population in Europe noting the apparent success of the TREC assay procedure in case detection [810]. CF studies documented the feasibility of using an IRT/PAP screening protocol [811] and a recent publication reported on the influence of season, storage temperature, and time of sample collection on PAP screening algorithms [812]. A DNA-based third tier for all children with a screen-positive PAP was shown to significantly improve the PPV [813]. The current CF-NBS algorithm combines a three-step protocol (IRT/PAP/DNA) with a “safety-net”, lassifying an ultra-high IRT (>99.9 percentile) as a positive screen directly. The PPV using this algorithm is about 0.2 and adaptation is currently under discussion. Parents’ satisfaction with a more centralized system of confirmatory CF diagnostics has also been reported [814]. Two other reports reviewed the outcomes of screening cases detected over time. One looked at clinical outcome of cases reported between 1999 and 2016 [815], and the other reviewed the annual screening quality reports between 2006 and 2018 [816]. Both found that NBS was successful. As noted in other reports, tracking and case registries were a program deficiency. Other pilot studies have included the rare LSD, metachromatic leukodystrophy [817], and cystinosis, a rare autosomal recessive systemic disease with high morbidity and mortality, both using NGS methodologies as part of the screening algorithm [818].
  The possibility of NBS for vitamin B_12_ deficiency has also been reported including strategies, results, and public health implications [819], outcomes of early treatment resulting from NBS [820], and the results of screening 1.2 million newborns for MMA (and vitamin B_12_ deficiency) using a two-tier screening strategy [821]. A collaborative pilot study conducted at three NBS sites in Germany evaluated 18 candidate disorders for German NBS, many using second-tier strategies, with the majority found suitable for inclusion on the screening panel [822]. Several reports have targeted IVA NBS (added in 2005). Not only have researchers looked at clinical outcome and the issues raised by identification of mild variants [823], but also difficulties with high false-positive rates due to maternal use of pivaloylester-containing antibiotics, which is reduced by the use second-tier testing to differentiate C5 isomers [824].
  Machine learning (ML) is becoming increasingly popular as a second-tier digital test for testing sensitivity improvement and a literature review was recently published [825]. ML has now been used to improve the reliability of the IVA screening by combining linear discriminant analysis and ridge logistic regression to maintain 100% sensitivity and reduce false-positive screens by almost 70% [826]. Despite the successes of the German NBS program, there are still issues that can be improved (PPV, tracking, education, etc.) as noted in the referenced comments [827]. A recent report reviewed the requirements for NBS infrastructure and screening procedures through a literature search as part of a project “on the quality and shortcomings of the NBS pathway in Germany” [828].

- **Greece**—NBS dates to the mid-1970s and a detailed look at NBS expansion in Greece exists [829]. A recent publication provides a mini review of the psychological impact on parents of children who have received an inconclusive diagnosis for cystic fibrosis following newborn screening [830]. Looking to the future, a commercial venture to contact at least 1000 families for inclusion in a WGS project looking at over 400 NBS conditions has been announced and is moving forward with plans to reach all Greek newborns by 2027 [831]. The project, BeginNGS, includes the genomic data platform Lifebit (London, U.K.) in collaboration with PlumCare RWE (Delaware, USA), the Rady Children’s Institute for Genomic Medicine (San Diego) and the National Organization of Public Health (Greece).
- **Hungary**—Expanded NBS was introduced in Hungary in 2007 and experiences in detecting vitamin B_12_ deficiency and in diagnosing neonatal/infantile vitamin B_12_ deficiency have been described [832]. A collaboration between universities in Hungary and Germany identified potential interferents in the analysis of methylmalonic acid, which has not previously been reported and represents a pitfall in clinical diagnostics and NBS [833]. Because no commercial quality control material is available for the second-tier NBS CAH assay, a 2021 report compared five different QC preparation approaches used in routine diagnostics for CAH on the concentrations of cortisol, 21-deoxycortisol, 11-deoxycortisol, 4-androstenedione, and 17-hydroxyprogesterone in dried bloodspots [834]. Also in 2021, a report detailed the issues relating to NBS for SMA in Hungary, with particular emphasis on the baby Zente case and the need to have routine NBS for SMA [835].
- **Iceland**—A 2023 thesis describes a completed CF screening pilot suggesting the addition of CF to the NBS panel in the near future [836]. Two metabolomic studies were reported in 2021. One found that low birth weight and extremely macrosomic newborns showed dissimilar metabolomic profiles when compared to appropriate-for-gestational age neonates [837]. The other estimated potential differences in neonatal metabolomic profiles at birth and at the time of NBS by delivery mode and found small differences, if any, and concluded that the mode of delivery does not affect the results of NBS [838].
- **Ireland**—The NBS program in Ireland is one of the oldest national programs in the world, having started with PKU in February 1966 [839]. To facilitate NBS expansion systematically and logically, a 2021 guide, “Review of processes in use to inform the expansion of newborn bloodspot screening programmes”, was published [840]. The importance of early diagnosis and skilled multidisciplinary team management is highlighted in another 2021 report showing the value of NBS by reviewing outcomes of MSUD patients detected through NBS since 1972 [841]. In 2022, the Irish NBS program guide featured summary information on adenosine deaminase-deficient SCID (ADA-SCID), which was added in 2022, and updated program consent and feeding requirements [839]. The National Screening Advisory Committee (NSAC) previously recommended inclusion of SCID (not just ADA-SCID) to the NBS panel and in early 2023, this recommendation was accepted by the MOH [842]. The NSAC has also noted the importance of SMA as the next condition and elevated the consideration of SCD, TYR-I, and BIO for the screening panel while suggesting that NBS for PD was not a priority due to its low incidence. Discussions on the prioritization of some 30 other conditions are ongoing.
  In 2020, a publication on CF NBS, initiated in 2011, brought accolades from the CF community for having “achieved a model CF program” [843,844]. A separate report provided evidence that NBS improved growth, reduced hospitalization for acute episodes, and delayed P. aeruginosa acquisition to age 3 [845]. Beginning first with CF and then expanding to NBS in general, a series of studies from 2016 to 2019 sought to determine the level of knowledge about NBS in the lay community as an aid to developing better educational outreach [846,847,848]. Prior to inclusion of SCID on the panel earlier this year, a study showing its high prevalence in Ireland and the need for NBS was reported in 2021 [849]. Studies supporting the addition of other conditions have also been recently published including one on CAH and another on metachromatic leukodystrophy [850,851].

- **Italy**—Expanded NBS became required by law in 2016 in all 20 autonomous regions and includes over 40 screened conditions paid by the government. The law not only lists the conditions included in screening, but also other program requirements (consent, specimen collection, result communication, follow-up, etc.) [852]. Further description of the expanded program and the results of the first two years of testing from 15 of 16 screening laboratories are provided in a 2022 report. Also included is a description of the national landscape regarding screening, molecular confirmatory testing, and case management [853]. Additional information on the history and legal aspects of the NBS program is reviewed in a recent editorial [854].
  Variations in screening and diagnosis protocols between regions have resulted in a number of regional reports on screening and diagnosis outcomes. Since 2020, at least three studies have focused on BIO: a 12-year data review showing a high incidence in the Tuscany and Umbria regions [855]; a study describing experiences in disease management (and unanswered questions) in Verona [856]; and a report documenting a higher rate of partial BIO than predicted in Abruzzo [857]. CF screening experiences also have been reported: improved program efficacy when DNA testing was added to the screening protocol in Tuscany [858]; a six-center study (Ancona, Brescia, Florence, Milan, Naples, Rome) of the psychological impact on parents receiving an inconclusive diagnosis following NBS [859]; and the impact of incorporating PAP into the screening protocol (IRT/PAP/DNA) to decrease screen-positive inconclusive diagnoses [860]. Several reports have focused on screening for various LSDs: 5.5 year review of NBS for Fabry disease in Northeast Italy including clinical, biochemical, and molecular features of detected patients [861]; 7 year review of NBS for PD in Veneto (Northeast Italy) including infantile- and late-onset disease [862]; a review of MPS-I screening in Padua and the inclusion of a second-tier testing to improve specificity [863]; and a comprehensive review of NBS for MPS-I [864]. An earlier report detailed the Tuscany experiences with NBS for PD, Fabry disease, and MPS-I [865]. Other disease reviews have also been recently published: an overview of galactose metabolism, molecular genetics, NBS and novel treatments potentially able to prevent long-term disease complications [866]; and a review of DMD including a proposed NBS pilot project protocol [867].
  Decreasing unnecessary recall to resolve a positive or inconclusive screening results is an ongoing goal of NBS programs and a discussion of various approaches to its accomplishment has been noted [868]. Several reports of improved screening sensitivity have been published: adding DNA or PAP to the screening protocol in Tuscany (previously mentioned) [858,860]; second-tier testing for CAH in the Northeast region of Italy [869]; and changing the CH cutoff in Western Sicily (to decrease late-diagnosed cases) [870]. Additionally, improvements in CH case detection have been noted in certain cases: use of a lower cutoff on second specimens (when two specimens are required) [871]; and analysis of a second specimen in the case of twins [872]. Preterm newborns are at higher risk for CH and may require a different targeted screening protocol. A study of births in Piedmont indicated that their current two-screen protocol for all newborns was sufficient [873]. In the Apulia region, relocation of the screening laboratory to a more central location has also been shown to improve clinical care for CH through more timely initiation of treatment and improved case management [874].
  The results of pilot efforts aimed at expanding the NBS panel of conditions continue to be reported: SCD—first report of screening for an entire province [875]; SCD—the feasibility of a multicentric NBS program and epidemiology in two northern areas demonstrating a high percentage of Caucasian carriers (impossible to identify in targeted NBS [876]; ALD—a three-tiered pilot in Lombardy started in 2021 (FIA-MS/MS, ultra-high performance liquid chromatography–tandem mass spectrometry (UHPLC-MS/MS), and focused NGS (genetic confirmation) [877]; and SMA—a pilot in Lazio and Tuscany to determine the proper clinical assessments to systematically evaluate the possible presence of early neurological signs of SMA [878]. Other reports have included the successful inclusion of ADA-SCID [879] and purine nucleoside phosphorylase-deficient SCID (added earlier) [880] on the screening panel, detection of SCAD in family members as a result of NBS [881], mild elevations of citrulline detected through NBS [882] and a possible algorithm to guide the diagnostic process, and a pilot demonstrating the value of dried bloodspot analysis of cCMV in infants with hearing loss [883,884]. A recent report reviews the eight-year experience with screening and follow-up of approximately 250,000 newborns screened for four LSDs (PD, MPS-I, Fabry disease, and Gaucher disease) [885].

- **Kazakhstan**—NBS for PKU has been ongoing since 2007 and the 10-year history has been reviewed in the Russian language and describes an increasing coverage [886]. Kazakhstan has 16 regions and 3 megalopolises, with screening coverage of approximately 92% [669]. A recent report defines the limits of a study to review various IEMs detectable with LC-MS/MS to determine their frequency in Kazakhstan as part of the effort to expand NBS [887].
- **Latvia**—A recent report on the 25-year CF diagnostic data in Latvia, including the first report on NBS for CF has been published. Two and one-half years of NBS data were available and indicated a lower disease incidence than expected based on calculations from previous F508del carrier detection studies. There is concern about false negatives and the current IRT-IRT-DNA protocol may soon change to IRT-DNA-IRT [888]. A report on NBS for SMA reviewed implementation issues and improvements in a pilot study that showed the SMA 5q procedure employed could be applied to the whole of Latvia to facilitate early diagnosis and more effective treatment [889].
- **Lithuania**—NBS has been implemented at the Centre for Medical Genetics of Vilnius University Hospital Santaros Klinikos since 1975, testing for four diseases 3–5 days after birth. Funding is from the Compulsory Health Insurance Fund and NBS is not mandatory. Expanded screening for 30 or more conditions is available on request [890]. A project to compare TSH levels in newborns to evaluate iodine status showed mild iodine deficiency in some areas and a need to reevaluate the proposed satisfactory level of TSH vs. iodine level [891]. A 2021 review of CAH case detection showed 100% sensitivity and specificity of NBS in detecting classical SW CAH, but a poor (4%) positive predictive value [892].
- **Luxembourg**—NBS in Luxembourg was recently expanded to include screening for CF in addition to PKU (1968), CH (1978), CAH (2001), and MCAD (2008) [893].
- **North Macedonia**—As part of the MOH’s National program for mothers and children’s care, NBS for PKU and some 30 inborn errors of metabolism by LC/MS/MS has been ongoing in six larger delivery facilities across Macedonia since 2011 [894]. Earlier NBS for both PKU and CH have been documented [895]. Complementing earlier reports [896,897], data from the national CH NBS program over 20 years has recently been analyzed and the prevalence, along with geographic, and ethnic variations, reported [898]. Following a 6-month pilot, NBS for CF by IRT-IRT was introduced in April 2019, and 2-year data show a 4 times higher prevalence of Albanians compared to Macedonians (1:4530 vs. 1:1284) [899].
- **Malta**—NBS began with a for CH and HGB in 2017 [697,900]. Utilizing funds from Norway and other countries from the European economic zone, screening for PKU was started in 2018 [901].
- **Netherlands**—NBS was initiated in 1974. The Ministry of Welfare and Sport defines NBS policy and is responsible for funding and facilitation of program coordination, which is a responsibility of the Center for Population Screening (CPS) of the National Institute for Public Health and the Environment (RIVM). An independent national scientific advisory body summarizes scientific evidence and provides policy recommendations. The framework for considering additions to the screening panel has been detailed in a 2021 report [902]. In consideration of professional stakeholder involvement in NBS, a 2017 project evaluated the inter- and intra-personal moral issues that may be encountered by health professionals in NBS, and the support required as they navigate complex NBS system logistics [903]. Multi-disciplinary stakeholder involvement in the NBS system is critical. A 2021 report looked at the perspectives of parents, policy makers and professionals regarding NBS expansion [904]. Studies have recently been reported concerning parents’ views on accepting, declining, and expanding NBS [905] and their perspectives on retention and secondary use of NBS specimens [906]. The Health Council of the Netherlands has specified criteria to assess the long-term harms and benefits of NBS, and in 2021, performed an initial assessment for 11 of the 25 conditions on the NBS panel [907]. In 2022, at the request of the MOH and RIVM CPS, an external consulting service prepared a recently published report on the future of NBS in the Netherlands [908].
  NBS continues to expand and an evaluation framework for adding new conditions was developed in 2015 when the program was considering expansion from 17 to 31 condition. This framework has been suggested as an ongoing model to assist in future expansion efforts [902]. Recently a report sought to define “treatability” as part of the evaluation framework. This process is envisioned as a starting point for better defining each of the criteria used in selecting screening disorders going forward [909]. Several reports contribute to documenting the process for adding SCID to the NBS panel [910], including preliminary SCID incidence findings [911], screening assay evaluation [912], screening cost effectiveness [913], reporting (or not) incidental findings [914], various screening strategies and their real life economic evaluation [915], and parents’ perspectives and societal acceptance of SCID screening [916,917]. Interestingly, the Health Council has been formally asked for advice on when “non-treatable” disorders might be screened and a report on their response has been published [918]. In addition to SCID, recent reports about other new conditions have included a data report with a recommendation for the addition of GALK [919], a cost-effectiveness study of NBS for SMA [920], two reports concerning the sex-specific NBS pilot for ALD [921,922], a retrospective study of referrals for primary carnitine deficiency in consideration of moving it from an incidental finding to a member of the screening panel [923], and a retrospective evaluation of the Dutch pre-NBS cohort for PA and MMA (what to aim, expect, and evaluate from NBS) [924].
  PA and MMA were added to the screening panel in 2019, and other recent additions have included GALK (2020), SCID (2021), MPS-I (2021), SMA (2022), and ALD (2023). The program now contains 27 target conditions and two incidental findings (sickle cell trait and primary carnitine deficiency). Evaluation studies ongoing include MPS-I, ALD (5-year study), 3-MCD 3-year study, and the data registry (10-year study) [925]. Recently, there have been several evaluations of different aspects of the NBS program: 11 years of NBS data for MSUD [926]; 11 years of data for CH [927]; 12 years of data for CAH [928]; 15 years of data for SCD [929]; and 10 years for VLCAD [930]. Laboratory-specific studies have also been reported: second-tier 21-deoxycortisol screening for CAH [931]; a 4-step screening protocol for CF (IRT-PAP-DNA-EGA) [932]; a reevaluation of NBS for TYR-I using succinyl acetone in view of a late-diagnosed case (false negative) [933]; and the use of thyroxine-binding globulin concentrations to improve screening for central CH [934]. The stability of analytes in stored specimens has also been reviewed with particular emphasis on instability of certain acylcarnitines [935]. The TSH concentrations in NBS specimens have been studied as a means of monitoring iodine status [936]. Looking to NBS in the future, NBS for CTX has been considered using both flow-injection and UPLC MS/MS [937], machine learning has been studied as a means of improving the PPV for CH NBS [938], and a 3-step study has been outlined as a means of exploring NGS techniques as a first-tier approach in NBS [939].

- **Norway**—In 2012, the Norwegian NBS program expanded from two conditions to twenty-three and the addition of two more conditions in 2018. A recent report describes the screening results, experiences with second-tier MS/MS methods, DNA testing, use of the Region 4 Stork (R4S)/CLIR post-analytical interpretive tool, and clinical outcomes and follow-up challenges after expansion. To continue program improvements, an appeal is made for coordinated international collaborations [940]. Also in 2018, approval of the use of residual NBS specimens for epidemiological research not related to NBS and the indefinite storage of residual specimens resulted in a critical review of the mechanism for deciding the changes and their extent. A perceived lack of sufficient involvement of ordinary citizens in the decision-making process, the presence of a national personal identity number, and an ambitious national biobanking system (including multiple central health registries) were all identified as contributing to possible “function creep” (serving purposes other than helping newborns) of the NBS program [941].
  Multiple studies concerning the feasibility and value of NBS for vitamin B_12_ deficiency were reported. A 2022 study evaluated the predictive value of NBS algorithms from Austria and Germany (Heidelberg) in detecting infants that were clinically diagnosed later with symptomatic B_12_ deficiency and found that neither could identify most infants diagnosed with symptomatic B_12_ deficiency after the neonatal period. This study also investigated whether being born in a hospital using nitrous oxide (N_2_O) as pain relief in labor may have had an impact on total homocysteine at NBS and found that N_2_O may impact total homocysteine at NBS [942,943]. A more recent study looked at the detection of B_12_ deficiency when second-tier dried bloodspot (DBS) analyses of total homocysteine and methylmalonic acid are included and concluded that NBS for B_12_ deficiency is not straightforward. Additionally, there is a need to address whether screening and treatment of maternal B_12_ deficiency during pregnancy to evaluate its value as a preventive strategy for B_12_ deficiency in both mother and baby [944].
  Other reports have included a review of the first 3 years of screening for CF (and the need to revise the IRT/DNA protocol) and the possibility of NBS for branched chain ketoacid dehydrogenase kinase (BCKDK) deficiency, which is linked to a neurodevelopmental disorder characterized by autism, intellectual disability, and microcephaly [945,946]. A report on a pilot using second-tier NGS with an amplicon based targeted gene panel has been shown to provide a rapid molecular diagnosis (or not) of SCID [947]. A recent report notes the value of Sanger sequencing for monogenic disorders caused by variants in one single gene or in a few genes and presents their Sanger methodology including primer sequences and the genetic test algorithms [948].

- **Poland**—NBS for SMA was approved in 2021 and has undergone staggered implementation across the country with the last provinces completed in 2022 [949]. A discussion of the disease and some of the diagnostic issues was published in 2020 and provides information that is potentially useful as screening policies are developed [950]. The Polish Vaccinology Association published the first recommendations for gene therapy for newborns who received live vaccination against tuberculosis, which might be helpful in other countries where live vaccine against tuberculosis is still used [951]. Looking to the possibility of further program expansion and building on experiences in Germany, where SCID was added in 2019, the first 14 months of a border collaboration to identify newborns with T and/or B immunodeficiency was reported in 2020 [952].
  Several reports of screening activities for other conditions have been reported since 2020. At least three reports related to CF NBS have been published: a study of the clinical characteristics of Polish patients with rare and novel CFTR mutations to determine their pathogenicity [953]; a 10-year review of the impact of NBS on clinical outcomes of pediatric patients in Lodz Voivodeship [954]; and an investigation into the clinical complications in children with late-diagnosed (false-negative) CF (with a reminder that, “*in the presence of clinical symptoms, additional diagnostics must be implemented, in spite of the negative screening results*”.) [955]. At least three reports related to screening for endocrinopathies have been published: a case studies of the value of NBS in detecting CAH absent clinical signs in two female newborns [956]; a review of the challenges of CH diagnosis and treatment in preterm newborns and the value of NBS [957]; and a look at the prevalence of hypothyroxinemia in newborns born before the 32nd week of gestation with a birth weight below 1500 g, with a suggestion to measure serum TSH and FT4 between the third and fifth day of life supplementary to NBS [958]. A report on BIO presented the molecular spectrum (profound and partial forms) of the disease in Polish patients and estimated overall disease prevalence to be 1:66,966 (1:178,577 for profound and 1:107,146 for partial) [959]. A study looking at pain management during the heel stick procedure found that over 60% of newborns felt no pain or discomfort and that the non-pharmacological interventions studied (breastfeeding, oral glucose dosing and non-nutritive sucking) were all effective pain management techniques [960].

- **Portugal**—The NBS program started in 1979 with screening for PKU, and CH was added in 1981. The program expanded to >99% national coverage within 10 years [961]. It is now a voluntary public health program screening for 28 conditions in a single laboratory with specimens collected on the 3rd day of life. A 2024 report reviewed the implementation and results of MS/MS expansion since 2004, including the importance of second tier testing for many of the metabolic conditions [962].
  Results of a 3-year pilot that included CF resulted in a recommended screening algorithm that included IRT/PAP/DNA [963]. CF NBS was added to the Portuguese NBS program in 2019. A report analyzing data from 1 or 5 CF centers and that the European Cystic Fibrosis Society (ECFS) recommends that NBS programs should aim for a minimum PPV of 30%, a minimum sensitivity of 95% and suggested that these criteria should be applied to a national program evaluation [964].
  Portugal has one of the highest incidences of Cbl C/D globally (~1:85,000). A 2017 report compared the genotype/phenotype of patients identified with Cbl C/D before and after implementation of expanded NBS with positive findings [965]. A 2022 report noted that, “*… early detection through NBS may have prevented hematological abnormalities and irreversible neurological damage in infants with acquired vitamin B_12_ deficiency due to maternal vitamin B_12_ deficiency*”, and that, “*Acquired B_12_ deficiency should be ruled out before proceeding in a differential diagnosis of cobalamin metabolism deficits, methylmalonic acidemia, and homocystinuria*” [966].
  A recent review on PKU and lipidomics noted that, “*MS-based lipidomics is a promising approach to evaluate the effect of the diet restrictions on lipid metabolism in PKU patients, monitor their outcome, …, and find possible prognosis biomarkers*” [967]. Another report gave a brief overview of the use of DBSs in lipidomic studies and noted the variables that can affect lipidomic analyses (DBS card matrix, hematocrit, specimen homogeneity, etc.) [968].

- **Romania**—NBS for phenylketonuria is carried out at the national level in five regional centers, must have the mother’s consent after explanation from her physician, and is funded by the MOH within the framework of the National Women and Child Health Programme [969]. A 2021 report summarizes the importance of NBS for early detection and treatment of both metabolism and endocrine conditions and emphasizes the lack of international standardization in NBS operations and makes the case for a standardization organization in Europe [970]. Several reports have focused on CH NBS in Romania in recent years: a report on 8 years of experiences with the MEDILOG registry for monitoring CH screening steps, CH incidence, diagnosed case management, iodine deficiency (through TSH), and more [971]; a review of CH screening in North-East Romania with suggestions for follow-up improvement—speedier diagnosis communication, tighter follow-up schedule, etc. [972]; and a review of CH data looking at the impact of moving the time of specimen collection and cutoff levels in order to improve patient outcomes [973]. A review of the value of NBS for GAL (all types) was published in 2021 updating the incidence, clinical manifestations, diagnosis, therapy, and prognosis [974].
- **Russia**—The NBS program expanded to include 36 new conditions, including SCID and SMA, on 1 January 2023 with screening available in 10 centers across the country [975,976]. NBS is carried out at the expense of budgetary allocations of the Regions of the Russian Federation and is free to the public [977]. A 2017 review of the past, present, and future of pediatrics in Russia helps to better understand national pediatric healthcare issues [978]. In preparation for the expansion, three SCID-related studies were published: two reviews of TREC and KREC screening, including the potential role of NGS in increasing the diagnostic accuracy of PIDS in general [979,980]; and determination of the 95% confidence intervals for TREC and KREC levels for different gestational groups with the suggestion that gestational age is an important factor that affects both TREC and KREC levels in newborns [981]. A recent publication reported on a pilot project for SMA NBS in St. Petersburg, which identified cases and supported the idea of earlier detection and diagnosis through NBS [982]. At least two other studies aimed at improving NBS have been reported since 2020: an assessment showing the effectiveness of CF case detection by screening versus clinical symptoms [983]; and a study showing the necessity of introducing differential diagnostics for tetrahydrobiopterin-deficient types of hyperphenylalaninemia to ensure the correct therapy [984].
- **San Marino**—Newborns in San Marino are screened for the same large number of conditions as newborns in Italy. Their specimens are sent to the Emilia-Romagna regional center laboratory at the Bologna University Hospital [985].
- **Serbia**—Two studies sought to improve the NBS program by reviewing CH case data over a 30-year time span. The first found a nearly 3-fold increase in detected CH in Central Serbia, which was presumably associated with lowered TSH cutoffs. Other factors affecting the increases were unclear and require additional studies [986]. The second study focused on the 30-year data and 14 late-diagnosed cases, finding 71% due to sample collection errors with the remainder as false-negative screening results with the goal of overall program improvement [987]. Recently, a program expansion pilot for SMA began (April 2022) in Narodni Front maternity hospital in Belgrade and was scheduled for national expansion in April 2023. The State Health Insurance Fund (RFZO) covers the costs of diagnostics, treatment, medical aids, and rehabilitation for all detected patients [988].
- **Slovak Republic**—The first ethnic results of the National Extended Newborn Screening (ENS) in Slovakia for the majority and the Roma ethnic populations were reported in 2017 for all 23 conditions. Significant differences in the incidence and prevalence of almost all disorders were found, especially in the IEM group, allowing increased ethnic focus on care, especially for children living in socially disadvantaged areas [989].
- **Slovenia**—A comprehensive review of the earlier years of NBS in Slovenia was published in 2015 [990]. In 2018, building on pilots that experimented with both expanded NBS and NGS confirmatory testing, expanded NBS was introduced in Slovenia, 17 metabolic conditions were added to the Slovenia screening panel, and the NBS program was reorganized and upgraded. Further expansion is expected soon [990,991,992]. A 2021 review gives an overview of the current state of NGS use in NBS, the remaining obstacles to its implementation, and the wider implications of its use in the NBS program [989]. The use of MS/MS has resulted in a study of a higher incidence of VLCAD than expected, which also resulted in one of the first reports of 4 novel variants of the acyl-CoA dehydrogenase very long-chain gene in Central-Southeastern Europe [993]. Two studies of phenylalanine detection also have been recently reported: a parallel study of MS/MS versus fluorometry showed lower analytical results with MS/MS but no missed cases [994]; and a review of fluorometric cutoff values as a protocol review/update showed that a slight upward adjustment (120 µmol/L 160 µmol/L) would make the screen more precise, resulting in fewer patient recalls (false-positive screens) [995].
- **Spain**—NBS in Spain is complex and is offered as a public health program in 15 screening laboratories within the 17 Autonomous Communities. The 2021 comprehensive review of NBS in Spain provides the history since initial implementation in1968 and details of the current screening situation [996]. A contemporary article reviews the implementation of national legislation specifying conditions that must be a part screening nationally, which begins removing inequalities in access inequalities. Forums coordinated by the MOH with the participation of those responsible for public health from the Autonomous Communities and scientific societies have been fundamental in serving as an example of the convergence of research and science for the benefit of a basic public health program [997]. Two other reports in 2021 draw attention to NBS management issues: one reflects on the revolution envisioned for NBS with the emergence of genomic technology and the continuing need for international harmonization of NBS programs; and the other points to NBS as a healthcare model of precision medicine [998,999]. A three-part series of articles reviewed the history of Spanish NBS focusing on ethical [1000], legal [1001], and social [1002] aspects of the NBS system.
  As a result of the variations in NBS across the country, several reports since 2020 have reviewed the history and status of various regional screening successes: 50 years of NBS in Catalonia [1003]; 20 years of NBS in Galicia [1004]; 10 years of NBS in Western Andalusia [1005]; 15 years of NBS for SCD in Madrid [1006]; 3 years of NBS for SCD in Western Andalusia [1007]; screening for propionic, methylmalonic acidemia and vitamin B_12_ deficiency in Madrid [1008]; 3 years’ experience in NBS for SCID in Catalonia [1009,1010]; 8 years’ experience with expanded NBS in Madrid [1011]; evaluation of positive cases from NBS in Madrid [1012]; CAH screening in Madrid [1013]; CH cutoff values in the Balearic Islands [1014]; the role of public health in NBS success in the Basque Country [1015]; and, an improved specimen transport system in Catalonia [1016]. Reports have also addressed issues related to second-tier testing to detect cobalamin (vitamin B_12_)-related disorders, both genetic and acquired [1017], differences in IRT levels between the different groups of newborns with a positive screen (healthy, healthy carriers, affected by CF or CFSPID) [1018], and the potential usefulness of NBS in detecting cystinuria [1019].
  Looking to the future, a 2021 review highlights the relevance of NBS for CH reviewing the status nationally and globally and the challenges and future prospects [1020], and a 2023 report notes the usefulness of elevated TSH NBS in detecting other conditions as part of the differential diagnosis of CH [1021]. The economic value of NBS also has been reviewed along with a look at progress in the automated economic assessment of NBS [1022,1023]. In addition to a review of the value of genetic analyses for NBS confirmation [1024], current screening tools, and future screening perspectives [1025], a recent report describes the use of LC-MS/MS as a second-tier panel of 31 metabolites increasing the efficiency of NBS by reducing false-positive and false-negative results, second sample requests, and the time to diagnosis [1026]. A two-part report from the Ethics Commission of the Spanish Society for Human Genetics summarizes and addresses NBS and NGS issues moving forward [1027,1028].

- **Sweden**—The NBS program began in 1965 and now has one central screening laboratory, two IEM diagnostic centers, three treatment centers (beginning in 2024), two neuromuscular treatment centers, and three SCID diagnostic/treatment centers. CH and CAH are usually handled in pediatric clinics and local hospitals [1029]. Since 1975, all residual bloodspots remaining after NBS have been stored in a biobank for QA and research in accordance with Swedish biobank law. Expansion to include 19 additional disorders by MS/MS occurred in 2010, a national IEM registry was initiated in 2013, and NBS for SCID was added in 2019. Two reports summarize SCID findings: one defines the 2-year pilot study; and one summarizes the first year’s screening results [1030,1031]. In the case of expanded NBS, implementation of second-tier NBS analyses for MMA, PA, IVA, HCY, and the use of collaborative laboratory integrated reports (CLIR) has helped to improve the overall PPV to around 60% [1032]. Additionally, a 2022 report on NBS for VLCAD suggests that a clinical severity score, compiled using treatment interventions and clinical symptoms, may correlate with the NBS CLIR score and residual enzyme activity as an aid in defining treatment [1033].

Emphasizing the importance of public investments in early diagnosis and treatment, a 2023 report evaluated the long-term costs and health effects of the Swedish NBS program for classic PKU alone and in combination with CH compared with no screening [1034]. Also emphasizing the value of NBS, two reports in 2020 addressed CAH screening: one addressed the success of NBS for CAH through close laboratory/follow-up collaborations and the other provided updated data on case detections and outcome [1035,1036]. A 2023 report reviewed CH cases from 1980–2013 (particularly focused on late-diagnosed cases) and detailed the improved case findings as the TSH screening threshold was lowered through the years [1037]. Emphasizing the importance of public investments in early diagnosis and treatment, a somewhat controversial 2023 report evaluated the long-term costs and health effects of the Swedish NBS program for classic PKU alone and in combination with CH compared with no screening [128,129].

Continuing to expand NBS, SMA was added to the screening panel on 30 August 2023 by the National Board of Health and Welfare policy development [1038]. Regarding other conditions, a study developed G6PD incidence data and found that NBS was probably not warranted [1039], two studies looked at the possibility of analyzing DBSs for phosphatidylethanol, an alcohol biomarker [1038,1040], and another study looked at the characteristics and outcomes of patients with formiminoglutamicc aciduria detected through NBS [1041]. In the long term, NBS may be significantly impacted by a new project, Screen4Care, which draws on a collaboration of 21 academic partners, 9 industrial project partners, and 4 small- and medium-sized enterprises to initiate a multi-pronged strategy to shorten the time to diagnosis and treatment for patients with rare diseases through genetics and artificial intelligence [1042].

- **Switzerland**—NBS for PKU has existed in Switzerland since 1965 and was one of the first programs in Europe to add CH screening (mid 1970s). Specimens are collected on day 4 of life (written informed consent) and submitted to a centralized laboratory, which returns results within 1–3 days [1043]. A NBS TREC/KREC pilot for SCID was introduced in January 2019 to look for both T and B cell deficiencies [1044,1045]. In addition to outlining the NBS screening procedure, a 2020 report gave recommendations for diagnostic evaluations and precautionary measures against infection in children with abnormal screening test results. A recent 2023 Swiss publication reviews Primary Immune Deficiency Treatment Consortium data showing that more than half of NBS identified SCID patients developed at least one infection prior to hematopoietic stem cell transplantation and suggests access to monoclonal antibodies for RSV prevention for all SCID patients in countries with SCID NBS [1046].
  Other recent NBS activities have focused on laboratory testing: a report reviewing increased laboratory efficiency using a commercial robotic DBS extraction device in combination with LC-MS/MS [1047], a study to define reference intervals for total thyroxine (tT_4_) in NBS specimens with emphasis on premature and term-born infants [1048], and a clinical comparison and evaluation of two commercial testing platforms for NBS for TSH in DBS [1049]. A recent Swiss study sought to describe the IRT levels in healthy newborns in their first year of life and by gestational age, and to compare IRT values at two time points in healthy newborns and newborns with CF [1050]. While not a part of the Swiss NBS program, 30 years of data from TOXO NBS in Northwestern Switzerland were reported to inform the debate about the need for NBS for TOXO in Europe [1051]. Looking to the future, a commercial laboratory provides their “Baby Health Check” program as a supplemental screen providing testing for 15 additional conditions using “Screening of the Next Generation” [1052].

- **Turkey (Türkiye)**—Building on the 1983 PKU pilot program that showed a high incidence of PKU in Turkey (recently confirmed in a systematic review and meta-analysis [1053]) NBS was officially initiated by the MOH in 1986 and became the “National Phenylketonuria Screening Program” in 1994. Screening expansion (national) included BIO, CH, CF, and CAH in 2006, 2008, 2015, and 2021, respectively. There are 2 screening laboratories and specimens are collected at 48–72 h with results available in 1–2 days. Pilot testing with MS/MS began in 2002 but it is still not part of the national NBS program [1054]. A 2020 report of MS/MS NBS experiences in a multi-institutional hospital system across the country evaluated the distribution of 26 different acylcarnitine and amino acid disorders, established screening cutoff levels, confirmed the presence of several screened disorders in the population, and in view of the high rate of consanguineous marriages in Turkey, suggested expansion of the national NBS panel [1055].
  A 2022 mini-review of official NBS policies (or strategies) defining the number of conditions screened in 12 selected countries (including Turkey) confirmed that the Turkish NBS program should be expanded based on epidemiologic information, screening program infrastructure, cost estimates, and pilot screening data [1056]. Early in 2022, the MOH announced the addition of SMA to the Turkish screening panel and it was added in September 2022 [1057,1058]. Also, near the end of 2022, a Turkish molecular diagnostics startup launched an exome-sequencing-based newborn DNA screening test, which has the potential to become a suite of tests for various genetic diseases [1059]. Other recent reports concerning Turkish NBS have been published: a study of lymphopenia screening from cord blood demonstrating a cheaper, less invasive alternative to TREC analyses for SCID [1060]; a report of the status of genetic diagnostic laboratories and the frequency of genetic variants associated with CF detected through the Turkish NBS program [1061]; a 5-year data review of CF screening in a tertiary care center showing low sensitivity and PPV and a need for a national study to establish new cutoff values [1062]; a 6-year data review of the CF NBS protocol also showing low PPV in 3 tertiary care facilities with a similar appeal for re-evaluation of the screening protocol [1063]; and a report showing that mothers in Turkey are not sufficiently knowledgeable about NBS and recommending that education using mass media, educational environments (pregnancy schools), and screening test centers (including increasing awareness of this deficiency among midwives and nurses) [1064]. Most recently, a report on case reviews noted that ENBS can also play a pivotal role in identifying conditions in mothers of newborns identified by NBS [1065].

- **Ukraine**—Despite the challenges of war currently being faced in Ukraine, efforts to expand the NBS program are underway, beginning with a virtual international conference in 2022. Building on a screening program that includes PKU (2001), CH (2005), and CAH and CF (2012–2014 and 2017), a presidential declaration in 2021 listed 21 conditions for inclusion in the national NBS program as funding allows. There are currently 12 regional/inter-regional screening laboratories with four more for the new conditions requiring MS/MS [1066]. A description of the healthcare system in Ukraine published in 2020 helps to understand responsibilities within the system and how it affects NBS [1067]. Preparing for expanded NBS, a pilot study of the TREC/KREC screening procedure for SCID and other PIDs was recently published [1068].
- **United Kingdom (England, Northern Ireland, Scotland, Wales)**—The NBS program includes specimen collection at day 5 of life (birth is day 0) at home by a midwife or health visitor with results returned within 6 weeks. Information relating to the nine conditions included in the screening panel (PKU, MCAD, IVA, HCY, MSUD, GA-I, CH, CF and SCD) and other basic program information are included online with similar information available in the four health departments (England, Scotland, Wales, and Northern Ireland) [1069]. Various expert committees and advisory groups make NBS recommendations, including the UK National Screening Committee (UK-NSC), among others, working to ensure that NBS in the UK is evidence based, ethically sound, cost effective, and aligned with national public health goals. A well-defined process for both population and targeted screening exists. Examples of recent costing studies informing the process of the addition of screening conditions include studies on the economic impact of adding ALD [1070], the cost effectiveness of adding SCID [1071], and re-examination of the cost effectiveness adding five conditions (GA-I, HCY, IVA, LCHAD, and MSUD) [1072]. A costing analysis of teleconsultations as a means of communicating positive screening results was recently reported [1073] along with an in-depth description of the communication project and its collaborators (laboratory staff, clinicians communicating positive screening results, and parents) [1074]. Since 2020, several reports on result communications have been published [1075,1076,1077,1078,1079,1080,1081].
  NBS for CF has been part of the UK screening panel since 2007 and the use of NGS as part of the screening process for this and other conditions has begun. A few CF-related issues have been recently reported: the impact of NBS on outcomes and social inequalities [1082]; the psychological impact of NBS for CF, particularly when awaiting confirmatory results [1083]; parents’ experiences following inconclusive results from CF NBS [1084]; and the quality of the specimen being tested [1085]. A study of parents’ views on the use of NGS for improving the sensitivity and/or specificity of the CF NBS protocol was reported in 2022 [1086]. Discussions on the broader use of NGS as part of NBS, including relevant ethical issues, have also been published: a review of the opportunities and challenges for NBS using genomic sequencing [1087]; a report on the value of the use of ethics, engagement and co-design in developing a national newborn genomes program [1088]; a report of a research project within the National Health Service (NHS) to perform whole-genome sequencing of up to 200,000 newborn babies [189]; a review of the lessons learned from ongoing NBS programs looking at the psychological and ethical challenges of introducing WGS into NBS [1089]; and a 2023 ethics study using newborn genome screening as an example [1090].
  The UK-NSC has recently reviewed and approved the addition of TYR-I to the NBS screening panel for all UK departments [1091] and is currently considering the addition of SCID. To this end, at least two reports have been published: a review of SCID considering the NBS approach [1092]; and a study design for evaluating the outcomes and care costs for a group of babies identified by NBS compared with a group outside of the screening cohort [1093]. Screening for SMA is being considered by the UK-NSC and the results of a 5000-specimen pilot have been reported [1094], and another independent research study has been launched [1095]. Studies of disease-modifying therapies are underway in the UK [1096]. Already there has been a call to add SMA to NBS in Scotland [1097]. Other published NBS activities include a review of the feasibility and ethics of using data from archived Scottish NBS specimens [1098], a report on the importance NBS and early diagnosis in metachromatic leukodystrophy based on a caregiver survey in the UK and the Republic of Ireland [851], a pre-pilot MLD NBS feasibility study in Manchester UK using a two-tiered screening test algorithm that added to the international evidence recommending NBS new for MLD [1099], and a discussion of whether there is value to assessing maternal alcohol consumption in pregnancy by measuring phosphatidylethanol on day 5 DBS cards [1100]. A recent report on the efficacy of detecting GALT incidentally from the current NBS process notes that cases of GALT are being detected through the process and additional cases would be detected if screening was moved closer to birth [1101].

#### 3.3.3. Tabular NBS Data for Europe

In 2021, there were 2 published reports containing comprehensive data on European NBS activities [668,669] and another in 2022 from a manufacturer’s perspective [1102]. A report on NBS for SCD in Europe also exists [697] along with various national updates, on this and other conditions. We have updated the European data on screening tests with the best available data at the time of this writing (Table 7). Because NBS continues to move forward as science, technology, and resources permit, so too do the data. For that reason, the reader is cautioned to verify any data seen here before it is used elsewhere. In the future, the ISNS is planning to maintain reliable information on its website (https://www.isns-neoscreening.org). In addition to population and birth data, we have provided start dates for NBS programs based on our 2015 report and new information obtained from our current literature reviews. We have included information on the number of screening laboratories and time of specimen collection from the 2021 European update to succinctly describe European NBS programs [668]. These data are important in understanding some of the nuances that may differ between European NBS programs and those in other countries or regions. For example, most European programs collect NBS specimens 48 h after birth or longer in contrast to U.S. states where specimen collection is at or after 24 h of age (12 h in California). Also, there are several European countries where more than a single screening laboratory exists, which means that the volume of specimens tested may be quite low in some cases. Because some laboratories within a multi-laboratory country may not perform testing for all conditions, specimens may require splitting to facilitate testing at more than one laboratory. As NBS laboratory testing becomes more sophisticated, potentially requiring more specialized testing personnel and more expensive equipment, this splitting approach to laboratory testing is gaining popularity both in Europe and elsewhere (e.g., USA).

### 3.4. Latin America (LATAM) and the Caribbean

**Figure 5:**
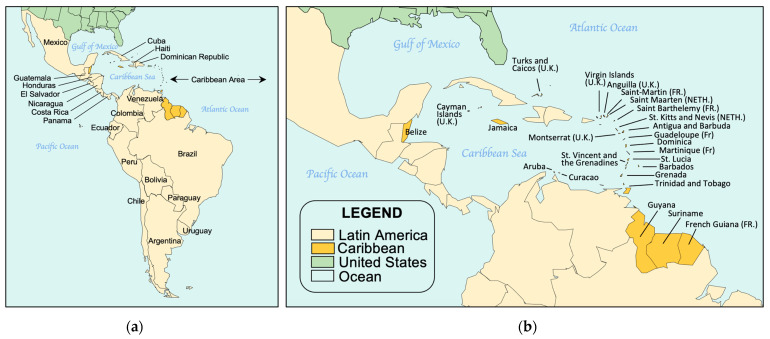
Map of Latin America (**a**) and Caribbean region (**b**).

Latin America (LATAM) consists of the nations in the Americas and the Caribbean whose residents predominantly speak Spanish or Portuguese, both languages having descended from Latin. In our previous reports, we have focused on 20 countries in LATAM based on the definition of the Economic Commission for Latin America and the Caribbean (ECLAC), which includes three countries in the Caribbean (Cuba, Dominican Republic, Haití) [16,30]. In this report, we expand the definition of the region to include both the 20 LATAM countries and 25 additional Caribbean countries using the ECLAC listing for LATAM and the Caribbean [1103,1104]. The listings include two countries, Puerto Rico and the U.S. Virgin Islands, which are not included here but are addressed in Section 3.1. To facilitate comparisons with our 2015 report, we continue to include data from Cuba, Dominican Republic, and Haití with the data from LATAM, despite their location in the Caribbean. The Caribbean area is made up of over 700 islands and the surrounding coastal areas of North, Central, and South America and jurisdictions within the area are complex as illustrated in Figure 5a,b.

The population of the LATAM and the Caribbean region is about 650 million, the land area is six times larger than India, and there are about 10 million births annually. There are six different languages that predominate: (1) Spanish (18 countries)—Argentina, Bolivia, Chile, Dominican Republic, Ecuador, El Salvador, Nicaragua, Panama, Paraguay, Colombia, Costa Rica, Cuba, Guatemala, Honduras, Mexico, Peru, Uruguay, Venezuela; (2) English (18 countries)—Anguilla, Antigua and Barbuda, Bahamas, Barbados, Belize, Bermuda, British Virgin Islands, Cayman Islands, Dominica, Grenada, Guyana, Jamaica, Montserrat, Saint Kitts and Nevis, Saint Lucia, Saint Vincent and the Grenadines, Turks and Caicos Islands, Trinidad and Tobago; (3) French (4 countries)—French Guiana, Guadeloupe, Haití, Martinique; (4) Dutch (2 countries)—Sint Maarten, Suriname; (5) Papiamento, a Portuguese-based Creole language, (2 countries)—Aruba, Curaçao; and (6) Portuguese (1 country)—Brazil.

The region is diverse in geography, demography, and healthcare. Healthcare diversity extends to NBS with some countries having well-established programs with pilots dating to the 1970s, and some just beginning. The number of screened conditions, the level of involvement of the various health ministries, and the extent to which NBS is available to the public also vary. The complexities of jurisdictions in the Caribbean Sea include both dependencies and sovereign nations. Some of these continue to have relationships steeped in the history of their European conquerors.

Since the first LATAM report in 2007 [30] and our 2015 update [15], there have been two recent reviews of NBS in LATAM [1105,1106]. A 2019 report on NBS for SCD in the Caribbean [1107] provides a comprehensive discussion of the Caribbean and its history, including the fact that the Caribbean ranks second to sub-Saharan Africa in SCD prevalence.

Currently, there are five levels of activity that describe the status of NBS across LATAM: (1) well-established expanding programs (Cuba, Costa Rica, Uruguay); (2) older established program, growing nationally (Argentina, Mexico, Venezuela, Chile, Colombia, Brazil, Paraguay, Nicaragua, Panama, El Salvador; (3) young established regional or national programs beginning to grow (Ecuador, Peru, Bolivia); (4) some screening, working towards sustainability (Guatemala, Honduras, Dominican Republic); and (5) little or no screening activity (Haití) [1105]. In the Caribbean, NBS is generally sparse but there has been growing success in establishing NBS programs for HGB [1107]. Also, some island maternities have developed efficient systems for submitting specimens to screening laboratories in more economically developed countries that provide expanded testing panels.

#### 3.4.1. Regional NBS Activities Involving Multi-National Collaborations

##### Regional NBS Activities Involving Multi-National Collaborations in LATAM 

While there have been differing amounts of NBS activity within LATAM countries since our 2015 report, there have also been activities more broadly focused on the region. For example, several NBS activities that are focused on the NBS laboratory have been described. Primary is the development of a quality assurance (QA) and proficiency testing (PT) program for LATAM screening laboratories, the “External Quality Assurance Scheme for Neonatal Screening” (PEEC-PN—after its Spanish name, “Programa de Evaluación Externa de Calidad—Pesquisa Neonatal*”*), located at the Fundación Bioquímica Argentina [1108]. Since 2000, participation in the PEEC-PN has steadily increased, improving the quality and harmonization of NBS screening test results across the region. As of June 2023, there were 152 laboratories from 13 countries enrolled: Argentina (42); Bolivia (3); Brazil (13); Chile (2); Colombia (62); Cuba (1); El Salvador (2); Guatemala (2); Mexico (7); Panama (2); Paraguay (5); Peru (10); and Uruguay (1). Of the 152 laboratories, 147 participated in proficiency testing for thyrotropin (TSH), 94 for phenylalanine (Phe), 90 for immunoreactive trypsinogen (IRT), and 81 for total galactose (TGal) [1109]. Areas of difficulty include laboratory turnover (laboratories that register and begin participating only to withdraw after a few surveys), participants’ understanding of the analytical units used by manufacturers, use of correct units in reporting results, and timely participation [1110].

A study on the long-term stability of leftover NBS screening specimens from diagnosed patients in 2021 sought to determine their usefulness for retesting. The specimens had been stored up to 23 years in suboptimal and uncontrolled conditions of temperature and humidity and were analyzed for Phe, TSH, IRT, TGal, and 17-hydroxyprogesterone (17OHP). The findings were summarized for the benefit of laboratories in resource poor countries who might meet similar specimen storage challenges [1111].

Since our 2015 report, there has also been significant regional activity focused on PKU, beginning with the development of consensus NBS guidelines for PKU [1112]. These guidelines included recommendations on the type of specimen, the age at collection, the testing methodology, and the analytical cutoff values. A 2023 report based on a multicenter survey described adherence to these guidelines among PKU patients in Argentina, Brazil, and Mexico [1113]. In comparison, a 2021 report that described the current PKU diagnostic and dietary management practices in 13 LATAM countries, half of which had medical foods fully subsidized by the government (although special low-protein foods were not available in most countries) [1114]. The primary treatment obstacles were low purchasing power, limited/insufficient availability of low-protein foods, poor treatment compliance, and lack of technical resources to manage the diet. Looking regionally at PKU case findings, a 2022 report showed a high clinical burden with many unmet neuropsychological needs and socioeconomic challenges. Patient compliance to dietary treatment was poor. More studies of PKU patients in LATAM were considered necessary to further elucidate the full spectrum of disease complications [1115].

Other NBS conditions have also been studied regionally. A 2015 comprehensive review of SCD in among Latinos in the U.S. highlighted the substantial case numbers and population distribution of SCD and sickle trait in LATAM and the lack of inclusion of HGB screening in many LATAM NBS programs based on their population distribution of persons of African heritage [1116]. A 2016 short communication reviewed the lack of NBS for CF in many LATAM programs and the need for enhanced understanding and appreciation for the disease. Emphasis was given not only to the need for more CF screening but also the need for appropriate health policies that allow for inclusion of CF NBS, harmonization of CF registries; access to specialized CF healthcare practitioners, transition to adult clinics, and treatment regimens [1117].

Increased interest in NBS for SCID in some countries in the region encouraged the development of the first document attempting to standardize treatment for patients with SCID [1118,1119]. Although consensus was based on the perspectives of LATAM clinicians, the guidelines also were intended for use in other parts of the world. Looking towards LSDs on expanded screening panels in the future, a 2020 report reviewed pilot studies for MPS in LATAM using different methodologies: MS/MS, molecular analyses, digital microfluidics, and fluorimetry [1120], and a more recent report described a NBS pilot study for six LSDs [1121] (see further studies in the Brazilian national report that follows).

As in other more economically developed regions, there has been an ongoing concern in LATAM about equity, particularly as NBS expands. While newborns, their families, and society all benefit from a successful screening program, the reality is that these benefits are not realized globally and uniformly since NBS programs must exist within different economic and political environments. While these inequalities seem intuitively obvious, their documentation is sometimes necessary to demonstrate progress moving forward. At least two LATAM regional studies have focused on this issue. One estimated maternal–child health coverage gaps looking at absolute and relative inequality across LATAM. This study demonstrated substantial inequalities between socioeconomic groups and noted the health policies, programs, and practices that are required to promote equity [1122]. The other looked more directly at genomic sequencing as a part of expanded NBS and reviewed the academic arguments regarding social justice and ethics. This report suggests, “*working towards a world where all countries regardless of economic status can provide basic NBS with access to therapeutics upon diagnosis prior to engaging in further diagnostic advancements which perpetuates health inequities globally*” [1123]. Interestingly, a recent article from the U.S. noted that, “*Investigating the historical origins of a well-accepted health program across a region, such as newborn screening in Latin America, has the potential to reveal the role of historically-specific drivers in shaping national health policy*” [1124].

While we are focused in this report on screening for conditions using blood, it is also important that other types of screening occur that may not use blood but may be linked to the blood screening program’s infrastructure through tracking/follow-up activities, data collection, and/or administration. In this regard, screening for CCHD and retinopathy of prematurity have both received growing interest in LATAM and their progress has been recently reported [1125,1126]. And, while the most recent regional report on EHDI was published in 2011 [1127], updated progress reports from various countries exist and are occasionally noted in the brief national reports that follow. Reference is given here to a 2022 report on the value of EHDI and the global inequities that still exist [1128].

##### Regional NBS Activities Involving Multi-National Collaborations in the Caribbean Sea Area 

In the Caribbean, as in LATAM, there are NBS projects aimed at impacting more than a single country within the region. As an example, the Caribbean Network of Researchers on Sickle Cell Disease and Thalassemia (CAREST) was formed in 2012 to advance the SCD agenda in the Caribbean [1129]. Among other goals, CAREST sought to bridge language, cultural, economic, and geographic boundaries to regionally promote and facilitate NBS for HGB. CAREST offered subsidized screening for a 2-year period to establish a basic NBS infrastructure and to obtain the local prevalence of SCD for healthcare planning. Responses varied and, in some cases, resulted in external laboratory partnerships: Guadeloupe partnered with Tobago (started 2008) and Grenada (2014–2015) and Jamaica partnered with St. Lucia (2015–2017). Other single island/country pilots/programs also exist(ed), namely French Guiana (Lille, France—external laboratory), Jamaica (local laboratory), and Martinique (local laboratory), often providing support for other nearby islands/countries [1107].

Health policy implementation was the subject of another regional project across both LATAM and the Caribbean investigating “*the challenges that impede the uptake of evidence to enhance health policy implementation and the coverage, quality, efficiency, and equity of health systems*”. The Alliance for Health Policy and Systems Research, an international partnership hosted by WHO, developed an innovative research model led directly by decision makers and embedded in real-world health policies and programs. This initiative, Improving Programme Implementation through Embedded Research (iPIER), was tested in 10 settings in LATAM and the Caribbean. One of these was in St. Lucia, where a NBS project was implemented with the intent to modernize NBS by overcoming the barriers to using a heel prick specimen rather than cord blood (see country discussion for further information) [1130].

Part of the political complexity of the Caribbean involves six islands that are part of the Kingdom of the Netherlands—Aruba, Curaçao, and St. Maarten, Bonaire, St. Eustatius, and Saba (the last three known as BES-islands). The first three are “constituent countries” of the Kingdom of the Netherlands, while the BES-islands are “special municipalities”. In the BES-islands, the Dutch Minister for Health, Welfare, and Sport introduced NBS in 2013, coordinated by the local Public Health Services under the direction of the Dutch National Institute for Public Health and the Environment. NBS started in Bonaire in January and St. Eustatius and Saba in October of 2015. NBS DBSs are sent to the Netherlands once weekly and screened for disorders on the Netherlands’ screening panel. Screening coverage exceeds 90% [1131]. NBS activities differ in the other three Dutch countries (see individual country discussions in Section 3.4.2).

#### 3.4.2. NBS Activities with a Country Focus

##### NBS Activities with a Country Focus in LATAM 

The following brief country updates document recent NBS activities ongoing within both LATAM and Caribbean countries. These updates are designed to be brief reviews from published reports, meeting presentations, personal contacts, and other documented newsworthy activities. These reviews are intentionally brief, reflecting primarily recent activities and are not intended to provide detailed histories of NBS within the country. In some instances, historical references may be included to clarify the context of the activity described. We were unable to obtain information on recent activities in some Caribbean countries, namely the British Virgin Islands, Montserrat, St. Kitts, and Nevis and Suriname.

- **Argentina**—The progression of genetics and NBS in Argentina has recently been reviewed [1132,1133,1134]. NBS was formally established by a law requiring PKU screening in 1986. Laws adding screening for CH (1990), CF (1994), and CAH, BIO, and GAL (2007) have established the framework for NBS implementation. Currently, NBS is conducted through 20 regional programs, one in the Buenos Aires Province (1995), one in the Autonomous City of Buenos Aires (2000), and eighteen other provincial programs organized under the umbrella of the Strengthening National NBS Program of the MOH (2006) with a general coverage of the public sector exceeding 98% [1135,1136,1137]. NBS for MSUD also is included in the provinces of Buenos Aires and Mendoza using a locally developed enzymatic analysis. NBS using MS/MS has been conducted for PKU, MSUD, and MCAD since 2014 in the Autonomous City of Buenos Aires [1134]. Looking to improve the NBS program for CF, researchers have compared the current IRT/IRT method for CF screening to the IRT/PAP approach and found improved sensitivity that should decrease the requisite follow-up actions [1138].
- **Bolivia**—While there is a 2006 MOH resolution establishing NBS for CH, there is not yet a national program. Instead, screening, including for other conditions such as PKU, CF, and CAH, is progressively occurring in individual hospitals from the nine Bolivian departments presenting diverse percentages of coverage with a national mean for CH around 30% [1139,1140]. In 2016, a new MOH resolution expanded the NBS panel by adding CAH, PKU and CF. A recent report from a regional initiative in La Paz describes the results corresponding to the period 2017–2023, in which 103,220 newborns were tested for CH, PKU, CF, and CAH [1141].
- **Brazil**—Since 2001, the Brazilian MOH has coordinated the National Neonatal Screening Program, which covers all of the 26 states and the Federal District through 30 reference centers reaching over 82.5% of all newborns [1142]. Information about features of the national NBS program, centers involved in specimen collection, program quality indicators, screened disorders, and the pertinent legislations are available from the MOH [1143]. The 2021 Brazilian legislation is particularly important because it is the first in LATAM to include mandatory NBS by MSMS for aminoacidopathies, urea cycle disorders, FAO disorders, LSDs, and molecular testing for SMA and SCID. A progressive implementation plan was also included [1144]. In recent years, NBS in the Federal District (Brasilia) has included an extensive screening panel including SCID, SMA, and LSDs, NBS in Minas Gerais state has included some MS/MS conditions in addition to the basic six-test panel, and NBS in the São Paulo metropolitan area has included MS/MS and SCID screening.
  Recent studies have reviewed the progress of screening for various conditions, patient outcomes, and related topics: cost effectiveness, screening equity, and program expansion. Program progress studies have focused on CAH [1143,1145], HGB [1146,1147,1148], and CF [1149,1150,1151]. Outcome studies have targeted patients with CH [1152,1153,1154], PKU [1155], GAL [1156], MSUD [1157], and BIO [1158,1159]. The cost effectiveness of NBS for CAH has been reviewed along with inequities in access to NBS itself [1160,1161]. A need for toxoplasmosis NBS in Brazil and elsewhere has also been questioned [1162]. The opportunities for expanded NBS have included studies of SCID [1163,1164,1165], LSDs [1121,1166,1167,1168], SMA [1169], and Prader–Willi and Angelman syndromes [1170]. NHS also has expanded as equipment and speech therapists have become available so that over 65% of newborns now receive hearing screening. Reports on the extent and outcome of hearing screening have been published, including a look at the possibility of NBS for cCMV in the future [1171,1172,1173,1174].

- **Chile**—NBS in Chile dates to a pilot program at the National Institute for Nutrition and Food Technology (INTA) in 1989, which was adopted by the MOH in 1992 and expanded step-wise until it covered the full population in 1998 becoming a possible model for other LATAM NBS programs [1123]. Currently, the NBS program is beginning expansion in four phases to include 26 conditions after a pilot implemented at the INTA in 2017 [403,1175]. In addition to research regarding inclusion of other screening conditions like SCID [1118,1176] and cCMV (at least in high risk groups) [1177], others have focused on outcome studies for CH [1178,1179], PKU [1180,1181], and MSUD [1182].
- **Colombia**—A 2019 law formalized an expansion of the national NBS program from CH to seven conditions and included EHDI and vision screening [1182,1183,1184,1185]. Efforts to continue expanding DBS screening have included developing MS/MS cutoff values and reviewing experiences and policy issues with MPS and other LSDs [1186,1187,1188,1189]. Continued interest in CCHD screening has included cost-effectiveness studies as well as a 14-site collaboration with the Nicklaus Children’s Hospital (Miami, Florida USA) [1190,1191]. Despite work in pursuit of a national NBS program in the past 2 years, NBS is currently only available for CH through various clinical laboratories mostly using cord blood, with newborn coverage exceeding 80%.
- **Costa Rica**—NBS has continued in Costa Rica since its first pilots in the early 1980s and its history has been detailed elsewhere [1192]. Most recent changes have increased the number of screened disorders to 29 [1193]. A recent evaluation of 3 years of screened newborns, CH referrals, and CH diagnoses confirmed the validity of the CH screening algorithm [1194]. The first mutation study of CAH patients from NBS identified an overabundance of cases with c.292 + 5G > A suggesting a possible founder effect [1195]. NBS expansion to include Wilson’s disease is also of interest because of the highest incidence globally (~1:20,000) [1196]. Wilson’s disease is a “National Interest Disease”, which ensures treatment and management of patients in the Costa Rican Social Security National Health System. Researchers have recently reported genotype and phenotype data on Wilson’s disease in Costa Rican pediatric patients [1197]. Finally, a pilot in 20 collection facilities (of over 1000) has also been implemented to improve and standardize the transport of specimens from collection facilities to the central screening laboratory [1198]. It is important to note that Costa Rica has the largest NBS panel in the region and was the first country to implement expanded NBS by MS/MS at a national level (2004) [1105,1199].
- **Cuba**—While Cuba had one of the first screening programs in LATAM, program expansion has been relatively slow [1200]. Products and equipment for NBS are generally produced locally and NBS testing is conducted in a decentralized way through more than 175 laboratories. The Ultra Micro Analytical System (SUMA) is an ELISA-based analytical platform, developed and manufactured by the Cuban Immunoassay Center (CIE), and it has previously been used in many LATAM NBS programs. A 2016 progress report detailed the 30-year history of developing and using SUMA^®^ laboratory technology for several different screening conditions [1201]. A report in 2019 described the 20-year SUMA^®^ external QA program, a program limited exclusively to laboratories using the SUMA^®^ technology and confirmed its relevance in supporting quality NBS [1202]. Recently research reports have included CF test development [1203,1204,1205] and test improvement for CAH [1206] and CH [1207,1208]. As part of general health screening for newborns, attention has recently been given to piloting a new POC NHS procedure. A new screening device developed by the Cuban Neurosciences Center detects both auditory and visual disorders in newborns [1209]. The MOH is currently working on a national plan to introduce hearing screening equipment in health institutions with work underway to add a cardiovascular testing module to the equipment [1210].
- **Dominican Republic**—NBS testing is currently available only on request for private pay patients through hospital and laboratory contracts with a private NBS laboratory in the USA. Efforts to implement a national NBS program have been ongoing. A presidential decree creating the National Newborn Screening and High Risk Program was signed in December 2015, with the goal of screening 95% of all newborns, and a National Newborn Screening Counsel was created for its execution. A national database for metabolic diseases and related conditions was also created [1211]. NBS was to include a screening laboratory in Santo Domingo and evolve in three stages: (1) screening newborns of the three largest maternity facilities in Santo Domingo; (2) screening in centers in the different health regions selected by population density and health risks of the region; and (3) screening nationwide. During its first stage, the NBS program was to include five conditions: HGB, CH, PKU/HPA, GAL, and G6PDD, with specimens collected 48 h after birth. Unfortunately, progress was interrupted due to the pandemic when resources had to be diverted and the NBS decree was repealed due to changes in government authorities. Currently, local professionals interested in NBS are working with lawmakers to enact a comprehensive NBS law. Outside assistance is being provided by the Task Force on Global NBS from the International Federation of Clinical Chemistry and the ISNS [1212]. LATAM members of the Task Force are providing expert advice and assistance in NBS program development and advocacy to local policy makers on NBS cost effectiveness.
- **Ecuador**—NBS has existed as a program under the MOH since 2011. Specimens are collected in MOH hospitals and health centers around the country and tested for CH, CAH, PKU, and GAL at a private screening laboratory. In 2014, a MOH regulation was issued defining procedures for implementation of metabolic NBS [1213]. A recent review of cases detected through NBS identified different rates across disorders within various provinces, suggesting that in addition to reviewing the quality of testing, further investigations should look at rates versus ethnicities, genetics, consanguinity, and cultural beliefs [1214].
- **El Salvador**—A regional NBS program for CH was initiated by the MOH in 2008 and Technical Guidelines were published at the end of 2015 [1215]. In 2018, the coverage for CH reached 73% of newborns in the five regions of the country. Besides metabolic screening (CH, CAH, PKU, and CF), hearing (NHS) and cardiac (CCHD) screening are available at the Salvadoran Social Security Institute [1216]. The MOH and Social Security NBS efforts have expanded with two MS/MS systems now installed in each public center.
- **Guatemala**—Guatemala received technical cooperation support from the IAEA, which included technical visits by one of us (BLT) and contributed to the initiation of CH NBS in Guatemala City as early as 1995 [20,1217]. Expansion since then has been slow. Beginning in 2015, the Institute for Scientific Research and Education on Human Genetic and Metabolic Diseases (INVEGEM) initiated a NBS pilot in national hospitals in Guatemala City and other locations in the country for CH, CAH, PKU, GAL, and CF. The goal of the project is to gather incidence data for use in convincing policy makers to support the program [1218]. A local project in Guatemala City at the two national hospitals is also attempting to better organize NBS and collect similar data to convince policy makers [1219]. Work is also ongoing to determine incidences of other screenable conditions like HGB [1220]. A lack of useful information about NBS has been recognized and is being addressed on an individual hospital basis [1221].
- **Haití**—In 2010, newborns were screened in St Damien Hospital in Port-au-Prince, and the samples were processed at Pordenone Hospital, Italy as part of a project to determine the incidence of SCD. Despite a reported incidence of SCD of 0.58% (twice the U.S.) demonstrating a need for NBS, no national NBS program was continued [1222]. A 2018 report on capacity building for NBS in Haití noted that NBS for SCD is considered the standard of care in some hospitals and research at others [1223]. This report also noted that equipment for screening was a major need that was being addressed as funds become available, usually from external donations. While administration of the hospital-based programs is similar, the laboratory methods differ, including the use of POC testing in some hospitals. A POC validation study was also conducted in collaboration with the Florida, USA NBS program [1224]. Because of the impact of local advocacy groups in other countries, the Haití Sickle Cell Interest Group has been established [1223]. A recent report reviews the progress of the pilot study in demonstrating the feasibility of NBS while noting the difficulties with follow-up (which are much less with POC testing). The project continues with emphasis on “*understanding how to mitigate implementation limitations*” [1225].
- **Honduras**—In September 2016, the National Congress approved the “Mandatory Neonatal Screening Law” to be implemented in Secretariat of Health and Honduran Social Security Institute (IHSS) birthing facilities. The IHSS designed a model for NBS implementation, including a budget forecast and actions to provide personnel, facilities, and screening equipment. A NBS pilot began in 2017, expanding slowly, primarily in IHSS facilities. An operating procedures manual was established by the Secretariat in 2018. Screening includes CH, CAH, PKU, GAL, and CF based on the experiences of others like Cuba and Costa Rica, and the inclusion of HGB has already been suggested. Offering NBS is mandatory, and then parents may then choose whether to accept (informed consent) [1226].
- **Mexico**—The Mexican health system has recently been detailed including the history/process of overcoming some of the institutional, organizational, and resource barriers to establishing NBS [1227,1228]. A review of NBS also exists [1229], along with a review of over 33 years of outcomes in a group of Mexican patients with inborn errors of intermediary metabolism focusing on disparities and unmet needs [1230]. NBS in Mexico was one of the earlier NBS programs in LATAM, beginning in 1973 but paused from 1977 until 1986. In 1988, NBS for CH was formalized by rule [1231]. Currently, the National Center for Gender Equity and Reproductive Health within the MOH is responsible for NBS policies and the screening panel includes CH, CAH, PKU, GALT, CF, and G6PD [1232]. BIO is included in some parts of the health system, e.g., the Mexican Institute of Social Security (IMSS). There are three different healthcare providers in Mexico that serve the population according to employment-based social insurance, uninsured public assistance, and private employer’s insurance [1227,1233]. Different screening approaches occur in the various hospitals, groups of hospitals, and government departments without any unified central coordination [1231,1234,1235,1236]. The Secretary of Health oversees the highest percentage coverage of Mexican newborns [1237,1238].
  Interest in G6PD deficiency, and its addition to the official panel of tests in 2017, has resulted in several recent reports [1239,1240,1241]. Some states also offer expanded NBS by MS/MS and HBG screening. Research on CF and LSDs has also been the subject of recent reports along with screening methods for tyrosinemia and the need to screen for toxoplasmosis [1120,1233,1242,1243,1244,1245]. POC reports on NHS and CCHD have focused on hospital experiences for both [1246,1247,1248]. On 1 June 2021, a reform to article 61 of the General Statute of Health institutionalized CCHD screening and a national committee was initiated to assist in its implementation [1249,1250].

- **Nicaragua**—The NBS program began in 2005 as a collaboration between two local universities, and subsequently, other Spanish institutions. A cord blood NBS program for CH existed until August 2018 when it was discontinued due to the interruption of free testing reagents from the Spanish institutions and the lack of an adequate budget for sustaining the program [1251]. During the first 10 years, nine additional departments joined the screening effort, involving a total of 12 hospitals [1252]. In 2014, a pilot expansion that included CH, CAH, PKU, GAL, and BIO was initiated in the Social Security Institute [1253]. There was interest in HGB screening to the point that a specialty confirmatory laboratory was established through a German academic exchange program [1254]. Aside from blood screening, there has been growing interest in NHS as evidenced by a recent positive cost analysis and an NHS clinic visitation by the U.S. Ambassador [1255,1256].
- **Panama**—A 2007 law created a national NBS program that mandated testing for CH, PKU, GAL, CAH, G6PD, and HBG. By the end of 2014, screening had extended to 11 health regions of the country [1257]. Published HGB incidence results highlighted the importance of HGB as a public health issue [1258]. A recent comprehensive program overview for 2013–2018 noted that for hospitals throughout the 14 health regions, including MOH, Social Security, and private health facilities, the average NBS coverage was 72.3% [1259]. In 2022, a new law was enacted that established NBS as mandatory and extended the panel of screened diseases to CF, NHS, CCHD, and vision [1260]. Screening expansion has now begun, and new MS/MS equipment has been installed.
- **Paraguay**—In Paraguay, NBS began in 1999 as a research pilot project, “Program for the Prevention of Mental Retardation,” to detect CH and PKU. In 2003, with the addition of CF screening and a name change to the “Cystic Fibrosis and Mental Retardation Prevention Program”, the NBS program was formalized by law and officially implemented in 2004. It expanded, with assistance from the Japanese International Cooperation Agency (JICA), including technical advice and support by one of us (GJCB) as part of the JICA “Third Country Experts Program” between 2008–2011. In 2016 the national screening program was created by law, changing its name to the “National Neonatal Detection Program”. It now reaches over 90% of newborns in all 18 health regions and has evolved into a formal public health program [1261]. A more detailed history and progress of NBS in Paraguay has been recently reported [1262]. Since 2018, MS/MS testing has been offered to symptomatic newborns or those with a family history of AA, OA, and FAO [1261].
- **Peru**—Following a 1997 MOH resolution that recognized the need for NBS for CH, a pilot NBS program for CH and CAH was begun by the Social Security Institute of Peru in 2002. The program expanded to include PKU and GAL, and coverage included all Social Security birthing facilities [1263,1264]. In 2012, a law for universal NBS prioritized screening for CH, CAH, PKU, CF, NHS, and congenital cataracts, and in 2019, the MOH issued a Health Technical Rule for NBS for these disorders [1265]. Currently, NBS is available in the main hospitals of the MOH, the Social Security subsystem and the private sector, but it is not completely implemented in some of the public medical centers [1266]. In 2022, a new bill was presented to the Congress contemplating the newborn’s rights to access universal NBS throughout the health system [1267].
- **Uruguay**—NBS began with CH cord blood screening at the clinical laboratory of the maternity facility of the BPS (Banco de Previsión Social—the state-owned Uruguayan social security institute) in 1990 and became a mandate in 1994. Ancillary assistance from the IAEA and integration with the immunization program for logistics, soon led to over 95% newborn coverage [20,1268,1269]. Various health proclamations related to NBS and a challenging transition to heelstick specimens have led now to one of the more comprehensive, universal, free, and equitable screening programs [1268]. Among other activities, a pilot HGB project [1270] and an evaluation of newborns with abnormal screening results, including NHS, have recently been reported [1271], along with a 25-year history of NBS in Uruguay [1272]. Currently, implementation of a NBS pilot for BIO is planned for 2024 and recently the MOH designated BPS as a national resource center for rare diseases.
- **Venezuela**—There has been a NBS law in effect since 1999 for PKU and CH but coverage has been slow. NBS was implemented utilizing a network of 19 regional public health laboratories. In 2013, the national NBS program was created and in 2015, the MOH added GAL, CAH, and BIO to the diseases panel. A 2015 report of 2 years of screening for CH and PKU in Zulia state noted difficulties in getting good demographic data. National NBS coverage reported in 2015 was about 65% [1273], decreasing to about 61.5% in 2017 [1274]. In the past few years, the functioning of the national NBS program has been impacted by the economic problems affecting the country, thus reducing NBS program coverage and efficiency.

##### NBS Activities with a Country Focus in the Caribbean 

- **Anguilla**—A NBS for SCD does not exist in Anguilla. Instead, a program exists to check the sickle status of all pregnant women [1275].
- **Antigua and Barbuda**—The American University of Antigua College of Medicine (AUA) and the CAREST launched a NBS program for SCD in September 2020 at Mount St. John’s Medical Center (MSJMC.) CAREST paid for specimen transport to the laboratory in Guadeloupe. During and after the project, there is/was no cost to parents or the Antiguan government. Screening continues in Antigua with the intent to extend the program to Barbuda and other private medical facilities soon [1276,1277].
- **Aruba**—Now a self-governing constituent country of the Kingdom of the Netherlands, its inhabitants were historically not affected by slave trading and SCD as much as other countries in the area. A 2006 pilot showed a lower prevalence of SCD in Aruba than St. Maarten than Curaçao. Study statistics suggested that 2 patients would be detected every 3 years and questioned the true cost effectiveness of a NBS program for SCD. The possibility of a universal NBS program for SCD housed in a single laboratory strategically located in the Caribbean area was noted as a possibility that would also enable screening for other heritable diseases [1278]. In contrast to the BES-islands, Dutch “special municipalities”, there is no national NBS program, however, newborns admitted to the pediatric department of the Dr. Horacio E. Oduber Hospital are offered NBS for CH [1131].
- **Bahamas**—Expanded NBS is available through a contracted private NBS laboratory in the USA for the birthing hospital and a medical laboratory so that most newborns are screened for the disorders contained in the US RUSP [1279].
- **Barbados**—A 1999 NBS pilot project with 1000 newborns estimated the SCD incidence to be lower than other nearby islands, but a larger confirmation study was never carried out [1280]. In preparation for another pilot in 2014, a database of persons with sickle cell anemia was developed along with a process for documenting clinical manifestations. A pilot comparing SCD prevalence with Jamaica was planned as a kickoff for NBS; however, the screening pilot was cancelled and replaced by the screening of children and pregnant women thought to be at risk of SCD [1281]. Currently, there is no NBS for SCD despite continued calls for its implementation by NBS advocates and the local HOPE Foundation, which publicly reminds policy makers that Barbados is one of the few Caribbean countries where NBS for SCD is not available publicly [1282]. Specimens from private pay patients are sent for an expanded NBS package at a private NBS laboratory in the USA.
- **Belize**—Interestingly, while there is currently no formal NBS program in Belize, a pilot study to demonstrate the feasibility of using the Sickle SCAN POC screening test and to gather prevalence data in support of screening was reported in 2017 [1283].
- **Cayman Islands**—In 1997, routine cord blood NBS for HGB was initiated for babies born at the Cayman Islands Hospital in Grand Cayman and at Faith Hospital in Cayman Brac. In 2002, the Cayman Islands Hospital expanded NBS to include over 50 conditions utilizing the services of Revvity Omics in Pennsylvania, USA. The Doctors Hospital also offers expanded NBS using the same commercial NBS laboratory [1281,1284].
- **Curaçao**—Curaçao is a constituent country in the Kingdom of the Netherlands. Results of a 2006 pilot have been previously noted under Aruba (above) [1278]. There is no national NBS program but there are hospital-based initiatives that offer limited NBS. Screening for HGB and CH using cord blood is offered to all newborns delivered at the Curaçao Medical Center. NBS for PKU is targeted to newborns that are not, or only partly, of Afro-Caribbean ancestry because of the lower PKU incidence in persons of African descent [1131].
- **Dominica**—CAREST is working with patient advocates in Dominica to implement NBS for SCD.
- **French Guiana**—French Guiana is an overseas territory of France and participates in the French NBS program. Specimens are sent to the screening laboratory in Lille for analysis using the French screening panel.
- **Grenada**—A two-year pilot neonatal screening program to establish the local SCD birth prevalence and to foster NBS in Grenada was undertaken in 2014–2015 at the Grenada General Hospital. Subsidized by CAREST, the pilot was a collaboration between the MOH of Grenada, the Sickle Cell Association of Grenada, and the HGB diagnostic laboratory of the University Hospital of Guadeloupe. This pilot study demonstrated the feasibility of NBS for SCD. The SCD birth prevalence resulted in a commitment from the MOH begin NBS to reduce the burden of disease in Grenada [1285]. NBS was not continued, however, due to funding and a lack of human resources [1107]. NBS HGB/SCD stakeholders continue to explore options for the continuation of the program.
- **Guadeloupe**—In 1984, a universal NBS program for SCD and other abnormal hemoglobins was initiated. In 1990, NBS became part of a SCD comprehensive management program coordinated by the Comprehensive Sickle Cell Center of Guadeloupe. The NBS program is part of the French national NBS program for SCD supported by the French Association for Screening and Prevention of Child Handicaps and by the national public medical insurance system for salaried persons. Prior to 1995, cord blood specimens collected on the day of birth were used for NBS and afterwards, specimens were dried bloodspots taken two days after birth [1286]. DNA testing is available as a second-tier test in certain instances and the Guadeloupe laboratory serves as a screening laboratory for Grenada and Tobago. Current NBS coverage exceeds 98% [1107].
- **Guyana**–There is currently no organized NBS program in Guyana. A partnership was established with Newborn Screening Ontario to initiate a pilot in 2016–2017 to report on screen-positive rates, obtain prevalence for CH and SCD, and to assess the feasibility of implementing NBS. This first-time study provided baseline data useful for informing future NBS policies. The small sample size and difficulties in follow-up limit generalizations but suggest that NBS for both conditions could be beneficial, although the data for HGB screening is more compelling. Larger pilots are needed, and a remote screening laboratory may be necessary, at least in the early stages [119].
- **Jamaica**—The original Jamaican cohort of 100,000 babies has been well studied over time leading to significant knowledge increases in the natural history of SCD [1287,1288,1289]. The current program has been ongoing since 1995, when it was restarted because of advocacy by the Sickle Cell Support Club of Jamaica. Screening coverage reached the full population in 2015. Because there is no legislative mandate, the program previously relied on funding at the discretion of incumbent policy makers [1107]. However, as SCD is now a part of non-communicable disease policy, and it is likely that the program will continue to improve. The collaboration of academics, clinicians, patients, and public health practitioners has accelerated progress [1290].
  There are two screening laboratories in Jamaica. The lab at The University of the West Indies (UWI) screening laboratory screens the eastern half of the island and the laboratory in the Southern Regional Health Authority screens the western half. Work from Jamaica has highlighted barriers to tracing affected infants and bringing them into care as part of the NBS program [1291]. The UWI screening laboratory has been an essential component of many of the island pilots and pilots in other developing countries internationally. While the typical NBS specimen is whole blood collected from a heel stick onto a special absorptive paper, cord blood is also used in some settings in liquid form. In a 2017 report, the Jamaican program reported their technique for using cord blood to validate its usability in low-resource settings in situations where cord blood is the only available option [1292].

- **Martinique**—In 1989, NBS for SCD began, mirroring NBS in Guadeloupe. Like Guadeloupe, the NBS program is part of the French national NBS program for SCD supported by the French Association for Screening and Prevention of Child Handicaps and by the national public medical insurance system for salaried persons [1107,1293].
- **Sint Maarten**—St. Maarten is a “constituent country” in the Kingdom of the Netherlands. Results of a 2006 pilot have been previously noted under Aruba (above) [1278]. There is currently no national NBS program but there are hospital-based initiatives that offer limited NBS. Screening for CH is offered to all newborns delivered at Sint Maarten Medical Center [1133].
- **St. Lucia**—In the mid-1980s, St. Lucia became the first Caribbean country to attempt NBS for CH. Cord blood samples were collected on filter paper and transported to the Illinois NBS laboratory for testing. While the validity of their blood collection and transport process was demonstrated, no case of CH was detected during the 3-year pilot and the NBS program was not continued [1294]. In 1992, supported by the St. Lucia Sickle Cell Association, cord blood screening for SCD was introduced through the MOH with the goal of working toward universal screening. Because cord blood limited the screening conditions and had unacceptably high false-positive and false-negative rates due to contamination with maternal blood and method-specific errors, heel prick DBS specimens were introduced in 2014 but not widely accepted. In an effort to improve acceptance of DBS specimens, a 2-year iPIER (Improving Program Implementation through Embedded Research) study sought to assess healthcare workers’ knowledge of and attitude toward DBS screening and to determine new mothers’ favorability toward DBS screening [1131,1295]. The use of HP specimens in St. Lucia remains a challenge, and cord blood continues as the specimen of choice [1107].
- **St. Vincent and the Grenadines (SVG)**—In 2015, a prospective NBS-SCD study of mothers and their newborns was conducted at the maternity hospital that accounts for about 95% of all newborn deliveries in SVG. This study assessed the feasibility of NBS in collaboration with the NBS laboratory at the South Carolina Department of Health and Environmental Control. Results confirmed that partnering with an established NBS laboratory is a feasible way to develop NBS when the infrastructure might not allow an in-country laboratory. It also showed that targeted NBS for SCD using the protocol in place at the time (parents’ sickle history) missed cases and was not acceptable for NBS [1296]. NBS was not continued due to funding and a lack of human resources [1107].
- **Trinidad and Tobago**—NBS for SCD in Tobago is funded by the Regional Health Authority of Tobago, which made diagnosis and treatment of SCD a priority. It has been ongoing since 2008. It now covers more than 95% of births with family members of identified cases are also screened. Indicating expanding acceptance of the Tobago program, a major obstetric hospital in Trinidad began NBS for HGB in 2018 [1107].
- **Turks and Caicos**—NBS is available to private pay patients for a limited number of conditions through arrangements with a private NBS laboratory in the USA.

#### 3.4.3. Tabular Display of LATAM NBS Information

There have been three significant reports discussing NBS in the region in the past few years, each contributing to the data contained in this section [1105,1106,1107]. With our 2015 data as a baseline, we have used data in these publications and other publicly available information from websites, meeting presentations and personal contacts to create the summary data in Table 8. As with our other tables, we have used population data from the UN, and birth and IMR data from UNICEF so that these data can be compared across tables. We have used similar disorder column headings to other tables in this report to give an overview of the strengths and weaknesses of NBS implementation across regions. NBS is still in its infancy in many countries within LATAM and the Caribbean with data are just beginning to accumulate, particularly in the Caribbean, so that blank spaces may exist in some parts of the table. For comparison with other regions, we have included a column on the number of laboratories, for which regional data do not currently exist. By displaying these and other blank spaces, we hope to encourage centralized collection of the missing data for future comparisons and areas of focus for program improvement. The data displayed here should be considered fluid and only showing a snapshot of the available information at the time of this writing.

### 3.5. Middle East and North Africa

**Figure 6:**
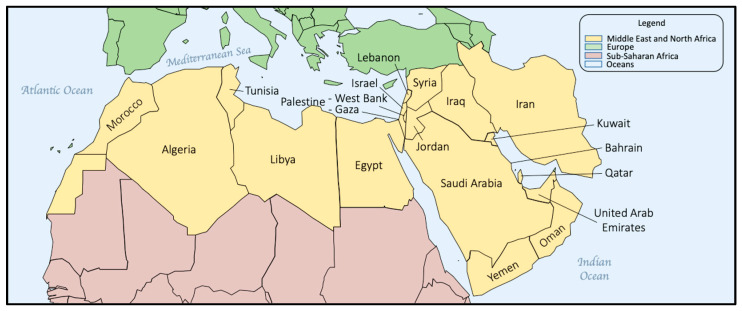
Map showing the 19 countries included in the Middle East–North Africa (MENA) region.

For purposes of our report, the Middle East–North Africa (MENA) includes 19 countries—17 of 22 countries comprising the Arab League (members Comoros, Djibouti, Mauritania, Somalia, and Sudan are included as part of sub-Saharan Africa) plus Israel and Iran (Figure 6). While Israel is geographically included in the region and therefore listed in this section of our report, its program is more aligned with European screening efforts and the ISNS includes it as part of Europe [668]. In addition to geographic closeness, these countries generally share interlocking histories and many common challenges, but they are widely diverse in their economic development. The population of the region is about 400 million, with an estimated 10 million newborns annually. There is significant diversity in geographic and population size, per capita income, health systems, and insurance coverage. The high rates of consanguinity and first cousin marriages result in increased risk for genetic conditions and a greater need for, and value of, NBS. Here we will first discuss broadly NBS activities affecting the region as a whole and then review NBS activities in the individual countries. A summary table of NBS activities will be included in Section 3.5.3.

#### 3.5.1. NBS Activities Focused on the Middle East North Africa Region

NBS continues to develop slowly in MENA with many contributing issues, including geography, politics, ethics, and logistics. In addition to the Marrakech Declaration [1297] from the first regional MENA meeting that recommended screening for at least a single condition in every country, other recommendations have also been made locally: (1) sharing knowledge, training, and research expertise (including information and other resources); (2) developing national disease registries and communication systems linking identified children with treatment providers for long-term follow-up care; (3) establishing a central NBS organization for training, surveillance, and program oversight across the region; (4) continuing to expand NBS where possible including inter-program collaborations to overcoming limited resources; (5) stimulating regional research capacity for conditions specific to MENA; and (6) educating parents, pediatric healthcare providers and policy makers about available comprehensive NBS [1298].

Significant progress in institutionalizing NBS programs in the region has occurred since our 2015 report [16]. Even though there have been four international meetings (Marrakech, Cairo, Doha, Limassol) with the goal of supporting NBS implementation in MENA countries, there continues to be difficulties in organizing and institutionalizing NBS in some countries [1299] and their goals formulated during the early meetings have not been met [1300]. Because it is difficult to find information on NBS activities in MENA countries, we have expanded the scope of this section to briefly update the current NBS situation in each country. Poverty and political uncertainty continue to challenge the development of NBS in Yemen. While there has been some NBS activity (primarily private or military hospitals) in Algeria, Libya, Morocco, Syria, and Tunisia, a formal NBS program is not yet present, although initial steps to initiate a national program have been taken by some governments. On the other hand, NBS appears to be increasing in Iraq and there is widespread NBS for one or two conditions in Bahrain, Oman, and Palestine (both Gaza and West Bank). National NBS programs for many conditions (to varying degrees) are now present in the remaining countries [Egypt, Iran, Israel, Jordan, Kuwait, Lebanon, Qatar, Saudi Arabia, United Arab Emirates (UAE)].

There have been several studies of regional interest and importance since our 2015 review. Looking to assess the prevalence of CF in the Arab world, a 2019 report reviewed the spectrum of CF mutations in 22 Arab countries uncovering a wide spectrum of mutations. While some mutations were shared with other ethnic groups, some were unique to Arab patients with several distinct clinical phenotypes reported [1301]. In view of the high incidence of HCY in Qatar (1:1800), studies of the various HCY mutations in the region are also of interest, particularly to countries still considering whether to include HCY on their NBS panel. A recent review focusing on the MENA region examined the mutations spectrum of the cystathionine beta-synthase gene, the management of HCY, and the current and potential treatment approaches in order to provide additional information for deciding about inclusion of HCY in the screening panel [1302].

Looking to the future, PIDs detectable through NBS are likely significantly underdiagnosed in countries with large populations and high consanguinity, such as MENA. While limited resources currently prevent widespread PID NBS implementation in the region, expanded NBS including PIDs has begun in some countries in MENA and studies should continue along with physician training in the skills necessary for recognizing and treating PIDs in newborns [1303,1304]. While not a primary subject of this review, it is worth noting that POC screening for CCHD is expanding in the region and an educational workshop at the 2019 Rabat NBS meeting focused on MENA issues in addition to hands-on demonstrations and discussions of lessons learned from previous screening implementations [1305]. An increase in NBS for HGB and G6PD deficiency has also been observed.

#### 3.5.2. NBS Activities within Countries

- **Algeria**—There continues to be no organized NBS in Algeria despite the recognition that cases of CH are being diagnosed late and that NBS would have a profound impact. A 2016 report showed that, absent NBS, the mean age for referral for 75 suspect cases of CH was 17.6 months with 56 true cases confirmed [1306]. A 2019 review of CH cases detected between 2007 and 2018 showed developmental delay and short stature in a number of cases diagnosed beyond 3 months of age [1307]. Both study groups appealed for CH NBS as a national emergency. While not the subject of our report, we note that NBS for hearing loss has already become available in some settings [1308].
- **Bahrain**—In 1984, recognizing the large number of genetic blood disorders, the Bahrain MOH organized one of the first genetic clinics in the MENA region and began an educational campaign to reduce the incidence of Hgb. In mid-2007, NBS was begun for HGB and G6PD deficiency in MOH maternity units [1309]. Through the years, retrospective studies have provided incidence estimates for both CH [1310], other IEMs [1311], and NHS [1312], all supporting the need for a national NBS mandate. Absent a government NBS program, private hospitals provide varied screening panels with some NBS specimens reportedly being analyzed for IEMs in Saudi Arabia [30].
- **Egypt**—Egypt has the largest and one of the fastest growing populations in the MENA Region. The Egyptian NBS program began with CH in 2000 and expanded to include PKU in 2015. The first NBS pilot in 2016 found that Egypt has one of the highest birth prevalence rates for IEMs detectable by MS/MS [1313]. Other pilots have followed, each looking at possible conditions to be added to the NBS panel, including GALT and G6PD deficiency [1314,1315,1316,1317]. In 2021, NBS was expanded nationally to include 19 conditions, with Phase I consisting of screening babies born at public hospitals and Phase II expanding the scope to includes babies born at university hospitals, private hospitals, and healthcare units [1318]. As a part of “Egypt 2030”, many health programs, including NBS, will undergo digital transformations in the coming years. This effort by the Ministry of Communications and Information Technology is intended to make internal communications and data transfer from peripheral sites easier and more efficient [1319]. Building on earlier research from the German NBS program, Egyptian collaborators have also participated in research demonstrating the feasibility of NBS for cystinosis using molecular techniques [818,1320]. Recently, screening for HGB and SMA have been included in the available NBS tests and screening for HGB has been included in premarital screening tests. A 2023 report reviews the impact of rare diseases in Egypt including newborn screening and the Egyptian Genome Project, which began in 2021 [1321].
- **Iran**—Based on the success of pilot studies in three provinces, and basic support from IAEA, national NBS for CH in Iran was integrated into the national health system in 2005 and newborn coverage now exceeds 95% [20,1322]. To assess the quality and outcomes of the NBS program, at least three audits were completed in recent years, which showed normal growth and development at school age because of early diagnosis and treatment [1323], and good coverage but high recall and losses to follow-up. The latter program issues were addressed through the re-evaluation of cutoff levels and better surveillance of follow-up [1324,1325]. Originally, the national NBS for PKU began because of its relatively high incidence in the Iranian population and corrections were also made through cutoff changes [1326,1327]. A 2014 report noted that when NBS for G6PD deficiency was begun nationally, sensitivity issues with the laboratory procedures were also reported as implementation obstacles [1328].
  A detailed analysis of the implementation of NBS for PKU and related policies pointed (at least partially) to the continuous persistence of parents and interest at the public health executive level (a top-down approach) as significant factors in program implementation [1329]. Recently, a study on the quality of life of parents of children with PKU showed a need for interventions to help the parents better cope with the problems associated with PKU detected through NBS. In addition to family education to increase awareness and improve attitudes, increased financial support for families, funding assistance for PKU patients from support organizations, and medical cost coverage from insurance companies were identified as probable benefits [1330].
  Expanded NBS with MS/MS pilots for laboratory and clinical follow-up were begun in six provinces in 2017. Ten laboratories nationally were certified by the MOH, each linked to a referral hospital with a panel of metabolic specialists [1299]. Looking towards future screening expansion, at least one pilot study has evaluated the use of MS/MS and other second-tier tests to improve screening quality and to consider the possibilities of adding up to 38 possible IEMs. This study included the evaluation of reference ranges and screening cutoffs [1331]. To replace the current manual data management system, a framework for implementing electronic data management has been proposed based on literature reviews of the data management systems in the USA, UK, and Australia [1332]. A recent study compared and validated for the first time, two fluorometric methods for measuring α-glucosidase acid activity in dried bloodspot specimens (DBSs), with potential use for NBS and diagnosis of PD. in Iran [1333]

- **Iraq**—Building on a pilot begun in two provinces in 2013, the U.S. Agency for International Development (USAID) assisted with a 2014 project to screen for PKU, CH, and GAL using the project’s detailed instruction guide [1334,1335]. Reviews of the developing program showed lower coverage than desired but progressing. Recommendations included expansion of the screening panel and increased implementation to more provinces [1336,1337]. A 2020 report noted a continued lack of expansion but plans to cover all 18 governorates and expand the screening panel [1334]. A study of mothers’ knowledge and attitudes towards NBS showed enthusiasm despite a lack of knowledge [1338].
  A 2019 report of CH birth prevalence in the Kurdish part of Iraq, Duhok Governorate, showed a prevalence of 1:250–1:900 across the governorate, which includes a mountainous iodine-deficient area [1339]. A pilot non-profit, non-governmental program, managed by a private lab, provides seven basic screening tests for a small fee. Expansion to approximately 60 screening tests by this laboratory has been implemented, with the possibility of providing screening on a national level [1340]. The Duhok NBS laboratory is the only facility performing screening for the LSDs. Because NBS is not yet routine across the whole of the country, and there is a high degree of consanguinity lack of metabolic specialists, conditions like PKU (which are likely higher in number than currently reported) are being diagnosed late. There is a growing concern over the lack of full coverage for all newborns through a national NBS program [1341]. There are currently two larger screening laboratories (Baghdad and Karbala) with gradual expansion occurring nationally.

- **Israel**—Like many Western countries, Israel has maintained a NBS program since the 1960s beginning with PKU and later adding CH [13,1342]. An expanded NBS panel of 11 additional conditions was implemented in 2008 [1343]. Screening is free of charge as part of “The Healthcare Basket” under the National Health Insurance Act [1344]. A website with program information and secure screening results was launched in 2009. A study exploring the views of health professionals regarding parental education and informed consent for screening after expansion indicated a need for in-depth discussions and considerations regarding parental education, result communication, and informed consent processes for screening prior to any future program expansion [1345].
  Following a successful pilot study and anticipating a high incidence because of consanguinity, NBS was expanded to include SCID. A report on the first year of screening and a 5-year data review have been published [1346,1347] along with an upbeat editorial comment [1348]. Noting an incidence as high as 1:29,000, the authors of the 5-year study called for SCID screening globally, particularly in consanguineous populations. Also, recently there was increased interest in adding NBS for the galactosemias to the screening panel. To develop a NBS pilot algorithm for galactosemia, archived dried bloodspots from newborns with GALT, galactosemia variants, and normal controls were analyzed by MS/MS for galactose-1-phosphate (Gal-1-P) by FIA MS/MS. Based on a successful pilot, a screening algorithm with Gal-1-P as the first-tier screening test, and GALT enzyme activity as the second-tier to identify newborns suspected to be at risk for classical galactosemia has been implemented [1349]. Another FIA MS/MS study demonstrated the feasibility of screening newborns for CTX, a progressive metabolic leukodystrophy, using two-tier screening with FIA MS/MS and LC MS/MS [1350].
  Also not on the current screening panel, CF case detections identified through the national population carrier screening (PCS) pro/gram have been reviewed and found to support a more balanced case detection approach using both PCS and NBS [1351]. A review of the barriers to adding CF to the NBS screening panel suggests a balance of bioethics and cost effectiveness should be used in considering whether CF should be added to the screening panel [1352]. Other interesting research projects possibly affecting NBS policy include: (1) a study of the effect of NBS on rea productive decision making following the detection of a child with a disorder detected through NBS [1353]; (2) a study of the founder effect of a pathogenic variant among Bukharan Jews associated with severe early-onset Wilson’s disease, for which NBS could lead to early diagnosis, treatment, and improved outcomes [1354]; and (3) decisions of which conditions to screen and exploration of the different ‘gene worlds’ that constitute NBS programs in Israel and the US [1355]. Looking to the future, provocative articles on next generation sequencing in NBS have been published by Israeli researchers [1356,1357].

- **Jordan**—Following a PKU and CH pilot initiated in 2004, the first national NBS project screening for PKU was begun in 2006 along with a nationwide training program for healthcare workers. The NBS program was expanded to include CH in 2008 and G6PD in 2012. Currently, 29 conditions are included and at least one private laboratory provides a larger number [1358,1359]. A recent report reaffirmed the importance of the NBS program for early identification and treatment of PKU and other genotyped and biochemically characterized Jordanian hyperphenylalaninemia patients for the first time [1360]. A retrospective study of TYR-I found that a lack of clinical familiarity with the disease resulted in delayed diagnosis which might be ameliorated by inclusion of TYR-I on the NBS panel [1361]. A 2022 report on mothers’ NBS knowledge and attitudes confirmed that healthcare providers, particularly nurses, were the main source of mothers’ information and education and their knowledge should be improved [1362]. For health workers and parents, a 2022 comprehensive NBS manual in Arabic exists online [1363].
- **Kuwait**—The Kuwait NBS program began in 2005 with two conditions, PKU and CH, and was limited to public hospitals. In 2014, the Kuwait Ministry of Health began a publicly funded, expanded NBS program screening for 22 conditions. The aim of the program was to ensure that all infants born in Kuwait were (and are) screened. Before April 2015, NBS was only offered in public hospitals since private hospitals individually offered their own screening testing, often provided by screening laboratories in the U.S. In May 2019, the Kuwait NBS program, located in the Kuwait Medical Genetics Center (KMGC), began covering 100% of newborns born in Kuwait. Noteworthy has been the increased detection of cases of homocystinuria by utilizing the methionine to phenylalanine ratio to improve predictability [1364]. Kuwaiti experiences with NBS for VLCAD deficiency have also been reviewed and reported. This study provides evidence that expanded screening detects cases of VLCAD. Molecular genetic testing for the ACADVL gene was recommended for inclusion in the screening algorithm [1365].
- **Lebanon**—NBS in Lebanon has been ongoing since 1996 when it began as a fee-for-service program in both Saint Joseph University in Beirut (USJ) and the American University of Beirut Medical Center (AUBMC). Its expansion to include MS/MS technology in 2006, involved collaboration between the NBS laboratory at USJ and the metabolic laboratory at the Hamburg University Medical Center (HUMC) [1366]. Additionally, an independent laboratory began to offer expanded NBS, which in theory made screening available to all. The fact that NBS cost was usually borne by the patient likely decreased the number screened by half. A 2015 cost-effectiveness study provided an evidence-based model to encourage the government to make NBS free and provided a model for other developing countries in the region. It encouraged patient advocacy and other concerned civil activist groups throughout the region, “to revive their demands for mandatory universal publicly funded NBS for IEM [1367].” Also in 2015, a review of genetic disorders highlighted the urgent need for community genetic services nationally. It noted that NBS services were generally localized within the capital and not nationally mandated [1368]. Another 2015 study by AUBMC found a high alpha-globin carrier rate and highlighted the presence of two common alpha gene mutations in the consanguineous Lebanese population [1369]. This supported the conclusions of an earlier study on the presence of sickle cell and other hemoglobin variants and supported the need for national NBS [1370]. In 2018, the government launched its “National Program of Neonatal Screening for Primary Immunodeficiency Diseases” (NaSPID), noting that, “*babies born under the coverage of the MOH will have the screening test done with no additional costs*” [1371]. Currently, NBS is available to all residents in Lebanon through three community-based universities and screening coverage exceeds 50% despite a severe nationwide economic crisis. The NBS program in Lebanon has assisted several other developing NBS programs [1372].
- **Libya**—Targeted NBS exists in Libya in certain high-risk situations. For example, babies with diabetic mothers are screened for CH and those with cataracts are screened for GAL [1373]. A 2010 study evaluated the cost- effectiveness of establishing a NBS program for PKU in Libya and showed that implementing a NBS program for PKU would generate a 90% return on investment [1374]. A 2022 report outlined the extent of IEM in Libya and supported implementation of a NBS program [1375]. A survey of Libyan physicians rated lack of technology, infrastructure, and specialized medical professionals as the largest obstacles to developing a NBS program [1373]. A suggested possible solution involved stepwise NBS implementation using a laboratory and/or other support from another country. This type of collaboration was essential to establishing sustainable NBS in Qatar, Lebanon, the Philippines, and others [1366,1376,1377]. In 2019, a new IEM Committee was formed to organize NBS. A central laboratory in Tripoli is planned along with the ambitious goal of screening for a large number of disorders [1378], yet nothing has been implemented due to lack of funding.
- **Morocco**—The first MENA Regional NBS meeting, Enhancing Neonatal Screening in MENA, was held in Marrakech in 2006 to begin to organize and expand NBS in the Region (see Marrakech Declaration [1379]). During 2010–2011, a NBS steering committee was organized by the MOH and the first phase of a pilot NBS program was launched in the Rabat region. Assistance was provided by the Japanese International Cooperation Agency (JICA) [1380], including training and a program review with recommendations for improvements. In 2013, the MOH created Ministerial Circulars requiring the availability of CH screening at all childbirth facilities and in 2014, designated a specific protocol for diagnosis and therapy of confirmed cases. NBS has continued to expand in university hospitals with continuing interest in adding other conditions such as PKU [1381]. A recent pilot study in 34 centers in Fez determined an incidence of CH of 1:1952 [1382]. While there is not yet a sustainable national NBS program, there is expanding interest and periodically a new facility or laboratory will begin screening, increasing the number of screened babies. A recent report on the use of MS/MS in a targeted study in Rabat showed that IEMs detectable by MS/MS are present in the population and that NBS using MS/MS would be useful for their detection [1383].
  CCHD screening is also receiving attention in Morocco and a recent collaborative pilot study between Mohammed VI University Hospital of Marrakech and Children’s National Hospital in Washington, DC, USA demonstrated the feasibility of including CCHD screening in the developing NBS program [1384].

- **Oman**—Building on experiences from a hospital cord blood NBS program initiated in 1991 and a 2004 pilot, the National NBS Program for CH was begun in 2005 using cord blood [1385,1386]. This program was provided to all newborns in all healthcare facilities, including MOH hospitals, extended health centers, sister government and private health institutions. Detailed guidance for CH NBS was created in 2010 by the MOH and updated in 2021 [1385]. Because there is a high incidence of HGBs in Oman and the cord blood used for CH screening can be used for HGB screening, a prospective NBS study provided HGB incidence data and suggested HGB addition to the CH NBS program. [1387]. A later study showed the capabilities of HPLC in detecting HGBs from newborn specimens, but the NBS program was not expanded [1388]. Expansion to include other IEM conditions using MS/MS and other procedures was proposed in detail noting that diagnostic MS/MS screening for multiple IEMs had been available at one Omani university hospital since 2002. The combination of years of diagnostic experience coupled with a recently opened genetic center appeared to provide the necessary expertise for NBS follow-up [1389].
  While expanded NBS that includes MS/MS technology is not yet available in Oman, this may soon change. In anticipation of the need for reliable references ranges for laboratory testing, a study has already been completed providing reference ranges for most of the conditions that would be included [1390]. Similarly, anticipating the possibility of NBS expansion has resulted in the generation of a study defining how SCID might be added to the program. Finally, NHS was introduced nationally in Oman after limited pilot testing in 2002 [1391]. Experiences with this program are detailed elsewhere along with the challenges of adding NBS to the public health system in a developing economy [1392,1393].

- **Qatar**—The Qatar NBS program is one of the more advanced in the region. In late 2003, collaboration began between Hamad Medical Corporation and the University Children’s Hospital of Heidelberg in Germany, updating a cord blood CH screening program operational since 1996 [1376]. The NBS collaboration began with 28 screening conditions with an eye towards expansion when and where possible. Recognizing the high numbers of clinical cases and the opportunities for early detection and treatment from NBS, HCY and HGB were added to the program in 2014. Additional MS/MS disorders were also added along with second-tier testing for MMA and methyl citric acids. Methodologies to detect LSDs were reportedly under evaluation [1394,1395]. A two-step NBS approach to diagnosing carnitine deficiency in extremely pre-term newborns has also been reported [1396]. A 2020 report noted that NBS in Qatar included over 80 disorders, which required a wide range of technologies including photometric techniques, MS/MS, and liquid chromatography. Second-tier testing was being used for some disorders (abnormal levels of methionine, homocysteine, etc.) to reduce unnecessary recall. Next generation sequencing (NGS) using an in-house NBS panel of 122 genes associated with 63 metabolic and 18 other NBS disorders was also in use. Screening availability and treatment breakthroughs with SMA make its addition to the Qatari screening panel likely [1299,1397].
- **Saudi Arabia**—An NBS program for PKU, CH, HGB, and G6PD was initiated by Aramco for its 60,000 employees in 1980 [1398]. Through the combined efforts of the Ministry of Health, King Salman Center for Disability and Research, King Faisal Specialist Hospital, Research Center-Riyadh (KFSHRC), and others, a national NBS program for 16 conditions based on disease frequency and availability/accessibility of confirmatory tests and treatments was begun in August 2005. The national NBS committee that selected the 16 tests was also tasked with overseeing the screening program, future planning, policy development and performance evaluation. The NBS program began with 24 centers and an aim of full population coverage within 10 years (based on funding availability). The panel of conditions was soon expanded to 17, with the addition of VLCAD, and 3 additional conditions were proposed for addition, TYR-I, HCY, and free carnitine [1399].
  A 2015 report of the knowledge and attitudes of Saudi mothers towards NBS found high acceptance of NBS among Saudi women but a considerable need to increase awareness by improving communications between the medical community and parents [1400]. A later study (2020) concluded similarly that medical healthcare professionals were responsible for interacting with various NBS stakeholders, including families of screened newborns and prenatal educators [1401]. In 2016, keeping with the Kingdom’s “Vision 2030” goals, an Institutional Transformation and Health Care Model was developed that included as one of its foci “Health Promotion Against Health Risks” to increase public awareness of programs such as NBS [1402].
  In 2016, the MOH announced the inclusion of CCHD and NHS in the national screening effort. Included in the announcement were plans for implementation in 30 MOH referral hospitals [1403]. A 2017 Letter to the Editor questioned national readiness as NBS expanded and noted the need for establishing NBS as a comprehensive system [1404]. A review NBS and the five regional programs in Saudi Arabia the following year noted the success of expanded NBS in the center of the country, and suggested that, “*MOH should implement plans to start testing for the maximum number of conditions in all of its five branches at one time and with the same diseases … and there is no single region immune from any disease…*” [1405]. Also in 2017, a review and review response provided regional and national incidences for all screened disorders based on data from 2005–2012, with particular emphasis on the apparently low incidence of CH [1406,1407]. In 2018, a unification/harmonization of NBS occurred during the COVID-19 pandemic when MOH laboratories were centralized from three laboratories to one laboratory, National Health Laboratory, in Riyadh. There are several smaller volume laboratories outside of Riyadh and there are now eight NBS laboratories throughout the country working in parallel.
  Looking to the future, a 2018 report summarized a TREC-based SCID NBS pilot study which confirmed the high incidence of SCID in the Saudi population and demonstrated, “*…the feasibility of using T-NGS PID panel from Guthrie card DBSs as a new reliable, rapid, and cost-effective mutation screening method for newborns with low TREC counts*” [1408]. A prospective NBS pilot study of TOXO was initiated to serve as a basis for developing neonatal screening policies in the Eastern Province of Saudi Arabia, considering the health plan and the Kingdom’s health system outlook [1409].
  Since population-based laboratory information on disease ranges, cutoff values, etc. for Saudi Arabia were previously lacking, a 2022 study reported clinical disease ranges for most of the Saudi NBS analytes and several of the ratios used to improve screening specificity and sensitivity [1410]. Also in 2022, a review explored the possible impact of implementing an updated health information exchange (HIE) system into an ongoing LTFU data system and the importance of having a well-established collaboration infrastructure. It also surveyed the current HIE status of the Saudi National NBS program and barriers that might be faced on implementation [1411]. Another 2022 report reviewed readiness for NGS in Saudi Arabia finding that the question of “Are we ready yet?” is still up for debate [1404,1412]. A recent publication reported on the accurate determination of biotinidase activity in serum by HPLC and its utilization as a second-tier test for the confirmation of initial positive newborn screening results [1413].

- **Syria**—Despite training assistance in NBS for CH from the International Atomic Agency in the 1990s, a national NBS program still does not exist in Syria [20]. There have been several studies relating to NBS in recent years. A 2015 report summarized the results of two studies looking at traditionally screened conditions (CH, GAL, CAH) and conditions identified by MS/MS (FAOs, AAs, OAs). The incidences of these disorders supported “*consideration of a comprehensive nationwide neonatal screening program*” [1316]. A 2015 report also summarized the results of a 5-year review of CAH cases and supported the idea of a mandatory NBS program, with further efforts needed to confirm its effectiveness in Syrian society [1414]. Another 2015 study looked at OAs and a 2019 study looked at CH with both concluding that NBS was needed [1415,1416]. Other reports supporting NBS are available in non-English journals. In early 2019, the military health authorities began a limited panel of NBS conditions for military newborns.
- **Tunisia**—Even though in the 1990s Tunisia was featured by the IAEA as a model NBS pilot project for CH with the intent to develop nine screening centers in the country, there is currently no systematic NBS program [1417]. More recently, studies have demonstrated the detection of hemoglobin disorders, a major local health problem, in a more cost-effective way using local materials and methods [1418]. The presence of genetic diseases amenable to NBS, including PKU, have also been reviewed, including a limited number of NBS pilot projects [1419,1420]. These studies not only assess the status of genetic diseases in Tunisia, but also emphasize the importance of early interventions, like NBS, to reduce morbidity and mortality. One report describes many of the reasons to have a national NBS program in Tunisia and the barriers to implementation and suggests a targeted cascading carrier screening alternative to implementing systematic NBS [1421]. A 2018 genetics review identifies some of the major healthcare (and NBS) challenges, noting that, “*… there is an urgent need to increase genetic literacy among the health care personnel, in particular primary care practitioners, by providing the appropriate education and training. … The public must be informed and educated to seek services and counseling by creating support groups, organizing workshops, and by creating a network between the different care providers, the school or institution and the parents*” [1422].
- **United Arab Emirates**—A national NBS program for PKU was established in the UAE in 1995. CH was added in 1998 and screening coverage was 50%. Screening conditions gradually expanded over the years to include MS/MS testing in 2011 and screening coverage reached 95%. Expansion to include even more conditions occurred in 2013 [1423]. A 2016 report pointed to possible testing improvements including integration of NBS and the premarital screening program “*to better mitigate IEM disorders in the community*” [1424]. Interestingly, a recent report from Gulf Cooperation Council Countries that included summaries from 3 studies in UAE noted that in the case of thalassemias, “*Despite the premarital screening and genetic counseling (PMSGC) program for thalassemia, the incidence of high-risk couple marriages in GCC countries cannot be effectively diminished*”. This study suggested that, at least for thalassemias, an enhanced level of consciousness about the disease and the consequences of consanguinity might be more effective than the PMSGC in reducing the incidence of disease [1425]. A 2021 report of a 5-year project in Dubai found that, among other results, G6PD deficiency was the most prevalent condition found by NBS [1426].
  Point-of-care screening also occurs in the UAE. A 2013 report on the implementation of CCHD in Abu Dhabi, in collaboration with Children’s National Medical Center (D.C.—USA), suggested the value of a national screening program [1427]. In 2018, the Ministry of Health and Prevention (MOHAP) launched a CCHD screening program for all newborns [1428]. A 2022 report noted that the Emirates Health Services (EHS) had adopted a stringent NBS program for CCHD and showed how an electronic medical record (EMR) could improve the efficiency of the program [1429]. Additionally, a local hearing screening project in 2021 recommended a national NHS program and highlighted “*the importance of parental engagement to increase the awareness regarding timely NHS, follow-up NHS, and hearing loss in children in the UAE*” [1430]. In addition to CCHD and NHS, 49 conditions from DBSs are included in screening. A national law is under consideration that would “*obligate all health sectors, hospitals and families to participate in the test*”. Additionally, NBS expansion to include SCID is expected sometime in 2023 [1431].
  A recent UAE report noted that, “*Studies of genomic NBS are highly skewed towards populations in high-income countries. The evidence generated by these studies will be similarly biased and is likely to lead to disparate global implementation. Studies inclusive of historically under-represented populations are needed for equitable global access to genomic newborn screening*”. It concluded with a call-to-action, commenting that, “*Inclusive studies involving globally diverse populations will be needed to achieve equitable implementation of genomic screening for all newborns irrespective of their ancestries or geographic origins. Failure to achieve this goal will exacerbate the disparate access to genomic medicine for understudied populations*” [1432].

- **West Bank and Gaza**—Approximately 60% of the Palestinian population resides in the West Bank and 40% in Gaza. NBS began in 1994 and currently, while screening laboratory services are provided by two separate laboratories, screening coverage is high and only screening for CH and PKU is universally provided [1433,1434]. Incidence studies of the IEMs in the West Bank (a collaboration between the MOH and Liege University in Belgium) and G6PD studies in Gaza support expanding the NBS program [1435]. A 2020 report defined a PKU cutoff for laboratory use to replace the kit manufacturer’s recommended cutoff, which was in use at the time in the West Bank screening laboratory [1436]. In 2021, a shortage of PKU test materials likely due to supply chain issues from the pandemic caused a backup in testing and threatened the stability of the NBS program in Gaza [1437].
- **Yemen**—While representatives from Yemen attended the first regional MENA meeting, there has been no information regarding the status of NBS activities in Yemen.

#### 3.5.3. Tabular NBS Data for Middle East North Africa

We have provided basic information on NBS activities in MENA based on our 2015 report, published reports over the interim, and personal contacts within the various countries and the region (see Table 9). We have attempted to show the extent of screening for the various conditions. The relatively high rates of consanguinity across the region predict high impact from NBS for most IEMs [1438]. In the case of HGB, its inclusion in the NBS program is often influenced by the presence of a strong premarital screening (PMS) program [1439]. Following on successes reported in the first PMS for hemoglobin disorders in Cyprus in 1973, programs in the MENA regions have been ongoing in many countries in the region since the 1990s: Lebanon (1994), Iran (1997), Palestine (2000), Saudi Arabia and Jordan (2004), Bahrain (2005), UAE (2007), Iraqi Kurdistan (2008), Kuwait (2009), and Qatar (2012) [1439,1440,1441,1442]. More recently, PMS became a legal mandate in Egypt in 2023 [1442]. However, there is still no consensus on whether HGB should be included in NBS.

### 3.6. Sub-Saharan Africa

**Figure 7:**
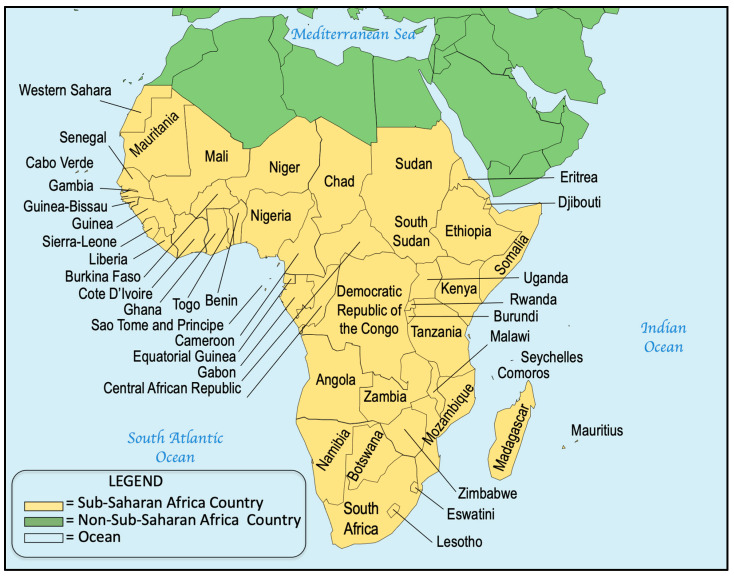
Map showing the countries of sub-Saharan Africa.

Sub-Saharan Africa (SSA) spans the bulk of the African continent south of the Sahara Desert (see Figure 7). It encompasses a diverse range of countries and landscapes, rich in cultural heritage, natural resources, and ecological diversity. It is bordered by the Atlantic Ocean to the west, the Indian Ocean to the east, and the countries of the MENA region bordering the Mediterranean Sea to the north. SSA is characterized by a variety of ecosystems, including dense rainforests, expansive savannahs, arid deserts, and lush coastal areas. It is home to a diverse population belonging to numerous ethnic groups and speaking more than 2000 different languages. The region exhibits wide-ranging cultural diversity, with vibrant traditions, art, music, and folklore. Economically, it is known for its abundance of natural resources, including oil, gas, minerals, and arable land. Unfortunately, SSA faces many development challenges, including poverty, political instability and conflict, and inadequate infrastructure. Despite these challenges, many countries in the region have experienced significant economic growth in recent years.

**Figure 8:**
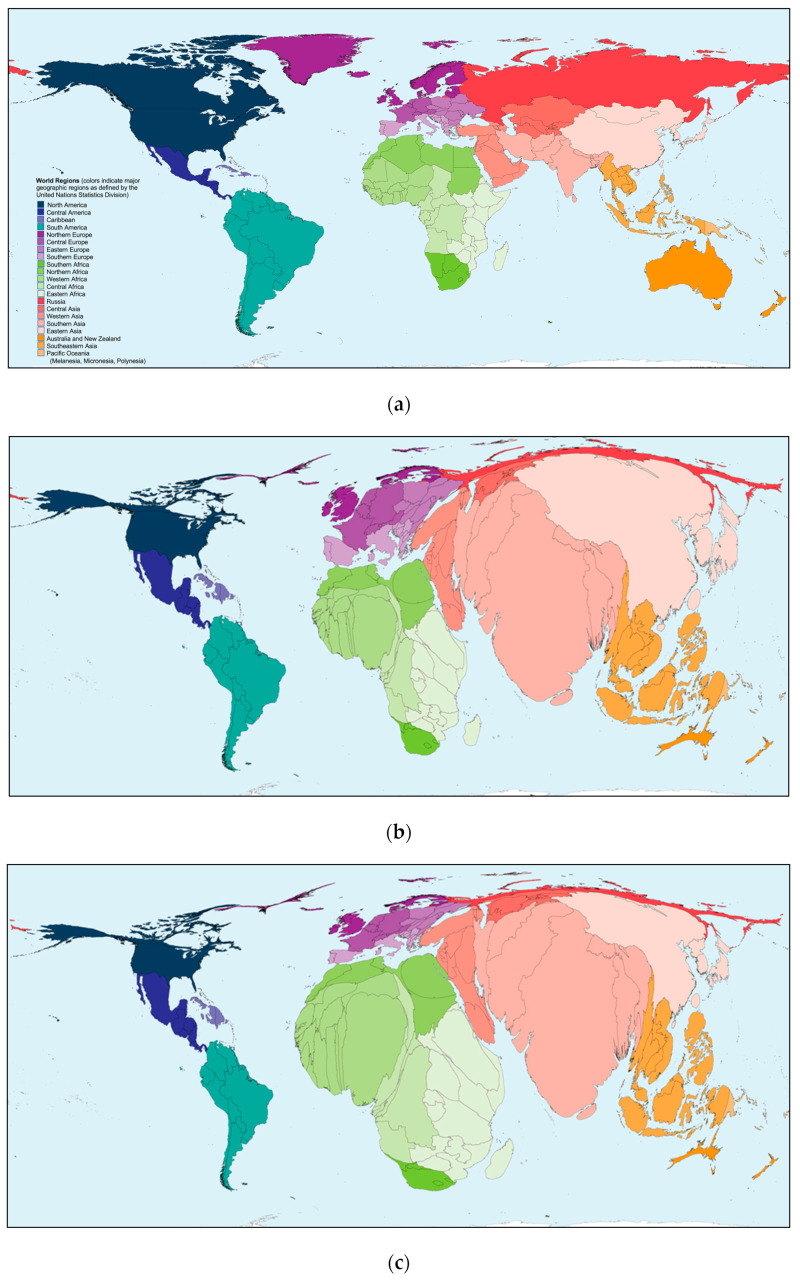
(**a**) Cartogram showing conventional view of world land masses (for reference), (**b**) cartogram showing proportional land masses based on current populations, (**c**) cartogram showing proportional land masses based on current births showing influence of sub-Saharan Africa (green). [Note: All maps are available online at: https://worldmapper.org/ (accessed on 10 January 2024)].

SSA has a population of 1.2 billion with over 41 million births annually across 55 countries. Overall, 25 of the 30 poorest countries in the world are in SSA along with 45 of the top 50 countries with highest birth rates (see Section 3.6.3). The result is a burgeoning population struggling to overcome poverty while seeking to improve quality of life. The impact of the large and increasing birth rate in Africa, and particularly SSA, can be more easily visualized in the three cartograms shown in Figure 8. 

Compared to a normal world map of relative land masses (a), resized maps of the various countries’ land masses are displayed according to total population (b) and total births (c). The UN predicts that the population of SSA will double by 2050, a 10-fold population increase, primarily due to high fertility rates and improving medical care in turn leading to fewer childhood deaths, increased numbers of young people (childbearing age), and longer life spans. The net result will be a world population of almost 10 billion in 2050 (compared to about 8 billion now) of which 25% (about 2 billion) will reside in SSA [1443]. This population growth will continue to pose significant challenges in provision of essential services—healthcare, education, and infrastructure. There is currently limited infrastructure, inadequate healthcare facilities (hospitals, clinics, and healthcare facilities), and a shortage of healthcare professionals, particularly in rural areas. Lack of medical equipment and maintenance, essential medicines, and trained healthcare personnel further strain the healthcare system. While limited public funding is sometimes available, high out-of-pocket expenses combined with the lack of effective health insurance systems often leads to financial barriers for persons seeking healthcare services.

#### 3.6.1. NBS Activities with a Regional Focus

As in other LMICs, communicable diseases (HIV/AIDS, malaria, tuberculosis, and other neglected tropical diseases) pose a major health challenge and require substantial resources for prevention, diagnosis, and treatment. High maternal mortality rates, low rates of births with skilled attendants, and limited access to quality prenatal and postnatal care contribute to poor maternal and child health indicators. Much of SSA is working to strengthen the various healthcare systems by investing in infrastructure, training healthcare workers, and improving access to essential medicines. Initiatives such as community health workers, mobile health technologies, and innovative healthcare delivery models are being implemented to overcome some of the challenges. It is important to note that there are significant variations within SSA itself with some countries making significant progress in improving their healthcare access and outcomes, while others continue to face significant challenges. Many of the countries in the region are not experiencing the classical epidemiological transition completed by HIC/developed countries decades earlier. Instead, many LMICs in SSA are facing continued burdens from infectious diseases, which parallel the growing mortality and morbidity from non-communicable diseases, including congenital disorders and rare diseases.

Approximately 70% of the world’s 300,000 persons with SCD reside in SSA and SCD has recently been shown to be the most common contributor to mortality globally among 5–14-year-olds [1444]. Efforts to address SCD are focused on multiple fronts including early and accurate diagnosis through NBS programs [1445]. Other activities include raising awareness about the disease, providing educational counseling and support services to affected individuals and families, and improving access to comprehensive care (when available), including vaccinations, preventive interventions, and disease management strategies. NBS has been recognized as a valid prevention activity for SCD in SSA since the early pilots in Benin (1993) [1446] and Ghana (1995) [1447]. A review of outcomes of NBS pilots, published literature, and lessons learned from experiences in Kinshasa (Democratic Republic of the Congo) and Ouagadougou (Burkina Faso) was published in 2008, noting that there must be a balancing of public health priorities to implement NBS [1448]. A cost-effectiveness study of NBS and prophylactic intervention (NSPI) for SCD (only) in 47 SSA countries was reported in 2016. The NSPI package was found to be cost effective in most SSA countries; however, NBS for SCD remains unavailable at the population level in SSA countries [1449].

While NBS for CH is the most productive screening strategy globally, CH as a part of NBS in SSA is minimal and its incidence across SSA is not well known, with limited reports available only in a few SSA countries to date. A 2012 review of CH and NBS in SSA noted the positive benefit to cost ratio of CH NBS worldwide and suggested that every pregnant woman should be informed about CH and the potential of NBS. Further, the authors suggested that NBS for CH should be made mandatory and offered freely throughout SSA, like birth registration and immunizations [1450]. While CH screening is available in private settings in some countries in SSA, interest in its inclusion in a national program has been limited [1451], largely because the corresponding burden of disease and economic and health benefits are yet to be comprehensively quantified in the region. A 2023 review highlights the challenges associated with testing for SCD and discusses some of the strategies for meeting these challenges and future efforts. Further, it discusses perspectives for expanding efforts like NBS for detecting and managing SCD in Africa, including the provision of affordable diagnostic tools, mobile clinics, government subsidies, education campaigns, and electronic medical records to help in bridging gaps in health service delivery [1452].

In 2018, the American Society of Hematology (ASH) initiated the Consortium on Newborn Screening in Africa (CONSA) aimed at demonstrating and evaluating the effectiveness of NBS for SCD in SSA. The CONSA network includes seven countries—Ghana, Kenya, Liberia, Nigeria, Tanzania, Uganda, and Zambia, and all countries successfully launched NBS by the end of 2021 [1453,1454]. Current CONSA funding supports a 5-year project that includes collaborations between ASH, African-based hematologists, allied health professionals, and other organizations with the intent of ensuring long-term sustainability through government, corporate, and other partner support. Current funding supports 11 laboratory sites focused on implementing standardized HGB NBS for HGB, early intervention in detected cases, and data reporting to a shared database. There are presently several SCD databases across SSA, each with its own purpose and construction and without interconnectivity. There is increasing interest among SSA countries and other SCD collaborative organizations in sharing patient data for clinical and epidemiologic purposes. Within this context, a pilot database for this purpose has been developed by the Sickle Pan-African Research Consortium (SPARCO—see below) and is currently under evaluation [1455].

There are many ongoing SCD collaborations within SSA focused on the same final goal (decreased morbidity and mortality), each seeking to complement the other without added or duplicated effort. Often the same individuals are involved in multiple organizations/projects across the region because of their leadership role in their home country. Since we are focused here on NBS, many of these collaborations are not discussed even though they may have peripheral impact on the availability and functioning of NBS. As one example, a 2020 article describes both the need and operation of SickleInAfrica [1456]. Funded by the U.S. National Institutes of Health, SickleInAfrica was organized to connect clinicians, academics, and scientists from existing SCD programs, with ongoing activities in health, education, and research embedded in local infrastructures. It has three initiatives: (1) SPARCO, with sites in Tanzania, Nigeria, and Ghana to develop research capacity for SCD through a multidimensional approach; (2) SADaCC (Sickle Africa Data Coordinating Center) in South Africa, the administrative center coordinating data standardization and supporting communications for SPARCO; and (3) SPAN (Sickle Cell Pan-African Network), a network of researchers, clinicians, funders, and centers working in SCD in 22 sites in 17 countries across Africa.

Several international NBS meetings have been held in and around Africa since 2006 [1445]. Unfortunately, these meetings focused on NBS in MENA with little attention paid to SSA. To remedy this situation, the First Pan African Workshop on Newborn Screening was held in Rabat, Morocco in July 2019, to assist ongoing efforts to implement sustainable NBS in SSA [1457]. Stakeholders from within Africa, and global experts in NBS and SCD discussed NBS rationale, techniques, system development, implementation barriers, ongoing research, and collaborations within and outside of Africa. Updates on NBS activities in the region until 2019 are included for 11 countries in the meeting report, specifically, Angola, Benin, Democratic Republic of Congo, Malawi, Mali, Nigeria, Sierra Leone, Tanzania, and Uganda. Updates to these reports and reports on activities in other SSA countries are included in Section 3.6.2.

Of increasing interest in some SSA settings is the potential use of point-of-care (POC) testing. Since the current NBS methods for HGB are relatively expensive and complex, and results are often not available for days due to transport difficulties over vast geographical distances, and other analytical and post-analytical issues, there has been an ongoing interest in developing an inexpensive sensitive and specific POC test, both for screening newborns within the first day or two after birth, or later when the first immunization is due [1458,1459,1460]. An extensive review of evaluation studies of commercial POC tests and those in development was published in 2017 [1461], and a 2018 white paper from the NBS working group of the Africa Sickle Cell Research Network (AfroSickleNet) identified POC testing as a possible solution to more widespread implementation of NBS for SCD [1462]. Several other articles have focused on various aspects of successful NBS, and reports on clinical outcomes have included reference to the value of POCs [1463,1464,1465]. A 2020 report from Nigeria on integrating POC testing into an ongoing immunization program [1466] prompted an editorial comment on the possibility of this combination as a model for other SSA programs [1467]. A recent editorial commenting on two different successful methods and strategies for SCD screening in Mali and Denmark suggested that “*countries should tailor their screening programmes according to local needs, resources and opportunities*” [1468]. References to other recent experiences within various countries are included in Section 3.6.2.

It is important to note that the WHO Regional Committee for Africa is monitoring the progress of NBS for SCD in SSA and periodically reports on regional and national activities. In its 10-year report in 2020, NBS for SCD was listed as an “*important component of surveillance and prevalence determination*”. It was available at the subnational level in 12 Member States (Benin, Burkina Faso, Cameroon, Democratic Republic of the Congo, Ghana, Kenya, Liberia, Mali, Nigeria, Senegal, Tanzania, and Uganda), which was below the 50% implementation target for all Member States. NBS was reportedly available at all levels of the health system in Mali, DRC, Uganda, and Ghana, integrated into the HIV screening program in Burkina Faso and Uganda, and integrated into the reproductive, maternal, newborn and child health programs in six countries (DRC, Gabon, Ghana, Guinea, Tanzania, and Uganda). These linkages are all intended to improve the early detection and management of SCD [1469].

Multi-national NBS activities have been reported recently in at least three separate studies. A 2021 report described a U.S. research team’s feasibility assessment for adapting a computer-based SCD education program, CHOICES, for use in Ghana and Nigeria, with three major recommendations: (1) material must be culturally appropriate; (2) community healthcare workers must be involved in delivering the CHOICES program; and (3) religious and community leaders and elders must be partners in a public awareness campaign [1470]. A digital App has been used in Ghana to store and transmit NBS results since 2017 [1455] and is now the subject of a collaboration between ASH and a commercial company for distribution to the six other CONSA countries. The App has been successful even when there is limited internet connection and is allowing babies in Ghana to receive earlier clinical referrals and care [1471].

To determine enablers and barriers to the implementation of NBS programs for SCD, the NBS programs in six SSA countries (Angola, DRC, Ghana, Liberia, Nigeria, and Tanzania) were systematically assessed using established qualitative research methods This study found that the most important enablers were strong political will, financial support, and the availability of trained personnel. The most important barriers were the high cost of screening, the lack of awareness of SCD, and the stigma associated with the disease. The analysis suggested that developing programs in SSA should prioritize government partner participation and funding from the earliest stages of program development [1472].

A 2021 article describing the implementation of NBS at Korle Bu Teaching Hospital, Accra, Ghana [1473] was described in an accompanying editorial comment as, “*… one of the most detailed accounts yet published of the programmatic structure, resources, personnel, and efforts needed to operationalize an LMIC newborn SCD screening program in its full breadth from sample collection to follow-up care*” [1468]. With this and other published models, NBS programs should continue to develop in SSA. A recent comprehensive literature review assessing the applicability of traditional and genomic NBS in Africa, unsurprisingly, found limited data on the involvement of Africa in genomic NBS (gNBS). Overall, the authors concluded that their findings demonstrated the unpreparedness of both developed and under-resourced settings in SSA to implement full genomic NBS and noted their future intention to “*…develop a gNBS workflow or pipeline that will be Africa-specific*” [1474].

Recently, at the WHO’s 72nd Regional Committee for Africa meeting (22–26 August 2022), the SSA health ministers appeared united in their efforts to ramp up awareness, and bolster prevention and care in order to curb the tolls from SCD [1475]. Hopefully, they are aware of the promise of NBS in SCD identification and case management and will work to assist NBS implementation and sustainability efforts across the continent. A recent analysis of the prevalence and mortality burden of SCD, 2000–2021, from the 2021 Global Burden of Disease Study, noted that SCD has increased by almost 14% globally between 2000 and 2021, and 27% in SSA primarily due to population growth. Six countries, Benin, Burkina Faso, Equatorial Guinea, Nigeria, Sierra Leone, and Togo, accounted for 44% of the global births with SCD in 2021, with 79% of the SCD births globally were accounted for by the larger SSA (up from 70% in 2000). Deaths from SCD were up 65% from 2000 to 2021 in SSA and SCD ranks high as a contributor to deaths in children under age 5. This report strongly urges NBS inclusion in all national healthcare plans in SSA, particularly as a strategy for meeting WHO Sustainable Development Goals [1444].

While assistance from pharma companies is not often referenced in the literature, the generous contributions of equipment and expertise in furthering laboratory science across SSA from both Novartis AG (Switzerland) and Revvity (Massachusetts) previously affiliated with Perkin Elmer, have been instrumental in program implementation in several SSA countries. Both companies are partnering in the “Norvartis Africa Sickle Cell Disease program” (https://www.novartis.com/diseases/sickle-cell-disease), which aims to expand advocacy efforts to educate patients, caregivers, and communities about the importance of NBS and early intervention with hydroxyurea and other SCD treatments. These collaborative programs are working with governments and other partners to improve diagnosis, strengthen healthcare systems, and provide access to treatment safely and sustainably. Additionally, the Fondation Pierre Fabre has provided immense support to the French speaking countries of SSA. In March 2019, the Fondation hosted a meeting entitled “Initiative Drépanocytose Afrique” (African SCD Initiative) in Paris to discuss policy directions and potential regional strategies in 11 African countries: Burkina Faso, Burundi, Cameroon, Central African Republic, Kenya, Madagascar, Mali, Nigeria, Democratic Republic of Congo, Senegal, and Togo. The intent is to have SCD made part of global health programs to help SSA countries meet the U.N. Sustainable Development Goal 3.2: “*By 2030, end preventable deaths of newborns and children under 5 years of age, with all countries aiming to reduce neonatal mortality to at least as low as 12 per 1000 live births and under-5 mortality to at least as low as 25 per 1000 live births*” [1476].

#### 3.6.2. NBS Activities with a Country Focus

In the following section, recent publications from various countries are reviewed, with emphasis on more recent activities, to provide information on the status of NBS and relevant studies.

- **Angola**—Expanding on the success of a SCD clinic established in 2005 in Luanda, a pilot NBS program focused on sickle cell anemia (SCA) was begun in 2011 in five birthing centers in Luanda. The program was a partnership between the Angolan Ministry of Health, Chevron Corporation, and Baylor College of Medicine and demonstrated that NBS was feasible in a developing country such as Angola. Follow-up was challenging but successful in the majority of cases [1477]. A cost-effectiveness study using data from the pilot program demonstrated that NBS and early treatment would be cost effective across all scenarios by WHO criteria [1478]. The program was continued and expanded to 11 health centers in Luanda and 11 in Cabinda province but was not present in the other 16 provinces [1469]. The funding and project completed in 2020 and in 2022, the project was reported to be on hold while the government considered funding options [1470]. While the pilot utilized IEF as the laboratory screening technique, its use outside of the major population centers continues to be recognized as a challenge due to its relative technical complexity and high cost, among other considerations. As a result, POC tests are still in development as noted previously. One such study demonstrated the feasibility and diagnostic accuracy of a paper-based assay in a resource-limited clinical setting in Cabinda, Angola [1479]. A 2023 report notes that a pilot was begun in June 2023 at the Hospital Materno Infantil Dr. Manuel Pedro Azancot de Menezes in Luanda, a referral of the main maternity facility, to be used as a model for other hospitals. Specimens are sent to Lisbon, Portugal for laboratory testing as part of the ARISE project [1480].
- **Benin**—Pilot NBS focused on SCA began in 1993 with initial funding from the CAMPUS Program of the French Ministry and the European Union and later as a partnership between the Beninese Ministry of Health, the March of Dimes, and the University of Benin. The program used repeated information and education of pregnant women as a way of getting them to request NBS [1446]. The pilot program was successful and has slowly expanded through the years. A study of hemoglobin abnormalities in Benin highlighted the need for NBS to enhance early disease detection, prevention, and comprehensive care [1481].
- **Burkina Faso**—The first NBS pilot study was conducted in Ouagadougou in 2004, and the incidence of SCD in Burkina Faso was estimated to be 1 in 57 births [1482]. In 2014, Fondation Pierre Fabre signed a partnership with the Comité d’Initiative contre la Drépanocytose au Burkina (SCD Initiative Committee in Burkina), a non-profit organization, for a 3-year project to raise awareness of SCD, to improve access to care, and to offer systematic neonatal diagnosis. The overall goal was to reduce morbidity and mortality from SCD [1483]. A more recent study between 2015 and 2019 focused on NBS in Ouagadougou and Bobo-Dioulasso, another ethnic area of Burkina Faso, finding a SCD incidence of 1 in 53 births [1484]. Both studies strongly encouraged the development of a national NBS program for SCD.
- **Burundi**—Aside from a study of a new enzyme-linked immunosorbent assay test conducted in the Great Lakes region in Central Africa from July 2004 until July 2006, there is little information available about NBS in Burundi. In the reported study specimens from Kigali and Butare in Rwanda, Bujumbura in Burundi, and Goma in the East of the Democratic Republic of Congo were analyzed, and the technique found usable for NBS for SCD [1485].
- **Cameroon**—There are about 20 million inhabitants of Cameroon with a SCD carrier frequency of 8 to 34%, and there are no specialized centers for lifelong medical care of those affected. A 2015 report noted the urgent need to develop and implement policy actions in Cameroon on at least five levels including policies and practices that improve the early detection and care of SCD, e.g., neonatal screening [1486]. A 2018 report documented the use of NBS filter cards in determining the normal range for TSH in newborns at Yaounde Gyneco-Obstetric and Paediatric Hospital. This small pilot (*n* = 180) did not detect any cases of CH and concluded that a larger study was necessary to determine the potential for NBS in the country [1487]. And a 2020 article on knowledge, attitudes, and practices towards SCD in unmarried youths recommended NBS for SCD as a needed preventive method [1488]. The Fondation Pierre Fabre and partners from Cameroon and three Central African countries (Côte d’Ivoire, Democratic Republic of Congo, Central African Republic and) have designed a project to support the fight against SCD. This multi-country project, headed and coordinated by the Fondation Pierre Fabre and co-financed by the Agence Française de Dévelopement, intends to address the challenge of reducing morbidity and mortality linked to SCD by acting on several levels. One of these is to make NBS available free of charge at more than 50 healthcare structures partnering with the project. Several screening strategies and techniques are being utilized, including POC testing, to maximize the decentralization of access to diagnoses. Other action levels include treating, training, public awareness, and support of public authorities [1489].
- **Central African Republic**—Since 2019, The Fondation Pierre Fabre has provided support for the Sickle Cell Research and Treatment Centre (CRTD), a national referral center founded in 2018 at the initiative of the Central African Republic Ministry of Health and Population. The CRTD is located on the grounds of Bangui’s National University Hospital Centre and specializes in SCD treatment [1490]. The Fondation Pierre Fabre has also partnered with the Central African Republic and three Central African countries (Côte d’Ivoire, Democratic Republic of Congo, and Cameroon) have designed a project to support the fight against SCD. See a project description under Cameroon [1489]. In September 2022, the “drep.ACCI“ project was launched in Bangui, under the patronage of the First Lady of the Central African Republic. This project is a partnership between the Pasteur Institute of Bangui, the Fondation Pierre Fabre and the AFD—Agence Française de Dévelopement, which is co-financing the project, and is aimed at introducing systematic NBS, using POC tests, in three maternity wards in Bangui (40,000 newborns), as part of the national strategy to combat SCD [1491].
- **Comoros**—The Comoros islands consist of four volcanic islands to the west of the northern tip of Madagascar and is one of the poorest and least developed countries in the region. Since gaining independence from France in 1975 (except for one island, Mayotte, which remains as a French Department), the country has been characterized by political instability which has adversely affected the health of the population. A comprehensive look at the healthcare issues was published in 2021 [1492]. A relatively high incidence of HGBs and G6PD deficiency has been reported [1493]. To the best of our knowledge, there is no ongoing NBS activity.
- **Congo, Democratic Republic**—The Democratic Republic of the Congo (DRC), formerly Zaire, is the second-largest country in Africa, after Algeria, with a population of about 100 million. It is a land-locked country bordered by nine other countries and home to over 260 different tribes distinguished into four main groups: Lingala, Luba, Swahili and Kongo. The DRC has made some progress in terms of peace and stability; however, there are still many challenges, including poverty, corruption, and the ongoing conflict in the east of the country. A 2009 report documents the introduction of NBS in the DRC in 2006 and notes the main challenges are tracking new cases for confirmatory testing and early management [1494]. A 2022 study of NBS for SCD in Lubumbashi City found that NBS was widely accepted and the primary challenges to its adoption were likely to be financial and practical rather than social or cultural [1495]. A 2023 report on NBS in Butembo and Beni cities found that while the prevalence of SCD was lower compared to other regions of the country, it was still an alarming public health issue, particularly in view of the lack of SCD policies and priorities. The authors urged decision makers at national and global level to implement systematic NBS, patient/carrier education and early management programs, integrated into the national health system [1496].
  While some NBS is ongoing in Kinshasa, the capital city, its implementation in the rural and remote areas is extremely difficult for several reasons. One of these centers on the laboratory methodology required for NBS, which is relatively expensive and complex. For this reason, POC tests (both SickleSCAN^®^ and HemoTypeSC^TM^) have been evaluated in different DRC locations with good results: a 2-month study in 2018 of 310 neonates in 9 maternity homes in the city of Kindu by SickleSCAN^®^ [1497]; a 2-month study of 87 newborns in Kisangani city by HemoTypeSC^TM^ [1498]; testing the parents of 58 newborns screened with HemoTypeSC^TM^ in 8 maternity centers in Kisangani city in 2019 to validate the screening results [1499]; a 10-month study of 448 newborns in Kindu using HemoTypeSC^TM^ during 2019/2020 [1500]; and a 2-year study of 1432 newborns in 12 maternities using HemoTypeSC^TM^ to update the prevalence of SCD (SCA = 2.2%; twice as high as in 2010) as part of a doctoral research study [1501]. A 2022 overview of the current progress and challenges in diagnosis, and management of pediatric SCD in the DRC also noted the use of POC as a possible solution to NBS in outlying areas [1502].
  The Fondation Pierre Fabre and its partners from DRC and three other Central African countries (Côte d’Ivoire, Central African Republic, and Cameroon) have designed a project to support the fight against SCD. See project description under Cameroon [1489].

- **Congo, Republic**—The Republic of Congo is made up of 12 departments and lacks a systematic NBS program. A descriptive cross-sectional study was conducted from 1 October 2019 to 31 March 2020 throughout the Congolese national territory, to update Congolese hemoglobinopathy epidemiological data. It used a central NBS laboratory, Centre National de Référence de la drepanocytose (CNRDr), for HPLC screening. In addition to documenting the prevalence of HbS and other hemoglobins, the study concluded that implementation of a national systematic NBS program is needed to identify and provide early care for newborns with SCA, thus providing a better quality of life by limiting infectious and vaso-occlusive complications [1503].
- **Cote d’Ivoire**—A 2021 report provides evidence to support the use of the HemoTypeSC^TM^ test for the rapid screening of SCD in Côte d’Ivoire. The test was accurate and easy to use, making it a promising option for improving the early diagnosis and treatment of SCD in the country [1504]. The Fondation Pierre Fabre and its partners from Côte d’Ivoire and three Central African countries (Democratic Republic of Congo, Central African Republic, and Cameroon) have designed a project to support the fight against SCD. See a project description under Cameroon [1489].
- **Equatorial Guinea**—Equatorial Guinea consists of two parts, two small islands and a small mainland region. Bioko Island is the northernmost part and is the site of the country’s capital. A formal NBS program does not yet exist in Equatorial Guinea. An epidemiologic study reported in 2015 confirmed the high prevalence of G6PDD and hemoglobinopathies on Bioko Island and noted that obligatory NBS, prenatal screening, and counseling for these conditions, especially HbS, were needed on the island [1505].
- **Eswatini**—HIV testing at birth of HIV-exposed infants using polymerase chain reaction (PCR) screening may improve the identification of infants infected with HIV in utero and accelerate antiretroviral treatment (ART) initiation. A pilot program was initiated in two maternity facilities to demonstrate the feasibility of this approach in a low-resource and high-burden setting. While high uptake of testing was documented, few newborns were found to be infected [1506]. A national NBS program is not currently in place. While HIV screening is not generally thought of as NBS, it is still included as one of the screened disorders in the New York NBS program.
- **Ethiopia**—While there is currently no organized NBS in Ethiopia, there is increasing interest in organizing a pilot and at least one group of university researchers is beginning the process of evaluating condition(s) to be included in a screening program and seeking funding [1507]. Only a single study related to NBS is available assessing the possibility of NBS for CH in Addis Ababa in the 1990s. This study looked at 4206 newborns without identifying a case of CH and concluded that larger studies were needed [1508]. No further studies are known.
- **Gabon**—There is currently limited NBS for SCD in Gabon. Screening is sometimes carried out only at the request of the mother and a recent study in rural Eastern Gabon found that about 30% of newborns obtained NBS for SCD. The barriers to acceptance of NBS for SCD were more related to habits and customs than to knowledge of the disease. For example, blood sampling at 3–4 days of age was thought to be unrealistic in an environment where traditions confine the newborn during a lunar cycle (1 month) before it is presented to the community. Establishment of rural NBS for SCD will likely require a team composed of a psychosocial and health worker familiar with rural communities, to address inhibitions related to neonatal blood collection [1509]. A 2022 report reviewed data obtained from 6 of the 9 provinces in Gabon as part of a national pilot study of NBS for SCD between January 2007 and September 2010. Results indicated a high prevalence of SCD, likely underreported since study participation required parental consent, and led the authors to conclude that compulsory NBS was needed in countries with a high incidence of malaria, like Gabon [1510].
- **Ghana**—The NBS program for SCD in Ghana started in 1993 limited to 2 of the 16 health regions—Greater Accra and Ashanti. Significant efforts and sacrifice have contributed to the implementation and expansion of NBS since the 1990s in Ghana and other SSA countries under the leadership of the late Dr. Kwaku Ohene-Frempong [1511]. Recently, at least two POC screening tests for SCD have been evaluated for possible use in Ghana, (see POC discussion in Section 3.6.1): a multicenter (low-, medium- and high-resource environments—Ghana, Martinique, and USA) evaluation of HemoTypeSC^TM^ [1512]; and a prospective diagnostic accuracy study of paper-based microchip electrophoresis using a multispectral platform [1513].
  Despite the government’s acceptance NBS for SCD as a project for national expansion in 2010, efforts to provide more trained genetic counselors through a SCD genetic counseling training and certification program, and the creation of a mobile application (App) to enhance program efficiency, NBS is still confined to the larger cities of Kumasi and Accra, and a limited number of rural facilities [1457]. The value of the Ghana App has been recognized by ASH, through its CONSA project and, in partnership with Novartis AG (Switzerland), is providing it to the six other CONSA members: Kenya, Liberia, Nigeria, Uganda, Tanzania, and Zambia. The App allows NBS follow-up coordinators to track patients and migrate them to further care. It is being used to collect and store screening results and medical histories for diagnosed newborns (and others) and allows for offline data collection when internet connections are down, and then syncs the data once the connection is restored [1470].
  The lack of a robust NBS program for SCD in Ghana, and elsewhere in SSA, led to a study of SCD diagnosis patterns at Korle Bu Teaching Hospital (KBTH), the third largest hospital in Africa and a major Ghanaian referral center, between 2009 and 2013, which indicated that a SCD diagnosis was usually made at the first pain crisis. This late diagnosis excludes many patients from life-saving preventive care and argues for a more robust NBS program [1514]. After determining the feasibility and challenges of implementing NBS for SCD at KBTH and relying on a multi-year partnership with SickKids Center for Global Child Health, Toronto, and Pfizer corporation, a comprehensive NBS program was implemented in progressive phases to best overcome the various challenges of sustainability [1472,1515]. This has the potential to provide a model for other hospitals in SSA.
  Beginning in 2021, the Clinton Health Access Initiative (CHAI), the Gates Foundation, the Ministry of Health, the Ghana Health Service, the Sickle Cell Foundation of Ghana, and the National Newborn Screening Laboratory at Noguchi Memorial Institute for Medical Research have partnered to begin sustainable NBS expansion. To date, NBS is reaching 12 health regions with a focus on closing the gaps in care for SCD, including developing a clear and centrally coordinated pathway to comprehensive NBS, diagnosis, and treatment [1516]. In September of 2023, the American Society of Hematology, in collaboration with Revvity, organized an international, in-person laboratory training summit at the Noguchi Memorial Institute in Accra, Ghana. Representatives from seven SSA CONSA countries came together to learn, share, and develop laboratory skills with Migele^®^ IEF NBS hemoglobinopathy techniques, procedures, and interpretations.

- **Guinea**—NBS does not yet exist in Guinea despite a 2012 epidemiological study that assessed the frequency of G6PDD and hemoglobinopathies and concluded that the development of NBS was needed and would help patients benefit from early diagnosis and treatment [1517]. A recent Memorandum of Understanding between the Novo Nordisk Haemophilia Foundation (NNHF) and the Fondation Pierre Fabre to jointly improve awareness, diagnosis, care, and advocacy for blood disorders in Guinea is intended to leverage synergies for a joint approach to hemophilia and SCD at the request of Guinea’s MOH. The Programme Manager, Fondation Pierre Fabre, was quoted as saying, “*We want to encourage greater screening of newborns…*” Implementation of the project is beginning in 2023 [1518].
- **Guinea-Bissau**—There is currently no organized NBS in Guinea-Bissau. A 2018 report summarized an evaluation study of the feasibility of simultaneously screening for HIV, SCD and TB in a rural area of the country using POC tests for HIV and SCD and a standardized clinical questionnaire with clinical examination for TB. This pilot study demonstrated the feasibility of this approach in rural areas as a model that could be replicated in other rural settings in low-resource countries [1519]. A 2022 article describes an epidemiologic study in two hospitals managed within the National Health System of Guinea Bissau by Italian NGOs. In a collaborative effort, newborn DBS specimens were sent to Padova for Hb quantification and molecular analysis, and Hb haplotypes of the HbSS and HbSA patients were obtained in South Africa. Higher prevalence of the HbS allele in a pediatric population compared to previous studies confirms the need to develop nationwide SCD screening and comprehensive care programs in the country, perhaps building on the POC model referenced above. A high prevalence of G6PDD was also confirmed [1520].
- **Kenya**—A 2019 report provided data on a NBS pilot in Kisumu Country, Kenya. Specimens were collected by heel prick onto filter paper and analyzed by IEF in the AMPATH (Academic Model for Providing Access to Health Care) reference laboratories at the Moi Teaching and Referral Hospital, Eldoret. High prevalence rates of SCD and SCT were confirmed along with high acceptability of NBS for SCD. These data were important for informing the development and implementation of NBS in Kenya. The study concluded that NBS for SCD was a valuable and needed public health tool for early identification and management of SCD [1521]. A 2020 report also described the critical role played by NBS program for SCD in Western Kenya. This report noted that NBS for SCD was established in 2012 with support from the Indiana Hemophilia and Thrombosis Center (IHTC) who trained laboratory personnel in screening techniques. The use of a POC test was suggested for consideration as screening policies are developed nationally [1522].
  Through partnerships with ASH, Revvity, and CONSA, and building on the 2019 pilot in Kisumu noted above, a SCD screening center was opened in December 2021. A screening package that included screening equipment to begin NBS was included, and services were intended to serve 17 other counties with high burdens of SCD [1453,1523]. As a CONSA member, Kenya is a recipient of the Ghana App for follow-up and case management (see discussion under Ghana) [1453,1470]. In Kenya, NBS is commercially available and includes 14 other screening conditions in addition to SCD [1524]. Genetic testing for 106 conditions is also commercially available [1525]. Also in Kisumu, researchers at the Kenya Medical Research Institute, Center for Global Health Research, have partnered with researchers in Canada, Bangladesh, Zambia, and Zimbabwe to validate an algorithm that derives gestational age estimates from dried bloodspot samples using metabolic data. Results from this research have the potential to assess gestational age at birth in LMIC countries where reliable estimates may otherwise be unavailable [164].

- **Liberia**—Updating the epidemiology of SCD in the area is always necessary for a new program such as NBS for SCD. Thus, the NBS pilot program begun in 2013 had two main objectives: to determine the feasibility of NBS in Monrovia, the capital city, and to define the incidence of various abnormal hemoglobins in this post-war, post-migration city [1526]. While the pilot data supported the need for NBS, the Ebola Virus outbreak in 2014 forced the pilot program to pause. In 2021, the Liberian MOH partnered with CONSA to initiate NBS as part of the seven-country CONSA coalition (see Section 3.6.1) and is part of the user community for the Ghana App for NBS follow-up [1453,1470,1527,1528].
- **Madagascar**—While representatives from Madagascar were present for the signing of the joint statement from attendees at the Paris meeting “Initiative Drépanocytose Afrique” (African Sickle-Cell disease Initiative), hosted by the Fondation Pierre Fabre in 2019, there has been little movement towards a national NBS program (see Section 3.6.1) [1476].
- **Malawi**—Malawi has a well-established regionalized HIV Early Infant Diagnosis program that uses DBS specimens collected within 6 weeks of birth. The first regional SCD surveillance study in Malawi used leftover HIV screening specimens to estimate the prevalence of SCD and SCT across Central Malawi where 43% of the population resides. This study, reported in 2021, was a collaboration between the Malawi MOH, the University of North Carolina Project-Malawi community tracing team and project laboratories, the Kamuzu Central Hospital, Mzimba District Hospital, Partners in Hope, and UNC Project laboratories, Cincinnati Children’s Hospital Medical Center (CCHMC) and Revvity, and showed that inherited hematological disorders, including G6PDD are common in Central Malawi and that early identification through NBS improves clinical outcomes and should be supported [1529].
- **Mali**—Traditional NBS methods for SCD have been limited due to cost, lack of laboratory equipment and trained personnel, and an unreliable supply chain. Studies of POC testing have shown promise as a way of initiating a broad NBS program for SCD. The HemoTypeSC^TM^ POC test kit, which only requires capillary blood samples and unfiltered tap water, was tested over a 2-year period in Koutiala hospital in rural Mali and found to be satisfactory for NBS [1530]. Similarly, the acceptability and diagnosis performances of two POC tests (SickleScan^®^ and H HemoTypeSC^TM^) were evaluated against HPLC methodology in three maternity hospitals in Mali (two urban clinics in Bamako and one rural clinic in Kayes) using cord blood, and both tests were found to be acceptable NBS tests [1531]. A study of the Emmel test routinely used to test adults for SCA was found to be severely lacking when used for screening newborns in Bamako, and its use was NOT recommended despite its low cost and relative simplicity [1532]. A Research Centre to combat SCD (CRLD) was opened in Bamako in 2010, supported by the Fondation Pierre Fabre. The center’s mission was “*to screen for the disease, receive and hospitalise patients, conduct training and information campaigns, but also to perform clinical research*” [1533]. Despite these efforts, there is not yet an organized national NBS program for SCD in Mali.
- **Mauritania**—Mauritania is an extremely poor country with a developing healthcare system. Previous studies have confirmed the presence of both G6PD deficiency and HGBs and have supported the concept of NBS as a means of decreasing infant morbidity and mortality [1534,1535,1536]. We are aware of no current efforts to introduce a national NBS program.
- **Mauritius**—NBS testing has been available in Mauritius since 2019. The test panel includes CF, CH, CAH, PKU and HGB. Screening is performed at C-Lab, Wellkin Hospital, Reduit, Moka. Though screening coverage is still low, 100% of babies born in Wellkin hospital are screened and some samples from other hospitals are sent to Wellkin based on recommendation of the pediatrician. Presently there is no government involvement.
- **Namibia**—A preliminary report describes pilot testing at Rundu Intermediate hospital in Kavango East Region using the HemoTypeSC^TM^ POC test to determine the prevalence of SCD. Between February and March 2023, slightly more than 200 newborns were screened with the results intended to inform policy on NBS [1537].
- **Nigeria**—With almost 220 million people, Nigeria is Africa’s most populous country, with one of the highest birth rates in the world, and may become the third most populous country globally by 2050.There is currently no national NBS program in Nigeria; however, there have been several pilot studies in recent years to assess the feasibility and acceptability of NBS for both SCD and CH. A 2018 report on the acceptability of NBS across various attitudes to, and acceptability of, NBS in Nigeria among various socio-demographic groups (including health professionals, undergraduate students, parents of children with SCD, and SCD patients) found good acceptability of NBS with main barriers likely to be financial and practical, rather than social or cultural [1538]. A 2019 report provided a comprehensive assessment of the current burden of SCD in Nigeria with the aim of identifying surveillance and treatment gaps. Surveillance using NBS was noted as a necessity for early detection and management to improve SCD survival [1539]. Also in 2019, NBS for CH was suggested in combination with NBS for SCD, but no serious effort to screen for CH has yet appeared [1451], but no serious effort to screen for CH has yet appeared, although at least one recent report provided information on normative values of cord blood TSH in Nigerian newborns preparatory to developing NBS [1540].
  A number of recent reports have addressed acceptability and feasibility of NBS for SCD in different areas of the country: validation of the use of IEF as a screening tool in Awka, Southeast Nigeria [1541]; affirmation of the feasibility and acceptability of a SCD screening intervention program in Lagos State using the HemoTypeSC^TM^ POC for ease and reduced cost [1542]; evaluation of HPLC as a suitable NBS method for HGB at the National Hospital Abuja [1543]; documentation of the feasibility and acceptability of integrating HGB screening into an existing community-based maternal–child HIV program to increase awareness and demand for NBS from pregnant women in Benue State, Nigeria [1544]; acceptance of NBS based on awareness, education and cultural beliefs with a preference for screening away from the birthing site in Ibadan, Nigeria [1545]; and improvement of NBS program over time in Kaduna State, Nigeria [1546].
  The use of POC testing has resulted in studies, including the report from Lagos State mentioned above on HemoTypeSC^TM^ [1542], on their reliability and effectiveness in improving the speed of analysis and impact on acceptability on parents. At least two studies looked at integrating POC NBS into immunization clinic protocols. One addressed the feasibility of eluting blood from DBS for screening using HemoTypeSC^TM^ (to avoid the cumbersome practice of using blood collected in capillary tubes) at immunization clinics in 6 Primary Health Centers in Abuja, Nigeria [1547], and another looked at the feasibility of both HemoTypeSC^TM^ and SickleSCAN^®^ versus HPLC in five immunization clinics in Abuja [1467].
  Nigeria is part of CONSA, and as such, has formally implemented NBS with the goal of becoming both sustainable and nationally available [1454]. The first babies with SCD identified through the CONSA program were in the Angwan Dodo Primary Health Care Centre in the University of Abuja Teaching Hospital in December 2020, with three more detected by mid-April 2021, via the CONSA Abuja Network. Since then, other babies have also been identified in the CONSA Kaduna location [1548]. Reports from NBS pilots in various locations continue including 2023 reports from Bida, North-Central Nigeria (attendees at routine immunization clinics) [1549], and Abakaliki, South-East Nigeria (post-partum mothers) [1550]. As a member nation of CONSA, the App developed for follow-up should soon be available [1469].

- **Rwanda**—Despite documenting that SCD should be considered a public health problem in Kigali and Butare in 2006 and that a systematic NBS for those disorders and for G6PDD is deemed reasonable, a systematic national NBS program has not yet been implemented in Rwanda [1551].
- **Senegal**—A pilot NBS program for SCD was first reported in 2003 [1552], leading Senegal to become one of the first countries in Africa to implement a national NBS program for SCD. More recently, focus has been on development and implementation of a health information system for data management of the blood sampling from newborns at the maternity wards and disease screening at the Center for Research and Ambulatory Care of SCD (CERPAD) [1553,1554,1555]. Significant support has come from the Fondation Pierre Fabre through their “Operational study on early detection and treatment of SCD” [1556].
- **Seychelles**—The health system in Seychelles was recently reviewed. Significant health system development has occurred over the past three decades, largely because of investments in both the health services and other social sectors that directly impact child survival and the health of individuals. There is an understanding that public services need to use their resources to develop stronger and more sustained initiatives, in partnership with all relevant stakeholders [1557]. As an example, NBS for six conditions (PKU, CH, CAH, CF, HGB, GAL) was implemented in mid-February 2021, made possible by a donation from multiple stakeholders. Additionally, acquisition of MS/MS equipment will allow NBS expansion in the future [1558].
- **Sierra Leone**—There is currently no NBS in Sierra Leone but at least one stakeholder group, The Africa Sickle Cell Center for Education and Research (ASCC4ER), called on the Sierra Leone President, through the Minister of Health and Sanitation on World Sickle Cell Day (19 June 2023), to develop a policy on NBS for SCD in Sierra Leone. Additionally, ASCC4ER designated the theme for their 2023 Sickle Cell Day commemoration, “Why newborn screening should be a priority in Sierra Leone on sickle cell disorder?” [1559]. Interest in NBS is spreading in Sierra Leone as evidenced by a comment published by collaborators at the University of Sierra Leone, Freetown, concerning the value of carefully considering how NBS results are communicated [1560]. Additional information prior to 2020 is available [1457].
- **South Africa**—In the 1960s (1964–1967), a urine NBS program was conducted in Johannesburg with no cases detected. A pilot NBS program in Pretoria from 1979–1981 for “amino acidaemias and CH” identified no cases but based on clinical detection of PKU and CH, a second pilot was conducted from 1981–1986 (in mostly White newborns) with sufficient case findings of CH to warrant a recommendation for “*…a nationwide unified screening program…based on 5–6 regional screening laboratories*” [1561,1562]. Delegates to the first national NBS workshop in Cape Town in 1987 concluded that data were insufficient to justify national NBS. A second workshop (1992) under the joint auspices of the National Pathologists Group of the Medical Association of South Africa and Genetic Services of the Department of National Health and Population Development was held at the Johannesburg airport to review NBS progress and to exchange views and consider recommendations on NBS for CH. Aside from agreeing that there were insufficient screening data on Blacks, there was no consensus on the need for screening and additional workshops were scheduled [1563]. A 2008 report on NBS for CH and GAL in the Nkangala district of the Mpumalanga province noted the continuing lack of national prevalence data. These investigators found that “*a screening programme for both disorders (CH and GAL) that integrates disease identification and treatment will be highly cost effective*” [1564].
  Currently, despite the previous studies and continual education of health professionals and the public, there is no national plan for NBS. The HIV and concomitant tuberculosis epidemics that began in the early 2000s resulted in the redirection of both financial and human resources to these infectious diseases to the detriment of genetic services [1565]. NBS is currently only available to less than 5% of births in South Africa, specifically for CH, through a limited number of government-supported facilities in parts of Cape Town, with more comprehensive screening tests offered privately. Testing for CH and MS/MS testing for other IEMs is commercially available with variable pricing “*depending on which pathology laboratory the testing is done through*” [1566], and are paid out-of-pocket by the patient (through medical savings), even if the patient pays for private medical insurance. The NBS laboratory at North-West University (Centre for Human Metabolomics) has provided testing services for many years with screening services available nationally [1567,1568]. Interestingly, despite the lack of a national NBS program, South Africa is listed as one of the first African countries to explore use of genomics in the diagnosis of singular congenital disorders [1451,1474]. Today, efforts continue to develop an organized, sustainable NBS program in the national public health setting. At a national NBS meeting in February 2023, plans were discussed to potentially expand CH NBS across the country and a NBS demonstration project for CH in two contrasting provinces is under development, at the request of the National Department of Health, to assist in decision making [1569].

- **Sudan**—A 2012 overview of the diagnosis, management, and outcome of CH in Sudan was intended to “*shed a light on this important health problem”* and to ask for *“help establishing a national screening program*”. Moreover, it noted that the cost of long-term treatment of a child with neurodevelopmental delay exceeds the cost of screening and would be cost beneficial in the long run [1570]. A 2016 report on SCD epidemiology in West Kordofan, Sudan provided evidence that the documented HbS allele frequency is one of the highest in the world. Furthermore, the knowledge, attitude and practices towards the disease are unsatisfactory, resulting in the strong recommendation to develop public health programs to control and manage SCD in the western parts of Sudan [1571]. Until now, to our knowledge, a national NBS program does not exist.
- **Tanzania**—Tanzania in East Africa has a population of over 65 million people with the fifth highest prevalence of SCD in the world estimated at 11,000 new cases (babies) annually. From January 2015 to November 2016, a pilot NBS program for SCD was initiated at Muhimbili National Hospital and Temeke Regional Hospital in Dar-Es-Salaam. The pilot was coordinated by Muhimbili University of Health and Allied Sciences in their hematology research laboratory using IEF. This was the first report on NBS as a health program for SCD in Tanzania and strongly supported the concept of NBS [1572]. A 2020 follow-up report reviewed the past, present, and future of NBS for SCD in Tanzania cautioning that, “*The successful introduction and expansion of NBS in Tanzania will require careful planning and advocacy at community to national level*” [1573]. To aid in implementation, Tanzania is a member of CONSA (see Section 3.6.1) and is one of the countries receiving the follow-up cell phone App developed in Ghana [1453,1470].
  Because prevalence data on SCD in Tanzania were sparse and outdated, a surveillance study was performed on residual specimens from the HIV Early Infant Diagnosis program in northwestern Tanzania from February 2017 to May 2018. This was a collaborative effort with Cincinnati Children’s Hospital Medical Center (CCHMC), USA, and found a prevalence rate twice as high as previously reported (SCD ~1.2%), reinforcing the need for NBS and expanded diagnostic services [1574]. High prevalence results (SCD ~4%) were also obtained at a hospital in Shirati (rural northern Tanzania) in a separate study undertaken from February to September 2019. In addition to birth prevalence data, this study also demonstrated the value of POC testing (HemoTypeSC^TM^) and the need to improve community awareness [1575].
  A 2021 report reviewed NBS activities related to SCD in Tanzania and analyzed the experiences and perspectives of policy makers, healthcare providers, and families on expanding and sustaining NBS for SCD and related comprehensive care services. The opportunities and areas that need to be addressed for proper implementation and sustainability of NBS for SCD in a low-resource setting were identified. Sustainability efforts at the local level were encouraging and could be a useful model for other low-resource programs [1576]. A 2022 report of a study of POC tests (HemoTypeSC^TM^ and SickleSCAN^®^) showed reliability of either versus IEF, and both were found to be acceptable for screening [1577]. A 2023 report of a study at three hospitals in Dar-Es-Salaam reported that maternal health education and maternal screening for SCD were feasible and efficacious interventions to increase the knowledge and uptake of NBS, and these interventions were strongly recommended for inclusion in comprehensive care packages for pregnant women attending antenatal clinics [1578].
  Tanzania NBS projects have also collaborated with North American studies focused on the machine learning prediction of gestational age using metabolic screening markers resistant to ambient temperature during transport. These “*Machine learning models applied to metabolomic gestational age dating offer a ladder of opportunity for providing accurate population-level gestational age estimates in LMICs settings*” [441,1579,1580].

- **Togo**—While representatives from Togo were present for the signing of the joint statement from attendees at the Paris meeting “Initiative Drépanocytose Afrique” (African Sickle-Cell disease Initiative) hosted by the Fondation Pierre Fabre in 2019, there has been little movement towards a national NBS program (see Section 3.6.1) [1476].
- **Uganda**—Design and operation of the Uganda Sickle Surveillance Study (US3) was the result of partnership between the Uganda MOH, Makerere University, and CCHMC, and sought to generate critical data on the prevalence of SCD, SCT, and any comorbidities that might be associated with early mortality in people with SCD. The study period spanned from February 2014 to March 2015 and found evidence for a large sickle burden among HIV-exposed infants and a high prevalence of SCD [1581]. These data resulted in broadening of the NBS program beyond HIV infants. A 3-year (March 2015–April 2018) targeted sickle cell screening project designed by the MOH confirmed a high sickle cell burden and HIV comorbidity, with common genetic modifiers likely influencing laboratory and clinical phenotypes. Further, these data supported the concept of NBS for SCD [1582] and potential improvements by evaluating contributors affecting specimen result turnaround time and determining the associated costs. The NBS program was found to be both efficient and cost-effective with the recommendation that universal NBS using a central laboratory was the best strategy for SCD NBS. A new SCD laboratory outfitted with isoelectric focusing (IEF) equipment was constructed at the Central Public Health Laboratories through a partnership with CCHMC using local personnel recruited and trained on a standardized analytical protocol [1583]. A cost-effectiveness study of POC tests and IEF was also reported in 2019 further informing NBS policy development [1584].
  To examine epidemiological trends and the degree of screening coverage in high-burden districts, data from the US3 and MOH studies collected on 0–24-month-old children between February 2014 and March 2019 were obtained and analyzed. Pertinent tables and graphs were developed and are included in the referenced report [1585]. More recently, Uganda has become a part of the CONSA group of seven countries working together to improve and sustain NBS in each country (see Section 3.6.1) [1453]. An App for follow-up, developed in Ghana, should help in tracking diagnosed cases and ensuring proper long-term care [1470]. Other NBS research in Uganda has included a study of SCD awareness, and feasibility and acceptability of text messaging about screening follow-up. While over 85% of participants had a cell phone, slightly more than half preferred contact by regular telephone, citing concerns about phone access, privacy, or cost, and readability of messages [1586]. Ugandan researchers have also reported studies with gestational age and metabolic profiles. Their “*findings support the notion that NBS metabolic profiles from heel-stick bloodspots can reliably determine gestational age at birth with the additional utility of accurately estimating preterm birth rates*”. This has the potential to improve preterm birth surveillance and epidemiology and may, at some point, help to inform treatment and clinical management shortly after delivery [1587].
  On 24 May 2023, as a side event to the 76th World Health Assembly in Geneva, Switzerland, the World Coalition on Sickle Cell Disease was launched. Dr. Aceng Ocero, Uganda Minister of Health and a pediatrician, embraced the importance of NBS and encouraged other SSA countries to develop sustainable goals.

- **Zambia**—As one of the seven member countries of CONSA (see Section 3.6.1), the Zambia NBS program was launched on 21 April 2021, at Arthur Davison Children’s Hospital in Ndola, Zambia. NBS is integrated into the Expanded Programme on Immunization and HIV Early Infant Diagnosis (HIV-EID). Screening is initially included in three hospitals with laboratory testing at the Tropical Diseases Research Centre laboratory using IEF. As part of CONSA, a SCD electronic follow-up registry of children diagnosed with SCD will be developed that includes patient-specific demographic, medical, and laboratory information [1548].
  In Zambia, as in some other African countries, World Sickle Cell Day on June 19 is an opportunity for recognition, education, and renewed emphasis on NBS for SCD. A 2019 report notes the presence of a less than perfect NBS system in Zambia, but a functioning system, nonetheless. A speech by a person living with SCD at Sickle Cell Day 2019 included a passionate appeal to the MOH for comprehensive healthcare services for persons living with SCD at all healthcare levels and was assured that priority was being given. Technical experts were tasked by the MOH to develop a Zambian Strategy for SCD [1588]. At the 2023 annual Sickle Day celebration in Lusaka, the Zambian Health Minister emphasized the ministry’s commitment to expanding screening services across all provinces. “*The Zambian health department stated that 8000 babies under the age of three months have been screened for SCD following the introduction of NBS services in selected hospitals in Lusaka and Copperbelt Provinces*” [1589].
  Other NBS research has included collaboration with Canadian researchers in the development and external validation of machine learning algorithms for postnatal gestational age estimation. This allows for a more accurate surveillance of the burden of preterm birth in LMIC jurisdictions where data are currently lacking. The current algorithms developed in Canada provided accurate estimates of gestational age when applied to external cohorts from Zambia and Bangladesh with superior model performance from heel prick data compared to cord blood data [166,1590]. Zambia is also a member nation of CONSA and is a recipient of the Ghana App for follow-up and case management (see discussion under Ghana) [1453,1470].

- **Zimbabwe**—Despite the absence of a NBS program, researchers at the University of Zimbabwe have participated with researchers in Kenya, Zambia, Bangladesh, and Canada in validating a protocol for determining gestational age from a metabolic profile [1525]. In low-resource settings, reliable estimates of gestational age are necessary to determine the burden of preterm birth, which in turn informs policies for allocating resources and prioritizing interventions. Currently, there is no organized NBS at the national level.

#### 3.6.3. Summary NBS Information for the SSA Region

Unlike the summaries from other regions, that have focused on the number and variety of conditions for which NBS is being conducted, we have instead included various data regarding births and burden of SCD, since very little NBS is currently implemented in the region. Globally, SSA is a leader in population growth and cases of SCD, a screenable condition. In Table 10, we have provided population and birth data, and birth rates for each country along with their global birth rate ranking showing that the top 12 countries with the highest birth rates are all in SSA. We have listed the global poverty ranking, which shows the top 11 countries are also in SSA. Listings of infant (<1 month old) mortality data and child (<5 years old) mortality data reemphasize the extreme challenges facing SSA as countries continue to transition epidemiologically, improve their healthcare systems, and decrease mortality in children. Because SCD is recognized as a significant contributor to SSA mortality statistics (~30,000 SCD-related deaths in 2021 [1477]), we have included a listing of the approximate number of SCD births in each country in 2000 and 2021 to show the significant increase. For comparison with other programs and to provide a baseline for future reference, we have included indications where NBS is available (although limited). Our table is compressed in this respect since only a few conditions are under discussion in most SSA settings. We have not included HIV screening in the table since operational data are not generally available. In the future, NBS screening may well be integrated with HIV screening to maximize efficiency (already ongoing in some countries).

We were unable to obtain information on 12 countries, and they are, therefore, not listed in Section 3.6.3. These countries are Botswana, Cabo Verde, Chad, Djibouti, Eritrea, Gambia, Lesotho, Mozambique, Niger, Sao Tome and Principe, Somalia, and South Sudan. Additionally, we obtained information on some countries that indicated previous prevalence studies on SCD or interest in SCD screening by organizations seeking to assist the government in developing NBS but with no evidence of a current functioning NBS program of any type: Comoros, Ethiopia, Sierra Leone, Sudan, and Zimbabwe. We anticipate that within a few years, this table can be completed as in the other regions. Current efforts to improve NBS in Africa by organizations (e.g., ISNS, IFCC, ASH, etc.), commercial partners (e.g., Revvity, Norvartis, etc.), and international academic/hospital partners (e.g., CCHMC, Nicklaus Children’s Hospital, etc.) are already filling in the blank spaces and these efforts will continue to overcome the current implementation challenges.

## 4. Discussion

This report builds on, and updates, regional reports published in 2007 [26,27,28,29,30,31] and our global report published in 2015 [16]. The amount of NBS activity that is, and has been, occurring in the past few years is impressive. In this report, we have tried to make available in a centralized location, a summary of recent NBS activities globally. By reviewing activities in the various regions, the reader can compare activities both within and across regions. NBS is a powerful tool for promoting health and well-being and can contribute significantly to meeting the UN Sustainable Development Goals (SDGs) (particularly those focused on health and equity) as part of “Transforming our world: the 2030 Agenda for Sustainable Development” [15]. Most notably, NBS can help to accomplish the targets in SDG 3, “Good Health and Well-being:” SDG Target 3.2—End preventable deaths of newborns and children under 5; and SDG Target 3.4—Reduce non-communicable diseases by one-third through prevention and treatment. By preventing costly lifelong medical interventions and disabilities, NBS also reduces societal and family financial burdens impacting SDG 1: “No poverty.” Further, it impacts SDG 3, “Gender equality” through equitable access to screening regardless of gender or socioeconomic status and SDG 10, “Reduced Inequalities” by ensuring universal screening for all newborns.

It is interesting and satisfying to see the NBS progress made globally since our last report. It remains true that as the IMR reaches 20 deaths per thousand, NBS becomes increasingly important as a public health prevention strategy and health budget priority. Most countries are able to implement a national NBS system for one or more disorders as the IMR approaches single digits. While NBS activities may be initiated in a public, private, or academic healthcare setting, sustainability of the system invariably requires intersection with, and support of, the public health system.

In recent years, NBS expansion in the USA has perhaps provided the greatest screening diversity, but researchers and NBS program experiences globally have provided critical information and actions to refine and expand the capabilities of NBS. In Section 3.4.2 and Section 3.6.2 of this report we have paid particular attention to the progress beginning in the Caribbean and SSA regarding NBS for SCD. While most NBS programs globally have focused on CH as a starting point, the programs in the Caribbean and in SSA instead face challenges with high incidences of SCD and extreme poverty. Initiating NBS for SCD can provide a basic screening infrastructure that could be expanded in the future to encompass other significant congenital conditions.

With Figure 8, we have provided a visual comparison of country land masses adjusted based on population and births to better illustrate the global impact of the increasing birth rate in SSA versus other countries. The combination of increasing birth rates and excessive poverty presents a dire health emergency for which NBS offers increasing hope. By reviewing this report, new screening initiatives can find references to screening activities in other locations that may be able to provide helpful ideas and collaborations to assist in overcoming NBS implementation challenges.

For additional guidance in NBS implementation and expansion, the interested reader is referred to the website of the Clinical Laboratory Standards Institute (CLSI—https://clsi.org), a volunteer-driven, membership-supported, not-for-profit, standards development organization. CLSI promotes the development and use of voluntary laboratory consensus standards and guidelines within the healthcare community and has taken a particular interest in NBS, having published in 2021 the 7th edition of its CLSI international “standard,” NBS 01: Dried Blood Spot Collection for Newborn Screening. In recent years, publication of CLSI international “guidelines” have included a number of NBS priority areas: NBS 02: Newborn Screening Follow-up and Education (2023); NBS 03: Newborn Screening for Preterm, Low Birth Weight, and Sick Newborns (2019); NBS 04: Newborn Screening by Tandem Mass Spectrometry (2017); NBS 05: Newborn Screening for Cystic Fibrosis (2019); NBS 06: Newborn Blood Spot Screening for Severe Combined Immunodeficiency by Measurement of T-cell Receptor Excision Circles (2013); NBS 07: Newborn Blood Spot Screening for Pompe Disease by Lysosomal Acid α-Glucosidase Activity Assays (2017); Newborn Screening for Hemoglobinopathies (2019); Newborn Screening for X-Linked Adrenoleukodstrophy (2021); and NBS 10: Newborn Screening for Congenital Hypothyroidism (2024). Materials are periodically reviewed and updated in compliance with CLSI protocols and using document development groups with a balance of public, private, academic and global expertise. While documents and accompanying materials are for sale, new pricing based on World Bank Income Groups allows for up to a 90% price discount for purchasers in LIC and LMIC countries.

Throughout the literature reviews provided here, are many examples of collaborations between NBS programs. Not only are these collaborations present in NBS start-up situations, but they are also present in expansion and follow-up activities. Collaborative projects exist in laboratory methodology, data collection and use, and NBS clinical follow-up, among others. While many collaborations exist between neighboring countries, states, and provinces, there are also ongoing projects between colleagues located far apart. We hope that the variations in collaborative activities reported here provide food for thought, especially for NBS researchers with innovative curiosity who are seeking to improve and expand NBS.

The data tables displayed were developed in a manner similar to those in our 2015 report, but are more harmonized. These tables provide a concise overview of NBS globally. Aside from the large number of conditions being screening in the USA, most other countries have taken a more conservative approach to screening, at least with respect to the number of disorders screened. For that reason, we have used a smaller number of conditions to harmonize regional data collection. While not formally adopted as a national model in Canada, a proposed listing of conditions suitable for expanded NBS by MS/MS was developed by a Canadian working group [37]. This list provides the basic conditions that seem to be the targets for most programs internationally and we have chosen to use it here for international comparisons (with a limited number of additions). Where additional screening activity is present, it has been listed in the table as “other” and further explained in table footnotes.

Data columns have been included for other items helpful in country and screening program comparisons and harmonization. All NBS programs seek to provide early information to help in identifying potential health issues. To this end, the number of NBS programs recommending specimen collection after 24 hours. of age appears to be increasing as testing analytical processes improve and the natural history of screened conditions is better understood. The screening efficiencies offered by centralizing and regionalizing screening testing appears to have led to increased consolidation of laboratory services, particularly for small birth population jurisdictions and some others as analytical techniques become more complex and expensive. Increasingly, smaller programs are “splitting” specimens for analysis, with one or more bloodspots for more complex conditions being analyzed in specialty laboratories. The reviews of state/province/country screening activities also contain references to the use of commercial laboratories and the transitions being made to accommodate NBS molecular laboratory services.

Among the activities reviewed, the number of projects involving NGS were particularly noteworthy with many projects for screening large numbers of genes in newborns underway. Not only are the laboratory procedures under study, but so too are the related financial, ethical, legal, and social issues. Such studies can be found in the reviews of countries in all regions, including in Africa, where despite the absence of organized NBS in most jurisdictions, private laboratories are already actively recruiting the submission of newborn specimens for NGS for hundreds of genes related to congenital conditions.

NBS advancements are poised to unlock a new era of personalized medicine for newborns. NGS is rapidly expanding the screening landscape from mostly metabolic disorders to neurogenetic and immune system vulnerabilities allowing for a wider spectrum of wider health challenges. DNA analyses extend beyond protein coding to other genetic regions with the potential to uncover genetic predispositions to diseases like diabetes, cancer, and autoimmune disorders leading to personalized healthcare strategies. As with the gene editing work ongoing with SCD, this technology has the potential to correct other genetic defects identified by NBS. Advanced bioinformatic tools are refining data analyses, reducing result turnaround times, and improving interpretations of complex genetic variations which in turn lead to earlier diagnosis and treatment.

Despite the excitement of expanded NBS and its potential in the future, it is also the responsibility of experts and companies experienced in our current NBS applications, usually from HIC settings, to assist developing programs in LIC or LMIC settings as they attempt to implement NBS programs. Our report is intended to serve as a ready reference on national and international NBS activities. As such, it can aid in knowledge sharing about program activities globally and assist in international NBS program harmonization. In addition to providing a comparative reference for developed NBS programs, it provides baseline data to begin comparisons on NBS in developing and undeveloped settings. The extensive number of references provides a comprehensive library of links to articles describing NBS activities since 2020, which are intended to assist the reader in accessing new or expanded knowledge about NBS. The program updates included here reflect the global NBS activities that are overcoming existing challenges and expanding NBS access to help create a healthier, more equitable future for all children.

## Figures and Tables

**Table 1:** Twenty-nine primary conditions included in the original USA RUSP.

	Organic Acid Disorders (OA)	Abbreviation		Fatty Acid Oxidation Disorders (FAO)	Abbreviation
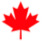	Isovaleric acidemia	IVA	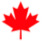	Medium-chain acyl-CoA dehydrogenase deficiency	MCAD
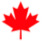	Glutaric acidemia-Type I	GA-I	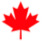	Very long-chain acyl-CoA dehydrogenase deficiency	VLCAD
	3-Hydroxy 3-methyl glutaric aciduria	HMG	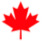	Long-chain L-3-OH acyl-CoA dehydrogenase deficiency	LCHAD
	Multiple carboxylase deficiency	MCD	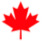	Trifunctional protein deficiency	TFP
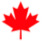	Methylmalonic acidemia (mutase deficiency)	MUT	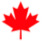	Carnitine uptake defect	CUD
	3-Methylcrotonyl-CoA carboxylase deficiency	3MCC		**Hemoglobinopathies (HGB)**	**Abbreviation**
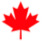	Methylmalonic acidemia (Cobalamin A and B)	CBL A, B	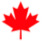	Hemoglobin S/S disease (Sickle cell anemia)	Hb S/S
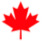	Propionic acidemia	PA	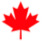	Hemoglobin S/β-thalassemia	Hb S/β-Th
	β-Ketothiolase deficiency	BKT	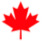	Hemoglobin S/C disease	Hb S/C
	**Amino Acid Disorders (AA)**	**Abbreviation**		**Other Clinically Significant Disorders**	**Abbreviation**
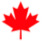	Phenylketonuria	PKU	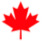	Congenital Hypothyroidism	CH
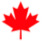	Maple syrup urine disease	MSUD	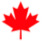	Biotinidase Deficiency	BIO
	Homocystinuria	HCY	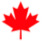	Congenital Adrenal Hyperplasia	CAH
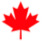	Citrullinemia	CIT	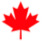	Galactosemia (classical transferase deficiency)	GALT
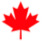	Argininosuccinic acidemia	ASA		Early hearing detection and intervention	EHDI
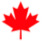	Tyrosinemia type I	TYR I	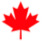	Cystic fibrosis	CF
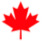 Indicates conditions included in the 2016 recommendations to the Canadian health ministry—See reference [36].					

**Table 2:** Twenty-five secondary conditions included in the original USA RUSP.

Organic Acid Disorders (OA)	Abbreviation	Fatty Acid Oxidation Disorders (FAO)	Abbreviation
Methylmalonic acidemia (Cobalamin C and D)	CBL C, D	Short-chain acyl-CoA dehydrogenase deficiency	SCAD
Malonic acidemia	MAL	Glutaric acidemia type II	GA-II
Isobutyryl-CoA dehydrogenase deficiency	IBDD	Medium/short-chain L3-OH acyl-CoA-dehydrogenase deficiency	M/SCHAD
2-Methyl 3-hydroxy butyric aciduria	2M3HBA	Medium-chain ketoacyl-CoA thiolase deficiency	MCKAT
2-methylbutyryl-CoA dehydrogenase deficiency	2MBG	Carnitine palmitoyl transferase II deficiency	CPT II
3-Methylglutaconic aciduria	3MGA	Carnitine acylcarnitine translocase deficiency	CACT
**Amino Acid Disorders (AA)**	**Abbreviation**	Carnitine palmitoyl transferase IA deficiency (liver)	CPT IA
Benign hyperphenylalaninemia	H-PHE	Dienoyl-CoA reductase deficiency	DE RED
Tyrosinemia type II	TYR II	**Hemoglobinopathies (HGB)**	**Abbreviation**
Defects of biopterin cofactor biosynthesis	BIOPT(BS)	Variant hemoglobinopathies (including Hb E)	Var HGB
Argininemia	ARG	**Other Clinically Significant Disorders**	**Abbreviation**
Tyrosinemia type II	TYR III	Galactokinase deficiency	GALK
Defects of biopterin cofactor regeneration	BIOPT(REG)	Galactose epimerase deficiency	GALE
Hypermethioninemia	MET		
Citrullinemia type II	CIT II		

**Table 3:** Conditions considered for addition to the original USA RUSP after its acceptance: 2008–2023.

Approved		Disapproved	
Condition	Date Added to RUSP	Condition	Date Disapproved
^a^ Infantile Krabbe disease (KD)	Sent to SHHS *	Duchenne muscular dystrophy (DMD)	February 2023
^b^ Guanidinoacetate methyltransferase deficiency (GAMT)	January 2023	Congenital cytomegalovirus (cCMV)	August 2022
Mucopolysaccharidosis II (MPS-II)	August 2022	Cerebrotendinous xanthomatosis (CTX)	November 2018
^c^ Spinal muscular atrophy (SMA)	July 2018	22q11.2 deletion syndrome	January 2012
^c^ Adrenoleukodystrophy (ALD)	February 2016	Neonatal hyperbilirubinemia/kernicterus	January 2012
Mucopolysaccharidosis I (MPS-I)	February 2016	Hemoglobin H disease (HbH disease)	May 2010
Pompe disease (PD)	March 2015	Niemann–Pick disease	October 2008
Critical congenital heart disease (CCHD)	September 2011	Fabry disease	August 2008
^d^ Severe combined immunodeficiency (SCID)	February2010		
* Secretary of the USA Department of Health and Human Services. ^a^ Approved on 3rd nomination October 2023. Disapproved on 2 previous nominations: September 2009 and February 2023. Recommendation for infantile KD, as defined by low GALC enzyme and psychosine ≥ 10 nM, forwarded to SHHS on 1 March 2024. ^b^ Approved on 3rd nomination. Not approved June 2016 and November 2016. ^c^ Approved on 2nd nomination. SMA not approved November 20008; ALD not approved September 2012. ^d^ T-cell lymphocyte deficiencies were included as a secondary condition.			

**Table 4:** Newborn Screening Data for the United States of America.

Jurisdiction	Demographic Information				Disorder																												
					Core RUSP										Secondary RUSP							Miscellaneous											
Shading Indicates a Shared Laboratory: 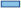 Iowa 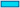 Colorado 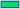 Revvity 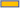 Washington 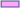 Massachusetts 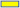 Oregon	^a^ Population (× 1000) Latest Data—2023	^b^ Births (Thousands) 2022, Provisional	Date Universal Screening Required	^c^ Newborn Screening Fee (US$)	^d^ 28 Core RUSP—DBS	EHDI	SCID	CCHD	Pompe	MPS-I	ALD	SMA	MPS-II	GAMT Deficiency	Other Hemoglobins	Galactokinase	Galactoepimerase	^d^ 8 Secondary AAs	^d^ 8 Secondary FAOs	^d^ 6 Secondary OAs	T-cell Defect	Krabbe	Fabry	Gaucher	Niemann Pick	FIGLU	HHH	G6PD Deficiency	HIV	TOXO	5-OXO	cCMV	Other
**Alabama**	**5108**	**58.1**	**1965**	**150.00 ^e^**	●	●	●	●	🞇	🞇	🞇	●			●			●	●	●	●												
**Alaska**	**733**	**9.3**	**1965**	**190.50**	●	●	●	●				●			●			●	●	●	●												
**Arizona**	**7431**	**78.5**	**1979**	**171.00 ^e^**	●	●	●	●			●	●			●			●	●	●	●												
**Arkansas**	**3068**	**35.3**	**1967**	**131.00**	●	●	●	●				●			●			●	●	●	●												
**California**	**38,965**	**18.5**	**1965**	**210.00**	●	●	●	●	●	●	●	●			●	●	●	●	●	●	●		●	●	●	●	●						
**Colorado**	**5878**	**62.33**	**1965**	**111.00 ^e^**	●	●	●	●	🞇	🞇	🞇	●			●			●	●	●	●												
**Connecticut**	**3617**	**35.3**	**1965**	**113.00**	●	●	●	●	●	●	●	●		●	●	●	●	●	●	●	●											🞇	
**Delaware**	**1032**	**10.7**	**1962**	**135.00**	●	●	●	●	●	●	●	●			●	●	●	●	●	●	●												
**District of Columbia**	**679**	**8.0**	**1980**	**0.00**	●	●	●	●	●	●	●	●			●	●	●	●	●	●	●							●					
**Florida**	**22,611**	**224.6**	**1965**	**15.00 ^f^**	●	●	●	●	●	●	●	●			●			●	●	●	●												
**Georgia**	**11,029**	**125.8**	**1966**	**80.40**	●	●	●	●	●	●	●	●			●			●	●	●	●	🞇											
**Hawaii**	**1435**	**15.2**	**1965**	**99.00**	●	●	●	●				●			●			●	●	●	●												
**Idaho**	**1965**	**22.3**	**1965**	**120.70 ^e^**	●	●	●	●	●	●	●	●			●			●	●	●	●												
**Illinois**	**12,550**	**128.3**	**1965**	**128.00**	●	●	●	●	●	●	●	●	●		●	●	●	●	●	●	●	●	●	●	●						●		
**Indiana**	**6862**	**79.6**	**1965**	**120.00**	●	●	●	●	●	●	●	●			●	●	●	●	●	●	●	●											
**Iowa**	**3207**	**36.4**	**1965**	**162.00**	●	●	●	●	🞇	🞇	🞇	●			●			●	●	●	●												
**Kansas**	**2941**	**34.4**	**1965**	**0.00**	●	●	●	●	●	●		●			●			●	●	●	●												
**Kentucky**	**4526**	**52.2**	**1966**	**150.00**	●	●	●	●	●	●	●	●			●			●	●	●	●	●											
**Louisiana**	**4474**	**56.1**	**1964**	**30.00**	●	●	●	●	●	●	🞇	●			●			●	●	●	●												
**Maine**	**1396**	**12.1**	**1965**	**220.00**	●	●	●	●	●	●	●	●			●	●	●	●	●	●	●					🞇	●						
**Maryland**	**6180**	**68.7**	**1965**	**106.00 ^e^**	●	●	●	●	●	●	●	●			●	●	●	●	●	●	●		●										
**Massachusetts**	**7001**	**68.6**	**1963**	**170.21**	●	●	●	●	●	●	●	●			●	●	●	●	●	●	●					🞇	●			●			
**Michigan**	**10,037**	**102.2**	**1965**	**166.38**	●	●	●	●	●	●	●	●		●	●	●	●	●	●	●	●												
**Minnesota**	**5738**	**63.9**	**1965**	**235.00**	●	●	●	●	●	●	●	●			●	●	●	●	●	●	●											●	
**Mississippi**	**2940**	**34.6**	**1985**	**110.00**	●	●	●	●	●	●		●			●	●	●	●	●	●	●						●				●		
**Missouri**	**6196**	**69.0**	**1965**	**102.00**	●	●	●	●	●	●	●	●	●		●	●	●	●	●	●	●	●	●	●	●								
**Montana**	**1133**	**11.2**	**1965**	**134.00**	●	●	●	●				●			●			●	●	●	●												
**Nebraska**	**1978**	**24.3**	**1967**	**86.00**	●	●	●	●	●	●	●	●			●	●	●	●	●	●	●												
**Nevada**	**3194**	**33.2**	**1967**	**81.00 ^e^**	●	●	●	●			🞇	●			●	●	●	●	●	●	●												
**New Hampshire**	**1402**	**12.1**	**1965**	**146.00**	●	●	●	●	●	●	●	●			●			●	●	●	●					🞇	●			●	●		
**New Jersey**	**9291**	**102.9**	**1964**	**150.00**	●	●	●	●	●	●	🞇	●			●	●	●	●	●	●	●	●	●	●	●								
**New Mexico**	**2114**	**19.5**	**1966**	**240.00 ^e^**	●	●	●	●	●	●	●	●			●			●	●	●	●		●	●									
**New York**	**19,571**	**207.5**	**1964**	**0.00**	●	●	●	●	●	●	●	●		●	●			●	●	●	●	●							●				
**North Carolina**	**10,835**	**121.4**	**1983**	**128.00**	●	●	●	●	●	●	●	●			●	●	●	●	●	●	●												
**North Dakota**	**784**	**9.6**	**1967**	**109.00**	●	●	●	●				●			●			●	●	●	●												
**Ohio**	**11,786**	**128.2**	**1965**	**98.63**	●	●	●	●	●	●	🞇	●			●	●	●	●	●	●	●	●											
**Oklahoma**	**4054**	**48.3**	**1965**	**160.42**	●	●	●	●	●	●	●	●			●	●	●	●	●	●	●												
**Oregon**	**4233**	**39.5**	**1963**	**175.00 ^e^**	●	●	●	●	●	●	●	●			●			●	●	●	●		●	●									
**Pennsylvania**	**12,962**	**130.0**	**1965**	**41.72**	●	●	●	●	●	●	●	●	●	●	●	●	●	●	●	●	●	●						🞇					
**Rhode Island**	**1096**	**10.2**	**1965**	**162.98**	●	●	●	●	●	●	●	●			●			●	●	●	●					🞇	🞇						
**South Carolina**	**5374**	**57.8**	**1965**	**127.00**	●	●	●	●	●	●		●			●	●	●	●	●	●	●												
**South Dakota**	**919**	**11.2**	**1973**	**98.00**	●	●	●	●				●			●			●	●	●	●												
**Tennessee**	**7126**	**82.3**	**1968**	**185.00**	●	●	●	●	●	●	●	●			●	●	●	●	●	●	●	●	●	●									
**Texas**	**30,503**	**389.5**	**1965**	**127.10 ^e^**	●	●	●	●			●	●			●			●	●	●	●												
**Utah**	**3418**	**45.8**	**1965**	**140.00 ^e^**	●	●	●	●			●	●		●	●			●	●	●	●												
**Vermont**	**647**	**5.3**	**1962**	**203.00**	●	●	●	●	●	●	●	●			●	●	●	●	●	●	●					🞇	●						
**Virginia**	**8716**	**95.4**	**1966**	**138.00**	●	●	●	●	●	●	●	●			●			●	●	●	●											🞇	
**Washington**	**7813**	**83.2**	**1967**	**135.10 ^e^**	●	●	●	●	●	●	●	●			●			●	●	●	●												
**West Virginia**	**1770**	**16.9**	**1965**	**241.35**	●	●	●	●	●	●		●	●		●			●	●	●	●												
**Wisconsin**	**5911**	**59.9**	**1965**	**195.00**	●	●	●	●	●			●			●			●	●	●	●												
**Wyoming**	**584**	**6.0**	**1983**	**97.32 ^e^**	●	●	●	●				●			●	●	●	●	●	●	●												
**Total**	**334,813**																																
**Guam**	**153**	**2.6**			●	●	●	●	●	●					●	●	●	●	●	●	●												
**Northern Mariana Islands**	**47**	**0.6**			●	●	●	●							●	●	●	●	●	●	●												
**Puerto Rico**	**3206**	**19.3**		**130.00**	●	●	●	●							●	●	●	●	●	●	●												
**American Samoa**	**50**	**-**																															
**Virgin Islands**	**87**	**0.9**			●	●		●																									
**Abbreviations not in the text: FIGLU = Formiminoglutamic Acid; HHH = Hyperornithinemia-Hyperammonemia-Homocitrullinuria; G6PD = Glucose-6-Phosphate Dehydrogenase; HIV = Human Immunodeficiency Virus; 5-OXO = 5-Oxoproline; cCMV = Congenital Cytomegalovirus; DBS = dried blood spot** ● **Indicates full population mandate.** 🞇 **Indicates less than full population mandate—includes pilot testing or testing on a part of the population based on another test (e.g., cCMV after positive EHDI)** **^a^ Available at: https://worldpopulationreview.com/states (accessed 10 January 2024)** **^b^ https://www.cdc.gov/nchs/data/vsrr/vsrr028.pdf (provisional data for 2022—accessed 10 January 2024)** **^c^ Available: https://www.newsteps.org/resources/data-visualizations/newborn-screening-fees-and-fee-details (accessed 10 January 2024)** **^d^ See Table 1** **^e^ Indicates a program requiring 2 screens—fee indicated covers the 2 required screens (fee may be charged in 2 parts—Texas charges $63.55 twice)** **^f^ An additional fee is charged to insurance by the NBS laboratory**																																	

**Table 5:** Newborn Screening Data for Canada.

Demographics				Disorders																																
Jurisdiction	Details			Miscellaneous											Amino Acid						Fatty Acid Oxidation								Organic Acid							
(See Footnotes in Table regarding Collaborations.)	^a^ Population (x1000) Latest Data—2023	^b^ Births 2022/2023 Latest Official Data.	^c^ Date Screening Began	^d^ CH	^d^ CAH	^d^ GAL	^d^ BIO	^d^ HGB	^d^ CF	^d^ SCID	SMA	cCMV	GAMT	Other (See below)	^d^ PKU	^d^ MSUD	^d^ TYR-I	^d^ ASA	^d^ CIT	HCY	^d^ CUD	^d^ TFP	^d^ MCAD	^d^ LCHAD/TFP	^d^ VLCAD	GA-II	CPT- I/CPT -II	CACT	^d^ IVA	^d^ MMA (MUT)/(CBL)	^d^ PA	^d^ GA-I	MCD	BKT	HMG	3-MCC
**Provinces**																																				
**^e^** **Alberta**	**4756.4**	**48,523**	**1967**	●	●	●	●	●	●	●	●				●	●	●		●		●	●	●	●	●				●	●	●	●			●	
**^f^** **British Columbia**	**581.15**	**4103**	**1964**	●	●	●	●	●	●	●	●		●		●	●	●	●	●	●	●	●	●	●	●				●	●	●	●				
**^g^** **Manitoba**	**1465.4**	**17,146**	**1965**	●	●	●	●		●	●	●	●		**1**	●	●	●	●	●	●	●	●	●	●	●	●	●		●	●	●	●	●	●	●	●
**^h^** **New Brunswick**	**842.7**	**6388**	**1966**	●		●	●	●	●	●					●	●		●	●		●	●	●	●	●		●	●	●	●	●	●				
**New Foundland and Labrador**	**540.4**	**3566**	**1978**	●	●		●	●	●	●					●	●	●	●	●	●	●	●	●	●	●			●	●	●	●	●				
**^h^** **Nova Scotia (Maritime)**	**1066.4**	**8122**	**1966**	●		●	●	●	●	●					●	●		●	●		●	●	●	●	●		●	●	●	●	●	●				
**^i^** **Ontario**	**15,801.8**	**137,748**	**1965**	●	●	●	●	●	●	●	●	●	●	**2**	●	●	●	●	●	●	●	●	●	●	●		●	●	●	●	●	●				
**^h^** **Prince Edward Island**	**175.9**	**1373**	**1963**	●		●	●	●	●	●					●	●		●	●		●	●	●	●	●		●	●	●	●	●	●				
**Quebec**	**8948.5**	**79,050**	**1971**	●			●	●	●	●	●			**3**	●	●	●	●	**†**			●	●	●	●					**†**	**†**	●				
**Saskatchewan**	**1219.0**	**13,343**	**1965**	●	●	●	●	●	●	●	●	●	●	**4**	●	●	●	●	●	●	●	●	●	●	●	●	●		●	●	●	●	●	●	●	●
**Territories**																																				
**^e^** **Northwest Territories**	**44.8**	**502**	**1965**	●	●	●	●	●	●	●	●				●	●	●		●		●	●	●	●	●				●	●	●	●			●	
**^e^** **Nunavut-Kitikmeot**	**40.8**	**708**	**1965**	●	●	●	●	●	●	●	●				●	●	●		●		●	●	●	●	●				●	●	●	●			●	
**^g^** ** - Kivalliq**			**1965**	●	●	●	●		●	●	●				●	●	●	●	●	●	●	●	●	●	●	●	●		●	●	●	●	●	●	●	●
**^i^ - Kivalliq Qikiqtaaluk**			**1965**	●	●	●	●	●	●	●	●	●	●		●	●	●	●	●	●	●	●	●	●	●		●		●	●	●	●				
**^f^** **Yukon**	**45.1**	**399**		●	●	●		●	●	●	●		●		●	●	●	●	●	●	●		●	●	●				●	●	●	●				
**Totals**	**40,528.4**	**357,903**																																		
● **Indicates availability to full population** **† Screening performed using urine specimens (versus dried bloodspots).** ^**a**^ **Statistics Canada. 2023 Quarter 4 population estimates. Available: https://doi.org/10.25318/1710000901-eng (accessed 26 December 2023).** **^b^ Statistics Canada. Table: 17-10-0008-01 Release date: 2023-09-27. Available: https://doi.org/10.25318/1710000801-eng (accessed 26 December 2023).** **^c^ Dates previously published—see Reference [27]** **^d^ Twenty-two conditions have been suggested for NBS in Canada, including mutase and cobalamin disorders (counted as one condition in the table). See Reference [37]**. **^e^ University of Alberta Hospital (UAH); Edmonton, Alberta, performs screening testing for Alberta, the Northwest Territories and Kitikmeot, Nunavut.** **^f^ British Columbia Children’s Hospital; Vancouver, British Columbia, performs screening testing for British Columbia and for the Yukon.** **^g^ The Cadham Provincial Laboratory; Winnipeg, Manitoba, performs screening testing for Manitoba and for a limited number of newborns in Kivalliq, Nunavut.** **^h^ (Maritime Program) Izaak Walton Killam (IWK) Children’s Health Centre; Halifax, Nova Scotia, performs testing for Nova Scotia, New Brunswick, and Prince Edward Island.** **^i^ The Ontario laboratory at Children’s Hospital of Eastern Ontario (CHEO); Ottawa, Ontario, performs screening testing for Ontario,** ***Qikiqtaaluk (Baffin),*** **Kitikmeot, Nunavut.** **1 Also screens for congenital cytomegalovirus (cCMV)** **2 Also screens for mucopolysaccharidosis type 1 (MPS-I)** **3 Also performs urine screening for hyperornithinemia-hyperammonemia-homocitrullinuria (HHH) syndrome** **4 Babies automatically receive testing in the Keewatin Yatthé Health Region, all Meadow Lake Tribal Council communities, and all babies born at the Meadow Lake Hospital due to overrepresentation in northwest Saskatchewan. Performed by special request in other areas of the province.**																																				

**Table 6:** Newborn Screening Data—Asia-Pacific Region.

Demographic Information							Disorder																														
							Miscellaneous										Amino Acids						Fatty Acids							Organic Acids							
Country	^a^ Population (Millions) 2023 UNICEF Data	^b^ Births (Thousands) 2023 UNICEF Data	^c^ Infant (<1 Year) Mortality Rate (per 1000) 2022	Date Screening Began	Screening Laboratories	Specimen Collection Time (Hrs.) -	^d^ CH	^d^ CAH	^d^ GAL	^d^ BIO	^d^ HGB	G6PDD	^d^ SCID	SMA	^d^ CF	Other (see key below)	^d^ PKU	^d^ MSUD	^d^ TYR-I	^d^ ASA	^d^ CIT	HCY	^d^ CUD	CACT	^d^ MCAD	^d^ LCHAD/TFP	^d^ VLCAD	SCAD	CPT- I/CPT -II	^d^ IVA	^d^ MMA (MUT)/(CBL)	^d^ PA	^d^ GA-I	MCD	BKT	HMG	3-MCC
**Australia**	**26.4**	**300.2**	**3.3**	**1964**	**5**	**48–72**	●	●	●				🞇	🞇	●	**1**	●	●		●	●	●	●		●	●	●		●	●	●	●	●		●	●	
**Bangladesh**	**173.0**	**2995**	**22.9**	**1999**	**1**	**168–240**	**§**																														
**Bhutan**	**0.8**	**9.6**	**22.5**	**n/a**	**No information**																																
**Brunei Darussalam**	**0.5**	**6.1**	**9.6**	**n/a**	**No information**																																
**Cambodia**	**16.9**	**318.3**	**21.3**	**2013**	**1**	**48**	**§**					**§**																									
**China**	**1425.7**	**10,758**	**5.1**	**1981**	**269**	**48**	●	●	**§**		🞇	●	**§**	**§**			●	🞇	🞇	🞇	🞇	🞇	🞇	🞇	🞇	🞇	🞇	🞇	🞇	🞇	🞇	🞇	🞇	🞇	🞇	🞇	🞇
**Hong Kong SAR, China**		**41.9**	**1.2**	**1984**	**1 #**	**24–72**	●	●	●	●		●	●	**§**		**2**	●	●	●	●	●	●	●	●	●		●		●	●	●	●	●	●	●	●	
**India**	**1428.6**	**23,056**	**25.5**	**1980**	**49 **†****	**24–72**	**§**	**§**			**§**	**§**	**§**	**§**			**§**	**§**	**§**	**§**	**§**	**§**	**§**	**§**	**§**	**§**	**§**	**§**	**§**	**§**	**§**	**§**	**§**	**§**	**§**	**§**	**§**
**Indonesia**	**277.5**	**4462**	**18.9**	**1999**	12 *	**24–72**	●																														
**Japan**	**123.3**	**814.7**	**1.7**	**1977**	**35**	**120–168**	●	●	●				**§**	**§**		**3**	●	●		●	●	●			●	●	●		●	●	●	●	●	●		●	●
**Korea (North)**		**340.4**	**10.1**	**n/a**	**No Information**																																
**Korea (South)**		**285.7**	**2.5**	**1991**	**13**	**24–72**	●	●	●				●				●	●	●	●	●	●	●	●	●	●	●	●	●	●	●	●	●	●	●	●	●
**Lao, PDR**	**7.6**	**161.7**	**34.2**	**2008**	**1**	**-**	**†**																														
**Macau SAR, China**		**7.0**	**4.7**	**1977**	**1**	**48**	●	●	**§**		🞇	●	**§**	**§**			●	**§**	**§**	**§**	**§**	**§**	**§**	**§**	**§**	**§**	**§**	**§**	**§**	**§**	**§**	**§**	**§**	**§**	**§**	**§**	**§**
**Malaysia**	**34.3**	**509.2**	**6.5**	**1980**	**4#**	**24–168**	●					●					**§**	**§**	**§**	**§**	**§**	**§**	**§**	**§**	**§**	**§**	**§**	**§**	**§**	**§**	**§**	**§**	**§**	**§**	**§**	**§**	**§**
**Mongolia**	**3.4**	**69.5**	**12.7**	**2000**	**2**	**24–72**	**§**	**§**	**§**			**§**			**§**																						
**Myanmar**	**54.6**	**912.3**	**33.7**	**n/a**		**-**	**§**																														
**Nepal**	**30.9**	**616.7**	**22.8**	**n/a**	**1**	**24–48**	**§**	**§**		**§**	**§**	**§**																									
**New Zealand**	**5.2**	**64.1**	**3.9**	**1964**	**1**	**24–72**	●	●	●	●			●	**§**	●	**4**	●	●	●	●	●	●	×	●	●	●	●		●	●	●	●	●	×	×	×	×
**Pakistan**	**240.5**	**6425**	**52.8**	**2000**	**1**	**48–240**	**§**	**§**	**§**	**§**					**§**																						
**Papua New Guinea**	**10.3**	**254.7**	**34.4**	**n/a**	**No Information**																																
**Philippines**	**117.3**	**2499**	**20.5**	**1996**	**7**	**24–48**	●	●	●	●	●	●			●	**5**	●	●	●	●	●	●	●		●	●	●	●	●	●	●	●	●	●	●		●
**Singapore**	**6.0**	**42.3**	**1.7**	**1965**	**1 #**	**24–72**	●	●	●	●		●	●		●	**6**	●	●	●	●	●	●	●	●	●	●	●	●	●	●	●	●	●	●	●	●	●
**Sri Lanka**	**21.9**	**301.4**	**5.8**	**2006**	**2**	**6–48**	●																														
**Taiwan**		**186.2**	**4.1**	**1981**	**3**	**48–72**	●	●	●	●		●	●	●		**7**	●	●	●	●	●	●	●	●	●	●	●	●	●	●	●	●	●	●	●	●	●
**Thailand**	**71.8**	**631.2**	**7.1**	**1992**	**2**	**48–72**	●										●	●	●	●	●	●	●	●	●	●	●	●	●	●	●	●	●	●	●	●	●
**Việt Nam**	**98.9**	**1443**	**16.4**	**2000**	**8**	**24–48**	●	🞇	🞇	🞇	🞇	●	🞇	🞇	🞇		🞇	🞇	🞇	🞇	🞇	🞇	🞇	🞇	🞇	🞇	🞇	🞇	🞇	🞇	🞇	🞇	🞇	🞇	🞇	🞇	🞇
**Totals**		**57,511**	**15.0**	**-**	**-**	**-**																															
**Abbreviations: SAR = Special Administrative Region; PDR—Peoples’ Democratic Republic** **●** **More than 50% coverage; part of national or administrative region implementation/law** **🞇** **Less than 50% coverage; part of national or administrative region implementation/law** **×** **Discontinued testing—see appropriate note under ‘Other’** **§** **Only available on a limited basis—screening may part of a pilot or limited to a specific birthing facility by contract and not generally available to the full public** **† Pilot has been completed but program is not yet stable, and the number of screening laboratories is not yet fixed** # NBS using cord blood is performed in all hospitals for CH and G6PDD * NBS samples are analyzed within the existing laboratories of the hospitals **^a^ https://data.unicef.org/resources/data_explorer/unicef_f/?ag=UNICEF&df=GLOBAL_DATAFLOW&ver=1.0&dq=.DM_POP_TOT..&startPeriod=2023&endPeriod=2023 (accessed 27 March 2024)** **^b^https://data.unicef.org/resources/data_explorer/unicef_f/?ag=UNICEF&df=GLOBAL_DATAFLOW&ver=1.0&dq=.DM_BRTS.&startPeriod=2023&endPeriod=2023 (accessed 27 March 2024)** **^c^ https://data.unicef.org/resources/data_explorer/unicef_f/?ag=UNICEF&df=GLOBAL_DATAFLOW&ver=1.0&dq=.CME_MRY0._T.&startPeriod=2021&endPeriod=2023 (accessed 27 March 2024)** **^d^ Indicates a condition chosen to be on the Canadian newborn screening panel and which is being used to harmonize the tables in this report.** **1 =Multiple acyl*-*CoA dehydrogenase deficiency *(*MADD*);*GA-2, Holocarboxylase synthetase deficiency, Tyr II, III; For an up-to date listing, see: https://www.health.gov.au/our-work/newborn-bloodspot-screening/what-is-screened (accessed 27 March 2024)** **2 = Argininemia,6-pyruvoyl-tetrahydropterin synthase deficiency, Glutaric Acidemia Type II. Citrullinemia type II. Information shown is for government program. Additional testing may be available in the private sector.** **3 = GA-2, citrin deficiency, methylglutaconic aciduria—pilot** **4 = TFP, MADD—Discontinued August 2015: 3MCC; HMG; MCD; BKT; 3-methylglutaconyl-CoA-hydratase deficiency; 2-methyl-3-hydroxybutyryl CoA dehydrogenase deficiency—Discontinued April 2017: CUD (See: https://www.nsu.govt.nz/pregnancy-newborn-screening/newborn-metabolic-screening-programme-heel-prick-test/about-test) (accessed 27 March 2024)** **5 = MAT, GA-2, TFP (See: https://www.ncbi.nlm.nih.gov/pmc/articles/PMC8883932/)(accessed 27 March 2024)** **6 = GA-2, Medium-chain ketoacyl-CoA thiolase deficiency (MCAT), *Ethylmalonic encephalopathy*(*EE),* 2-methyl-3-hydroxybutyric aciduria (HSD10),2-Methyl-3-hydroxybutyryl-CoA dehydrogenase deficiency (2M3HBD), 2-methylbutyryl-CoA dehydrogenase deficiency, 6-pyruvoyl-tetrahydropterin synthase deficiency and 3-methylglutaconic aciduria** **7 = Pompe, Gaucher, citrin, MPS-1,2,3b, ALD—Not fully implemented at the time of writing: Fabry, DMD, MPS 4a, 6,aromatic 1-amino acid decarboxylase deficiency (AADC)**																																					

**Table 7:** Summary Newborn Screening Data from Europe.

Demographic Information						Disorder																														
						Miscellaneous										Amino Acid						Fatty Acid Oxidation							Organic Acid							
Country [Screening Covered By] 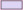 Member of European Union	^a^ Population (Millions) 2023 UNICEF Data	^b^ Births (Thousands) 2023 UNICEF Data	Date Screening Began	^c^ Screening Laboratories	^c^ Specimen Collection Time (Hrs.)	CH	CAH	GAL	BIO	HGB	G6PD	SCID	CF	SMA	Other (see key below)	PKU	MSUD	TYR-I	ASA	CIT	HCY	CUD	CACT	MCAD	LCHAD/TFP	VLCAD	SCAD	CPT- I/CPT -II	IVA	MMA (MUT)/(CBL)	PA	GA-I	MCD	BKT	HMG	3-MCC
**Albania**	**2.8**	**28.5**																																		
**Andorra [France]**	**0.1**	**0.6**			**48–72**	●	●			●			●			●	●	●			●	●		●	●				●			●				
**Armenia**	**2.8**	**31.6**		**2**	**48–96**	●										●																				
**Austria**	**9.0**	**84.1**	**1967**	**1**	**36–72**	●	●	●	●				●	●	**1, 4**	●	●	●	●	●	●	●	●	●	●	●		●	●	●	●	●				
**Azerbaijan**	**10.4**	**120.5**		**1**	**48–72**	●	●	●		**†**	●					●																				
**Belarus**	**9.5**	**86.2**	**1978**	**1**	**72–120**	●										●																				
**Belgium**	**11.7**	**116.8**	**1964**	**4**	**48–120**	●	●	●	●	🞇	🞇		●	●	**1**	●	●	●			●			●		●			●	●	●	●				
**Bosnia-Herzegovina**	**3.2**	**26.3**	**2000**	**3**	**48–96**	●	●									●																				
**Bulgaria**	**6.7**	**55.5**	**1978**	**2**	**72–120**	●	●									●																				
**Croatia**	**4.0**	**33.5**	**1978**	**1**	**48–72**	●								●		●						●		●	●	●			●			●				
**Cyprus**	**1.3**	**12.3**	**1988**	**1**	**48–168**	●										●																				
**Czechia**	**10.5**	**99.7**	**1975**	**4**	**48–72**	●	●		●				●	**2**	**4**	●	●			●	●		●	●	●	●		●	●			●				
**Denmark**	**5.9**	**65.0**	**1978**	**1**	**48–72**	●	●	●	●			●	●	●		●	●	●	●			●		●	●	●			●	●	●	●	●			
**Estonia**	**1.3**	**13.1**	**1993**	**1**	**48–72**	●								●		●	●	●	●	●	●	●	●	●	●	●		●	●	●	●	●				
**Finland**	**5.5**	**47.1**	**1980**	**1**	**36–120**	●	●					●			**1**	●	●	●	●	●	●	●	●	●	●	●		●	●	●	●	●				
**France**	**64.8**	**671.4**	**1967**	**16**	**48–72**	●	●			●			●	**2**		●	●	●			●	●		●	●				●			●				
**Georgia**	**3.7**	**47.8**	**2007**	**1**	**48–72**	●							●			●																				
**Germany**	**83.3**	**755.1**	**1969**	**11**	**36–72**	●	●	●	●	●		●	●	●		●	●	●					●	●	●	●		●	●	●	●	●			●	
**Greece**	**10.3**	**76.1**	**1974**	**1**	**48–72**	●		●			●	●				●																				
**Hungary**	**10.2**	**104.8**	**1975**	**2**	**48–72**	●		●	●				🞇	**2**	**1**	●	●	●	●	●	●		●	●	●	●	●	●	●	●	●	●	●	●	●	●
**Iceland**	**0.4**	**4.6**	**1972**	**1**	**48–72**	●	●					●			**1, 3**	●	●		●	●	●	●	●	●	●	●	●	●	●	●	●	●	●	●	●	●
**Ireland**	**5.1**	**56.7**	**1966**	**1**	**72–120**	●		●		🞇		●	●	**†**		●	●				●			●								●				
**Italy**	**58.9**	**406.2**	**1983**	**15**	**48–72**	●	🞇	●	●	🞇	🞇	🞇	●	**†**	**1, 4**	●	●	●	●	●	●	●	●	●	●	●	●	●	●	●	●	●	●	●	●	●
**Kazakhstan**	**19.6**	**395.3**	**2007**	**21**	**24–72**	●							🞇			●																				
**Kosovo**	**1.7**	**18.5**																																		
**Kyrgyz Republic**	**6.7**	**152.4**		**1**	**48–72**	●																														
**Latvia**	**1.8**	**16.0**	**1980**	**1**	**48–72**	●	●	●	●				●	●		●																				
**Liechtenstein [Switzerland]**	**0.04**	**0.4**			**72–96**	●	●	●	●			●	●	**2**		●	●							●								●				
**Lithuania**	**2.7**	**25.6**	**1975**	**1**	**48–96**	●	●	●								●																				
**Luxembourg**	**0.7**	**6.7**	**1968**	**1**	**48–72**	●	●						●			●								●												
**North Macedonia**	**2.1**	**19.8**	**1976**	**1**	**32–72**	●							🞇	●	**1**	●	●	●	●	●	●	●	●	●	●	●	●	●	●	●	●	●	●	●		●
**Malta**	**0.5**	**4.9**	**2017**	**2**	**72–120**	●				●						●																				
**Moldova**	**3.4**	**49.7**	**1989**	**1**	**>48**											●																				
**Monaco [France]**	**0.04**	**0.3**			**48–72**	●	●			●			●			●	●	●			●	●		●	●				●			●				
**Montenegro**	**0.6**	**6.8**	**2007**	**1**	**24–72**	●																														
**Netherlands**	**17.6**	**182.4**	**1974**	**5**	**72–96**	●	●	●	●	●		●	●	●	**3**	●	●	●						●	●	●		●	●	●	●	●	●		●	●
**Norway**	**5.5**	**54.5**	**1965**	**1**	**48–72**	●	●		●			●	●	●		●	●	●			●	●	●	●	●			●	●	●	●	●	●	●	●	
**Poland**	**41.0**	**423.7**	**1964**	**6**	**48–96**	●	●	🞇	●			🞇	●	●	**1**	●	●	●	●	●	●	●	●	●	●	●		●	●	●	●	●		●	●	●
**Portugal**	**10.2**	**79.5**	**1979**	**1**	**48–72**	●							●	●	**1**	●	●	●	●	●	●	●	●	●	●	●		●	●	●	●	●			●	●
**Romania**	**19.9**	**214.2**	**1999**	**5**	**48–72**	●								**2**		●		●																		
**Russia**	**144.4**	**1376**	**1993**	**78**	**48–72**	●	●	●					●	●		●																				
**San Marino [Italy]**	**0.03**	**0.2**	**1983**		**48–72**	●	🞇	●	●	🞇	🞇	🞇	●		**1, 4**	●	●	●	●	●	●	●		●	●	●	●	●	●	●	●	●	●	●	●	●
**Serbia**	**7.1**	**65.2**	**1982**	**2**	**48–72**	●						🞇	●	●		●															●					
**Slovak Republic**	**5.8**	**62.5**	**1985**	**1**	**72–96**	●	●						●	●		●	●	●		●		●	●	●	●	●	●	●	●	●	●	●		●	●	●
**Slovenia**	**2.1**	**18.5**	**1979**	**1**	**48–72**	●									**3**	●	●	●				●	●	●	●	●		●	●	●	●	●		●	●	●
**Spain**	**47.5**	**351.9**	**1968**	**15**	**24–72**	●	🞇	🞇	🞇	●		🞇	●	**†**	**1, 4**	●	🞇	🞇	🞇	🞇	🞇	🞇		●	●	🞇	🞇	🞇	🞇	🞇	🞇	●	🞇	🞇	🞇	🞇
**Sweden**	**10.6**	**113.7**	**1965**	**1**	**48–72**	●	●	●	●			●		●	**1**	●	●	●	●	●	●	●	●	●	●	●		●	●	●	●	●		●		
**Switzerland**	**8.8**	**86.1**	**1965**	**1**	**72–96**	●	●	●	●			●	●	**2**		●	●							●								●				
**Tajikistan**	**10.1**	**256.6**																																		
**Turkey (Türkiye)**	**85.8**	**1214**	**1983**	**2**	**48–72**	●	●		●				●	●		●																				
**Turkmenistan**	**6.5**	**130.7**		**1**	**48–72**	●	●	●					●			●																				
**Ukraine**	**36.7**	**184.5**	**2001**	**7**	**48–72**	●	●	🞇	🞇				●	●	**1**	●	🞇	🞇	🞇	🞇	🞇	🞇	🞇	🞇	🞇	🞇		🞇	🞇	🞇		🞇		🞇		🞇
**United Kingdom**	**67.7**	**677.7**	**1969**	**16**	**120**	●				●			●	**†**		●	●				●			●					●			●				
**Uzbekistan**	**35.2**	**755.1**		**14**	**72–96**	●			🞇				🞇			●	🞇	🞇	🞇	🞇	🞇	🞇		🞇	🞇	🞇		🞇	🞇	🞇	🞇	🞇				🞇
**Total**	**923.8**	**9886.9**																																		
**● More than 50% coverage; part of national plan/law** **🞇 Less than 50% coverage; part of national plan/law** **†** **Only available on limited basis—pilot, contracted service of specific birthing facility, etc. not generally available to all public** **^a^ https://data.unicef.org/resources/data_explorer/unicef_f/?ag=UNICEF&df=GLOBAL_DATAFLOW&ver=1.0&dq=.DM_POP_TOT..&startPeriod=2023&endPeriod=2023 (accessed 27 March 2024)** **^b^ https://data.unicef.org/resources/data_explorer/unicef_f/?ag=UNICEF&df=GLOBAL_DATAFLOW&ver=1.0&dq=.DM_BRTS.&startPeriod=2023&endPeriod=2023 (accessed 27 March 2024)** **^c^ See Reference [666]. Available online: https://www.mdpi.com/2409-515X/7/1/15 (accessed 27 May 2023)** **1 = GA-2 (pilot in Spain and Ukraine)** **2 = SMA pilot completed; awaiting decision on implementation: see details at https://old.sma-screening-alliance.org/map/ (accessed 21 January 2024)** **3 = ALD (pilot in Slovenia and Iceland)** **4 = RMD (pilot in Spain)**																																				

**Table 8:** Summary Newborn Screening Data from Latin America and the Caribbean.

Demographic Information								Disorder																													
								Miscellaneous									Amino Acid						Fatty Acid Oxidation							Organic Acid							
Country	^a^ Population (Millions) 2023 UNICEF Data 0	^b^ Births (Thousands) 2023 UNICEF Data	^c^ Infant (<1 Year) Mortality Rate (per 1000) 2022	Date Screening Began (P) = Public pilot (N) = National (L) = Limited (X) = Private		Screening Laboratories	Specimen Collection Time (Hrs.)	CH	CAH	GAL	BIO	HGB	G6PD	SCID	CF	Other (see key below)	PKU	MSUD	TYR-I	ASA	CIT	HCY	CUD	CACT	MCAD	LCHAD/TFP	VLCAD	SCAD	CPT- I/CPT -II	IVA	MMA (MUT)/(CBL)	PA	GA-I	MCD	BKT	HMG	3-MCC
**Latin America**																																					
**Argentina**	**45.8**	**624.9**	**8.4**	**1983 (P) 1995 (R)**	**2006 (N)**	**23**	**Var.**	●	●	●	●				●		●	●							🞇												
**Bolivia**	**12.4**	**263.9**	**19.6**	**1993 (P)**	**2008 (R)**	**9+**		**🞇**	**†**						**†**		**†**																				
**Brazil**	**216.4**	**2700.0**	**12.5**	**1976 (P)**	**2001(N)**	**30**	**48–72**	●	●	**†**	●	●		**†**	●	**1**	🞇	🞇	🞇	🞇	🞇	🞇	🞇	🞇	🞇	🞇	🞇	🞇	🞇	🞇	🞇	🞇	🞇	🞇	🞇	🞇	🞇
**Chile**	**19.6**	**226.3**	**5.4**	**1989 (P)**	**1992 (N)**	**2**	**40–48**	●									●																				
**Colombia**	**52.1**	**714.4**	**10.6**	**1986 (P)**	**2000 (N)**	**§**	**>24**	●	**†**	**†**	**†**	**†**			**†**		**†**																				
**Costa Rica**	**5.2**	**60.1**	**6.9**	**1982 (L)** **1988 (P)**	**1990 (N)**	**1**		●	●	●		●		●		**2**	●	●							●	●	●	●	●	●	●	●	●		●	●	●
**Cuba**	**11.2**	**98.9**	**6.2**		**1986 (N)**	**175+**		●	●	●	●				●		●																				
**Dominican Republic**	**11.3**	**200.6**	**26.8**	**1989 (L)**				**†**	**†**	**†**	**†**		**†**		**†**		**†**	**†**	**†**	**†**	**†**	**†**	**†**	**†**	**†**	**†**	**†**	**†**	**†**	**†**	**†**	**†**	**†**	**†**	**†**	**†**	**†**
**Ecuador**	**18.2**	**298.5**	**10.5**	**1987 (X)**	**2011 (N)**	**1?**		●	●	●							●																				
**El Salvador**	**6.4**	**99.7**	**10.2**	**2007 (P)**	**2008 (R)**		**>24**	●	**†**	**†**					**†**		**†**																				
**Guatemala**	**18.1**	**373.4**	**18.8**	**1995 (L)**	**2003 (L)**	**2**		🞇	**†**	**†**					**†**		**†**																				
**Haiti**	**11.7**	**267.6**	**44.0**	**2010 (P)**								**†**																									
**Honduras**	**10.6**	**217.2**	**13.8**	**2017 (R)**		**1**		🞇	🞇	🞇		**†**			🞇		🞇																				
**Mexico**	**128.5**	**1857.0**	**11.0**	**1973 (P)**	**1988 (L)** **1995 (R)**	**§**	**72–120**	●	●	●	●		●		●	**3**	●	**†**	**†**	**†**	**†**	**†**	**†**	**†**	**†**	**†**	**†**	**†**	**†**	**†**	**†**	**†**	**†**	**†**	**†**	**†**	**†**
**Nicaragua**	**7.0**	**138.1**	**13.1**	**2005 (R)**	**2014 (N)**																																
**Panama**	**4.5**	**76.6**	**11.4**	**2007 (P)**	**2023 (N)**	**2**		●	●	●		●	●		●		●																				
**Paraguay**	**6.9**	**137.4**	**15.0**	**1999 (L)**	**2003 (N)**	**1**	**24–48**	●							●		●																				
**Peru**	**34.4**	**588.9**	**11.7**	**2002 (X)**	**2012 (N)**	**3+**	**48–72**	🞇	🞇						🞇		🞇																				
**Uruguay**	**3.4**	**35.6**	**5.6**	**1990 (L)**	**1994 (N)**	**1**	**40**	●	●			●			●	**4**	●	●							●	●	●	●	●	●	●	●	●	●	●	●	●
**Venezuela**	**28.8**	**454.9**	**21.2**	**1985 (P)** **1999 (R)**	**2013 (N)**	**2+**		●	●	●	●						●																				
**Total**	**652.5**	**9434.0**					**Var.**																														
**Caribbean**																																					
**Anguilla**	**0.02**	**0.15**	**5.4**																																		
**Antigua and Barbuda**	**0.1**	**1.12**	**8.1**	**2020 (P)**								**†**																									
**Aruba**	**0.1**	**0.77**	**n/a**	**2006(L)**								**†**																									
**Bahamas**	**0.4**	**4.67**	**11.2**					**†**	**†**	**†**	**†**	**†**	**†**			**5**	**†**	**†**	**†**	**†**	**†**	**†**	**†**	**†**	**†**	**†**	**†**	**†**	**†**	**†**	**†**	**†**	**†**	**†**	**†**	**†**	**†**
**Barbados**	**0.3**	**3.01**	**10.1**	**1999 (L)**				**†**	**†**	**†**	**†**	**†**	**†**				**†**	**†**	**†**	**†**	**†**	**†**	**†**	**†**	**†**	**†**	**†**	**†**	**†**	**†**	**†**	**†**	**†**	**†**	**†**	**†**	**†**
**Belize**	**0.4**	**7.24**	**9.4**																																		
**British Virgin Islands**	**0.03**	**0.24**	**11.6**																																		
**Cayman Islands**	**0.07**	**0.65**	**n/a**	**1997 (L)**	**2002 (N)**			**†**	**†**	**†**	**†**	**†**	**†**			**5**	**†**	**†**	**†**	**†**	**†**	**†**	**†**	**†**	**†**	**†**	**†**	**†**	**†**	**†**	**†**	**†**	**†**	**†**	**†**	**†**	**†**
**Curaçao**	**0.2**	**2.20**	**n/a**	**2006 (L)**				**🞇**				**🞇**					**†**																				
**Dominica**	**0.1**	**0.96**	**30.8**																																		
**French Guiana**	**0.3**	**7.66**	**n/a**					●	●	●	●	●	●			6	●	●	●						●	●	●			●	●	●	●				
**Grenada**	**0.1**	**1.92**	**14.4**	**2014 (P)**								**†**																									
**Guadeloupe**	**0.4**	**4.59**	**n/a**	**1984 (P)**	**1992 (N)**							●																									
**Guyana**	**0.8**	**15.94**	**22.3**	**2016 (L)**								**†**																									
**Jamaica**	**2.8**	**32.25**	**16.1**		**1995 (N)**							●																									
**Martinique**	**0.4**	**3.53**	**n/a**		**1989 (N)**							●																									
**Montserrat**	**0.01**	**0.04**	**5.7**																																		
**Sint Maarten**	**0.04**	**0.40**	**n/a**	**2006 (L)**								**†**																									
**St. Kitts and Nevis**	**0.05**	**0.56**	**13.3**																																		
**St. Lucia**	**0.2**	**2.01**	**16.0**	**1986 (L)**	**1992 (N)**							🞇																									
**St. Vincent and the Grenadines**	**0.05**	**1.31**	**10.1**	**2015 (L)**								**†**																									
**Suriname**	**0.6**	**11.09**	**14.8**																																		
**Trinidad & Tobago**	**1.5**	**17.01**	**13.8**		**2008 (N)**							🞇																									
**Turks and Caicos**	**0.05**	**0.55**	**4.1**					**†**	**†**	**†**		**†**	**†**																								
**Total**	**9.02**	**119.87**																																			
**● More than 50% coverage; part of national implementation/law** 🞇** Less than 50% coverage; part of national implementation/law** **†** **Only available on limited basis—pilot, contracted service of specific birthing facility, etc.; not generally available to all public** **§ Complex laboratory services. Colombia decentralized through private clinical providers; Mexico has a variety of NBS providers** **^a^ https://data.unicef.org/resources/data_explorer/unicef_f/?ag=UNICEF&df=GLOBAL_DATAFLOW&ver=1.0&dq=.DM_POP_TOT..&startPeriod=2023&endPeriod=2023 (accessed 27 March 2024)** **^b^ https://data.unicef.org/resources/data_explorer/unicef_f/?ag=UNICEF&df=GLOBAL_DATAFLOW&ver=1.0&dq=.DM_BRTS.&startPeriod=2023&endPeriod=2023 (accessed 27 March 2024)** **^c^ https://data.unicef.org/resources/data_explorer/unicef_f/?ag=UNICEF&df=GLOBAL_DATAFLOW&ver=1.0&dq=.CME_MRY0._T.&startPeriod=2021&endPeriod=2023 (accessed 27 March 2024)** **Var. = Variable recommendations: National program (48 h to 120 h); Buenos Aires Province (24 h after first milk 0 120 h); Buenos Aires Autonomous (48 h to 72 h)** **1 = Limited screening for SMA, some LSDs, Prader-Willi, Angelman syndrome** **2 = Also includes GA-2** **3 = Complex system—policies set by National Center for Gender Equity and Reproductive Health of the Ministry of Health (Centro Nacional de Equidad de Género y Salud Reproductiva).** **4 = Also includes HHH, argininemia** **5 = expanded newborn screening available from overseas private laboratory** **6 = specimens sent to France and get French screening panel, including hemoglobinopathies**																																					

**Table 9:** Summary Newborn Screening Data from the Middle East—North Africa.

Demographic Information									Disorder																													
									Miscellaneous									Amino Acid						Fatty Acid Oxidation							Organic Acid							
Country		^a^ Population (Millions) 2023 UNICEF Data	^b^ Births (Thousands) 2023 UNICEF Data	^c^ Est. Birth Rate (Births/1000 Pop.) 2024	^d^ Infant (<1 Year) Mortality Rate (per 1000) 2022	^e^ Date Screening Began	Screening Laboratories	Specimen Collection Time (Hrs.)—2021	CH	HGB	GAL	BIO	CAH	G6PD	SCID	CF	Other (see key below)	PKU	MSUD	TYR-I	ASA	CIT	HCY	CUD	CACT	MCAD	LCHAD/TFP	VLCAD	SCAD	CPT- I/CPT -II	IVA	MMA (MUT)/(CBL)	PA	GA-I	MCD	BKT	HMG	3-MCC
**Algeria**		**45.6**	**895.4**	**20.2**	**18.7**	--			**†**																													
**Bahrain**		**1.5**	**16.6**	**12.2**	**5.6**	**2007**	**1**			**§** **†**		**†**	●	●		**†**	**1**		**†**	**†**			**†**			**†**												
**Egypt, Arab Republic**		**112.7**	**2,449.0**	**19.5**	**15.5**	**2000**	**1+1**	**24–48**	●	**§**	🞇	🞇	🞇	🞇		**†**	**2**	●	🞇	🞇	🞇	🞇	🞇			🞇	🞇	🞇			🞇	🞇	🞇	🞇		🞇	🞇	
**Iran, Islamic Republic**		**89.2**	**1,145.0**	**14.3**	**10.3**	**2005**	**10**	**72–120**	●	**§**	🞇	🞇					**3**	●	🞇	🞇	🞇	🞇	🞇	🞇		🞇					🞇	🞇	🞇	🞇	🞇	🞇	🞇	🞇
**Iraq**		**45.5**	**1,216.0**	**23.7**	**20.0**	**2013**	**2+1**	**72–120**	🞇	**§** **†**	**🞇**	**†**	**†**	**†**	**†**	**†**	**4**	**🞇**	**†**	**†**	**†**	**†**	**†**	**†**	**†**	**†**	**†**	**†**	**†**	**†**	**†**	**†**	**†**	**†**	**†**	**†**	**†**	**†**
**Israel**		**9.2**	**174.6**	**19.1**	**2.7**	**1964**		**>36**	●	●	●				●			●	●	●			●			●		●				●	●	●				
**Jordan**		**11.3**	**243.2**	**22.2**	**12.2**	**2004**	**1**	**72–96**	●	**§**	**†**	**†**		●				●																				
**Kuwait**		**4.3**	**39.9**	**17.5**	**7.4**	**2005**	**1**	**24–48**	●	●	●	●						●	●	●	●	●	●		●	●	●	●			●	●	●	●	●	●	●	●
**Lebanon**		**5.4**	**76.1**	**12.6**	**14.9**	**1996**	**3**	**24–48**	●	**§**	🞇			●	🞇			●	●		🞇	●	●			🞇	🞇	🞇		🞇	🞇	🞇	🞇	🞇		🞇	🞇	
**Libya**		**6.9**	**116.4**	**20.3**	**8.9**	**--**																																
**Morocco**		**37.8**	**637.5**	**16.8**	**14.8**	**2010**	**6**	**24–48**	🞇																													
**Oman**		**4.6**	**79.5**	**21.1**	**9.0**	**2005**	**1**	**24–48**	●																													
**Palestine**	**Gaza**	**5.4**	**146.6**	**26.8**	**12.3**	**1994**			●	**§**								●																				
	**West Bank**			**27.8**		**1994**			●	**§**								●																				
**Qatar**		**2.7**	**23.9**	**9.2**	**4.5**	**1996**	**1**	**24–48**	●	●	●	●	●		●		**5**	●	●	●	●	●	●		●	●	●	●	●	●	●	●	●	●		●	●	●
**Saudi Arabia**		**36.9**	**606.7**	**13.6**	**5.5**	**1980**	**1+7**	**24–48**	●	**§**●	●	●		●			**6**	●	●	●		●				●		●			●	●	●	●		●	●	●
**Syrian Arab Republic**		**23.2**	**490.8**	**21.7**	**17.8**	**--**	**1**		🞇									🞇																				
**Tunisia**		**12.5**	**188.3**	**13.5**	**9.9**	**--**			**†**																													
**United Arab Emirates**		**9.5**	**91.8**	**10.7**	**4.3**	**1995**	**5**	**24–48**	●	**§**●	●	●	●	●	**§**	**†**		●	●	●	●	●	●	●	●	●	●	●	●	●	●	●	●	●	●	●	●	●
**Yemen**		**34.4**	**1012.0**	**23.4**	**33.1**	**--**																																
**Totals**		**499.4**	**9665.2**																																			
**Abbreviations: n/a = not available** **● More than 50% coverage; part of national implementation/law** **🞇 Less than 50% coverage; part of national implementation/law** **† Only available on limited basis—pilot, contracted service of specific birthing facility, etc.; not generally available to all public** **§ an extensive national prenatal screening program exists** **^a^ https://data.unicef.org/resources/data_explorer/unicef_f/?ag=UNICEF&df=GLOBAL_DATAFLOW&ver=1.0&dq=.DM_POP_TOT..&startPeriod=2023&endPeriod=2023 (accessed 27 March 2024)** **^b^ https://data.unicef.org/resources/data_explorer/unicef_f/?ag=UNICEF&df=GLOBAL_DATAFLOW&ver=1.0&dq=.DM_BRTS.&startPeriod=2023&endPeriod=2023 (accessed 27 March 2024)** **^c^ https://www.cia.gov/the-world-factbook/field/birth-rate/country-comparison/ (accessed 27 March 2024)** **^d^ https://data.unicef.org/resources/data_explorer/unicef_f/?ag=UNICEF&df=GLOBAL_DATAFLOW&ver=1.0&dq=.CME_MRY0._T.&startPeriod=2021&endPeriod=2023 (accessed 27 March 2024)** **^e^ In some cases there may be a single “main” screening laboratory(ies) with smaller laboratories operating in some regions of the country—first number indicates number of “main” laboratory(ies)** **1 = Tests marked with † are available from Saudi Arabia laboratories** **2 = SMA** **3 = GA II, NKH, UCD** **4 = SMA, Pompe, Gaucher, Krabbe, MPS I, Fabry and Neiman Pick A&B** **5 = IBG, CTD, MADD** **6 = ASL**																																						

**Table 10:** Summary Newborn Screening Data from Sub-Saharan Africa.

Demographic Information										Disorders Screened					
Country	^a^ Population (Millions) 2023 UNICEF Data	^b^ Births (Thousands) 2023 UNICEF Data	^c^ Est. Birth Rate 2024 (Births/1000 Pop.)	^c^ Global Rank Birth Rate 2024	^d^ Global Rank Poorest Countries 2024	^e^ Child (<5 Year) Mortality Rate (per 1000) 2022	^f^ Infant (<1 Year) Mortality Rate (per 1000) 2022	^g^ Sickle Cell Disease Births—2000	^g^ Sickle Cell Disease Births—2021	HGB	CH	CAH	G6PD	CF	Other (See Footnotes)
**Angola**	**36.7**	**1382.0**	**41.1**	**2**	**52**	**66.8**	**45.7**	**8930**	**13,700**	**†**					
**Benin**	**13.7**	**487.5**	**40.3**	**3**	**36**	**80.8**	**53.6**	**10,600**	**13,400**	**†**					
**Botswana**	**2.7**	**60.2**	**19.6**	**72**	**104**	**38.7**	**31.2**	**20**	**21**	**No Information**					
**Burkina Faso**	**23.3**	**801.0**	**31.9**	**22**	**16**	**78.8**	**50.1**	**18,100**	**22,000**	**†**					
**Burundi**	**13.2**	**443.1**	**34.6**	**14**	**2**	**50.5**	**36.4**	**188**	**316**	**†**					
**Cabo Verde**	**0.6**	**9.8**	**17.9**	**79**	**64**	**12.3**	**10.6**	**15**	**12**	**No Information**					
**Cameroon**	**28.6**	**967.7**	**34.7**	**13**	**40**	**69.8**	**47.0**	**5050**	**7310**	**†**					
**Central African Republic**	**5.7**	**243.5**	**31.9**	**21**	**3**	**96.8**	**73.5**	**1420**	**1790**	**†**					
**Chad**	**18.3**	**779.3**	**39.2**	**7**	**13**	**102.8**	**64.1**	**940**	**1790**	**No Information**					
**Comoros**	**0.9**	**24.2**	**21.6**	**58**	**28**	**48.2**	**38.2**	**1**	**1**	**Interest but No Program**					
**Congo, Democratic Republic**	**102.3**	**4237.0**	**39.2**	**6**	**4**	**75.6**	**60.1**	**36,900**	**47,400**	**†**					
**Congo, Republic**	**6.1**	**182.2**	**28.7**	**30**	**39**	**41.6**	**31.2**	**782**	**851**	**†**					
**Cote d’Ivoire**	**28.9**	**960.0**	**27.5**	**35**	**49**	**69.4**	**52.4**	**5080**	**6050**	**†**					
**Djibouti**	**1.1**	**24.6**	**21.8**	**56**	**57**	**51.9**	**44.1**	**6**	**8**	**No Information**					
**Equatorial Guinea**	**1.7**	**50.3**	**29.0**	**28**	**95**	**73.4**	**55.1**	**663**	**801**	**†**			**†**		
**Eritrea**	**3.7**	**106.3**	**26.3**	**41**	**n/a**	**36.6**	**28.0**	**42**	**49**	**No Information**					
**Eswatini (Swaziland)**	**1.2**	**28.4**	**22.3**	**51**	**76**	**50.0**	**39.7**	**1**	**1**	**🞇**					
**Ethiopia**	**126.5**	**3965.0**	**29.6**	**27**	**33**	**46.2**	**33.9**	**147**	**184**	**Interest but No Program**					
**Gabon**	**2.4**	**63.8**	**25.7**	**43**	**99**	**38.8**	**28.8**	**439**	**412**	**🞇**					
**Gambia**	**2.8**	**89.5**	**27.3**	**36**	**20**	**45.6**	**32.8**	**414**	**443**	**No Information**					
**Ghana**	**34.1**	**909.9**	**27.6**	**33**	**53**	**42.3**	**31.6**	**11,900**	**13,700**	**🞇**					
**Guinea**	**14.2**	**473.4**	**35.3**	**12**	**25**	**96.0**	**62.2**	**6560**	**9200**	**†**					
**Guinea-Bissau**	**2.2**	**64.8**	**36.0**	**11**	**23**	**71.9**	**48.6**	**37**	**41**	**†**			**†**		
**Kenya**	**55.1**	**1503.0**	**25.6**	**44**	**51**	**41.1**	**30.5**	**15,600**	**18,100**	**🞇**	**†**	**†**		**†**	**1**
**Lesotho**	**2.3**	**59.4**	**22.9**	**50**	**22**	**72.2**	**56.4**	**2**	**1**	**No Information**					
**Liberia**	**5.4**	**167.0**	**32.4**	**20**	**8**	**73.2**	**54.9**	**669**	**682**	**🞇**					
**Madagascar**	**30.3**	**918.2**	**27.6**	**34**	**9**	**65.8**	**45.1**	**2530**	**3240**	**†**					
**Malawi**	**20.9**	**679.0**	**26.6**	**40**	**7**	**40.1**	**30.1**	**404**	**422**	**†**			**†**		
**Mali**	**23.3**	**952.5**	**40.0**	**4**	**15**	**93.8**	**60.1**	**3620**	**6720**	**†**					
**Mauritania**	**4.9**	**158.7**	**27.2**	**37**	**56**	**39.2**	**31.4**	**208**	**221**	**†**			**†**		
**Mauritius**	**1.3**	**13.2**	**9.8**	**191**	**126**	**15.0**	**13.4**	**0**	**0**	**†**	**†**	**†**		**†**	**2**
**Mozambique**	**33.9**	**1208.0**	**36.5**	**9**	**5**	**66.2**	**48.7**	**155**	**220**	**No Information**					
**Namibia**	**2.6**	**69.2**	**24.3**	**47**	**72**	**37.9**	**28.6**	**2**	**2**	**†**					
**Niger**	**27.2**	**1217.0**	**46.6**	**1**	**6**	**117.3**	**60.3**	**9640**	**14,500**	**No Information**					
**Nigeria**	**223.8**	**8109.0**	**33.8**	**17**	**46**	**107.2**	**68.5**	**145,000**	**179,000**	**🞇**	**†**				
**Rwanda**	**14.1**	**409.8**	**25.0**	**46**	**26**	**38.0**	**28.8**	**76**	**84**	**†**					
**Sao Tome and Principe**	**0.2**	**6.4**	**26.7**	**39**	**34**	**14.5**	**11.3**	**54**	**46**	**No Information**					
**Senegal**	**17.8**	**562.5**	**30.2**	**25**	**37**	**37.0**	**28.2**	**2160**	**2620**	**🞇**					
**Seychelles**	**0.1**	**1.6**	**11.8**	**152**	**137**	**14.5**	**12.5**	**0**	**0**	**🞇**	**🞇**	**🞇**		**🞇**	**3**
**Sierra Leone**	**8.8**	**266.8**	**30.8**	**24**	**12**	**100.8**	**76.0**	**5040**	**7250**	**Pilot Data but No Program**					
**Somalia**	**18.1**	**774.6**	**37.4**	**8**	**11**	**106.1**	**68.0**	**27**	**48**	**No Information**					
**South Africa**	**60.4**	**1141.0**	**17.7**	**72**	**85**	**34.5**	**27.7**	**19**	**18**		**†**				**4**
**South Sudan**	**11.1**	**321.4**	**36.4**	**10**	**1**	**98.8**	**63.8**	**97**	**105**	**No Information**					
**Sudan**	**48.1**	**1567.0**	**33.1**	**18**	**27**	**51.6**	**37.0**	**9070**	**5870**	**Studies but No Program**					
**Tanzania, United Republic of**	**67.4**	**2385.0**	**32.5**	**19**	**31**	**40.5**	**30.0**	**9600**	**13,100**	**🞇**					
**Togo**	**9.1**	**282.0**	**30.9**	**23**	**17**	**60.4**	**42.1**	**4200**	**4870**	**†**					
**Uganda**	**48.6**	**1729.0**	**39.6**	**5**	**24**	**40.5**	**30.2**	**7250**	**9410**	**🞇**					
**Zambia**	**20.6**	**694.8**	**34.1**	**16**	**35**	**55.6**	**39.0**	**3420**	**4700**	**🞇**					
**Zimbabwe**	**16.7**	**493.8**	**28.8**	**29**	**19**	**47.7**	**34.6**	**71**	**68**	**Interest but No Program**					
**SSA Total**	**~1212**	**42,043.4**						**327,149**	**410,777**						
**Global Total**	**~8000**	**134,280.0**						**453,000**	**515,000**						
**🞇 Less than 50% coverage; part of national implementation/law** ****†** Only available on limited basis—pilot, contracted service of specific birthing facility, etc.; not generally available to all public** **^a^ https://data.unicef.org/resources/data_explorer/unicef_f/?ag=UNICEF&df=GLOBAL_DATAFLOW&ver=1.0&dq=.DM_POP_TOT..&startPeriod=2023&endPeriod=2023 (accessed 27 March 2024)** **^b^ https://data.unicef.org/resources/data_explorer/unicef_f/?ag=UNICEF&df=GLOBAL_DATAFLOW&ver=1.0&dq=.DM_BRTS.&startPeriod=2023&endPeriod=2023 (accessed 27 March 2024)** **^c^ https://www.cia.gov/the-world-factbook/field/birth-rate/country-comparison/ (accessed 27 March 2024)** **^d^ https://www.gfmag.com/global-data/economic-data/the-poorest-countries-in-the-world According to gross domestic product per capita – purchasing power parity) (accessed 27 March 2024)** **^e^ https://data.unicef.org/resources/data_explorer/unicef_f/?ag=UNICEF&df=GLOBAL_DATAFLOW&ver=1.0&dq=.CME_MRY0T4._T&startPeriod=2021&endPeriod=2023 (accessed 27 March 2024)** **^f^ https://data.unicef.org/resources/data_explorer/unicef_f/?ag=UNICEF&df=GLOBAL_DATAFLOW&ver=1.0&dq=.CME_MRY0._T.&startPeriod=2021&endPeriod=2023 (accessed 27 March 2024)** **^g^ See reference** [1444]**. Supplemental Table S9, pp. 69–95. https://www.thelancet.com/journals/lanhae/article/PIIS2352-3026(23)00118-7/fulltext#sec1 (accessed 3 February 2024)** **1 = Commercial laboratory services available (FirstScreen). Other advertised conditions: Multiple Acyl-CoA Dehydrogenase Deficiency (MADD or Glutaric Acidemia II)** **2 = PKU** **3 = Pilot also includes PKU and GAL, 6 conditions total** **4 = Various Inborn Errors of Metabolism (IEM) conditions available through MS/MS testing at North-West University Potchestroom IEM Laboratory**															

## Abbreviations

2M3HBA	2-Methyl 3-hydroxy butyric aciduria (2-methyl-3-hydroxybutyryl CoA dehydrogenase deficiency)
2MBG	2-Methylbutyryl-CoA dehydrogenase deficiency
3MCC	3-Methylcrotonyl-CoA carboxylase deficiency
3MGA	3-Methylglutaconic aciduria (3-methylglutaconyl-CoA-hydratase deficiency)
22q11.2	22q11.2 deletion syndrome (DiGeorge syndrome)
5-OXO	Pyroglutamic acidemia
ADA-SCID	Adenosine deaminase-deficient SCID
ALD	Adrenoleukodystrophy
ARSA	Arylsulfatase AA
ASA	Argininosuccinic acidemia
ARG	Argininemia
BCKDK	Branched chain ketoacid dehydrogenase kinase deficiency
BIO	Biotinidase deficiency
BIOPT(BS)	Defects of biopterin cofactor biosynthesis
BIOPT(REG)	Defects of biopterin cofactor regeneration
BKT	β-Ketothiolase deficiency
CACT	Carnitine acylcarnitine translocase deficiency
CAH	Congenital adrenal hyperplasia
CBL A (or B) (or C) (or D)	Cobalamin A or B or C or D (Methylmalonic acidemia)
CCHD	Critical congenital heart disease
cCMV	Congenital cytomegalovirus
CF	Cystic fibrosis
CFSPID	Cystic fibrosis screen–positive with inconclusive diagnosis (also CRMS)
CRMS	Cystic fibrosis transmembrane conductance regulator related metabolic syndrome (also CFSPID)
CH	Congenital hypothyroidism
CPS-I	Carbamoyl phosphate synthetase deficiency—Type 1
CTD	Carnitine transport defect
CIT I (or II)	Citrullinemia Type 1 (or Type 2)
CPT I (or IA) (or II)	Carnitine palmitoyl transferase deficiency Type 1 (or Type 1A) (or Type 2)
CTX	Cerebrotendinous xanthomatosis
CUD	Carnitine uptake deficiency
DE RED	Dienoyl-CoA reductase deficiency
DMD	Duchenne muscular dystrophy
EHDI	Early hearing detection and intervention (newborn hearing screening)
EME	Ethylmalonic encephalopathy
FAOD	Fatty oxidation disorder
FIGLU	Formiminoglutamic aciduria
G6PD	Glucose-6-phosphate dehydrogenase
GA-I (or II)	Glutaric acidemia Type 1 (or Type 2)
GAL	Galactosemia—non-specific—sometimes used in the enabling legislation for screening. Use of the abbreviation in the text corresponds to the author’s use in the referenced report.
GALE	Galactosemia Type 3 (OMIM 230350) epimerase deficiency
GALK	Galactosemia Type 2 (OMIM 230200) kinase deficiency
GALM	Galactosemia Type 4 (OMIM 618881) mutarotase deficiency
GALT	Galactosemia Type 1 (OMIM 230350) transferase deficiency
GAMT	Guanidinoacetate methyltransferase deficiency
HGB	Hemoglobinopathies—nonspecific, all inclusive—usually used to describe general newborn screening for all hemoglobin disorders
HHH	Hyperornithinemia–hyperammonemia–homocitrullinuria syndrome
Hb H	Hemoglobin H disease (HbH disease)
H-PHE	Benign hyperphenylalaninemia
Hb S/β-Th (or Hb S/A)	Hemoglobin S/β-thalassemia (one of the sickle cell diseases)
Hb S/S	Hemoglobin S/S disease (sickle cell anemia—one of the sickle cell diseases)
Hb S/C	Hemoglobin S/C disease (one of the sickle cell diseases)
HCY	Homocystinuria
HIV	Human immunodeficiency virus
HMG	3-Hydroxy 3-methyl glutaric aciduria
Hyper ORN	Hyperornithinemia with gyrate deficiency
IBD	Inflammatory bowel disease
IBDD	Isobutyryl-CoA dehydrogenase deficiency
IVA	Isovaleric Acidemia
KD	Krabbe disease
LCHAD	Long-chain L-3-OH acyl-CoA dehydrogenase deficiency
LSD	Lysosomal storage disorder
MAL	Malonic acidemia
MCAD	Medium-chain acyl-CoA dehydrogenase deficiency
MCKAT	Medium-chain ketoacyl-CoA thiolase deficiency
MCD	Multiple carboxylase deficiency
MD 1	Myotonic dystrophy Type 1
MET	Hypermethioninemia
MLD	Metachromatic leukodystrophy
MMA	Methylmalonic acidemia (non-specific term describing the disease see CBL, MUT)
MPS-I (or II) (etc.)	Mucopolysaccharidosis Type 1 (or Type 2) (or others—3B, 4A, 6, etc.)
M/SCHAD	Medium/short-chain L3-OH acyl-CoA-dehydrogenase deficiency
MSUD	Maple syrup urine disease
MUT	Methylmalonic acidemia (mutase deficiency)
NHS	Newborn hearing screening (early hearing detection and intervention)
NICCD	Neonatal intrahepatic cholestasis caused by citrin deficiency
NKH	Nonketotic hyperglycinemia
OA	Organic aciduria
OTCD	Ornithine transcarbamylase deficiency
PA	Propionic acidemia
PCD	Primary carnitine deficiency
PD	Pompe disease
PID	Primary immunodeficiencies
PKU	Phenylketonuria
PRO	Prolinemia Type I/ Type II
PUCD	Proximal urea cycle disorder
SCADD	Short-chain acyl-CoA dehydrogenase deficiency
SCD	Sickle cell disease (a group of clinically significant sickle hemoglobin disorders)
SCID	Severe combined immunodeficiency
SMA	Spinal muscular atrophy
SW-CAH	Salt wasting congenital adrenal hyperplasia
TCL	T-cell lymphopenia
TFP	Trifunctional protein deficiency
TOXO	Congenital toxoplasmosis
TYR I (or II) (or III)	Tyrosinemia Type 1(or Type 2) (or Type 3)
Var Hb	Variant hemoglobinopathies (other than Hb S/S, Hb S/C, Hb S/A)
VLCAD	Very long-chain acyl-CoA dehydrogenase deficiency
XLA	X-linked agammaglobulinemia
